# Supplementary material for: Zn-Catalyzed Regioselective and Chemoselective Reduction of Aldehydes, Ketones and Imines
Source: Int J Mol Sci. 2022 Oct 21;23(20):12679. doi: 10.3390/ijms232012679 (PMC9604354; doi:10.3390/ijms232012679)
Supplement: Supplementary file 1 [file ijms-23-12679-s001.zip › ijms-1951930-supplementary.pdf]

Supporting Information

Zn-Catalyzed Regioselective and Chemoselective Reduction of Aldehydes, Ketones and Imines

Miaomiao Zhang, Hongmei Jiao, Haojie Ma, Ran Li, Bo Han,* Yuqi Zhang,* and Jijiang Wang

Laboratory of New Energy & New Function Materials and Shaanxi Key Laboratory of Chemical Reaction Engineering, College of Chemistry and Chemical Engineering, Yan’an University, Yan'an 716000, China

Table of Contents

1. Materials and Methods S1

2. Optimizing Reaction Parameters S1

3. The Preparation of Substrates S3

4. General Procedure for Zn-Catalyzed Regioselective and Chemoselective Reduction of Aldehydes S7

5. General Procedure for Zn-Catalyzed Regioselective and Chemoselective Reduction of Ketones S18

6.General Procedure for Zn-Catalyzed Regioselective and Chemoselective Reduction of α, *β*-Unsaturated Aldehydes/Ketones S25

7.General Procedure for Zn-Catalyzed Regioselective and Chemoselective Reduction of Imines S32

8. Gram-Scale Hydrogenation of 1a S35

9. Hg(0) Poisoning Experiments S35

10. Studying the Reaction profile of Zn-Catalyzed Reduction of 1a S35

11. Kinetic Experiments by Initial Rate Measurements for Zn-Catalyzed Reduction of 1a S37

12. Mechanistic control experiments S40

13. ^{1}H, ^{13}C and ^{19}F NMR Spectra S44

1. Materials and Methods

General. All reactions dealing with air or moisture-sensitive compounds were carried out in a flame-dried, sealed Schlenk reaction tube under an atmosphere of nitrogen. Analytical thin-layer chromatography was performed on glass plates coated with 0.25 mm 230–400 mesh silica gel containing a fluorescent indicator (Merck). Flash silica gel column chromatography was performed on silica gel 60N (spherical and neutral, 140–325 mesh) as described by Still. NMR spectra were measured on a Bruker AV-400 spectrometer and reported in parts per million. ^{1}H NMR spectra were recorded at 400 MHz in $CDCl_3$ or DMSO-d_6 were referenced internally to tetramethylsilane as a standard, and ^{13}C NMR spectra were recorded at 100 MHz and referenced to the solvent resonance.

Materials. Unless otherwise noted, materials and solvents were purchased from Tokyo Chemical Industry, Aldrich Inc., Alfa Aesar, Adamas-beta., and other commercial suppliers and used as received. $Zn(OAc)_2$ (99.5%), $ZnCl_2$ (98%) were purchased from Innochem and used as received. $CuCl_2$ (98%), $Zn(OTf)_2$ (99%) were purchased from Adamas-beta. and used as received. $CoBr_2$ (97%), $Mn(CO)_5Br$ (98%), $CrCl_3$ (99%), $NiBr_2$ (99%) were purchased from Aldrich Inc. and used as received. $FeCl_2$ (99.5%) were purchased from Aladdin and used as received. Aldehyde and ketone derivatives were purchased or were prepared according the known procedures.[1]

2. Optimizing Reaction Parameters

Table S1. Investigation of the Effect of Meatals on Regioselective Hydrogenation of 2-Naphthaldehyde.[a]

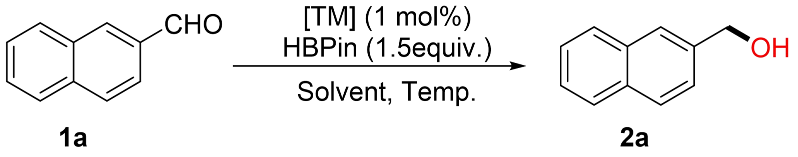

Entry	Catalyst	[H]	Solvent	Temp./℃	Time/h	Yield/%
1	$ZnCl_2$ (10mol%)	HBpin	THF	45	24	44
2	$Zn(OTf)_2$ (10mol%)	HBpin	THF	45	24	44
3	$Zn(OAc)_2$ (10mol%)	HBpin	THF	45	24	94

4	$Zn(OAc)_2$ (10mol%)	$PhSiH_3$	THF	45	24	nd
5	$Zn(OAc)_2$ (1 mol%)	HBpin	THF	rt	1	93
6	$FeCl_2$ (1mol%)	HBpin	THF	rt	1	58
7	$CoBr_2$ (1mol%)	HBpin	THF	rt	1	71
8	$CrCl_3$ (1mol%)	HBpin	THF	rt	1	nd[b]
9	$CuCl_2$ (1mol%)	HBpin	THF	rt	1	nd[b]
10	$NiBr_2$ (1mol%)	HBpin	THF	rt	1	41
11	$Mn(CO)_5Br$ (1mol%)	HBpin	THF	rt	1	80

[a]Conditions: 1a (0.2 mmol), Solvent (2 mL), and HBpin (1.5eq) , Isolated yield. [b]Not detected.

Table S2. Investigation of the Effect of the Amount of $Zn(OAc)_2$ on Regioselective Hydrogenation of 2-Naphthaldehyde.[a]

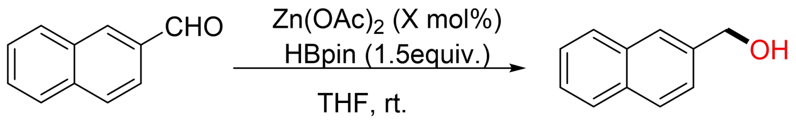

1a **2a**

Entry	$Zn(OAc)_2$ (X mol%)	Time/h	HBpin	Temp. (°C)	Yield of 2a (%)
1	1 mol%	24	1.5 equiv.	45	93
2	2 mol%	24	1.5 equiv.	45	93
3	5 mol%	24	1.5 equiv.	45	94

[a]Conditions: 1a (0.2 mmol), $Zn(OAc)_2$, Solvent (2 mL), and HBpin, Isolated yield.

Table S3. Investigation of the Effect of Solvents on Regioselective Hydrogenation of 2-Naphthaldehyde.[a]

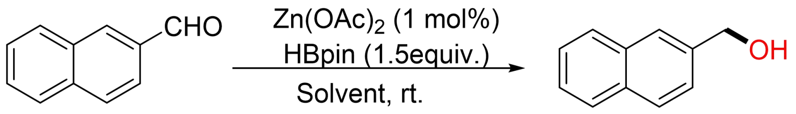

1a **2a**

Entry	Catalyst	[H]	Solvent	Temp./℃	Time/h	Yield/%
1	$Zn(OAc)_2$ (1mol%)	HBpin	THF	rt	1	93
2	$Zn(OAc)_2$ (1mol%)	HBpin	EtOH	rt	1	16
3	$Zn(OAc)_2$ (1mol%)	HBpin	Toluene	rt	1	41
4	$Zn(OAc)_2$ (1mol%)	HBpin	DCM	rt	1	48
5	$Zn(OAc)_2$ (1mol%)	HBpin	CH_3CN	rt	1	trace

6	$Zn(OAc)_2$ (1mol%)	HBpin	1,4-dioxane	rt	1	43

[a]Conditions: 1a (0.2 mmol), $Zn(OAc)_2$ (1mol%) , Solvent (2 mL), and HBpin (1.5equiv.) at room temperature for 1 h. Isolated yield.

Table S4. Investigation of the Effect of the Amount of HBpin on Regioselective Hydrogenation of 2-Naphthaldehyde.[a]

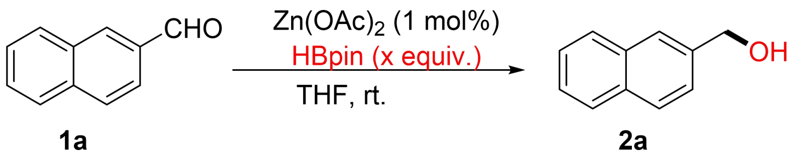

Entry	Catalyst	Time/h	HBpin (equiv.)	Solvent	Temp./°C	Yield of 2a %
1	$Zn(OAc)_2$	1	2	THF	25	93
2	$Zn(OAc)_2$	1	1.5	THF	25	93
3	$Zn(OAc)_2$	1	1	THF	25	66
4	$Zn(OAc)_2$	1	0.5	THF	25	28

[a]Conditions: 1a (0.2 mmol), $Zn(OAc)_2$ (1mol%) ,THF (2 mL),at rt for 1 h. Isolated yield.

3. The Preparation of Substrates

Preparation of Chalcone Compounds

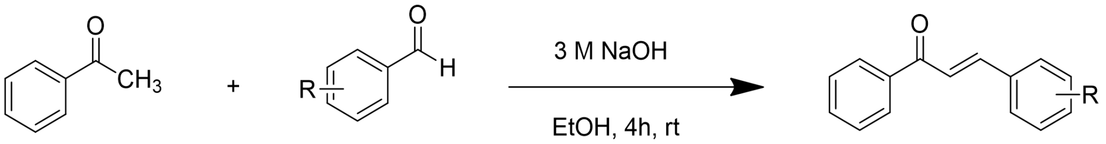

To a solution of the appropriate ketone (5mmol) in EtOH (3mL) at room temperature was added 3M NaOH (3mL). After 3 minutes, the appropriate aldehyde(5mmol) was added in one portion. The reaction was monitored by TLC. After completion, the filterated the mixture and solid was washed with amount of water and dried under vacuum. The product was purified by recrystallization in EtOH.[1]

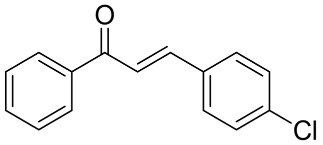

(*E*)-3-(4-chlorophenyl)-1-phenylprop-2-en-1-one

The title compound was obtained as a yellow solid (63% yield). ^{1}H NMR (400 MHz, $CDCl_3$): δ = 8.04-8.00 (m, 2H), 7.76 (d, J = 15.6 Hz, 1H), 7.61-7.56 (m, 3H),

7.54-7.48 (m, 3H), 7.40 (d, J = 8.4 Hz, 2H). ^{13}C NMR (100 MHz, $CDCl_3$): δ = 190.4, 143.5, 138.1, 136.6, 133.5, 133.1, 129.8, 129.4, 128.8, 128.7, 128.7, 122.6. The spectroscopic data are in accordance with those described in the literature.[2]

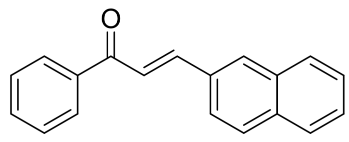

(*E*)-3-(naphthalen-2-yl)-1-phenylprop-2-en-1-one

The title compound was obtained as a yellow solid (60% yield).^{1}H NMR (400 MHz, $CDCl_3$): δ = 8.09-8.06 (m, 2H), 8.04 (s, 1H), 7.99 (d, J = 15.6 Hz, 1H), 7.87 (dq, J = 10.0, 5.2 Hz, 3H), 7.81 (dd, J = 8.6, 1.7 Hz, 1H), 7.66 (d, J = 15.6 Hz, 1H), 7.62-7.58 (m, 1H), 7.56-7.50 (m, 4H). ^{13}C NMR (100 MHz, $CDCl_3$): δ = 190.7, 145.1, 138.4, 134.5, 133.5, 132.9, 132.5, 130.8, 128.9, 128.8, 128.7, 127.9, 127.5, 126.9, 123.8, 122.3. The spectroscopic data are in accordance with those described in the literature.[3]

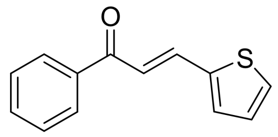

(*E*)-1-phenyl-3-(thiophen-2-yl)prop-2-en-1-one

The title compound was obtained as a yellow solid (80% yield).^{1}H NMR (400 MHz, $CDCl_3$): δ =8.01 (dd, J = 8.4, 1.3 Hz, 2H), 7.95 (dd, J = 15.4, 0.8 Hz, 1H), 7.61-7.56 (m, 1H), 7.51 (dd, J = 8.3, 7.0 Hz, 2H), 7.43 (dd, J = 5.0, 1.0 Hz, 1H), 7.38-7.31 (m, 2H), 7.11-7.08 (m, 1H). ^{13}C NMR (100 MHz, $CDCl_3$): δ = 190.1, 140.5, 138.2, 137.4, 132.9, 132.3, 129.0, 128.8, 128.6, 128.5, 120.9. The spectroscopic data are in accordance with those described in the literature.[4]

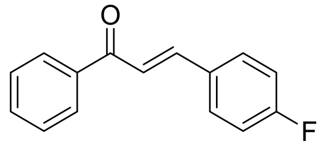

(*E*)-3-(4-fluorophenyl)-1-phenylprop-2-en-1-one

The title compound was obtained as a yellow solid (87% yield).^{1}H NMR (400 MHz, $CDCl_3$): δ = 8.02 (dd, J = 8.2, 1.4 Hz, 2H), 7.78 (d, J = 15.7 Hz, 1H), 7.64 (dd, J = 8.7,

5.5 Hz, 2H), 7.59 (d, J = 7.5 Hz, 1H), 7.54-7.44 (m, 3H), 7.12 (t, J = 8.6 Hz, 2H). ^{13}C NMR (100 MHz, $CDCl_3$): δ = 190.5, 164.2 (d, J = 251.7 Hz) 143.7, 138.2, 133.0, 131.3 (d, J = 3.2 Hz), 130.5 (d, J = 8.6 Hz), 128.7 (d, J = 18.0 Hz), 121.9 (d, J = 2.3 Hz), 116.3 (d, J = 22.1 Hz).^{19}F NMR (376 MHz, $CDCl_3$): δ = -108.88. The spectroscopic data are in accordance with those described in the literature.[5]

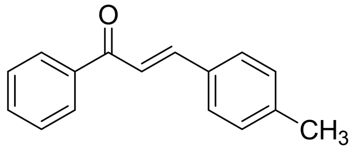

(*E*)-1-phenyl-3-(p-tolyl)prop-2-en-1-one

The title compound was obtained as a yellow solid (89% yield).^{1}H NMR (400 MHz, $CDCl_3$): δ = 7.95 (dd, J = 7.1, 1.4 Hz, 2H), 7.73 (d, J = 15.6 Hz, 1H), 7.52-7.40 (m, 6H), 7.19-7.14 (m, 2H), 2.32 (s, 3H). ^{13}C NMR (100 MHz, $CDCl_3$): δ = 190.9, 145.2, 141.3, 138.5, 132.9, 132.3, 129.9, 128.8, 128.7, 128.6, 121.2. 21.7. The spectroscopic data are in accordance with those described in the literature.[4]

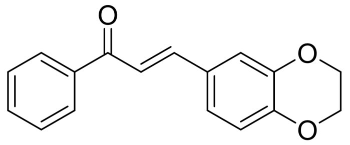

(*E*)-3-(2,3-dihydrobenzo[b][1,4]dioxin-6-yl)-1-phenylprop-2-en-1-one

The title compound was obtained as a yellow solid (87% yield).^{1}H NMR (400 MHz, $CDCl_3$): δ = 8.02-7.98 (m, 2H), 7.72 (d, J = 15.5 Hz, 1H), 7.60-7.55 (m, 1H), 7.50 (dd, J = 8.3, 6.7 Hz, 2H), 7.39 (d, J = 15.6 Hz, 1H), 7.20-7.14 (m, 2H), 6.90 (d, J = 8.3 Hz, 1H), 4.32-4.27 (m, 4H).^{13}C NMR (100 MHz, $CDCl_3$): δ = 190.7, 146.1, 144.8, 143.9, 138.5, 132.8, 128.7, 128.7, 128.6, 122.8, 120.4, 117.9, 117.2, 64.7, 64.4. The spectroscopic data are in accordance with those described in the literature.[6]

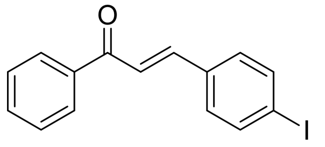

(*E*)-3-(4-iodophenyl)-1-phenylprop-2-en-1-one

The title compound was obtained as a yellow solid (77% yield).^{1}H NMR (400 MHz, $CDCl_3$): δ = 8.01 (dd, J = 7.2, 1.5 Hz, 2H), 7.79-7.70 (m, 3H), 7.64-7.57 (m, 1H), 7.56-7.48 (m, 3H), 7.37 (d, J = 8.4 Hz, 2H). ^{13}C NMR (100 MHz, $CDCl_3$): δ = 190.4, 143.697, 138.3, 138.1, 134.5, 133.1, 130.0, 128.8, 128.6,122.7, 97.0. The spectroscopic data are in accordance with those described in the literature.[7]

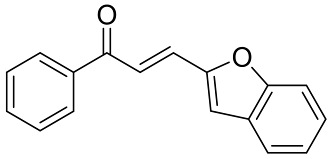

(*E*)-3-(benzofuran-2-yl)-1-phenylprop-2-en-1-one

The title compound was obtained as a yellow solid (93% yield).^{1}H NMR (400 MHz, $CDCl_3$): δ = 8.04-8.00 (m, 2H), 7.66-7.64 (m, 2H), 7.56-7.51 (m, 2H), 7.48-7.43 (m, 3H), 7.34-7.29 (m, 1H), 7.21-7.16 (m, 1H), 6.97 (s, 1H). ^{13}C NMR (100 MHz, $CDCl_3$): δ = 189.7, 155.7, 153.2, 138.0, 133.2, 131.0, 128.9, 128.7, 128.7, 126.9, 123.6, 122.0, 121.9, 112.8, 111.6. The spectroscopic data are in accordance with those described in the literature.[8]

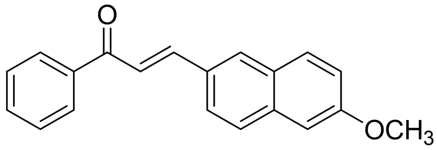

(*E*)-3-(6-methoxynaphthalen-2-yl)-1-phenylprop-2-en-1-one

The title compound was obtained as a yellow solid (91% yield).^{1}H NMR (400 MHz, $CDCl_3$): δ = 8.08-8.04 (m, 2H), 7.99-7.94 (m, 2H), 7.80-7.75 (m, 3H), 7.63-7.57 (m, 2H), 7.55-7.50 (m, 2H), 7.20-7.14 (m, 2H), 3.94 (s, 3H). ^{13}C NMR (100 MHz, $CDCl_3$): δ = 190.8, 159.1, 145.4, 138.6, 136.0, 132.9, 130.7, 130.4, 128.9, 128.8, 128.6, 127.7, 124.5, 121.2, 119.7, 106.1, 55.6. The spectroscopic data are in accordance with those described in the literature.[9]

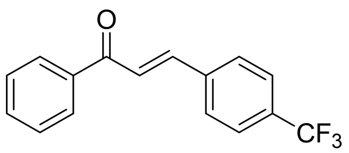

(*E*)-1-phenyl-3-(4-(trifluoromethyl)phenyl)prop-2-en-1-one

The title compound was obtained as a white solid (60% yield).^{1}H NMR (400 MHz, $CDCl_3$): δ = 8.05-8.01 (m, 2H), 7.81 (d, J = 15.6 Hz, 1H), 7.75 (d, J = 8.1 Hz, 2H), 7.68 (d, J = 8.2 Hz, 2H), 7.64-7.58 (m, 2H), 7.53 (ddd, J = 8.7, 6.8, 1.5 Hz, 2H). ^{13}C NMR (100 MHz, $CDCl_3$): δ = 190.1, 142.8, 137.9 (d, J = 49.0 Hz) 133.2, 128.8, 128.5 (d, J = 5.5 Hz), 125.9 (q, J = 4.5 Hz) 124.2.^{19}F NMR (376 MHz, $CDCl_3$): δ = -62.70. The spectroscopic data are in accordance with those described in the literature.[2]

4. General Procedure for Zn-Catalyzed Regioselective and Chemoselective Reduction of Aldehydes

A mixture of corresponding aldehyde derivatives (0.2 mmol) and $Zn(OAc)_2$ (0.002mmol) were added to Schlenk tube. Then HBpin (1.5 equiv) and THF (2 mL) were added by syringe under atmosphere of nitrogen. The reaction mixture was stirred at 25°C for 1 h. After quenching with saturated NH_4Cl/H_2O (10 mL). The crude product was extracted with EtOAc (3 × 10 mL). The combined organic phases were dried over anhydrous Na_2SO_4 and concentrated under vacuum, the crude product was purified by column chromatography to afford the desired hydrogenation compound.

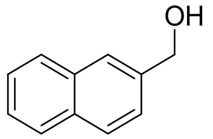

2-Naphthalenemethanol (2a)

The general procedure was applied to 2-naphthaldehyde (31.2 mg, 0.2 mmol) under an atmosphere of N_2 at 25 °C for 1 h. The crude product was purified by column chromatography on silica gel (Petroleum ether/EtOAc=5:1) to afford the title compound as white solid (29.3 mg, 93% yield); ^{1}H NMR (400 MHz, DMSO-d_6): δ = 7.84-7.79 (m, 3H), 7.76 (s, 1H), 7.44-7.38 (m, 3H), 5.29 (t, J = 5.7 Hz, 1H), 4.62 (q, J = 5.6 Hz, 2H); ^{13}C NMR (100 MHz, DMSO-d_6): δ = 140.2, 132.9, 132.2, 127.6, 127.6, 127.6, 126.1, 125.5, 125.3, 124.3, 63.0. The spectroscopic data are in accordance with those described in the literature.[10]

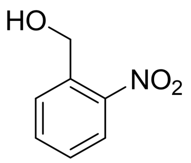

(2-Nitrophenyl)methanol (2b)

The general procedure was applied to 2-nitrobenzaldehyde (30.2 mg, 0.2 mmol) under an atmosphere of N_2 at 25 °C for 1 h. The crude product was purified by column chromatography on silica gel (Petroleum ether/EtOAc=10:1) to afford the title compound as oily liquid (27.2 mg, 89% yield); ^{1}H NMR (400 MHz, $CDCl_3$): δ = 8.09 (d, J = 8.2 Hz, 1H), 7.73 (d, J = 7.7 Hz, 1H), 7.67 (t, J = 7.5 Hz, 1H), 7.47 (t, J = 7.7 Hz, 1H), 4.96 (s, 2H), 2.80 (s, 1H); ^{13}C NMR (100 MHz, $CDCl_3$): δ= 147.5, 136.8, 134.2, 129.9, 128.5, 124.9, 62.4. The spectroscopic data are in accordance with those described in the literature.[11]

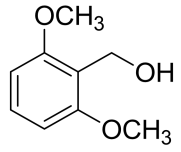

(2,6-Dimethoxyphenyl)methanol (2c)

The general procedure was applied to 2,6-dimethoxybenzaldehyde (33.2 mg, 0.2 mmol) under an atmosphere of N_2 at 25 °C for 1 h. The crude product was purified by column chromatography on silica gel (Petroleum ether/EtOAc=2:1) to afford the title compound as white solid (25.8 mg, 77% yield);^{1}H NMR (400 MHz, $CDCl_3$): δ= 7.20-7.12 (m, 1H), 6.49 (d, J = 8.3 Hz, 2H), 4.72 (d, J = 6.5 Hz, 2H), 3.78 (s, 6H), 2.40 (t, J = 6.7 Hz, 1H); ^{13}C NMR (100 MHz, $CDCl_3$): δ= 158.4, 129.1, 116.9, 103.7, 55.7, 54.7. The spectroscopic data are in accordance with those described in the literature.[10]

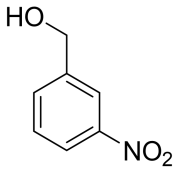

(3-Nitrophenyl)methanol (2d)

The general procedure was applied to 3-nitrobenzaldehyde (30.2 mg, 0.2 mmol) under an atmosphere of N_2 at 25 °C for 1 h. The crude product was purified by column chromatography on silica gel (Petroleum ether/EtOAc=10:1) to afford the title compound as oily liquid (28.4 mg, 93% yield);^{1}H NMR (400 MHz, $CDCl_3$): δ= 8.19 (d, J = 8.6 Hz, 2H), 7.52 (d, J = 8.3 Hz, 2H), 4.82 (s, 2H), 2.29 (s, 1H); ^{13}C NMR (100 MHz, $CDCl_3$): δ= 148.2, 147.1, 126.9, 123.7, 63.9. The spectroscopic data are in accordance with those described in the literature.[11]

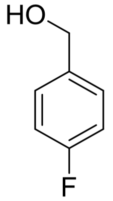

(4-Fluorophenyl)methanol (2e)

The general procedure was applied to 4-fluorobenzaldehyde (25.2 mg, 0.2 mmol) under an atmosphere of N_2 at 25 °C for 1 h. The crude product was purified by column chromatography on silica gel (Petroleum ether/EtOAc=2:1) to afford the title compound as yellow oily liquid (20.7 mg, 81% yield); ^{1}H NMR (400 MHz, $CDCl_3$): δ= 7.37-7.27 (m, 2H), 7.07-7.00 (m, 2H), 4.63 (s, 2H), 1.99 (s, 1H); ^{13}C NMR (100 MHz, $CDCl_3$): δ= 162.3 (d, J = 245.4 Hz), 136.5 (d, J = 3.2 Hz), 128.7 (d, J = 8.1 Hz), 115.4 (d, J = 21.3 Hz), 64.6. ^{19}F NMR (376 MHz, $CDCl_3$): δ= -114.8. The spectroscopic data are in accordance with those described in the literature.[11]

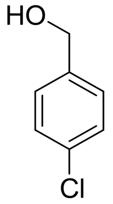

(4-Chlorophenyl)methanol (2f)

The general procedure was applied to 4-chlorobenzaldehyde (28.1 mg, 0.2 mmol) under an atmosphere of N_2 at 45 °C for 12 h. The crude product was purified by column chromatography on silica gel (Petroleum ether/EtOAc=2:1) to afford the title compound as white solid (24.7 mg, 87% yield); ^{1}H NMR (400 MHz, $CDCl_3$): δ =

7.26-7.17 (m, 4H), 4.56 (s, 2H), 2.00 (s, 1H); ^{13}C NMR (100 MHz, $CDCl_3$): δ = 139.2, 133.3, 128.6, 128.2, 64.4. The spectroscopic data are in accordance with those described in the literature.[11]

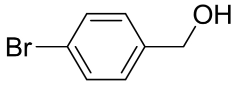

(4-bromophenyl)methanol (2g)

The general procedure was applied to (4-bromophenyl)methanol (37.0 mg, 0.2 mmol) under an atmosphere of N_2 at 25 °C for 1 h. The crude product was purified by column chromatography on silica gel (Petroleum ether/EtOAc=5:1) to afford the title compound as white solid (29.0 mg, 78% yield); ^1H NMR (400 MHz, $CDCl_3$): δ = 7.50-7.43 (d, J =8.4, 2H), 7.22 (d, J = 8.4 Hz, 2H), 4.63 (s, 2H), 2.00 (s, 1H). ^{13}C NMR (100 MHz, $CDCl_3$): δ =139.8, 131.7, 128.7, 121.6, 64.7. The spectroscopic data are in accordance with those described in the literature.[12]

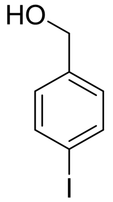

(4-Iodophenyl)methanol (2h)

The general procedure was applied to 4-iodobenzaldehyde (46.4 mg, 0.2 mmol) under an atmosphere of N_2 at 25 °C for 1 h. The crude product was purified by column chromatography on silica gel (Petroleum ether/EtOAc=5:1) to afford the title compound as white solid (43.0 mg, 92% yield); ^1H NMR (400 MHz, $CDCl_3$): δ =7.66 (d, J = 7.9 Hz, 2H), 7.07 (d, J = 8.3 Hz, 2H), 4.59 (s, 2H), 2.19 (s, 1H); ^{13}C NMR (100 MHz, $CDCl_3$): δ=140.3, 137.5, 128.8, 92.9, 64.5. The spectroscopic data are in accordance with those described in the literature.[11]

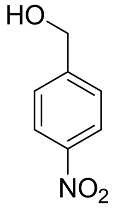

(4-Nitrophenyl)methanol (2i)

The general procedure was applied to 4-nitrobenzaldehyde (30.2 mg, 0.2 mmol) under an atmosphere of N_2 at 25 °C for 1 h. The crude product was purified by column chromatography on silica gel (Petroleum ether/EtOAc=10:1) to afford the title compound as oily liquid (24.7 mg, 81% yield); ^{1}H NMR (400 MHz, $CDCl_3$): δ= 8.19 (d, J = 8.6 Hz, 2H), 7.52 (d, J = 8.3 Hz, 2H), 4.82 (s, 2H), 2.29 (s, 1H); ^{13}C NMR (100 MHz, $CDCl_3$): δ= 148.2, 147.1, 126.9, 123.7, 63.9. The spectroscopic data are in accordance with those described in the literature.[10]

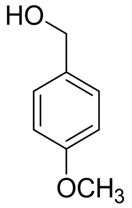

(4-Methoxyphenyl)methanol (2j)

The general procedure was applied to 4-methoxybenzaldehyde (27.2 mg, 0.2 mmol) under an atmosphere of N_2 at 45 °C for 12 h. The crude product was purified by column chromatography on silica gel (Petroleum ether/EtOAc=2:1) to afford the title compound as yellow oily liquid (20.9 mg, 76% yield); ^{1}H NMR (400 MHz, $CDCl_3$): δ= 7.20 (d, J = 8.4 Hz, 2H), 6.81 (d, J = 8.5 Hz, 2H), 4.52 (s, 2H), 3.73 (s, 3H), 1.86 (s, 1H); ^{13}C NMR (100 MHz, $CDCl_3$): δ=159.1, 133.1, 128.6, 113.9, 64.9, 55.2. The spectroscopic data are in accordance with those described in the literature.[13]

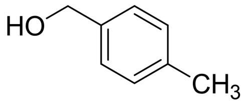

***p*-Tolylmethanol (2k)**

The general procedure was applied to 4-methylbenzaldehyde (24.0 mg, 0.2 mmol) under an atmosphere of N_2 at 45 °C for 12 h. The crude product was purified by column chromatography on silica gel (Petroleum ether/EtOAc=5:1) to afford the title compound as white solid (20.2 mg, 83% yield); ^{1}H NMR (400 MHz, $CDCl_3$): δ = 7.16 (d, J = 8.0 Hz, 2H), 7.08 (d, J = 7.8 Hz, 2H), 4.54 (s, 2H), 2.27 (s, 3H), 1.84 (s, 1H);

^{13}C NMR (100 MHz, $CDCl_3$): δ = 137.8, 137.3, 129.2, 127.1, 65.1, 21.1. The spectroscopic data are in accordance with those described in the literature.[13]

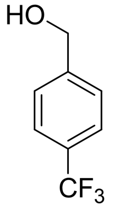

(4-(Trifluoromethyl)phenyl)methanol (2l)

The general procedure was applied to 4-methylbenzaldehyde (34.8 mg, 0.2 mmol) under an atmosphere of N_2 at 25 °C for 1 h. The crude product was purified by column chromatography on silica gel (Petroleum ether/EtOAc=2:1) to afford the title compound as yellow oily liquid (30.6 mg, 87% yield); ^{1}H NMR (400 MHz, $CDCl_3$): δ= 7.61 (d, J = 8.0 Hz, 2H), 7.46 (d, J = 8.0 Hz, 2H), 4.75 (s, 2H), 2.19 (s, 1H); ^{13}C NMR (100 MHz, $CDCl_3$): δ= 144.7, 129.7 (q, J = 32.2 Hz), 126.8, 125.4 (q, J = 3.8 Hz). 122.7, 64.4; ^{19}F NMR (376 MHz, $CDCl_3$): δ= -62.4. The spectroscopic data are in accordance with those described in the literature.[13]

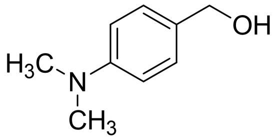

(4-(Dimethylamino)phenyl)methanol (2m)

The general procedure was applied to 4-(dimethylamino)benzaldehyde (29.8 mg, 0.2 mmol) under an atmosphere of N_2 at 25 °C for 1 h. The crude product was purified by column chromatography on silica gel (Petroleum ether/EtOAc=2:1) to afford the title compound as oily liquid (22.9 mg, 76% yield); ^{1}H NMR (400 MHz, $CDCl_3$): δ = 7.17 (d, J = 8.7 Hz, 2H), 6.65 (d, J = 8.7 Hz, 2H), 4.48 (s, 2H), 2.87 (s, 6H), 1.62 (s, 1H). ^{13}C NMR (100 MHz, $CDCl_3$): δ = 150.5, 128.9, 128.8, 128.8, 112.8, 112.7, 65.5, 40.8. The spectroscopic data are in accordance with those described in the literature.[11]

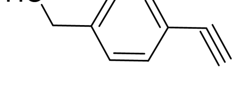

(4-Ethynylphenyl)methanol (2n)

The general procedure was applied to 4-ethynylbenzaldehyde (26.0 mg, 0.2 mmol) , under an atmosphere of N_2 at 25 °C for 1 h. The crude product was purified by column chromatography on silica gel (Petroleum ether/EtOAc=2:1) to afford the title compound as yellow oily liquid (17.2 mg, 65% yield); ^{1}H NMR (400 MHz, $CDCl_3$): δ= 7.49 (d, J = 8.2 Hz, 2H), 7.32 (d, J = 8.5 Hz, 2H), 4.70 (s, 2H), 3.08 (s, 1H).1.80(s, 1H); ^{13}C NMR (100 MHz, $CDCl_3$): δ= 141.5, 132.3, 126.7, 121.3, 83.4, 77.2, 64.8. The spectroscopic data are in accordance with those described in the literature.[14]

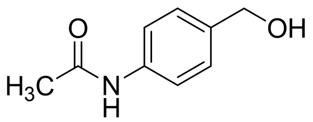

N-(4-(hydroxymethyl)phenyl)acetamide (2o)

The general procedure was applied to N-(4-formylphenyl)acetamide (32.6 mg, 0.2 mmol), HBpin (3 equiv.) under an atmosphere of N_2 at 45 °C for 24 h.The crude product was purified by column chromatography on silica gel (Petroleum ether/EtOAc=2:1) to afford the title compound as oily liquid (30.6 mg, 93% yield); ^{1}H NMR (400 MHz, DMSO-d_6): δ = 9.90 (s, 1H), 7.52 (d, J = 8.4 Hz, 2H), 7.22 (d, J = 8.4 Hz, 2H), 5.10 (s, 1H), 4.42 (s, 2H), 2.03 (s, 3H); ^{13}C NMR (100 MHz, DMSO-D_6): δ = 168.2, 138.0, 137.2, 127.0, 118.8, 62.7, 24.1.

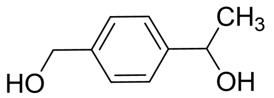

1-(4-(Hydroxymethyl)phenyl)ethanol (2p)

The general procedure was applied to 4-acetylbenzaldehyde (29.6 mg, 0.2 mmol) HBpin (3 equiv.) under an atmosphere of N_2 at 45 °C for 24 h. The crude product was purified by column chromatography on silica gel (Petroleum ether/EtOAc=5:1) to afford the title compound as oily liquid (26.6 mg, 87% yield); ^{1}H NMR (400 MHz, DMSO-d_6): δ = 7.78 (q, J = 8.1 Hz, 4H), 5.73-5.48 (m, 2H), 5.21 (d, J = 6.4 Hz, 1H), 4.98 (s, 2H), 1.82 (d, J = 6.5 Hz, 3H); ^{13}C NMR (100 MHz, DMSO-D_6): δ = 145.9, 140.8, 126.3, 125.1, 68.0, 62.9, 26.1.

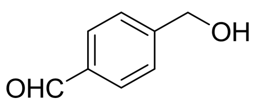

4-(Hydroxymethyl)benzaldehyde (2q)

The general procedure was applied to terephthalaldehyde (26.8 mg, 0.2 mmol) under an atmosphere of N_2 at 25 °C for 1 h. The crude product was purified by column chromatography on silica gel (Petroleum ether/EtOAc=5:1) to afford the title compound as oily liquid (23.4 mg, 86% yield);^{1}H NMR (400 MHz, $CDCl_3$): δ= 9.97 (s, 1H), 7.85 (d, J = 8.1 Hz, 2H), 7.51 (d, J = 8.2 Hz, 2H), 4.77 (s, 2H), 2.72 (s, 1H) ^{13}C NMR (100 MHz, $CDCl_3$): δ= 192.3, 148.2, 135.7, 130.2, 127.1, 75.2, 64.5. The spectroscopic data are in accordance with those described in the literature.[15]

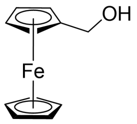

Ferrocenemethanol (2r)

The general procedure was applied to quinoline (42.8 mg, 0.2 mmol) under an atmosphere of N_2 at 25 °C for 1 h. The crude product was purified by column chromatography on silica gel (Petroleum ether/EtOAc=2:1) to afford the title compound as yellow solid (35.8 mg, 83% yield); ^{1}H NMR (400 MHz, DMSO-d_6): δ =4.72 (t, J = 5.8 Hz, 1H), 4.17 (d, J = 5.8 Hz, 2H), 4.15-4.13 (m, 2H), 4.09 (s, 4H), 4.05 (t, J = 1.8 Hz, 2H); ^{13}C NMR (100MHz, DMSO-D_6): δ = 88.4, 68.2, 68.2, 67.5, 59.2. The spectroscopic data are in accordance with those described in the literature.[10]

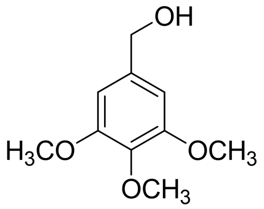

(3,4,5-Trimethoxyphenyl)methanol (2s)

The general procedure was applied to 3,4,5-trimethoxybenzaldehyde (39.6 mg, 0.2 mmol) under an atmosphere of N_2 at 25 °C for 1 h. The crude product was purified by column chromatography on silica gel (Petroleum ether/EtOAc=2:1) to afford the title

compound as oily liquid (35.6 mg, 89% yield); ^{1}H NMR (400 MHz, $CDCl_3$): δ= 6.54 (s, 2H), 4.58 (d, J = 5.5 Hz, 2H), 3.79 (d, J = 13.2 Hz, 9H); ^{13}C NMR (100 MHz, $CDCl_3$): δ=153.4, 137.2, 136.6, 103.7, 65.6, 60.9, 56.1. The spectroscopic data are in accordance with those described in the literature.[10]

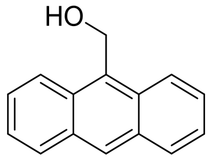

Anthracen-9-ylmethanol (2t)

The general procedure was applied to anthracene-9-carbaldehyde (41.3 mg, 0.2 mmol) under an atmosphere of N_2 at 25 °C for 1 h. The crude product was purified by column chromatography on silica gel (Petroleum ether/EtOAc=10:1) to afford the title compound as yellow solid (36.3 mg, 87% yield); ^{1}H NMR (400 MHz, DMSO-d_6): δ = 8.48 (s, 1H), 8.39 (d, J = 8.6 Hz, 2H), 8.00 (d, J = 8.0 Hz, 2H), 7.53-7.40 (m, 4H), 5.38 (d, J = 4.7 Hz, 2H), 5.28 (t, J = 5.0 Hz, 1H); ^{13}C NMR (100MHz, DMSO-D_6): δ = 132.9, 131.1, 129.9, 128.8, 127.1, 125.9, 125.1, 124.9, 55.4. The spectroscopic data are in accordance with those described in the literature.[16]

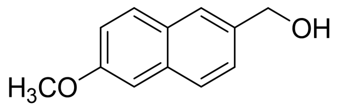

(6-Methoxynaphthalen-2-yl)methanol (2u)

The general procedure was applied to 6-methoxy-2-naphthaldehyde (37.2 mg, 0.2 mmol) under an atmosphere of N_2 at 25 °C for 1 h. The crude product was purified by column chromatography on silica gel (Petroleum ether/EtOAc=2:1) to afford the title compound as white solid (30.1 mg, 80% yield); ^{1}H NMR (400 MHz, DMSO-d_6): δ = 7.70 (t, J = 8.0 Hz, 2H), 7.66 (s, 1H), 7.35 (dd, J = 8.4, 1.7 Hz, 1H), 7.21 (d, J = 2.6 Hz, 1H), 7.06 (dd, J = 9.0, 2.6 Hz, 1H), 5.22 (t, J = 5.7 Hz, 1H), 4.55 (d, J = 5.7 Hz, 2H), 3.78 (s, 3H); ^{13}C NMR (100 MHz, DMSO-D_6): δ = 157.0, 137.8, 133.5, 129.2, 128.4, 126.6, 125.9, 124.5, 118.6, 105.9, 63.1, 55.2

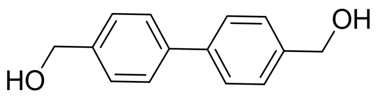

4,4'-Biphenyldiyldimethanol (2v)

The general procedure was applied to 4,4'-dicarbaldehyde (42.0 mg, 0.2 mmol), HBpin(3 equive) under an atmosphere of N_2 at 45 °C for 12 h. The crude product was purified by column chromatography on silica gel (Petroleum ether/EtOAc=1:1) to afford the title compound as white solid (32.9 mg, 77% yield); ^{1}H NMR (400 MHz, DMSO-d_6): δ = 7.53 (d, J = 8.3 Hz, 4H), 7.31 (d, J = 8.4 Hz, 4H), 5.17 (t, J = 5.7 Hz, 2H), 4.46 (d, J = 5.6 Hz, 4H); ^{13}C NMR (100 MHz, DMSO-D_6): δ = 141.7, 138.6, 127.1, 126.3, 62.7. The spectroscopic data are in accordance with those described in the literature.[10]

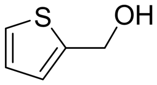

Thiophen-2-ylmethanol (2w)

The general procedure was applied to thiophene-2-carbaldehyde (22.4 mg, 0.2 mmol) under an atmosphere of N_2 at 25 °C for 1 h. The crude product was purified by column chromatography on silica gel (Petroleum ether/EtOAc=2:1) to afford the title compound as oily liquid (18.7 mg, 82% yield). ^{1}H NMR (400 MHz, $CDCl_3$): δ= 7.30-7.28 (m, 1H), 7.04-7.01 (m, 1H), 6.99 (ddd, J = 4.8, 3.4, 1.1 Hz, 1H), 4.84 (s, 2H), 1.81 (s, 1H); ^{13}C NMR (100 MHz, $CDCl_3$): δ=143.9, 126.9, 125.7, 125.5, 60.0. The spectroscopic data are in accordance with those described in the literature. [17]

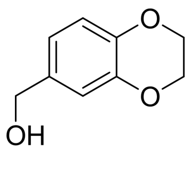

(2,3-Dihydrobenzo[b][1,4]dioxin-6-yl)methanol (2x)

The general procedure was applied to 2,3-dihydrobenzo[b][1,4]dioxine-6-carbalde-hyde (30.5 mg, 0.2 mmol) under an atmosphere of N_2 at 25 °C for 1 h. The crude product was purified by column chromatography on silica gel (Petroleum

ether/EtOAc=2:1) to afford the title compound as oily liquid (24.9 mg, 92% yield); ^{1}H NMR (400 MHz, $CDCl_3$): δ= 6.88-6.81 (m, 3H), 4.54 (d, J = 1.5 Hz, 2H), 4.24 (d, J = 1.6 Hz, 4H), 1.96 (s, 1H); ^{13}C NMR (100MHz, $CDCl_3$): δ=143.4, 142.9, 134.3, 120.3, 117.3, 116.2, 64.9, 64.3. The spectroscopic data are in accordance with those described in the literature.[10]

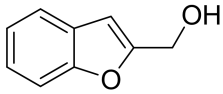

Benzofuran-2-ylmethanol (2y)

The general procedure was applied to benzofuran-2-carbaldehyde (29.2 mg, 0.2 mmol) under an atmosphere of N_2 at 25 °C for 1 h. The crude product was purified by column chromatography on silica gel (Petroleum ether/EtOAc=5:1) to afford the title compound as oily liquid (23.1 mg, 78% yield); ^{1}H NMR (400 MHz, $CDCl_3$): δ= 7.46 (d, J = 7.7 Hz, 1H), 7.38 (d, J = 8.1 Hz, 1H), 7.22-7.11 (m, 2H), 6.56 (s, 1H), 4.67 (s, 2H), 2.21 (s, 1H); ^{13}C NMR (100 MHz, $CDCl_3$): δ= 156.4, 155.0, 128.1, 124.4, 122.8, 121.1, 111.2, 104.1, 58.1. The spectroscopic data are in accordance with those described in the literature.[18]

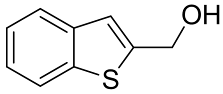

Benzo[b]thiophen-2-ylmethanol (2z)

The general procedure was applied to benzo[b]thiophene-2-carbaldehyde (32.5 mg, 0.2 mmol) under an atmosphere of N_2 at 25 °C for 1 h. The crude product was purified by column chromatography on silica gel (Petroleum ether/EtOAc=5:1) to afford the title compound as white solid (21.4 mg, 65% yield);^{1}H NMR (400 MHz, $CDCl_3$): δ= 7.84-7.79 (m, 1H), 7.74-7.70 (m, 1H), 7.38-7.29 (m, 2H), 7.20 (s, 1H), 4.91 (s, 2H), 2.16 (s, 1H); ^{13}C NMR (100 MHz, $CDCl_3$): δ= 144.7, 139.9, 139.5, 124.3, 124.3, 123.5, 122.4, 121.5, 60.8. The spectroscopic data are in accordance with those described in the literature. [19]

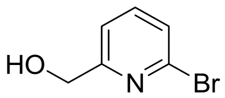

(6-Bromopyridin-2-yl)methanol (2aa)

The general procedure was applied to 6-bromopicolinaldehyde (37.2 mg, 0.2 mmol) under an atmosphere of N_2 at 25 °C for 1 h. The crude product was purified by column chromatography on silica gel (Petroleum ether/EtOAc=2:1) to afford the title compound as oily liquid (28.9 mg, 77% yield);^{1}H NMR (400 MHz, $CDCl_3$): δ= 7.54 (t, J = 7.7 Hz, 1H), 7.37 (dd, J = 7.9, 0.8 Hz, 1H), 7.29 (dd, J = 7.6, 0.8 Hz, 1H), 4.73 (s, 2H), 3.32(s, 1H). ^{13}C NMR (100MHz, $CDCl_3$): δ=161.3, 141.3, 139.1, 126.6, 119.3, 64.1. The spectroscopic data are in accordance with those described in the literature.[10]

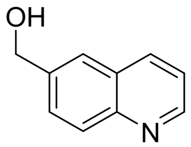

Quinolin-6-yl-methanol(2ab)

The general procedure was applied to quinoline-6-carbaldehyde (31. 4mg, 0.2 mmol) HBpin(3 equive) under an atmosphere of N_2 at 45 °C for 12 h. The crude product was purified by column chromatography on silica gel (Petroleum ether/EtOAc=2:1) to afford the title compound as yellow oily liquid (26.1 mg, 82% yield);^{1}H NMR (400 MHz, $CDCl_3$): δ= 8.74 (dd, J = 4.3, 1.7 Hz, 1H), 8.03 (d, J = 8.1 Hz, 1H), 7.96 (d, J = 8.7 Hz, 1H), 7.73 (s, 1H), 7.61 (dd, J = 8.7, 1.7 Hz, 1H), 7.31 (dd, J = 8.3, 4.3 Hz, 1H), 4.86 (s, 2H), 4.51 (s, 1H); ^{13}C NMR (100 MHz, $CDCl_3$): δ= 149.8, 147.3, 139.9, 136.2, 128.9, 128.8, 128.1, 124.7, 121.2, 64.3; HRMS (ESI): calcd for $C_{10}H_9NO$ $[M]^+$ 159.0684 found 159.0686.

5. General Procedure for Zn-Catalyzed Regioselective and Chemoselective Reduction of Ketones

A mixture of corresponding ketone derivatives (0.2 mmol) and $Zn(OAc)_2$ (0.002mmol) were added to Schlenk tube. Then HBpin (1.5 equiv) and THF (2 mL) were added by

syringe under atmosphere of nitrogen. The reaction mixture was stirred at 60 °C for 24 h. After quenching with saturated NH_4Cl/H_2O (10 mL). The crude product was extracted with EtOAc (3 × 10 mL). The combined organic phases were dried over anhydrous Na_2SO_4 and concentrated under vacuum, the crude product was purified by column chromatography to afford the desired hydrogenation compound.

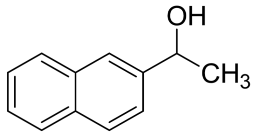

1-(Naphthalen-2-yl)ethanol (4a)

The general procedure was applied to 1-(naphthalen-2-yl)ethanone (34.1 mg, 0.2 mmol) under an atmosphere of N_2 at 60 °C for 24 h. The crude product was purified by column chromatography on silica gel (Petroleum ether/EtOAc=5:1) to afford the title compound as white solid (31.7 mg, 92% yield). ^{1}H NMR (400 MHz, $CDCl_3$): δ = 7.90-7.73 (m, 4H), 7.56-7.43 (m, 3H), 5.08 (dd, J = 6.5, 1.6 Hz, 1H), 1.98 (s, 1H), 1.59 (dd, J = 6.4, 1.6 Hz, 3H); ^{13}C NMR (100 MHz, $CDCl_3$): δ =143.1, 133.3, 132.9, 128.3, 127.9, 127.7, 126.2, 125.8, 123.8, 70.5, 25.1. The spectroscopic data are in accordance with those described in the literature.[11]

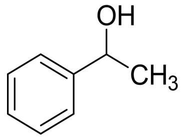

1-Phenylethanol (4b)

The general procedure was applied to acetophenone (24.0 mg, 0.2 mmol) under an atmosphere of N_2 at 60 °C for 24 h. The crude product was purified by column chromatography on silica gel (Petroleum ether/EtOAc=5:1) to afford the title compound as yellow oily liquid (22.2 mg, 91% yield). ^{1}H NMR (400 MHz, $CDCl_3$): δ = 7.34-7.25 (m, 4H), 7.23-7.18 (m, 1H), 4.83 (q, J = 6.5 Hz, 1H), 1.77 (s, 1H), 1.43 (d, J = 6.5 Hz, 3H). ^{13}C NMR (100 MHz, $CDCl_3$): δ =145.8, 128.5, 127.5, 125.4, 70.4, 25.2. The spectroscopic data are in accordance with those described in the literature.[11]

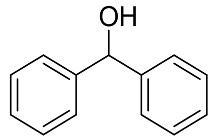

Diphenylmethanol (4c)

The general procedure was applied to benzophenone (36.5mg, 0.2 mmol) under an atmosphere of N_2 at 60 °C for 24 h. The crude product was purified by column chromatography on silica gel (Petroleum ether/EtOAc=5:1) to afford the title compound as white solid (30.2 mg, 82% yield). ^{1}H NMR (400 MHz, $CDCl_3$): δ = 7.29-7.21 (m, 8H), 7.20-7.13 (m, 2H), 5.71 (s, 1H), 2.31 (s, 1H). ^{13}C NMR (100 MHz, $CDCl_3$): δ = 142.7, 127.5, 126.5, 125.5, 75.2. The spectroscopic data are in accordance with those described in the literature.[11]

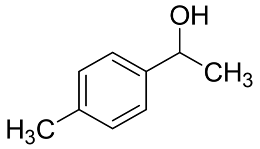

1-(p-tolyl)ethanol (4d)

The general procedure was applied to 1-(*p*-tolyl)ethanone (26.8 mg, 0.2 mmol) under an atmosphere of N_2 at 60 °C for 24 h. The crude product was purified by column chromatography on silica gel (Petroleum ether/EtOAc=5:1) to afford the title compound as oily liquid (23.4 mg, 86% yield). ^{1}H NMR (400 MHz, $CDCl_3$): δ =7.19 (d, J = 6.3 Hz, 2H), 7.09 (d, J = 7.5 Hz, 2H), 4.79 (q, J = 6.5 Hz, 1H), 2.27 (s, 3H), 1.74 (s, 1H), 1.41 (d, J = 6.5 Hz, 3H). ^{13}C NMR (100 MHz, $CDCl_3$): δ =142.8, 137.2, 129.2, 125.3, 70.2, 25.1, 21.1. The spectroscopic data are in accordance with those described in the literature.[11]

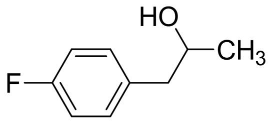

1-(4-Fluorophenyl)propan-2-ol (4e)

The general procedure was applied to 1-(4-fluorophenyl)propan-1-one (30.4 mg, 0.2 mmol) under an atmosphere of N_2 at 60 °C for 24 h. The crude product was purified

by column chromatography on silica gel (Petroleum ether/EtOAc=5:1) to afford the title compound as yellow oily liquid (26.8 mg, 87% yield). ^{1}H NMR (400 MHz, $CDCl_3$): δ =7.25-7.20 (m, 2H), 6.98-6.92 (m, 2H), 4.50 (t, J = 6.6 Hz, 1H), 1.86 (s, 1H), 1.78-1.58 (m, 2H), 0.82 (t, J = 7.4 Hz, 3H). ^{13}C NMR (100 MHz, $CDCl_3$): δ =162.1 (d, J = 245.0 Hz), 140.2 (d, J = 3.1 Hz), 127.6 (d, J = 8.1 Hz), 115.2 (d, J = 21.3 Hz), 75.3, 31.9, 10.0. ^{19}F NMR (376 MHz, $CDCl_3$): δ = -115.2. The spectroscopic data are in accordance with those described in the literature.[17]

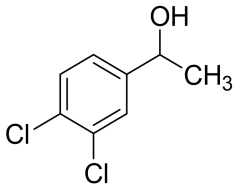

1-(3,4-Dichlorophenyl)ethanol (4f)

The general procedure was applied to 1-(3,4-dichlorophenyl)ethanone (37.8 mg, 0.2 mmol) under an atmosphere of N_2 at 60 °C for 24 h. The crude product was purified by column chromatography on silica gel (Petroleum ether/EtOAc=5:1) to afford the title compound as oily liquid (31.5 mg, 85% yield). ^{1}H NMR (400 MHz, $CDCl_3$): δ = 7.38 (d, J = 2.0 Hz, 1H), 7.32 (d, J = 8.3 Hz, 1H), 7.13-7.08 (m, 1H), 4.77 (q, J = 6.5 Hz, 1H), 2.03 (s, 1H), 1.38 (d, J = 6.5 Hz, 3H). ^{13}C NMR (100 MHz, $CDCl_3$): δ = 145.9, 132.5, 131.1, 130.4, 127.4, 124.8, 69.2, 25.3. The spectroscopic data are in accordance with those described in the literature.[20]

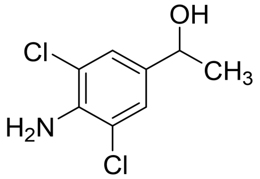

1-(4-Amino-3,5-dichlorophenyl)ethanol (4g)

The general procedure was applied to 1-(4-amino-3,5-dichlorophenyl)ethanone (40.8mg, 0.2 mmol) under an atmosphere of N_2 at 60 °C for 24 h. The crude product was purified by column chromatography on silica gel (Petroleum ether/EtOAc=5:1) to afford the title compound as white solid (30.7 mg, 75% yield). ^{1}H NMR (400 MHz,

$CDCl_3$): δ =7.12 (s, 2H), 4.67 (q, J = 6.4 Hz, 1H), 4.33 (s, 2H), 1.89 (s, 1H), 1.36 (d, J = 6.4 Hz, 3H). ^{13}C NMR (100MHz, $CDCl_3$): δ = 139.1, 136.2, 125.1, 119.5, 69.2, 24.9; HRMS (ESI): calcd for $C_8H_9C_{l2}NO$ $[M]^+$ 205.0061 found 205.0060.

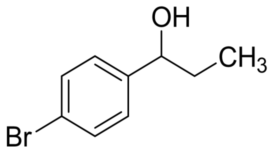

1-(4-bromophenyl)propan-1-ol (4h)

The general procedure was applied to 1-(4-bromophenyl)propan-1-one (42.6mg, 0.2 mmol) under an atmosphere of N_2 at 60 °C for 24 h. The crude product was purified by column chromatography on silica gel (Petroleum ether/EtOAc=5:1) to afford the title compound as white oily(33.4 mg, 78% yield). ^1H NMR (400 MHz, $CDCl_3$): δ = 7.46 -7.34 (m, 2H), 7.17 (d, J = 14.4 Hz, 2H), 4.50 (s, 1H), 1.84 (s, 1H), 1.79-1.59 (m, 2H), 1.18 (s, 3H). ^{13}C NMR (100 MHz, $CDCl_3$): δ =142.49, 130.44, 126.69, 120.17, 74.29, 30.89, 8.96. The spectroscopic data are in accordance with those described in the literature.[21]

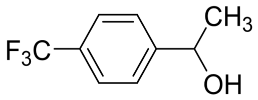

1-(4-(Trifluoromethyl)phenyl)ethanol (4i)

The general procedure was applied to 1-(4-(trifluoromethyl)phenyl)ethanone (37.6 mg, 0.2 mmol) under an atmosphere of N_2 at 60 °C for 24 h. The crude product was purified by column chromatography on silica gel (Petroleum ether/EtOAc=5:1) to afford the title compound as yellow oily liquid (32.7 mg, 86% yield). ^1H NMR (400 MHz, $CDCl_3$): δ =7.53 (d, J = 8.1 Hz, 2H), 7.41 (d, J = 8.0 Hz, 2H), 4.88 (q, J = 6.5 Hz, 1H), 1.99 (s, 1H), 1.44-1.40 (m, 3H). ^{13}C NMR (100 MHz, $CDCl_3$): δ =149.7, 129.6 (q, J = 32.3 Hz), 125.6, 125.4 (q, J = 3.7 Hz), 122.8, 69.8, 25.4. ^{19}F NMR (376 MHz, $CDCl_3$): δ = -62.3. The spectroscopic data are in accordance with those described in the literature.[21]

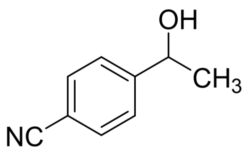

4-(1-Hydroxyethyl)benzonitrile (4j)

The general procedure was applied to 4-acetylbenzonitrile (29.0 mg, 0.2 mmol) under an atmosphere of N_2 at 60 °C for 24 h. The crude product was purified by column chromatography on silica gel (Petroleum ether/EtOAc=5:1) to afford the title compound as oily liquid (23.8 mg, 81% yield). ^{1}H NMR (400 MHz, $CDCl_3$): δ = 7.57 (d, J = 8.3 Hz, 2H), 7.42 (d, J = 8.2 Hz, 2H), 4.89 (q, J = 6.5 Hz, 1H), 2.04 (s, 1H), 1.43 (d, J = 6.5 Hz, 3H). ^{13}C NMR (100 MHz, $CDCl_3$): δ = 151.1, 132.3, 126.0, 118.9, 111.0, 69.6, 25.4. The spectroscopic data are in accordance with those described in the literature.[22]

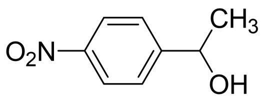

1-(4-Nitrophenyl)ethanol (4k)

The general procedure was applied to 1-(4-nitrophenyl)ethanone (33.0 mg, 0.2 mmol) under an atmosphere of N_2 at 60 °C for 24 h. The crude product was purified by column chromatography on silica gel (Petroleum ether/EtOAc=5:1) to afford the title compound as yellow oily liquid (29.7 mg, 89% yield). ^{1}H NMR (400 MHz, (400 MHz, $CDCl_3$): δ = 8.13-8.09 (m, 2H), 7.46 (d, J = 8.9 Hz, 2H), 4.94 (q, J = 6.5 Hz, 1H), 2.28 (s, 1H), 1.44 (d, J = 6.5 Hz, 3H). ^{13}C NMR (100 MHz, (400 MHz, $CDCl_3$): δ =152.2, 146.1, 125.1, 122.7, 68.5, 24.5. The spectroscopic data are in accordance with those described in the literature.[11]

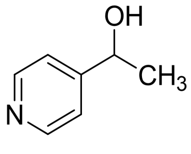

1-(Pyridin-4-yl)ethanol (4l)

The general procedure was applied to 1-(pyridin-4-yl)ethanone (24.2 mg, 0.2 mmol)

under an atmosphere of N_2 at 60 °C for 24 h. The crude product was purified by column chromatography on silica gel (Petroleum ether/EtOAc=1:1) to afford the title compound as oily liquid (20.1 mg, 82% yield). ^{1}H NMR (400 MHz, $CDCl_3$): δ =8.37 (d, J = 6.0 Hz, 2H), 7.23 (d, J = 6.1 Hz, 2H), 4.82 (q, J = 6.5 Hz, 1H), 1.41 (d, J = 6.6 Hz, 3H). ^{13}C NMR (100 MHz, $CDCl_3$): δ =154.5, 148.3, 119.6, 67.5, 24.1. The spectroscopic data are in accordance with those described in the literature.[23]

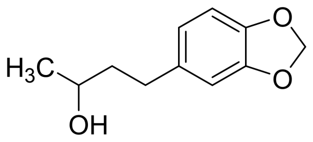

4-(benzo[d][1,3]dioxol-5-yl)butan-2-ol (4m)

The general procedure was applied to 4-(benzo[d][1,3]dioxol-5-yl)butan-2-one (22.4 mg, 0.2 mmol) under an atmosphere of N_2 at 60 °C for 24 h. The crude product was purified by column chromatography on silica gel (Petroleum ether/EtOAc=5:1) to afford the title compound as yellow oily liquid (30.6 mg, 79% yield). ^{1}H NMR (400 MHz, $CDCl_3$): δ = 6.67-6.61 (m, 2H), 6.57 (d, J = 9.5 Hz, 1H), 5.84 (s, 2H), 3.74 (q, 1H), 2.65-2.47 (m, 2H), 1.70 -1.60 (m, 2H), 1.45 (s, 1H), 1.15 (d, J = 6.2 Hz, 3H). ^{13}C NMR (100 MHz, $CDCl_3$): δ = 146.6, 144.6, 134.9, 120.0, 107.9, 107.2, 99.7, 66.4, 40.0, 30.9, 22.6; HRMS (ESI): calcd for $C_{11}H_{14}O_3$ $[M]^+$ 194.0943 found 194.0941.

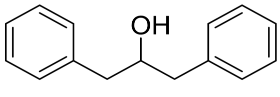

1,3-Diphenylpropan-2-ol (4n)

The general procedure was applied to 1,3-diphenylpropan-2-one (42.1 mg, 0.2 mmol) under an atmosphere of N_2 at 60 °C for 24 h. The crude product was purified by column chromatography on silica gel (Petroleum ether/EtOAc=5:1) to afford the title compound as oily liquid (38.1 mg, 90% yield). ^{1}H NMR (400 MHz, $CDCl_3$): δ = 7.26-7.19 (m, 4H), 7.15 (tt, J = 6.4, 1.4 Hz, 6H), 3.96 (dddd, J = 12.6, 7.8, 4.4, 2.4 Hz, 1H), 2.77 (dd, J = 13.6, 4.7 Hz, 2H), 2.66 (dd, J = 13.6, 8.2 Hz, 2H), 1.60 (s, 1H).^{13}C NMR (100 MHz, $CDCl_3$): δ = 137.4, 128.4, 127.5, 125.5, 72.6, 42.3; HRMS (ESI):

calcd for $C_{15}H_{16}O$ $[M]^+$ 212.1201 found 212.1204.

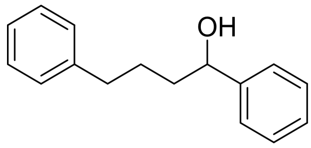

1,4-Diphenylbutan-1-ol (4o)

The general procedure was applied to 1,4-diphenylbutan-1-one (44.9 mg, 0.2 mmol) under an atmosphere of N_2 at 60 °C for 24 h. The crude product was purified by column chromatography on silica gel (Petroleum ether/EtOAc=5:1) to afford the title compound as oily liquid (40 mg, 89% yield). ^1H NMR (400 MHz, $CDCl_3$): δ =7.25 -7.14 (m, 7H), 7.11-7.03 (m, 3H), 4.55 (dd, J = 7.3, 5.3 Hz, 1H), 2.53 (t, J = 7.3 Hz, 2H), 1.90 (s, 1H), 1.77-1.70 (m, 1H), 1.69-1.62 (m, 2H), 1.55-1.47 (m, 1H). ^{13}C NMR (100 MHz, $CDCl_3$): δ =143.7, 141.20 127.4, 127.4, 127.3, 126.5, 124.9, 124.7, 73.4, 37.5, 34.7, 26.6; HRMS (ESI): calcd for $C_{16}H_{18}O$ $[M]^+$ 226.1358 found 226.1358.

6.General Procedure for Zn-Catalyzed Regioselective and Chemoselective Reduction of *α,β*-Unsaturated Aldehydes/Ketones

A mixture of corresponding *α, β*-unsaturated carbonyl compounds (0.2 mmol) and $Zn(OAc)_2$ (0.004mmol) were added to Schlenk tube. Then HBpin (1.5 equiv) and THF (2 mL) were added by syringe under atmosphere of nitrogen. The reaction mixture was stirred at 45 °C for 24 h. After quenching with saturated NH_4Cl/H_2O (10 mL). The crude product was extracted with EtOAc (3×10 mL). The combined organic phases were dried over anhydrous Na_2SO_4 and concentrated under vacuum, the crude product was purified by column chromatography to afford the desired hydrogenation compound.

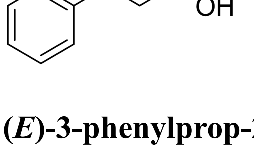

(*E*)-3-phenylprop-2-en-1-ol (6a)

The general procedure was applied to cinnamaldehyde (26.4 mg, 0.2 mmol) under an atmosphere of N_2 at 45 °C for 24 h. The crude product was purified by column

chromatography on silica gel (Petroleum ether/EtOAc=2:1) to afford the title compound as yellow oily liquid (24.1 mg, 90% yield). ^{1}H NMR (400 MHz, $CDCl_3$): δ= 7.44-7.21 (m, 5H), 6.60 (d, J = 15.5 Hz, 1H), 6.40-6.30 (m, 1H), 4.35-4.28 (m, 2H), 1.87 (s, 1H); ^{13}C NMR (100 MHz, $CDCl_3$): δ= 136.6, 131.0, 128.6, 128.4, 127.7, 126.4, 63.6. The spectroscopic data are in accordance with those described in the literature.[24]

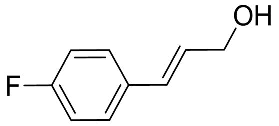

(*E*)-3-(4-fluorophenyl)prop-2-en-1-ol (6b)

The general procedure was applied to (*E*)-3-(4-fluorophenyl)acrylaldehyde (30.0 mg, 0.2 mmol) under an atmosphere of N_2 at 45 °C for 24 h. The crude product was purified by column chromatography on silica gel (Petroleum ether/EtOAc=2:1) to afford the title compound as yellow oily liquid (26.1 mg, 86% yield). ^{1}H NMR (400 MHz, $CDCl_3$): δ= 7.36-7.31 (m, 2H), 7.03-6.97 (m, 2H), 6.57 (d, J = 15.9 Hz, 1H), 6.32-6.23 (m, 1H), 4.31 (dd, J = 5.7, 1.5 Hz, 2H), 1.84 (s, 1H); ^{13}C NMR (100 MHz, $CDCl_3$): δ= 162.39(d, J = 246.7Hz) , 132.8 (d, J = 3.4 Hz), 129.9, 128.1 (d, J = 2.3 Hz), 127.9 (d, J = 8.1 Hz), 115.5 (d, J = 21.6 Hz), 63.6. ^{19}F NMR (376 MHz, $CDCl_3$): δ= -114.2. The spectroscopic data are in accordance with those described in the literature.[24]

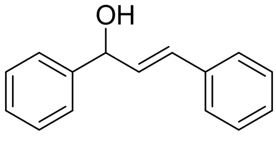

(*E*)-1,3-diphenylprop-2-en-1-ol (6c)

The general procedure was applied to (*E*)-chalcone (41.6 mg, 0.2 mmol) under an atmosphere of N_2 at 45 °C for 24 h. The crude product was purified by column chromatography on silica gel (Petroleum ether/EtOAc=5:1) to afford the title compound as yellow oily liquid (34.5 mg, 82% yield). ^{1}H NMR (400 MHz, $CDCl_3$):

δ=7.36-7.33 (m, 2H), 7.32-7.26 (m, 4H), 7.25-7.19 (m, 3H), 7.18-7.14 (m, 1H), 6.59 (d, J = 15.8 Hz, 1H), 6.29 (dd, J = 15.8, 6.5 Hz, 1H), 5.29 (d, J = 6.5 Hz, 1H), 2.14 (s, 1H). ^{13}C NMR (100 MHz, $CDCl_3$): δ= 142.7, 136.5, 131.4, 130.5, 128.6, 128.5, 127.8, 127.75, 126.6, 126.3, 75.1. The spectroscopic data are in accordance with those described in the literature.[25]

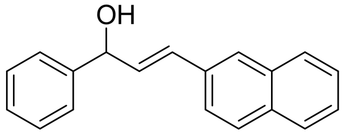

(*E*)-3-(naphthalen-2-yl)-1-phenylprop-2-en-1-ol (6d)

The general procedure was applied to (*E*)-3-(naphthalen-2-yl)-1-phenylprop-2-en-1-one (51.7 mg, 0.2 mmol) under an atmosphere of N_2 at 45 °C for 24 h. The crude product was purified by column chromatography on silica gel (Petroleum ether/EtOAc=5:1) to afford the title compound as yellow oily liquid (44.2 mg, 85% yield). ^{1}H NMR (400 MHz, $CDCl_3$): δ= 7.72-7.67 (m, 3H), 7.65 (d, J = 6.1 Hz, 2H), 7.49 (dd, J = 8.6, 1.6 Hz, 1H), 7.40-7.34 (m, 4H), 7.30 (dd, J = 8.5, 6.7 Hz, 2H), 6.75 (d, J = 15.8 Hz, 1H), 6.42 (dd, J = 15.6, 6.5 Hz,1H), 5.34 (d, J = 6.5 Hz, 1H), 2.10 (s, 1H). ^{13}C NMR (100 MHz, $CDCl_3$): δ=141.7, 132.9, 132.5, 132.0, 130.8, 129.6, 127.7, 127.2, 126.9, 126.8, 126.6, 125.7, 125.4, 125.3, 124.9, 74.2. The spectroscopic data are in accordance with those described in the literature.[26]

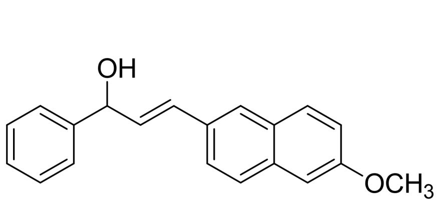

(*E*)-3-(6-methoxynaphthalen-2-yl)-1-phenylprop-2-en-1-ol (6e)

The general procedure was applied to (*E*)-3-(6-methoxynaphthalen-2-yl)-1-phenylprop-2-en-1-one (57.7 mg, 0.2 mmol) under an atmosphere of N_2 at 45 °C for 24 h. The crude product was purified by column chromatography on silica

gel(Petroleum ether/EtOAc=5:1) to afford the title compound as yellow oily liquid (50.1 mg, 86% yield). ^{1}H NMR (400 MHz, $CDCl_3$): δ= 7.72-7.65 (m, 3H), 7.56 (dd, J = 8.7, 1.5 Hz, 1H), 7.49 (d, J = 7.6 Hz, 2H), 7.41 (t, J = 7.3 Hz, 2H), 7.37-7.31 (m, 1H), 7.15 (ddd, J = 8.9, 2.4, 1.5 Hz, 1H), 7.11 (d, J = 2.4 Hz, 1H), 6.80 (d, J = 15.7 Hz, 1H), 6.47 (dd, J = 15.8, 6.7 Hz, 1H), 5.44-5.41 (m, 1H), 3.92 (s, 3H), 2.49 (s, 1H). ^{13}C NMR (100 MHz, $CDCl_3$): δ= 157.7, 142.9, 134.2, 131.8, 130.8, 130.7, 129.5, 128.9, 128.6, 127.7, 126.9, 126.5, 126.3, 124.1, 118.9, 105.8, 75.2, 55.2. The spectroscopic data are in accordance with those described in the literature.[27]

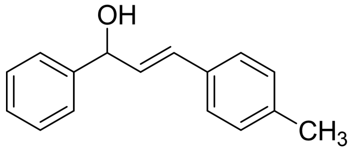

(*E*)-1-phenyl-3-(p-tolyl)prop-2-en-1-ol (6f)

The general procedure was applied to (*E*)-1-phenyl-3-(p-tolyl)prop-2-en-1-one (44.5 mg, 0.2 mmol) under an atmosphere of N_2 at 45 °C for 24h. The crude product was purified by column chromatography on silica gel (Petroleum ether/EtOAc=5:1) to afford the title compound as yellow oily liquid (38.5 mg, 86% yield). ^{1}H NMR (400 MHz, $CDCl_3$): δ= 7.37-7.33 (m, 2H), 7.29 (t, J = 7.5 Hz, 2H), 7.24-7.18 (m, 3H), 7.03 (d, J = 8.0 Hz, 2H), 6.57 (d, J = 15.8 Hz, 1H), 6.25 (dd, J = 15.8, 6.7 Hz, 1H), 5.28 (d, J = 6.4 Hz, 1H), 2.25 (s, 3H), 2.03 (s, 1H). ^{13}C NMR (100 MHz, $CDCl_3$): δ=141.8, 132.7, 129.5, 129.5, 128.3, 127.6, 126.7, 125.5, 125.3, 74.2, 20.2. The spectroscopic data are in accordance with those described in the literature.[26]

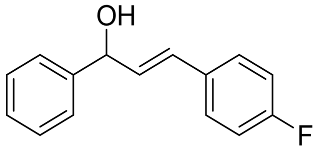

(*E*)-3-(4-fluorophenyl)-1-phenylprop-2-en-1-ol (6g)

The general procedure was applied to (*E*)-3-(4-fluorophenyl)-1-phenylprop-2-en-1-one (45.3 mg, 0.2 mmol) under an atmosphere of N_2 at 45 °C for 24 h. The crude product was purified by column chromatography on silica gel (Petroleum

ether/EtOAc=5:1) to afford the title compound as yellow oily liquid (37.0 mg, 81% yield). ^{1}H NMR (400 MHz, $CDCl_3$): *δ*= 7.37-7.20 (m, 7H), 6.92 (t, *J* = 8.6 Hz, 2H), 6.58 (d, *J* = 15.8 Hz, 1H), 6.23 (dd, *J* = 15.8, 6.5 Hz, 1H), 5.30 (d, *J* = 5.4 Hz, 1H), 2.01 (s, 1H). ^{13}C NMR (100 MHz, $CDCl_3$): δ=162.5 (d, *J* = 247.2 Hz), 142.8, 132.8 (d, *J* = 3.1 Hz), 131.3 (d, *J* = 2.3 Hz), 129.5, 128.8, 128.3 (d, J = 8.0 Hz), 128.0, 126.4, 115.60 (d, *J* = 21.6 Hz). 75.2.^{19}F NMR (376 MHz, $CDCl_3$): *δ*= -113.9. The spectroscopic data are in accordance with those described in the literature.[26]

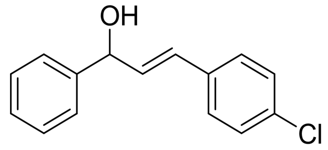

(*E*)-3-(4-chlorophenyl)-1-phenylprop-2-en-1-ol (6h)

The general procedure was applied to (*E*)-3-(4-chlorophenyl)-1-phenylprop-2-en-1-one (48.5 mg, 0.2 mmol) under an atmosphere of N_2 at 45 °C for 24 h. The crude product was purified by column chromatography on silica gel (Petroleum ether/EtOAc=5:1) to afford the title compound as yellow oily liquid (41.1 mg, 84% yield). ^{1}H NMR (400 MHz, $CDCl_3$): *δ*=7.36-7.26 (m, 4H), 7.22 (ddd, *J* = 14.6, 5.9, 1.9 Hz, 5H), 6.55 (d, *J* = 15.8 Hz, 1H), 6.30-6.23 (m, 1H), 5.28 (d, *J* = 6.2 Hz, 1H), 2.12 (s, 1H). ^{13}C NMR (100 MHz, $CDCl_3$): *δ*=141.5, 133.9, 132.4, 131.1, 128.1, 127.7, 126.9, 126.8, 125.3, 73.9. The spectroscopic data are in accordance with those described in the literature.[26]

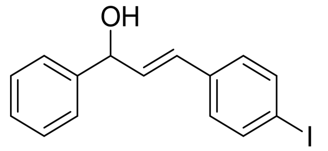

(*E*)-3-(4-iodophenyl)-1-phenylprop-2-en-1-ol(6i)

The general procedure was applied to (*E*)-3-(4-iodophenyl)-1-phenylprop-2-en-1-one (66.8 mg, 0.2 mmol) under an atmosphere of N_2 at 45 °C for 24h. The crude product was purified by column chromatography on silica gel (Petroleum ether/EtOAc=5:1) to afford the title compound as yellow oily liquid (55.4 mg, 81% yield). ^{1}H NMR (400

MHz, CDCl3): δ= 7.63 (d, J = 8.4 Hz, 2H), 7.44 -7.36 (m, 4H), 7.34- 7.29 (m, 1H), 7.10 (d, J = 8.4 Hz, 2H), 6.60 (d, J = 15.7 Hz, 1H), 6.38 (dd, J = 15.8, 6.3 Hz, 1H), 5.35 (d, J = 6.3 Hz, 1H), 2.32 (s, 1H). ^{13}C NMR (100 MHz, $CDCl_3$): δ= 142.4, 137.6, 135.9, 132.3, 129.2, 128.7, 128.3, 127.9, 126.3, 93.1, 74.9. The spectroscopic data are in accordance with those described in the literature.[26]

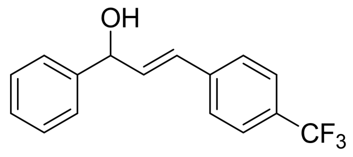

(*E*)-1-phenyl-3-(4-(trifluoromethyl)phenyl)prop-2-en-1-ol(6j)

The general procedure was applied to (*E*)-1-phenyl-3-(4-(trifluoromethyl)phenyl) prop-2-en-1-one (55.3 mg, 0.2 mmol) under an atmosphere of N_2 at 45 °C for24 h. The crude product was purified by column chromatography on silica gel(Petroleum ether/EtOAc=5:1) to afford the title compound as yellow oily liquid(47.8 mg, 86% yield). ^{1}H NMR (400 MHz, $CDCl_3$): δ= 7.56 (d, J = 8.2 Hz, 2H), 7.50-7.44 (m, 3H), 7.43-7.37 (m, 3H), 7.36-7.32 (m, 1H), 6.73 (d, J = 15.9 Hz, 1H), 6.48 (dd, J = 15.8, 6.1 Hz, 1H), 5.41 (d, J = 6.1 Hz, 1H), 2.24(s, 1H). ^{19}F NMR (376 MHz, $CDCl_3$): δ= -62.36. The spectroscopic data are in accordance with those described in the literature.[26]

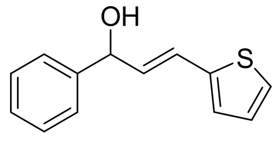

(*E*)-1-phenyl-3-(thiophen-2-yl)prop-2-en-1-ol (6k)

The general procedure was applied to (*E*)-1-phenyl-3-(thiophen-2-yl)prop-2-en-1-one (42.8 mg, 0.2 mmol) under an atmosphere of N_2 at 45 °C for 24h. The crude product was purified by column chromatography on silica gel (Petroleum ether/EtOAc=5:1) to afford the title compound as yellow oily liquid (40.1 mg, 93% yield). ^{1}H NMR (400 MHz, $CDCl_3$): δ= 7.33-7.25 (m, 4H), 7.24-7.18 (m, 1H), 7.06 (d, J = 4.9 Hz, 1H), 6.88-6.85 (m, 2H), 6.69 (d, J = 15.6 Hz, 1H), 6.12 (dd, J = 15.7, 6.4 Hz, 1H), 5.22 (d,

J = 6.4 Hz, 1H), 2.21 (s, 1H). ^{13}C NMR (100 MHz, $CDCl_3$): δ= 141.5, 140.6, 129.9, 127.6, 126.8, 126.4, 125.4, 125.2, 123.5, 122.7, 73.7. The spectroscopic data are in accordance with those described in the literature.[28]

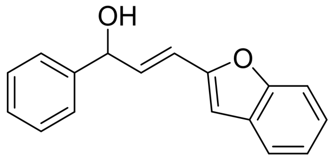

(*E*)-3-(benzofuran-2-yl)-1-phenylprop-2-en-1-ol (6l)

The general procedure was applied to (*E*)-3-(benzofuran-2-yl)-1-phenylprop-2-en-1-one (49.7 mg, 0.2 mmol) under an atmosphere of N_2 at 45 °C for 24 h. The crude product was purified by column chromatography on silica gel (Petroleum ether/EtOAc=5:1) to afford the title compound as yellow oily liquid (43.6 mg, 87% yield). ^{1}H NMR (400 MHz, $CDCl_3$): δ= 7.42-7.38 (m, 1H), 7.35-7.25 (m, 5H), 7.24-7.19 (m, 1H), 7.15 (ddd, J = 8.2, 7.3, 1.4 Hz, 2H), 7.12-7.06 (m, 1H), 6.54 (d, J = 1.7 Hz, 1H), 6.48 (s, 2H), 5.29 (s, 1H), 2.22 (s, 1H). ^{13}C NMR (100 MHz, $CDCl_3$): δ= 154.7, 154.1, 142.3, 133.4, 128.8, 128.7, 127.9, 126.5, 124.6, 122.8, 120.9, 118.4, 110.9, 105.1, 74.5; HRMS (ESI): calcd for $C_{17}H_{14}O_2$ $[M]^+$ 250.0994 found 250.0996.

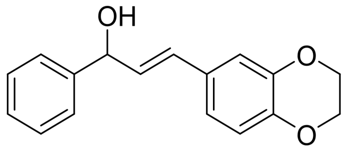

(*E*)-3-(2,3-dihydrobenzo[b][1,4]dioxin-6-yl)-1-phenylprop-2-en-1-ol (6m)

The general procedure was applied to (*E*)-3-(2,3-dihydrobenzo[b][1,4]dioxin-6-yl)-1-phenylprop-2-en-1-one (53.3 mg, 0.2 mmol) under an atmosphere of N_2 at 45 °C for 24 h. The crude product was purified by column chromatography on silica gel (Petroleum ether/EtOAc=5:1) to afford the title compound as yellow oily liquid (51 mg, 95% yield). ^{1}H NMR (400 MHz, $CDCl_3$): δ= 7.36-7.24 (m, 4H), 7.20 (q, J = 7.4 Hz, 1H), 6.85-6.80 (m, 1H), 6.80-6.76 (m, 1H), 6.71 (t, J = 7.7 Hz, 1H), 6.46 (dd, J = 15.7, 6.2 Hz, 1H), 6.14 (dt, J = 15.7, 7.1 Hz, 1H), 5.25 (t, J = 6.4 Hz, 1H), 4.14 (d, J = 6.8 Hz, 4H), 2.21 (s, 1H). ^{13}C NMR (100MHz, $CDCl_3$): δ= 143.3, 142.9, 130.2, 129.9,

128.5, 127.7, 126.3, 119.9, 117.3, 115.2, 75.1, 64.2; HRMS (ESI): calcd for $C_{17}H_{16}O_3$ $[M]^+$ 268.1099 found 268.1096.

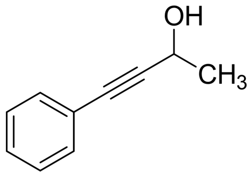

4-phenylbut-3-yn-2-ol (6n)

The general procedure was applied to 4-phenylbut-3-yn-2-one (28.8 mg, 0.2 mmol) under an atmosphere of N_2 at 45 °C for 24 h. The crude product was purified by column chromatography on silica gel (Petroleum ether/EtOAc=5:1) to afford the title compound as yellow oily liquid (21.5mg, 74% yield). ^1H NMR (400 MHz, $CDCl_3$): δ = 7.37-7.33 (m, 2H), 7.26-7.19 (m, 3H), 4.68 (q, J = 6.6 Hz, 1H), 2.21 (s, 1H), 1.47 (q, J = 6.6, 0.8 Hz, 3H). ^{13}C NMR (100 MHz, $CDCl_3$): δ = 131.8, 128.5, 128.4, 122.7, 91.1, 84.1, 58.9, 24.5. The spectroscopic data are in accordance with those described in the literature.[25]

7.General Procedure for Zn-Catalyzed Regioselective and Chemoselective Reduction of Imines

A mixture of corresponding Imines (0.2 mmol) and $Zn(OAc)_2$ (0.002mmol) were added to Schlenk tube. Then HBpin (1.5 equiv) and THF (2 mL) were added by syringe under atmosphere of nitrogen. The reaction mixture was stirred at 25 °C for 5 h. After quenching with saturated NH_4Cl/H_2O (10 mL). The crude product was extracted with EtOAc (3 × 10 mL). The combined organic phases were dried over anhydrous Na_2SO_4 and concentrated under vacuum, the crude product was purified by column chromatography to afford the desired hydrogenation compound.

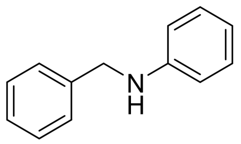

N-benzylaniline(8a)

The general procedure was applied to (E)-N,1-diphenylmethanimine (36.2 mg, 0.2

mmol) under an atmosphere of N_2 at 25 °C for 5 h. The crude product was purified by column chromatography on silica gel (Petroleum ether/EtOAc=10:1) to afford the title compound as yellow oily liquid (35.1mg, 96%yield); ^{1}H NMR (400 MHz, $CDCl_3$): δ = 7.31-7.22 (m, 4H), 7.15-7.05 (m, 2H), 6.63 (tt, J = 7.4, 1.1 Hz, 1H), 6.55 (dt, J = 7.7, 1.1 Hz, 2H), 4.23 (s, 2H), 3.94 (s, 1H). ^{13}C NMR (100 MHz, $CDCl_3$): δ = 147.1, 138.4, 128.3, 127.6, 126.5, 126.2, 116.5, 111.8, 47.3. The spectroscopic data are in accordance with those described in the literature.[29]

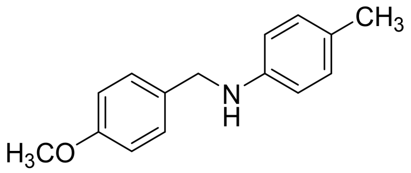

N-(4-methoxybenzyl)-4-methylaniline(8b)

The general procedure was applied to (E)-1-(4-methoxyphenyl)-N-(p-tolyl)methanimine (45.0 mg, 0.2 mmol) under an atmosphere of N_2 at 25 °C for 5 h. Thecrude product was purified by column chromatography on silica gel (Petroleumether/EtOAc=5:1) to afford the title compound as yellow oily liquid (42.6mg, 94% yield); ^{1}H NMR (400 MHz, $CDCl_3$): δ =7.22-7.17 (m, 2H), 6.96-6.86 (m, 2H), 6.81-6.76 (m, 2H), 6.52-6.43 (m, 2H), 4.13 (s, 2H), 3.70 (s, 3H), 2.15 (s,3H). ^{13}C NMR (100 MHz, $CDCl_3$): δ =157.7, 144.9, 130.6, 128.7, 127.8, 125.6,112.9, 111.9, 54.2, 47.1, 19.4. The spectroscopic data are in accordance with those described in the literature.[29]

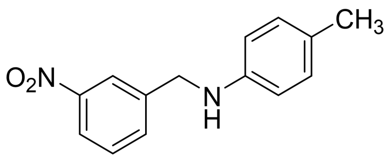

4-methyl-N-(3-nitrobenzyl)aniline(8c)

The general procedure was applied to (E)-1-(3-nitrophenyl)-N-(p-tolyl)methanimine (48.0 mg, 0.2 mmol) under an atmosphere of N_2 at 25 °C for 5 h. The crude product was purified by column chromatography on silica gel (Petroleum ether/EtOAc=5:1) to afford the title compound as yellow solid (42.1mg, 87%yield); ^{1}H NMR (400 MHz, $CDCl_3$): δ = 8.15 (s, 1H), 8.02 (d, J = 7.7 Hz, 1H), 7.62 (d, J = 6.4 Hz, 1H), 7.45 –

7.34 (m, 1H), 6.91 (s, 2H), 6.44 (d, J = 7.3 Hz, 2H), 4.35 (s, 2H), 4.05 (s, 1H), 2.15 (s, 3H).^{13}C NMR (100 MHz, $CDCl_3$): δ =147.5, 144.0, 141.2, 132.2, 128.8, 128.5, 126.4, 121.2, 121.0, 112.0, 46.7, 19.4. The spectroscopic data are in accordance with those described in the literature.[29]

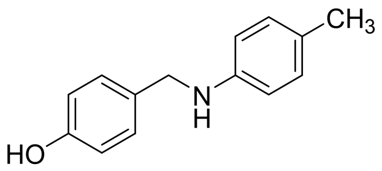

4-((p-tolylamino)methyl)phenol(8d)

The general procedure was applied to (E)-4-((p-tolylimino)methyl)phenol (42.2 mg, 0.2 mmol) under an atmosphere of N_2 at 25 °C for 5 h. The crude product was purified by column chromatography on silica gel (Petroleum ether/EtOAc=5:1) to afford the title compound as yellow oily liquid (37.9mg, 90%yield); ^{1}H NMR (400 MHz, $CDCl_3$): δ = 7.12 (d, J = 8.5 Hz, 2H), 6.91 (d, J = 8.3 Hz, 2H), 6.67 (d, J = 8.5 Hz, 2H), 6.49 (d, J = 8.4 Hz, 2H), 4.11 (s, 2H), 2.16 (s, 3H). ^{13}C NMR (100 MHz, $CDCl_3$): δ =153.8, 144.8, 130.4, 128.8, 128.0, 126.1, 114.4, 112.3, 47.3, 19.4. The spectroscopic data are in accordance with those described in the literature.[29]

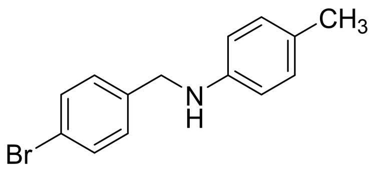

N-(4-bromobenzyl)-4-methylaniline(8e)

The general procedure was applied to (E)-1-(4-bromophenyl)-N-(p-tolyl)methanimine (54.8 mg, 0.2 mmol) under an atmosphere of N_2 at 25 °C for 5 h. The crude product was purified by column chromatography on silica gel (Petroleum ether/EtOAc=5:1) to afford the title compound as orange solid (49.6mg, 91%yield);^{1}H NMR (400 MHz, $CDCl_3$): δ = 7.37-7.31 (m, 2H), 7.17-7.09 (m, 2H), 6.93-6.82 (m, 2H), 6.48-6.40 (m, 2H), 4.16 (s, 2H), 3.83 (s, 1H), 2.14 (s, 3H). ^{13}C NMR (100 MHz, $CDCl_3$): δ = 144.5, 137.7, 130.6, 128.8, 127.9, 125.9, 119.8, 111.9, 46.9, 19.4. The spectroscopic data are

in accordance with those described in the literature.[30]

8. Gram-Scale Hydrogenation of 1a

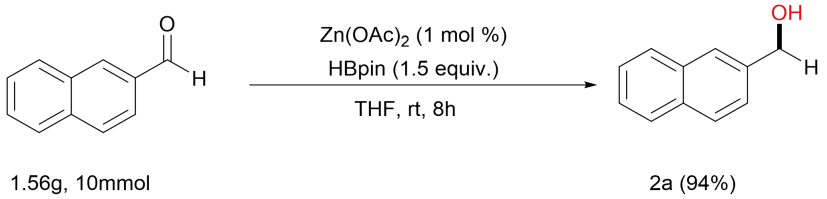

$Zn(OAc)_2$ (0.1 mmol, 1mol%) were added to an oven Schlenk tube under atmosphere of nitrogen, 2-naphthaldehyde (10 mmol), HBpin (1.5 equiv) and THF (100 mL) were added by syringe. The reaction mixture was stirred at 25 °C for 24h. extracted with EtOAc (3 × 40mL). The combined organic phases were dried over anhydrous Na_2SO_4 and concentrated under vacuum. The crude product was purified by column chromatography on silica gel using petroleum ether as eluent to provide the hydrogenated product **2a**

9. Hg(0) Poisoning Experiments

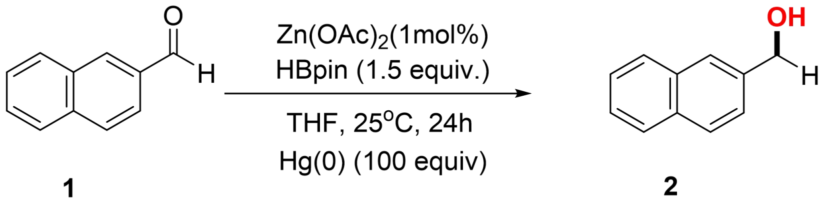

$Zn(OAc)_2$ (0.002mmol, 1mol %) were added to an oven dried high pressure tube under atmosphere of nitrogen, 2-naphthaldehyde (0.2 mmol), HBpin (1.5 equiv) and THF (2 mL) were added by syringe. Then, Hg(0) (100 equiv.) was added and the resulting mixture was stirred under nitrogen atmosphere with sufficient time. The reaction mixture was stirred at 25 °C for 1h. extracted with EtOAc (3 × 20mL). The combined organic phases were dried over anhydrous Na_2SO_4 and concentrated under vacuum.

10. Studying the Reaction profile of Zn-Catalyzed Reduction of 1a

Kinetic studies were performed by treating **1a** (0.2 mmol) with $Zn(OAc)_2$ (0.002mmol, 1mol %), HBpin (1.5 equiv) and THF (2 mL) at 45 °C. The yield of **2a** and the recovery of **1a** were determined by ^1H NMR analysis using 1,3,5-trimethoxybenzene

as an internal standard. The data points for reaction profiles were collected by performing multiple batches of the reaction with different reaction times.

Table S5. Studying the Reaction profile of $Zn(OAc)_2$ Catalyzed Reduction of 2-Naphthaldehyde.

Time (h)	Yield of **2a** (%)	Recovery of **1a** (%)
0	0	100
0.085	28	70
0.17	74	23
0.33	86	12
0.5	88	7
0.75	91	4
1	94	0
1.5	94	0
2	94	0

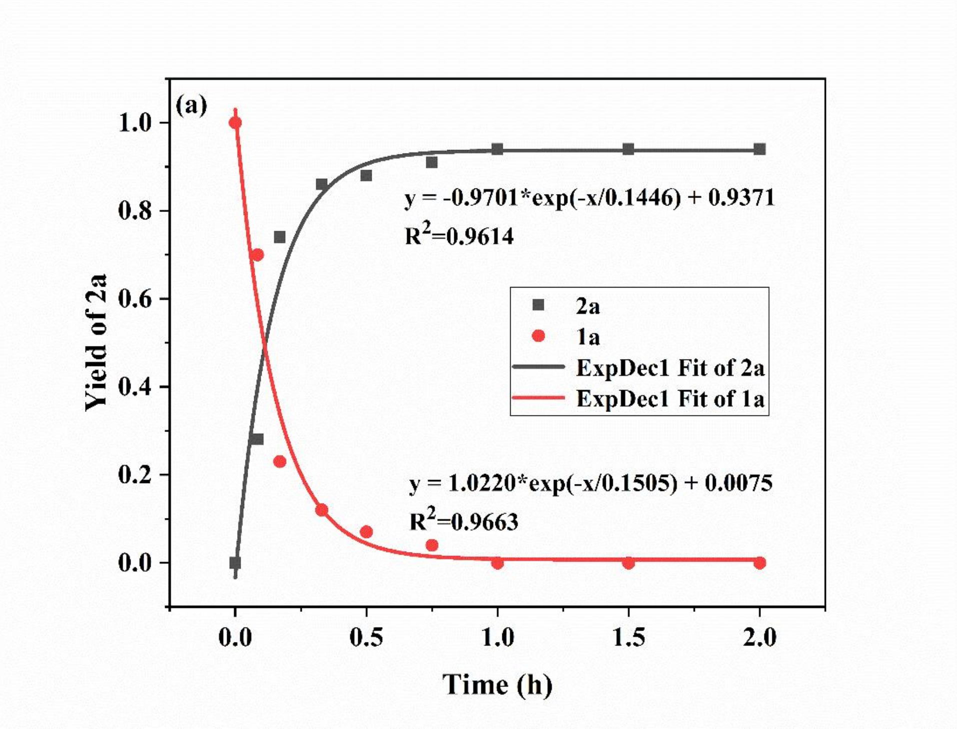

Figure S1. Reaction profile for $Zn(OAc)_2$ hydrogenation of 2-Naphthalene formaldehyde

11. Kinetic Experiments by Initial Rate Measurements for Zn-Catalyzed Reduction of 1a

Procedure for determining the order in Substrate 1a

Substrate **1a** (0.1~0.6 mmol), and $Zn(OAc)_2$ (0.002 mmol) were added to an oven dried Schlenk tube under atmosphere of nitrogen. HBpin (1.5 equiv) and THF (2 mL) were added by syringe. The reaction mixture was stirred at 25 °C for 16 min. The reaction was quenched and 1,3,5-trimethoxy benzene (0.0336, 0.20 mmol) was added as an internal standard for ^{1}HNMR analysis. Plots of the initial rate data for **2a** are shown below.

Table S6. Initial Rate Data Obtained by Variation of the Concentration of 1a

Entry	1a	1a [M]	Initial rate [M/min]
1	0.1mmol	0.04892	0.00018346
2	0.2mmol	0.09784	0.0011619
3	0.3mmol	0.1467	0.002018
4	0.4mmol	0.1956	0.00391389
5	0.5mmol	0.2446	0.00535
6	0.6mmol	0.2935	0.006788

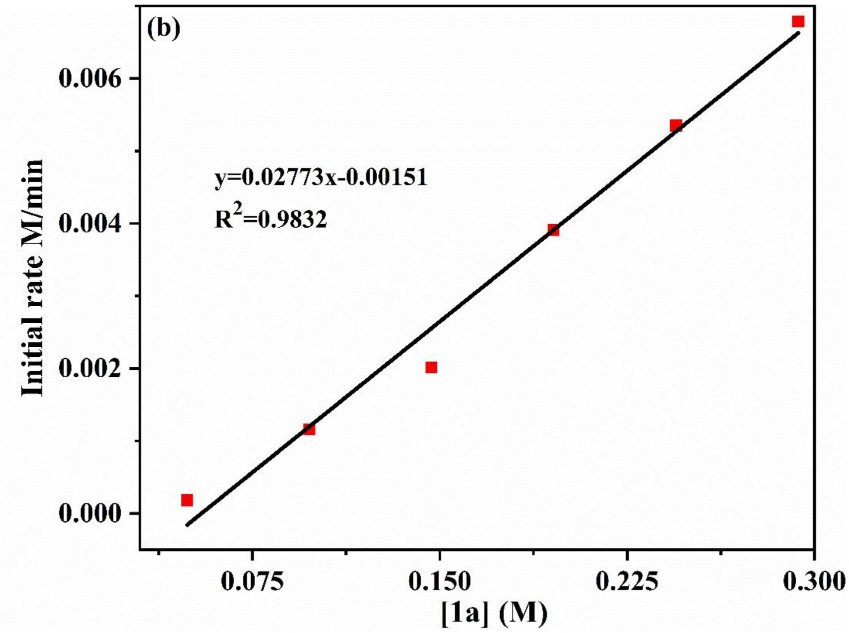

Figure S2. initial reaction rate against the concentration of 1a

Procedure for determining the order in $Zn(OAc)_2$

Substrate **1a** (0.2mmol), and $Zn(OAc)_2$ (1 mol% ~ 6 mol%) were added to an oven dried Schlenk tube under atmosphere of nitrogen. HBpin (1.5 equiv) and THF (2 mL) were added by syringe. The reaction mixture was stirred at 25 °C for 16 min. The reaction was quenched and 1,3,5-trimethoxy benzene (0.0336, 0.20 mmol) was added as an internal standard for ^{1}HNMR analysis. Plots of the initial rate data for **2a** are shown below.

Table S7. Initial Rate Data Obtained by Variation of the Concentration of $Zn(OAc)_2$

Entry	1a	Cat.	[M]	Initial rate [M/min]
1	0.2mmol	1%	0.0009784	0.0000004892
2	0.2mmol	2%	0.001957	0.000004464
3	0.2mmol	3%	0.002935	0.00006604
4	0.2mmol	4%	0.003914	0.00009069
5	0.2mmol	5%	0.004892	0.00011007
6	0.2mmol	6%	0.005870	0.00015777

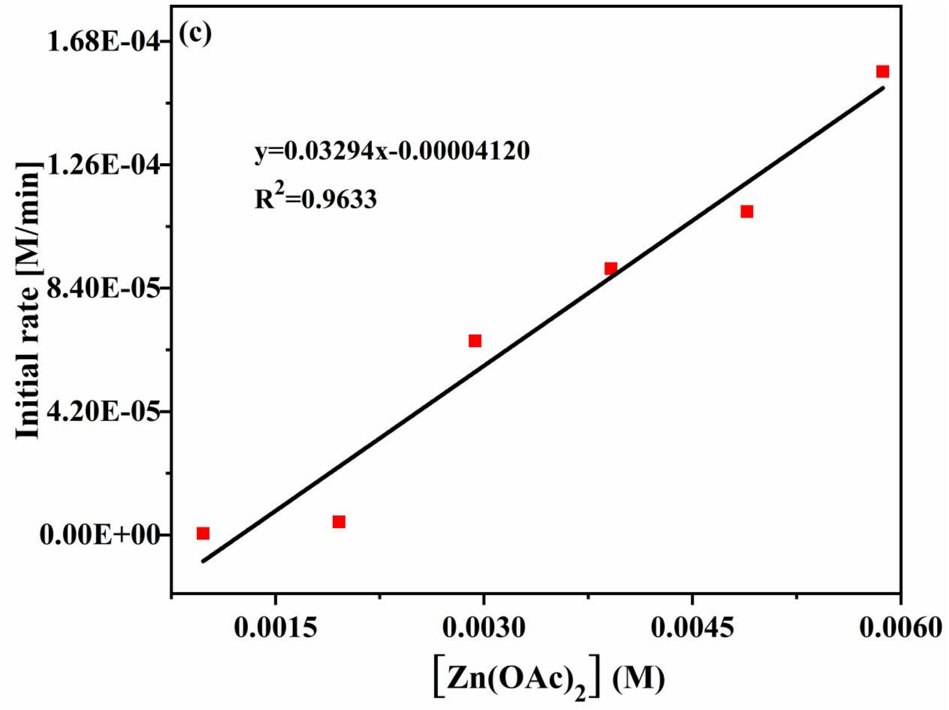

Figure S3. initial reaction rate against the concentration of catalyst of $Zn(OAc)_2$

Procedure for determining the order in HBpin

Substrate **1a** (0.2mmol), and $Zn(OAc)_2$ (1mol%) were added to an oven dried Schlenk tube under atmosphere of nitrogen. HBpin (0.5equiv.~3equiv.) and THF (2 mL) were added by syringe. The reaction mixture was stirred at 25 °C for 16 min. The reaction was quenched and 1,3,5-trimethoxy benzene (0.0336, 0.20 mmol) was added as an internal standard for ^{1}HNMR analysis. Plots of the initial rate data for 2a are shown below.

Table S8. Initial Rate Data Obtained by Variation of the Concentration of HBpin

Entry	1a	HBpin (equiv.)	V (mL)	[M]	Initial rate [M/min]
1	0.2mmol	0.5	2.011	0.04973	0.0002020
2	0.2mmol	1.0	2.029	0.09857	0.0008317
3	0.2mmol	1.5	2.044	0.1468	0.001376
4	0.2mmol	2.0	2.058	0.1944	0.002187
5	0.2mmol	2.5	2.072	0.2413	0.003016
6	0.2mmol	3.0	2.087	0.2875	0.004133

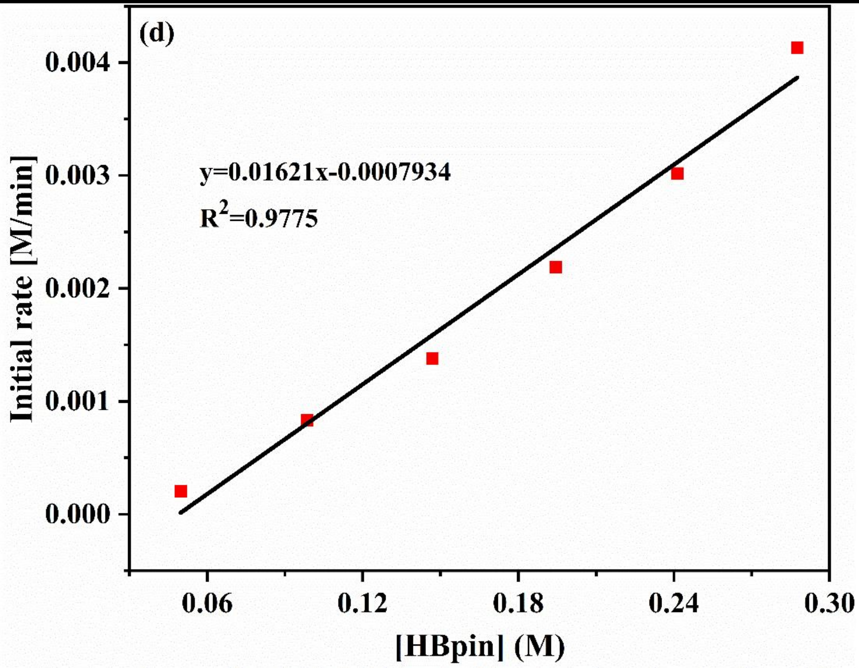

Figure S4. initial reaction rate against the concentration of HBpin.

12. Mechanistic control experiments

Formation of ZnH(OAc) active species

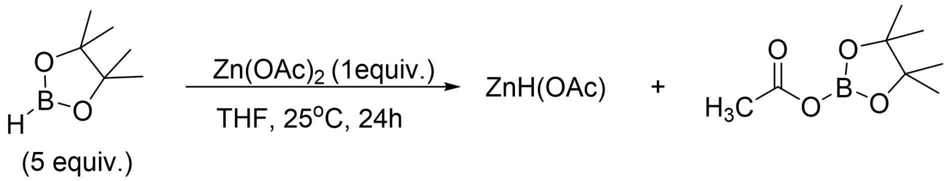

A mixture of HBpin (5.0 equiv.) and $Zn(OAc)_2$ (1.0 equiv.) were added to an oven dried high pressure tube under atmosphere of nitrogen. THF (2 mL) were added by syringe. The reaction mixture was stirred at 25°C for 24 h then evaporation. (^{11}B NMR: 22.15 ppm). [25]

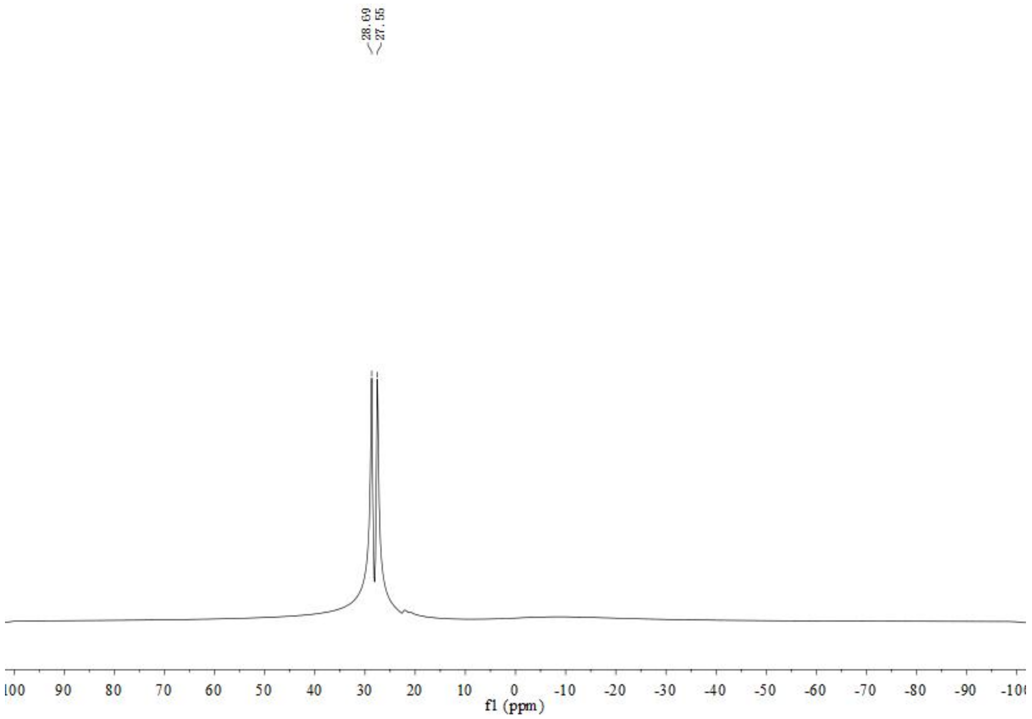

Figure S5. ^{11}B NMR spectra of formation of HBpin

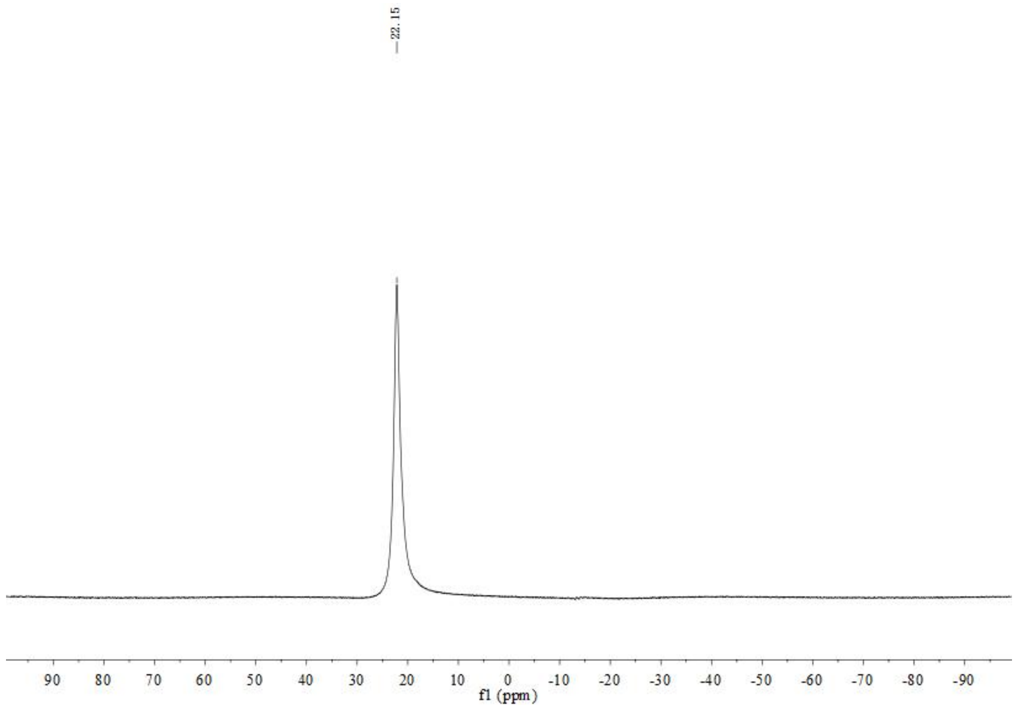

Figure S6.^{11}B NMR spectra of formation of (OAc)Bpin at 25°C.

Supplementary References

1. Thomson, C.; Barber, D. M.; Dixon, D. *Angew. Chem. Int. Ed.,* **2019**, *58*, 2469-2473.
2. Seitz, A-K.; Kohlpaintner, P. J.; Lingen, T.; Dyga, M.; Sprang, F.; Zirbes, M.; Waldvogel, S. R.; Dooßen, L. J. *Angew. Chem. Int. Ed.,* **2022**, *61*. DOI: 10.1002/ anie. 202117563.
3. Thomson, C, J.; Barber, D, M.; Dixon, D, J. *Angew. Chem. Int. Ed.* **2019**, *58*, 2469-2473.
4. Li, P.; Xiao, G.; Zhao, Y.; Su, H.; *ASC. Catal.* **2020**, *10*, 3640-3649.
5. Yatabe, T.; Mizuno, N.; Yamaguchi, K. *ACS. Catal.* **2018**, *8*, 11564-11569.
6. Luo, Y.; Zhou, Y.; Fu, J.; Zhu, H-L. *RSC, Adv.* **2014**, *4*, 23904-23913.
7. Bellier, Q.; Pégaz, S.; Aronica, C.; Guennic, B. L.; Andraud, C.; Maury, O.; *Org. Lett.* **2011**, *13*, 22-25.
8. Benn, K.; Nicholson, K.; Langer, T.; Thomas, S. *Chem. Commun.* **2021**, *57*, 9406-9409.
9. Prasanna, R.; Guha, S.; Sekar, G. *Org. Lett.* **2019**, *21*, 2650-2653.
10. Chen, Z.; Chen, G.; Aboo, A. H.; Lggo, J.; Xiao, J. *Asian J. Org. Chem.* **2020**, *9*, 1174-1178.
11. Wang, J.; Guo, Y.; Li, S.; Chen, X. *Synlett*. **2021**, *32*, 1104-1108.
12. Schmitzer, A. R.; Franceschi, S.; Perez, E.; Rico-Lattes, I.; Lattes, A.; Thion, L.; Erard, M.; Vidal, C. *J. Am. Chem. Soc*. **2001**, *123*, 5956.
13. Tondreau, A. M.; Lobkovsky, E.; Chirik, P. J. *Org. Lett.* **2008**, *10*, 2789-2792.
14. Rooden, E. J.; Kohsiek, M.; Kreekel, R.; Esbroeck, A. C. M.; Nieuwendijk, A. M. C. H.; Janssen, A. P. A.; Berg, R. J. B. H. N.; Overkleeft, H. S.; Stelt, M. *Chem Asian J.* **2018**,*13*, 3491-3500.
15. Gogoi, G.; Saikia, P.; Baruah, M.; Lee, S.; Park, Y-B.; Dutta, R.; Bania, K. K. *Micropor Mesopor Mat.* **2021**, *326*, 111392.
16. Zhu, Z.; Dai, P.; Wu, Z.; Xue, M.; Yao, Y.; Shen, Q.; Bao, X. *Catalysis*

Communications. **2018**, *112*, 26-30.

17. Kuciński, K.; Hreczycho,G. Eur. *J. Org. Chem*. **2020**, *2020*, 552-555.
18. Korvinson, K. A.; Akula, H. K.; Malinchak, C. T.; Sebastian, D.; Wei, W.;Khandaker, T. A.; Andrzejewska, M. R.; Zajc, B.; Lakshman, M. K. *Adv. Synth. Catal.* **2020**, *362*, 166-176.
19. Battilocchio, C.; Hawkins, J. M.; Ley, S. V.*Org. Lett.* **2013**, *15*, 2278-2281.
20. Zhang, G-Y.; Ruan, S-H.; Li, Y-Y.; Gao, J-X. *Chinese Chem Lett.* **2021**, *4*, 1415-1418.
21. Tamang, S. R.; Bedi, D.; Shafifiei-Haghighi, S.; Smith, C. R.; Crawford, C.; Findlater, M. *Org. Lett*. **2018**, *20*, 6695−6700.
22. Kim, Y. J.; Shin, W. K.; Jaladi, A. K.; An, D. K. *Tetrahedron,* **2018**, *74*, 4236-4231.
23. Bauri, S.; Donthireddy, S. N. R.; Illam, P. M.; Rit, Arnab. *Inorg. Chem.* **2018**, *57*, 14582-14593.
24. Dhokale, B.; Susarryey-Arce, A.; Pekkari, A.; Runemark, A.; Moth-Poulsen, K.; Langhammer, C.; Härelind , H.; Busch, M.; Vandichel, M.; Sundén, H. *Chem Cat Chem.* **2020**, *12*, 1-13.
25. Jang, Y. K.; Magre, M.; Rueping, M. *Org. Lett*. **2019**, *21*, 8349-8352.
26. Yu, Q.; Lu, C.; Zhao, B. *Organometallics*, **2021**, *40*, 2529-2537.
27. Chang, M.-Y.; Tsai, C.-Y.; Wu, M.-H. *Tetrahedron*, **2013**, *69*, 6364-6370.
28. Lu, S.-M.; Gao, Q.; Li, J.; Liu, Y.; Li, C. *Tetrahedron Letters,* **2013**, *54*, 7013-7016.
29. Tang, J.; Dong, W.; Chen, F.; Deng, L.; Xian, M. *Catal. Sci. Technol.,* **2021**, *11*, 5564-5569.
30. Dong, J.; Wang, X.; Wang, Z.; Song, H.; Liu, Y.; Wang, Q. *Chem. Sci*., **2020**, *11*, 1026-1031

13. ^{1}H, ^{13}C and ^{19}F NMR Spectra

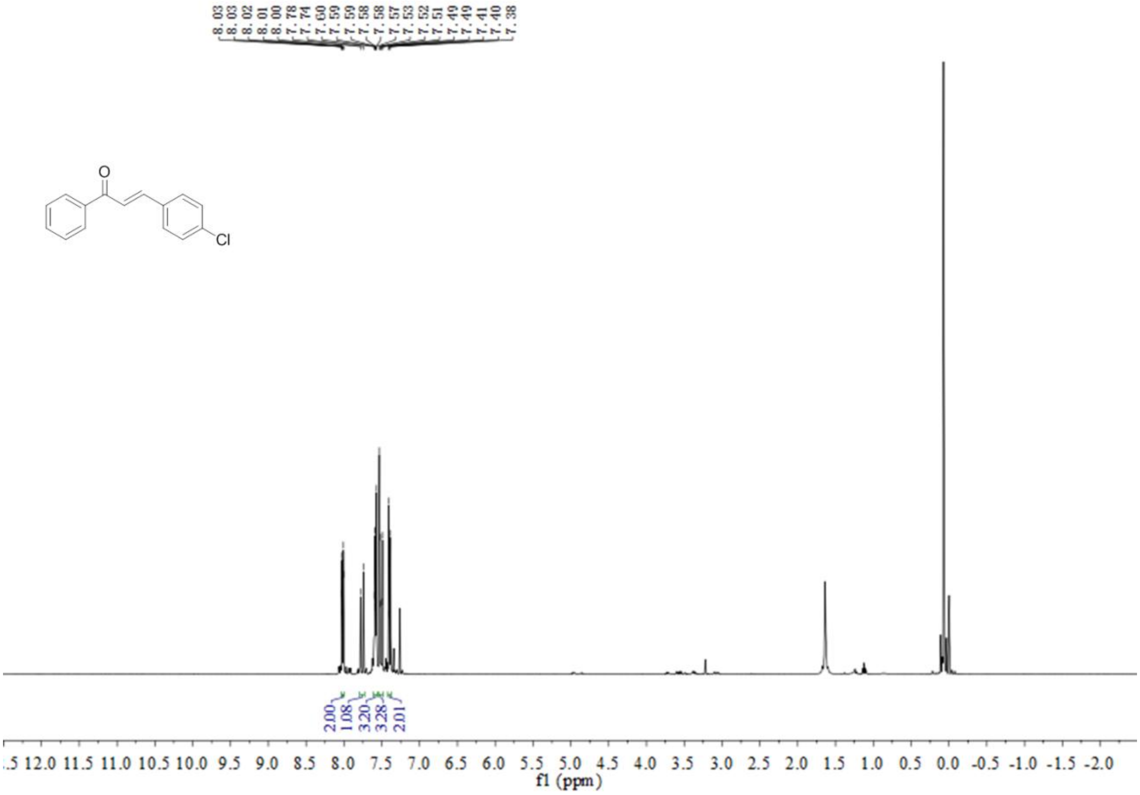

Figure S7. ^{1}H NMR spectrum for compound (E)-3-(4-chlorophenyl)-1-phenylprop-2-en-1-one

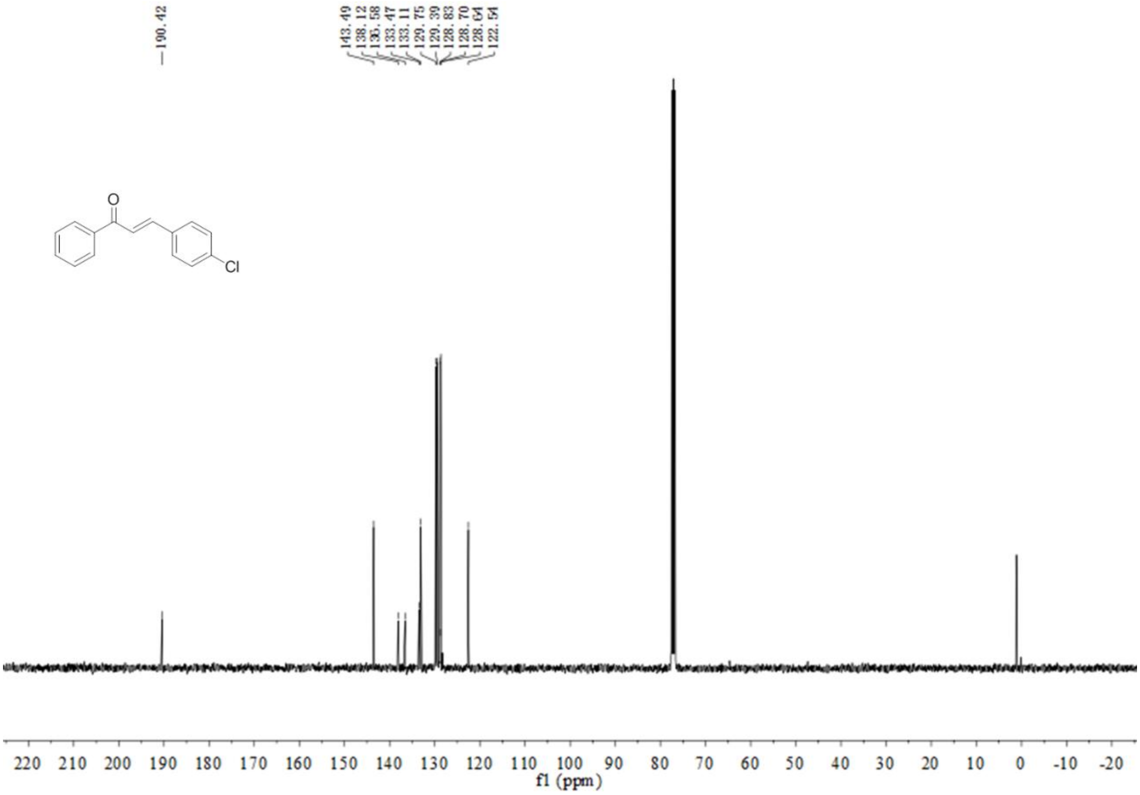

Figure S8. ^{13}C NMR spectrum for compound (E)-3-(4-chlorophenyl)-1-phenylprop-2-en-1-one.

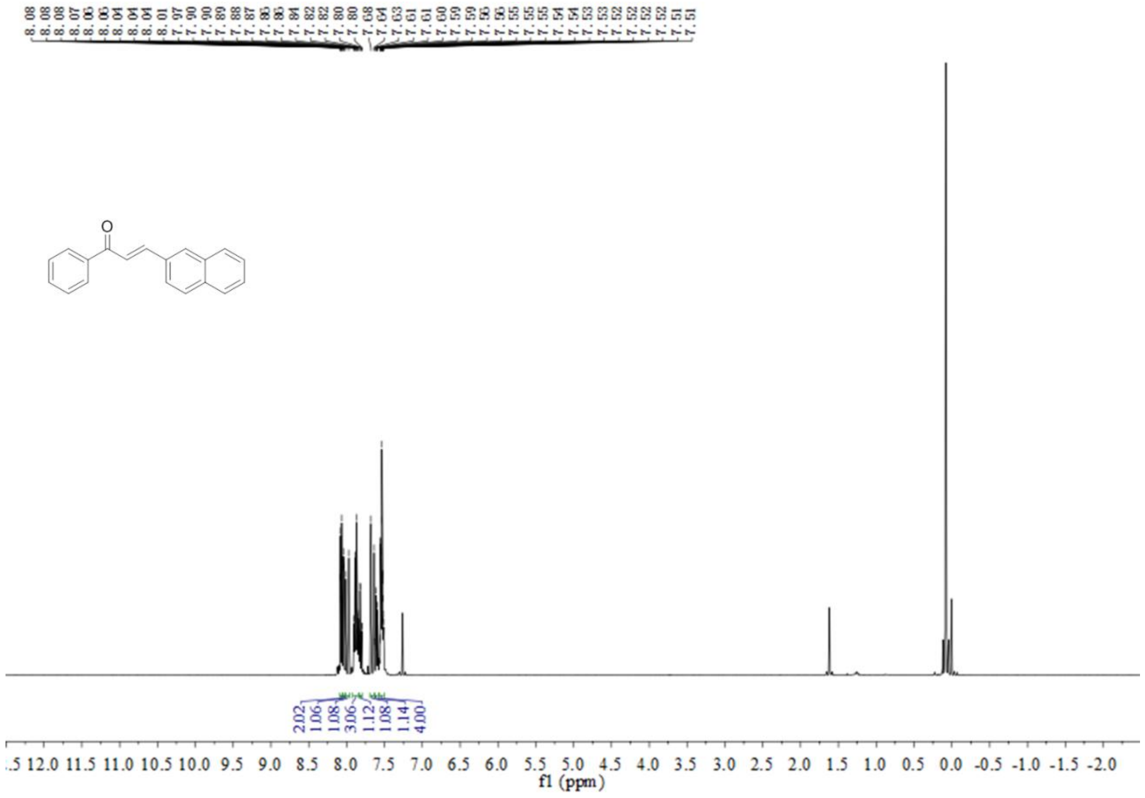

Figure S9. ^{1}H NMR spectrum for compound (E)-3-(naphthalen-2-yl)-1-phenylprop-2-en-1-one

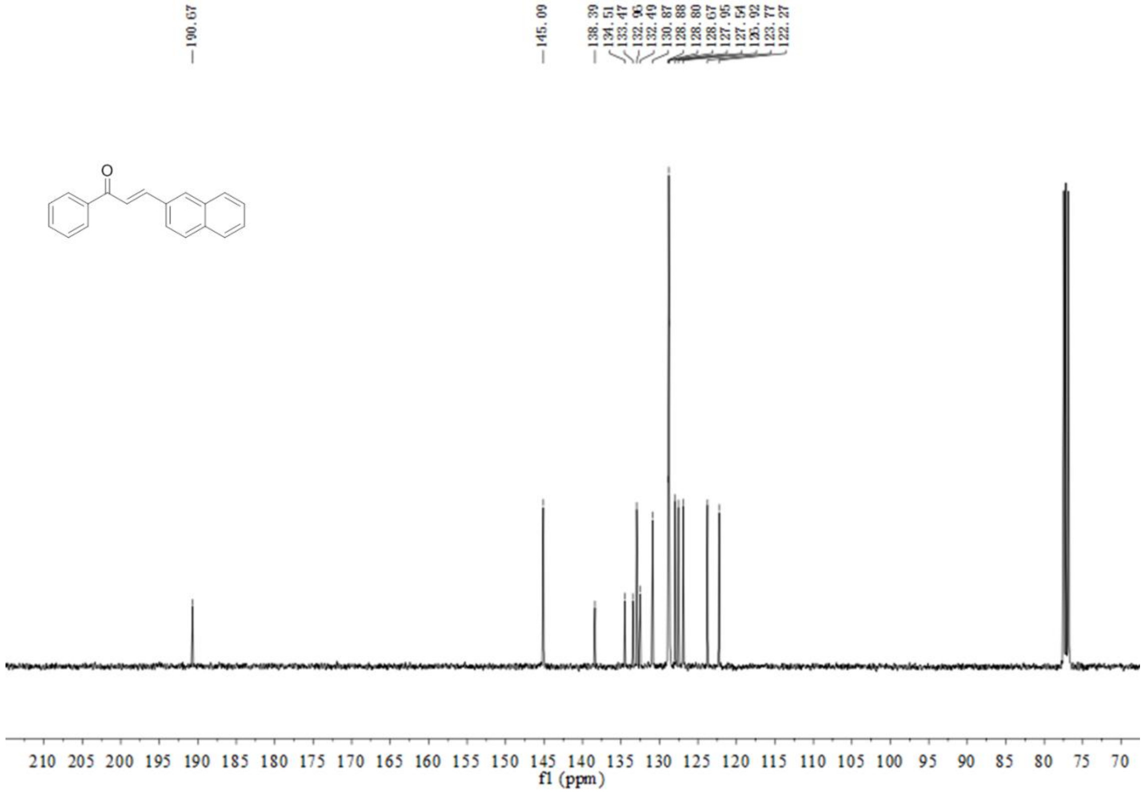

Figure S10. ^{13}C NMR spectrum for compound (E)-3-(naphthalen-2-yl)-1-phenylprop-2-en-1-one

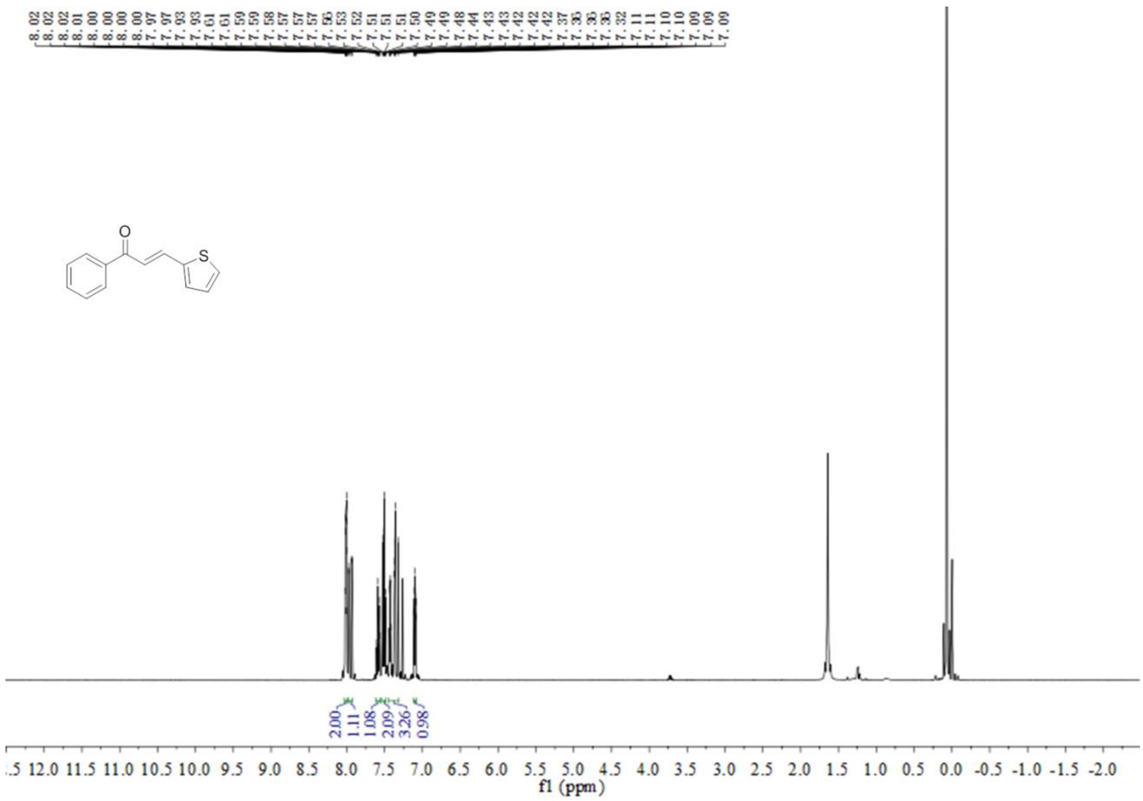

Figure S11. ^{1}H NMR spectrum for compound (E)-1-phenyl-3-(thiophen-2-yl)prop-2-en-1-one

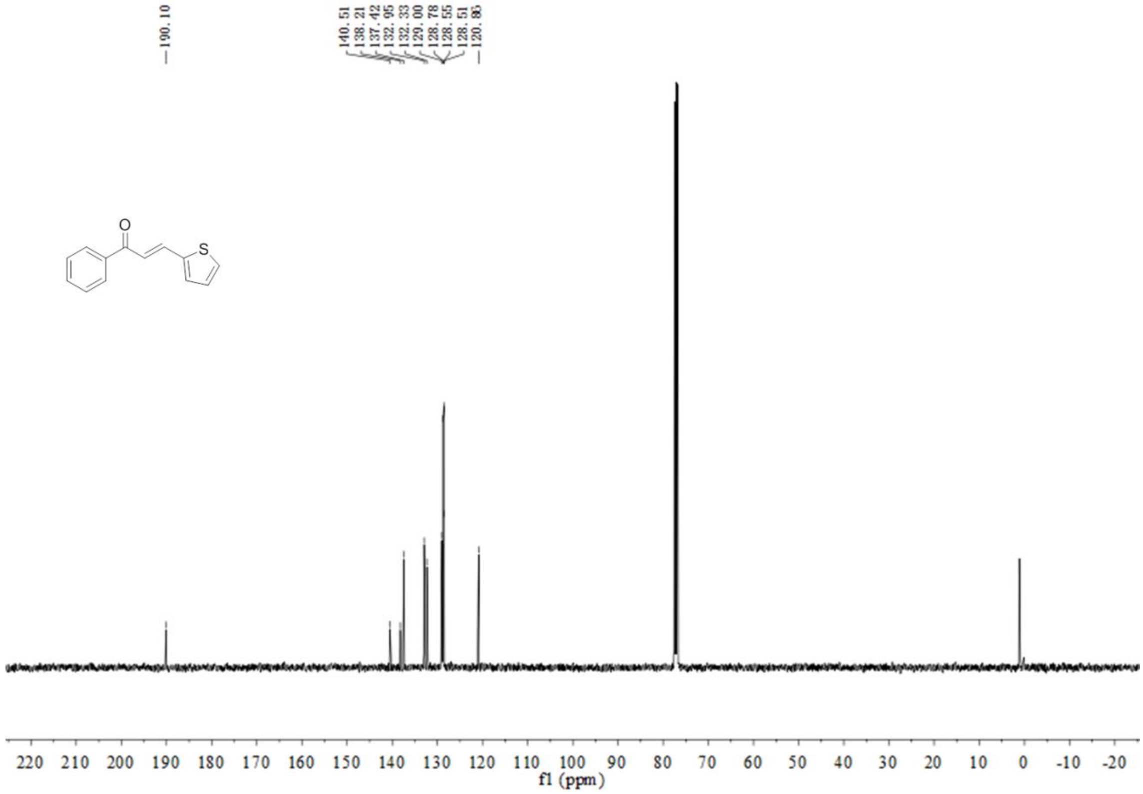

Figure S12. ^{13}C NMR spectrum for compound (E)-1-phenyl-3-(thiophen-2-yl)prop-2-en-1-one

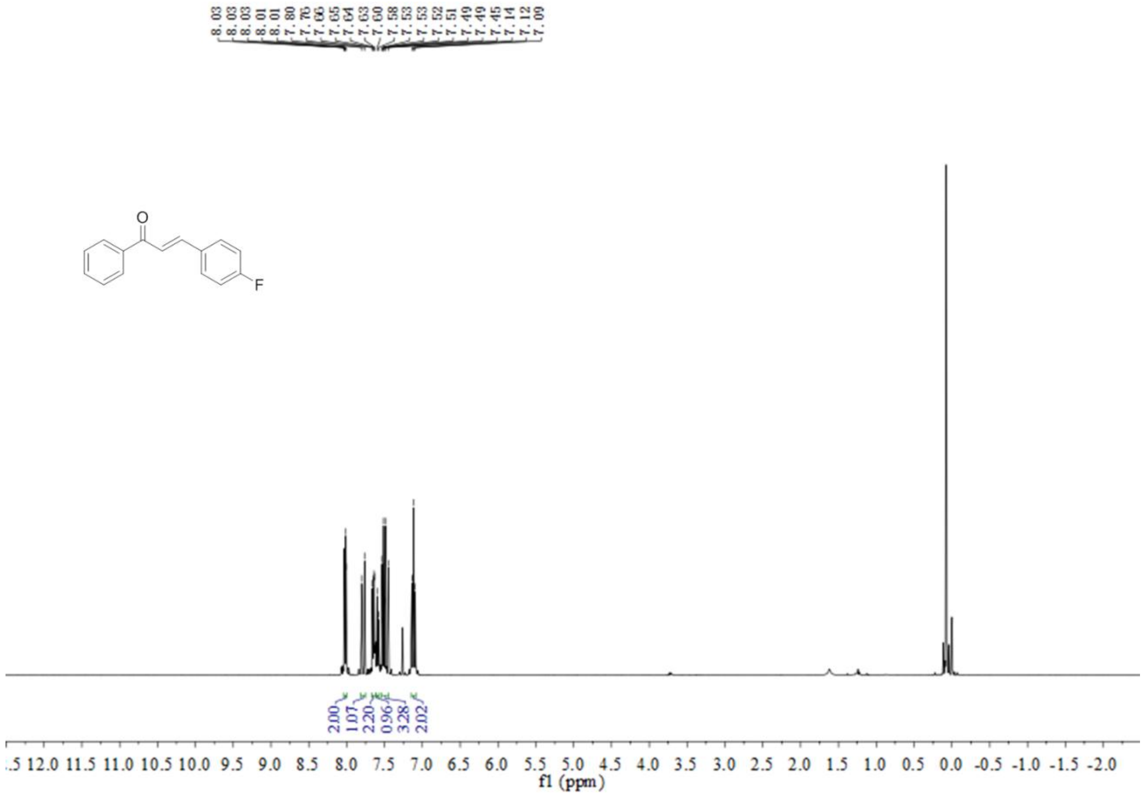

Figure S13. ^{1}H NMR spectrum for compound (E)-3-(4-fluorophenyl)-1-phenylprop-2-en-1-one

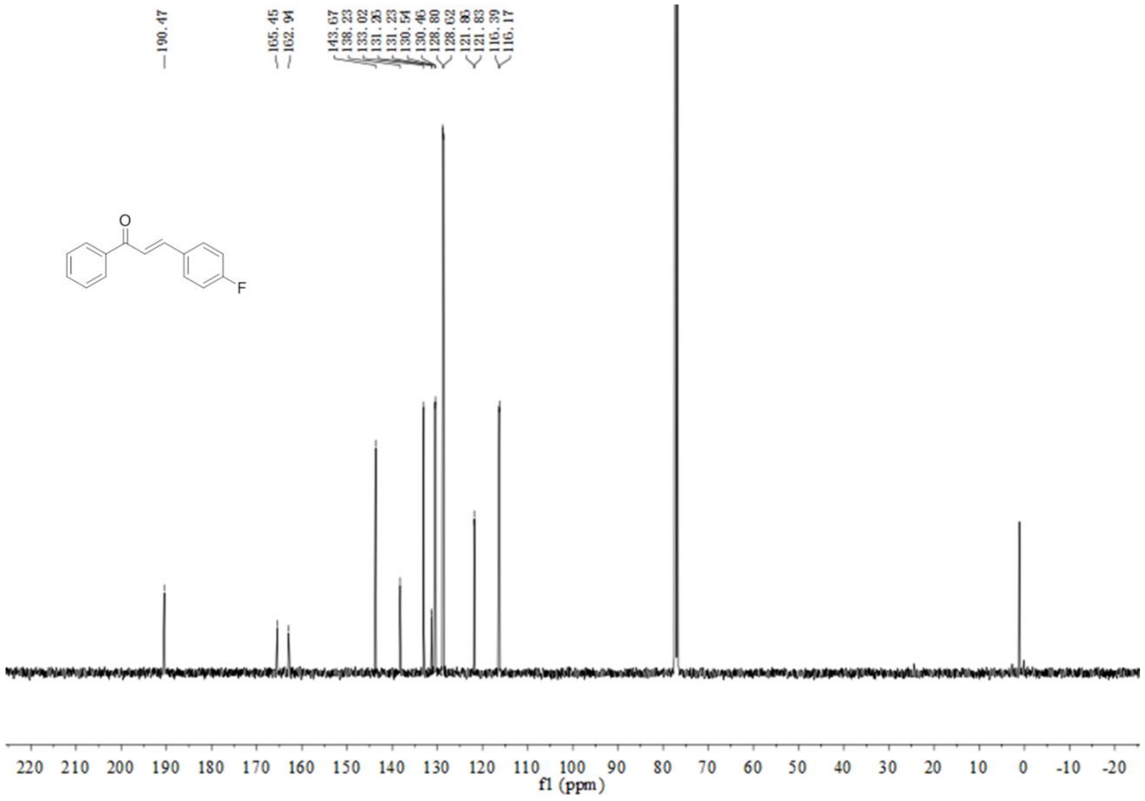

Figure S14. ^{13}C NMR spectrum for compound (E)-3-(4-fluorophenyl)-1-phenylprop-2-en-1-one

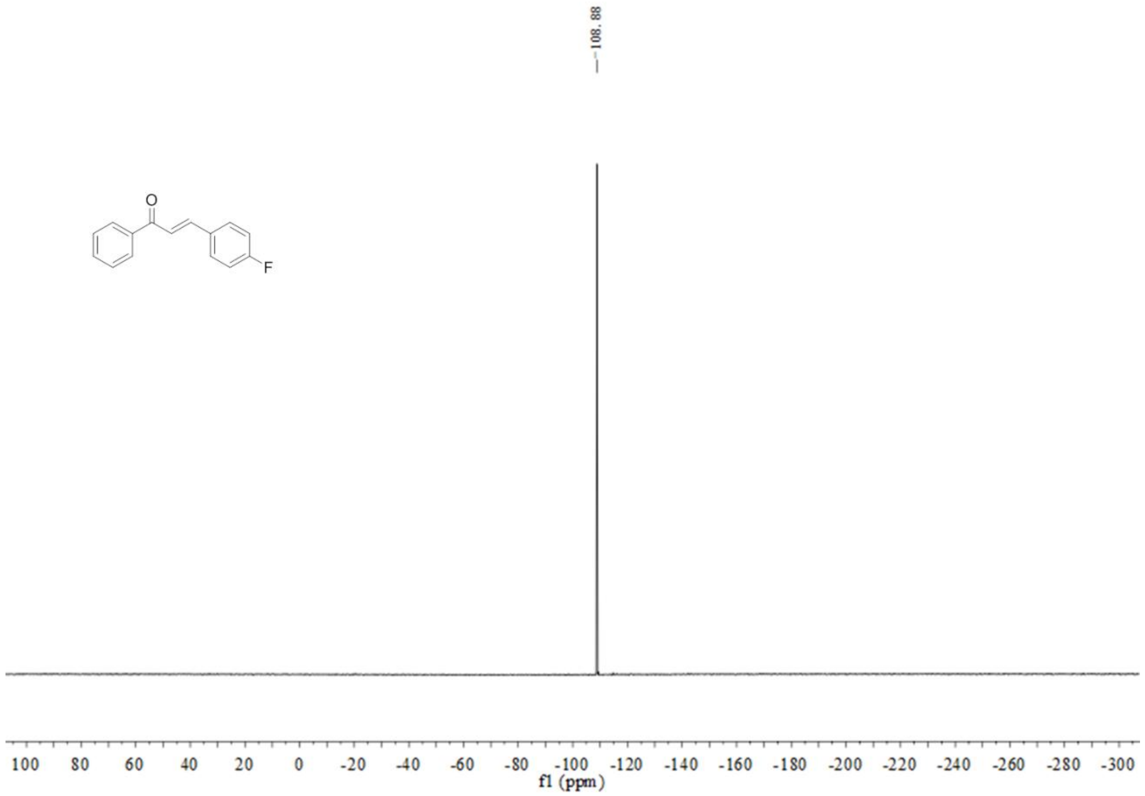

Figure S15. ^{19}F NMR spectrum for compound (E)-3-(4-fluorophenyl)-1-phenylprop-2-en-1-one

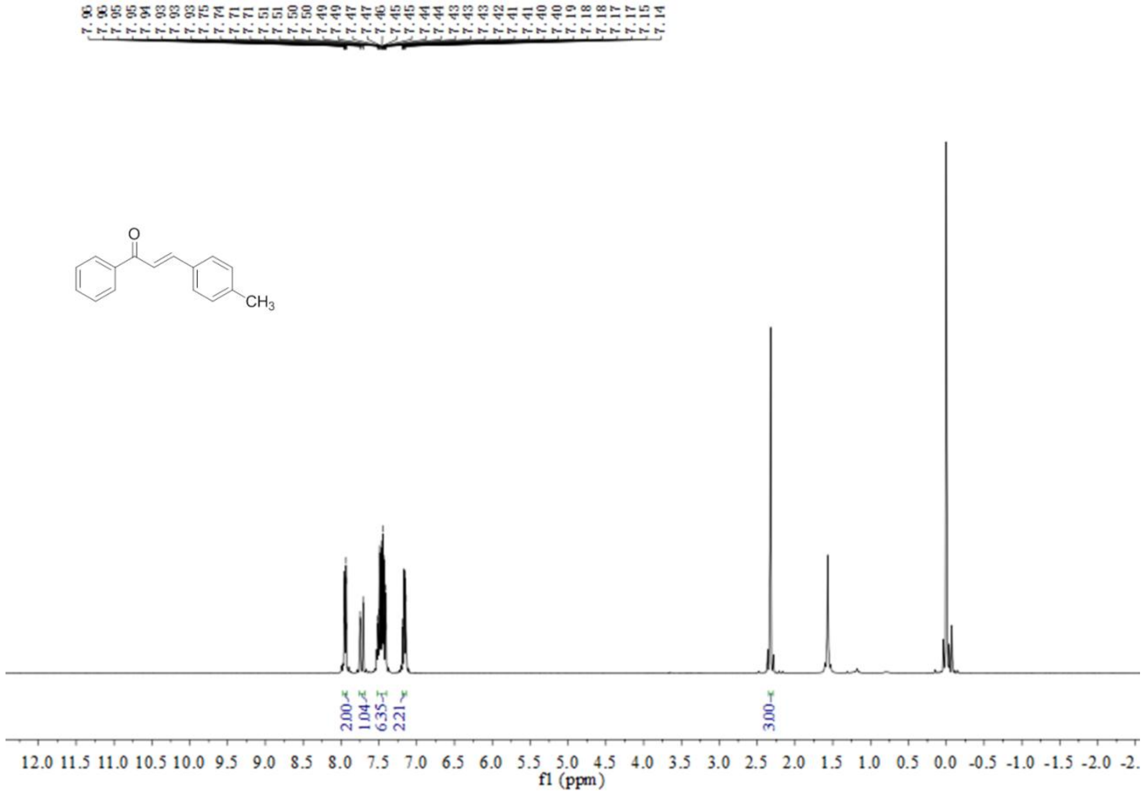

Figure S16. ^1H NMR spectrum for compound (E)-1-phenyl-3-(p-tolyl)prop-2-en-1-one

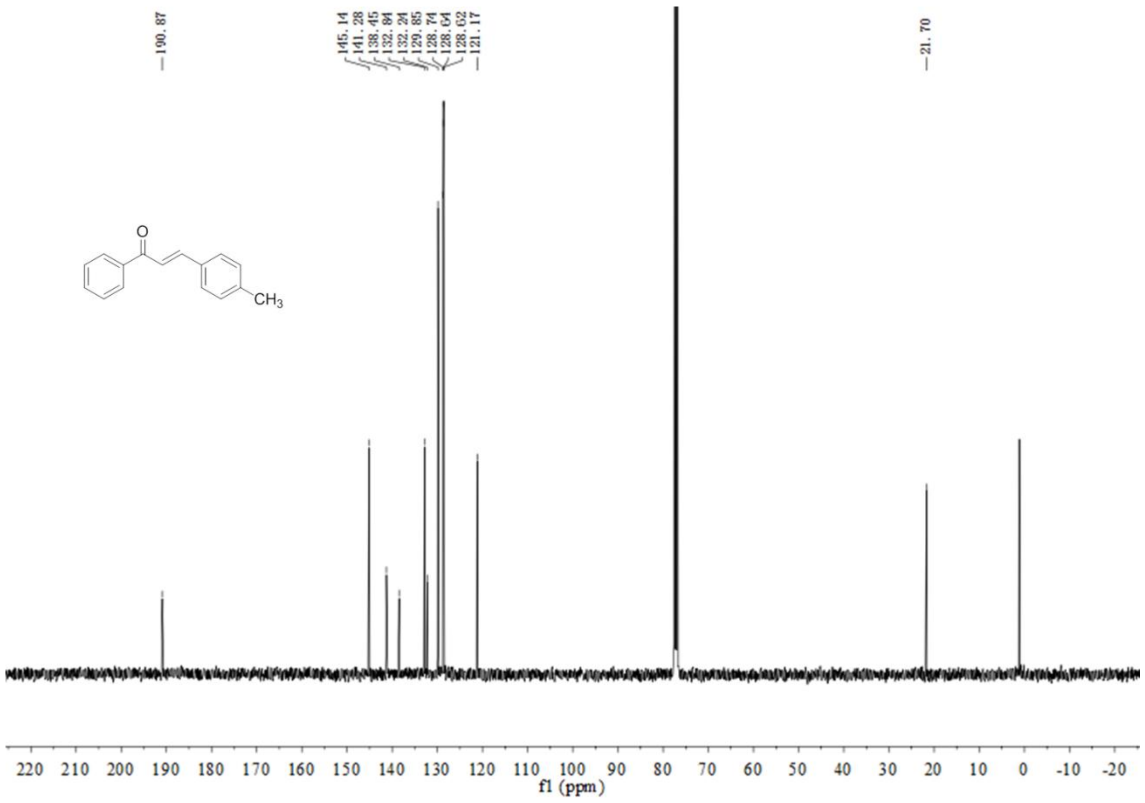

Figure S17. ^{13}C NMR spectrum for compound (E)-1-phenyl-3-(p-tolyl)prop-2-en-1-one

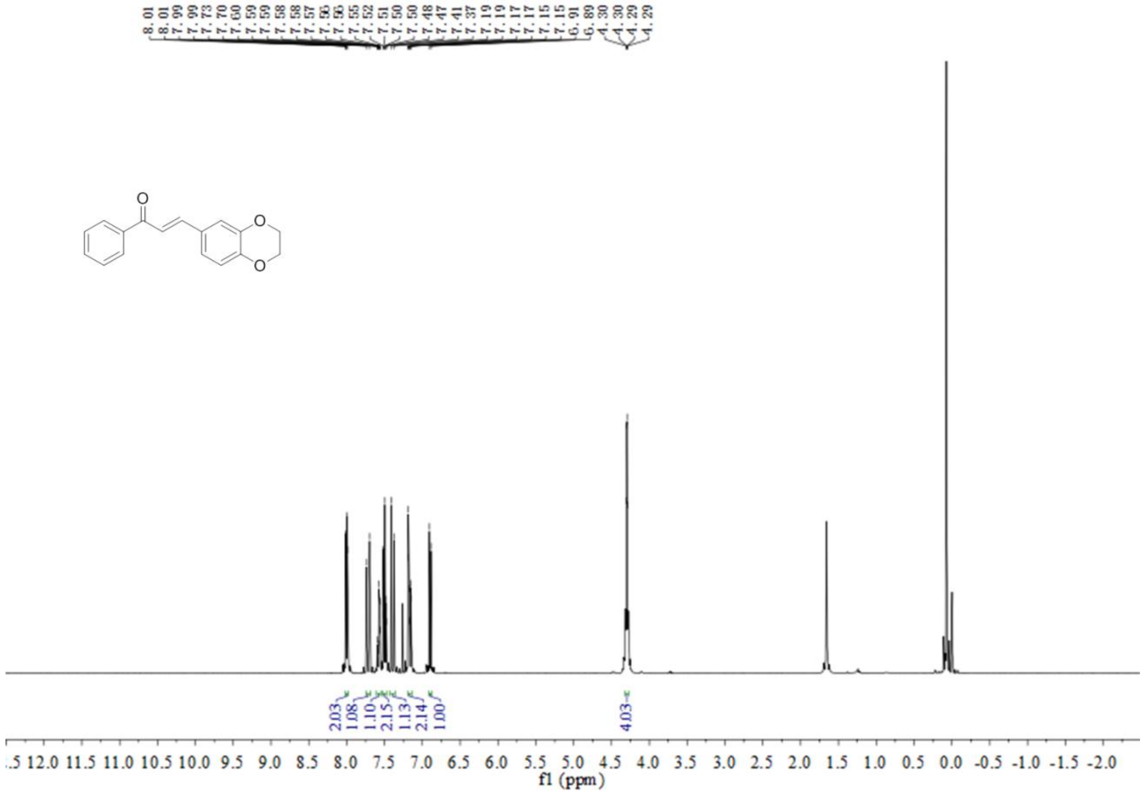

Figure S18. ^{1}H NMR spectrum for compound (E)-3-(2,3-dihydrobenzo[b][1,4]dioxin-6-yl)-1-phenylprop-2-en-1-one

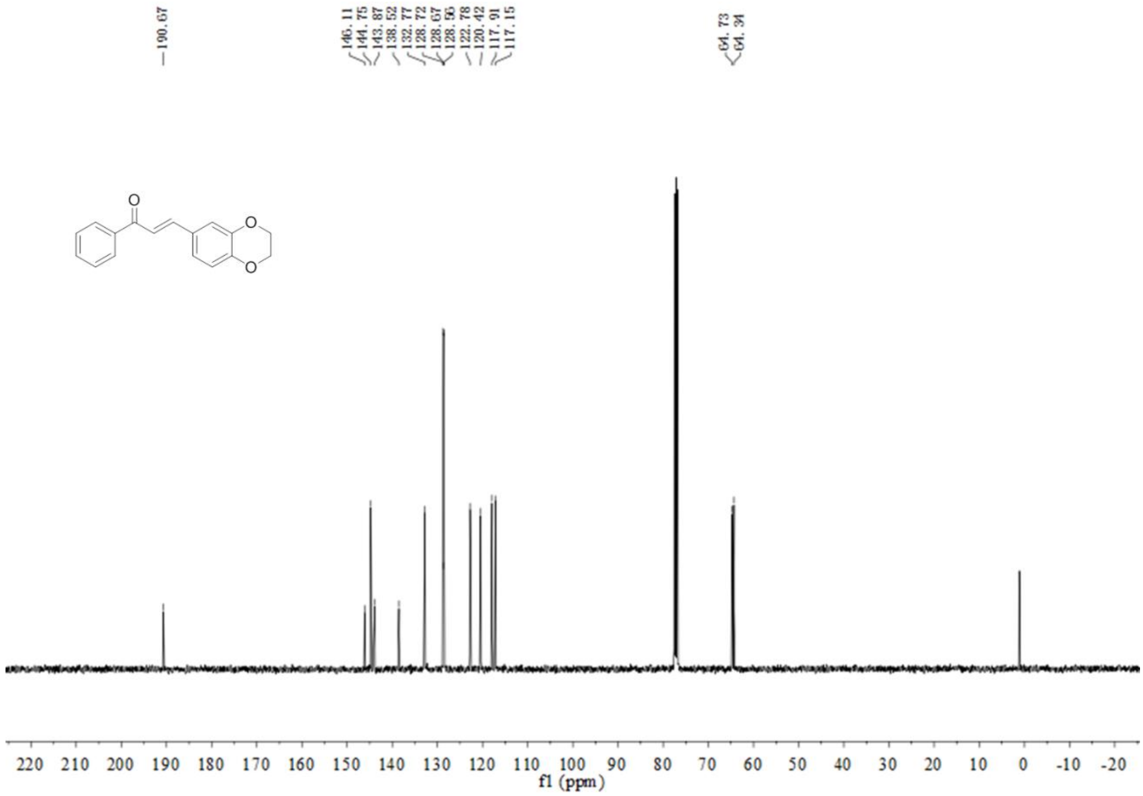

Figure S19. ^{13}C NMR spectrum for compound
(E)-3-(2,3-dihydrobenzo[b][1,4]dioxin-6-yl)-1-phenylprop-2-en-1-one

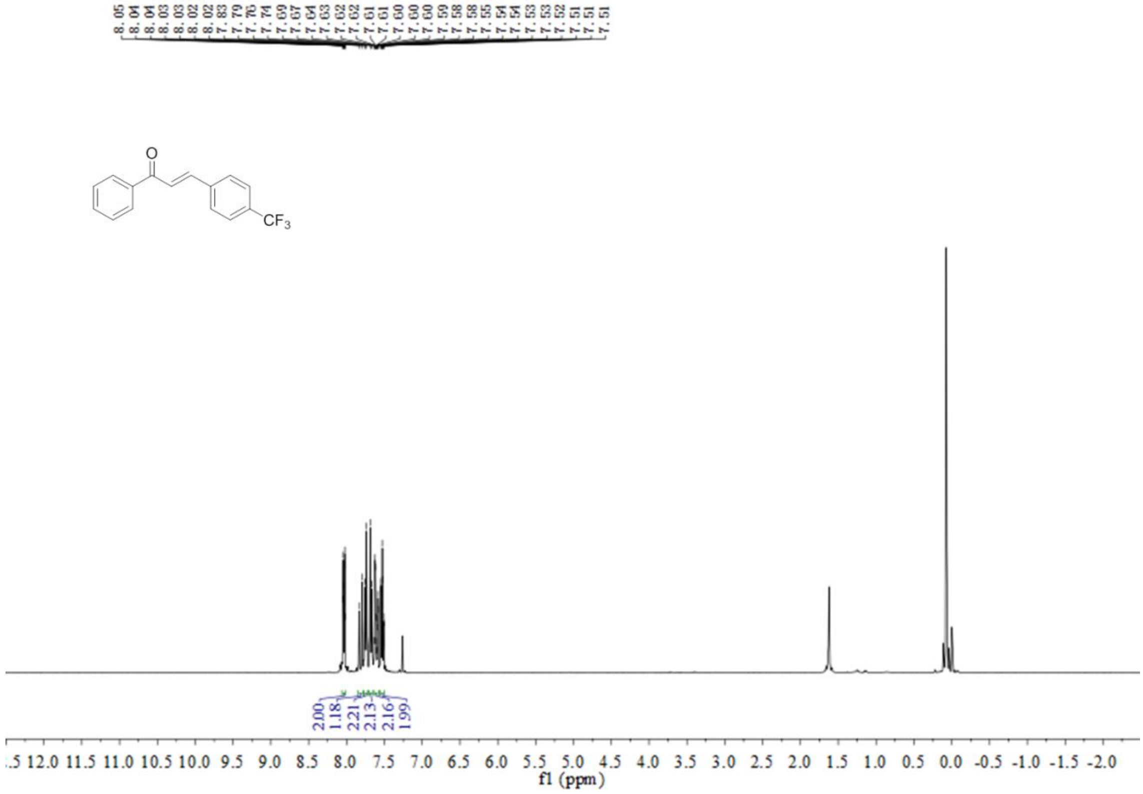

Figure S20. ^{1}H NMR spectrum for compound
(E)-1-phenyl-3-(4-(trifluoromethyl)phenyl)prop-2-en-1-one

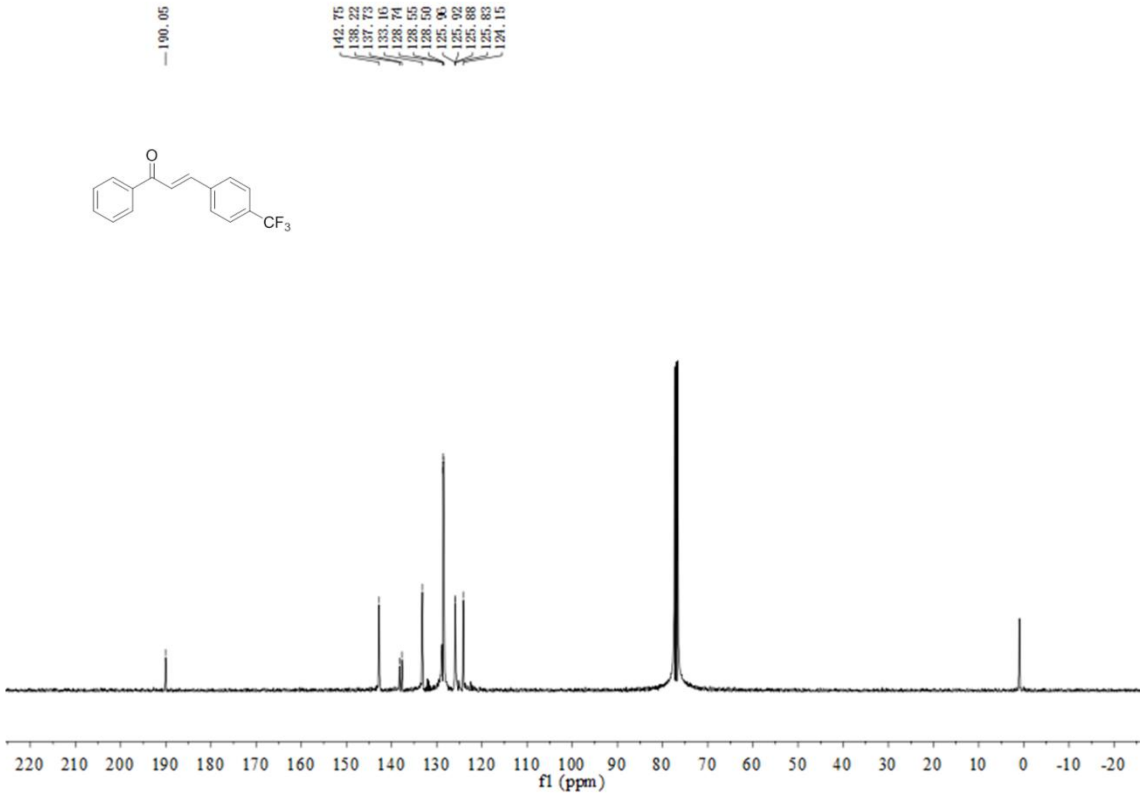

Figure S21. ^{13}CNMR spectrum for compound (E)-1-phenyl-3-(4-(trifluoromethyl)phenyl)prop-2-en-1-one

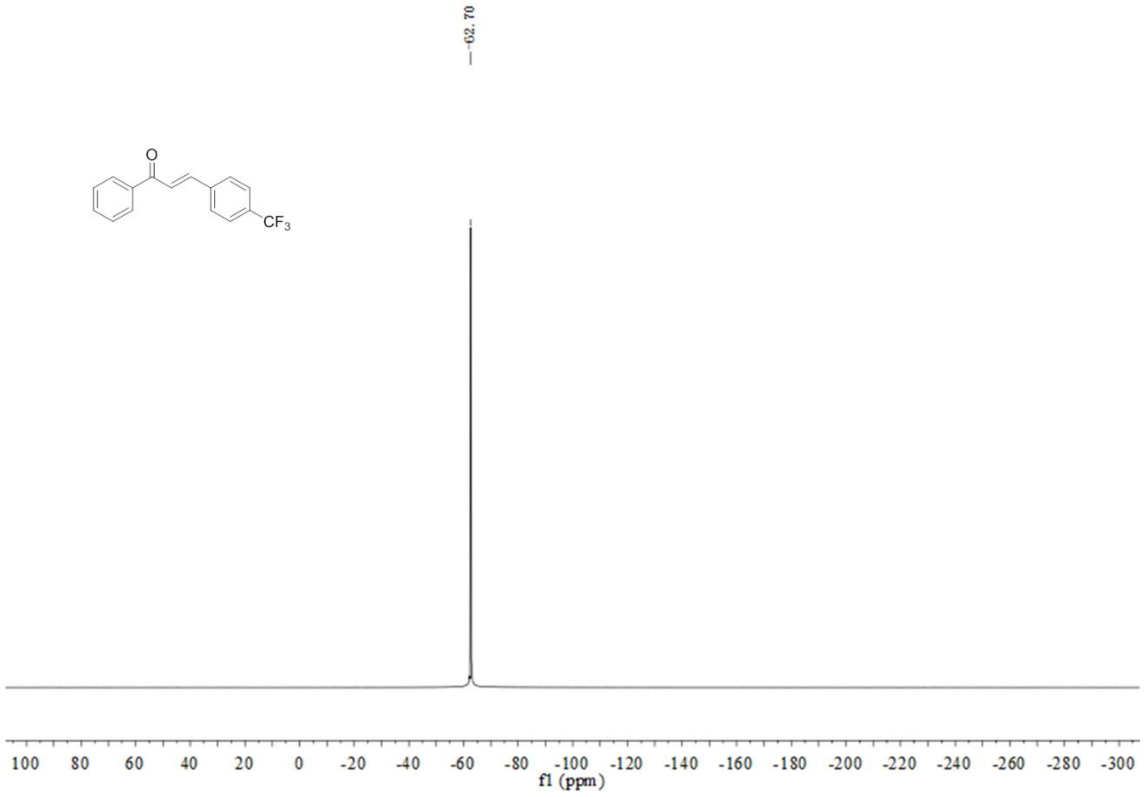

Figure S22. ^{19}F NMR spectrum for compound (E)-1-phenyl-3-(4-(trifluoromethyl)phenyl)prop-2-en-1-one

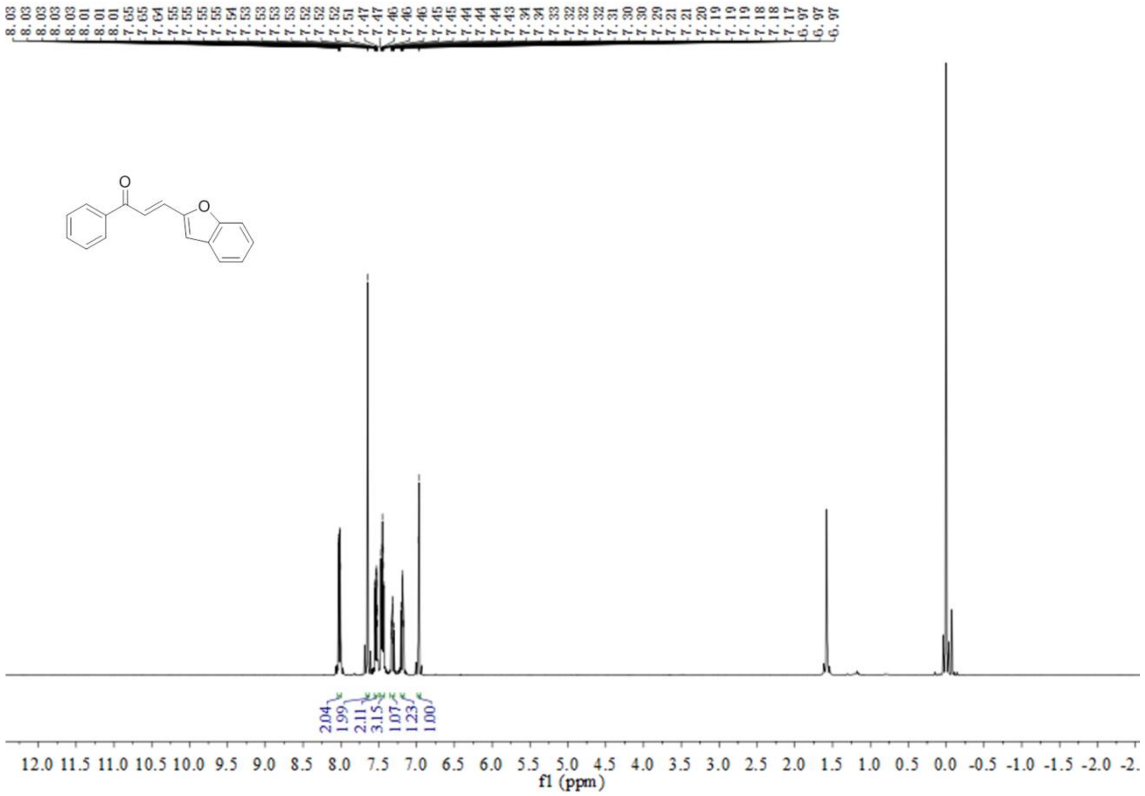

Figure S23. ^{1}H NMR spectrum for compound (E)-3-(benzofuran-2-yl)-1-phenylprop-2-en-1-one

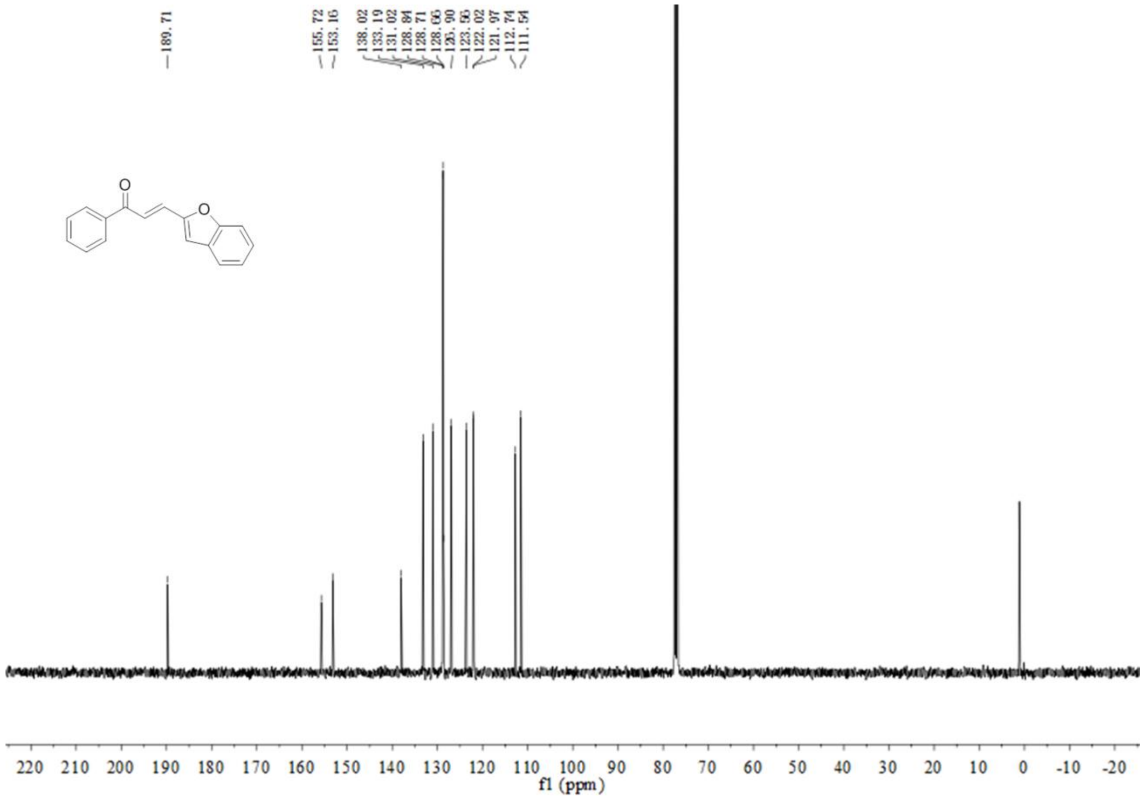

Figure S24. ^{13}C NMR spectrum for compound (E)-3-(benzofuran-2-yl)-1-phenylprop-2-en-1-one

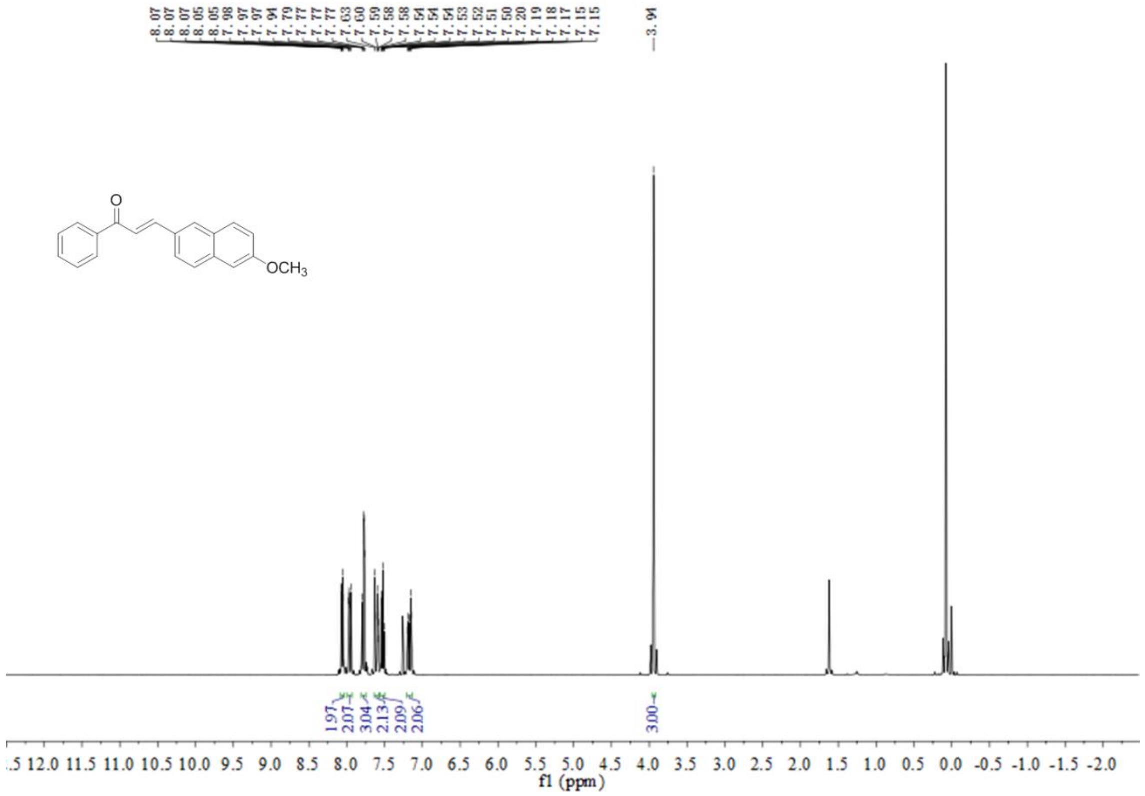

Figure S25. ^{1}H NMR spectrum for compound (E)-3-(6-methoxynaphthalen-2-yl)-1-phenylprop-2-en-1-one

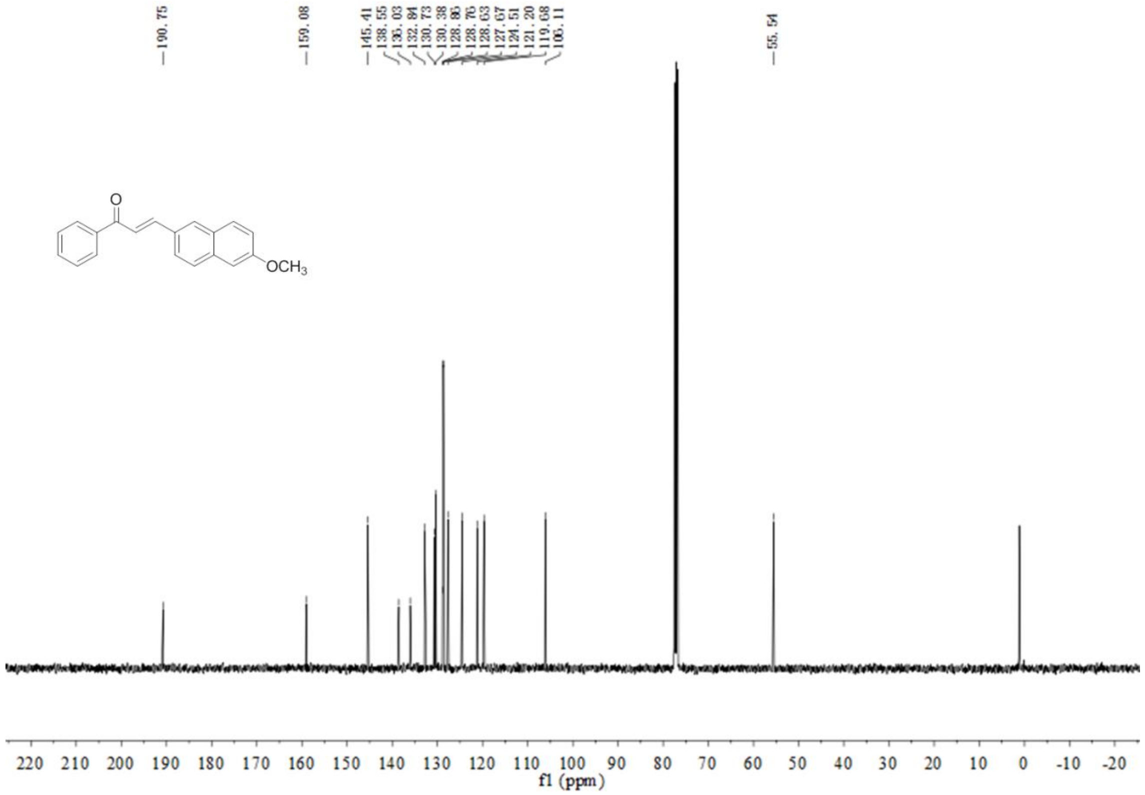

Figure S26. ^{13}C NMR spectrum for compound (E)-3-(6-methoxynaphthalen-2-yl)-1-phenylprop-2-en-1-one

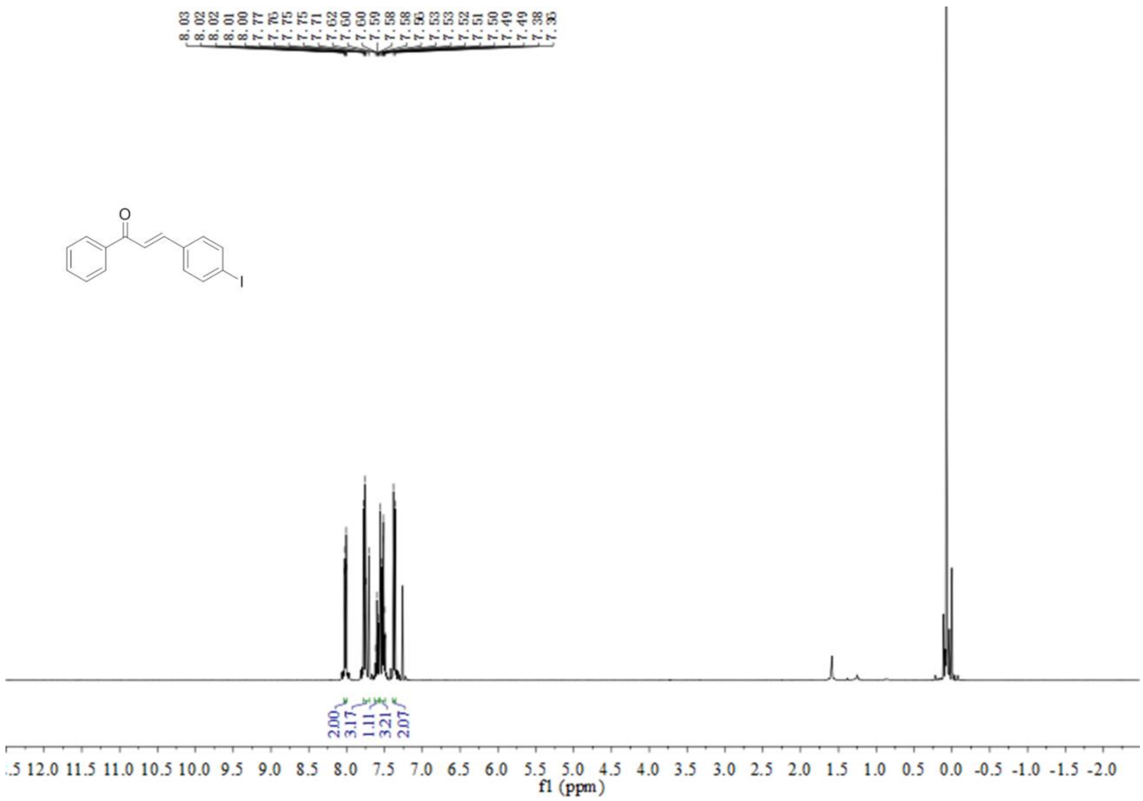

Figure S27. ^{1}H NMR spectrum for compound (E)-3-(4-iodophenyl)-1-phenylprop-2-en-1-one

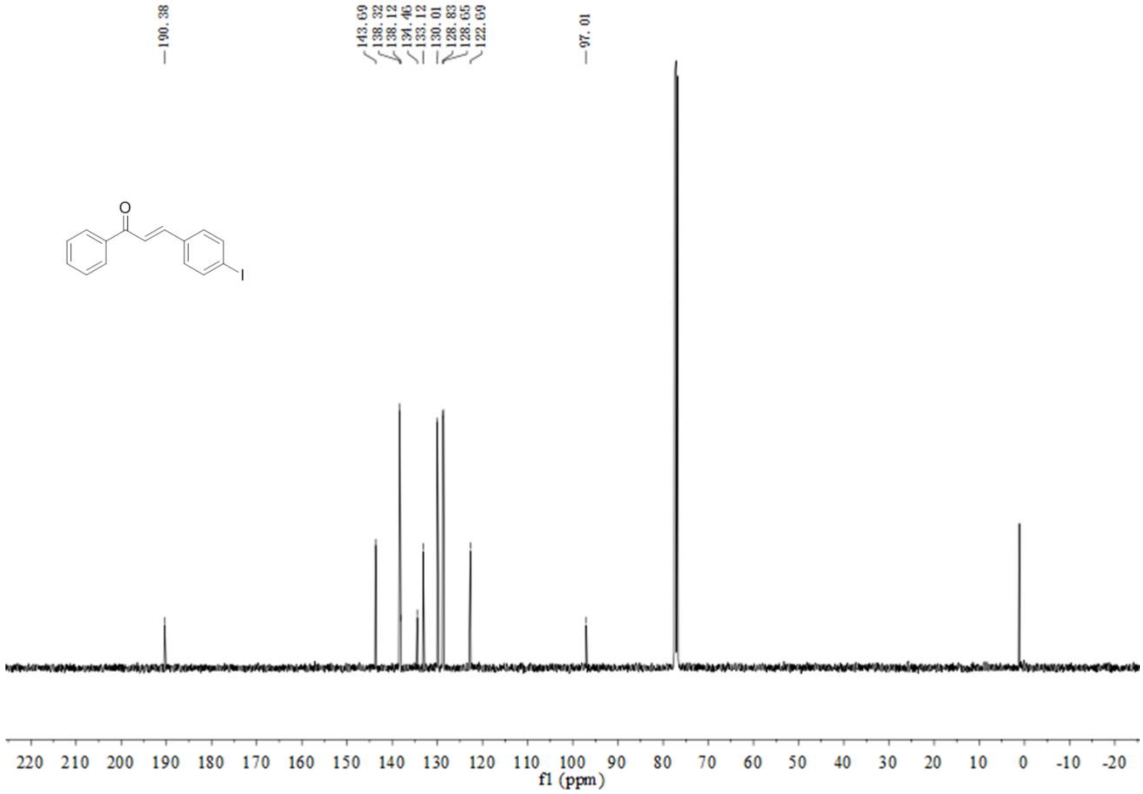

Figure S28. ^{13}C NMR spectrum for compound (E)-3-(4-iodophenyl)-1-phenylprop-2-en-1-one

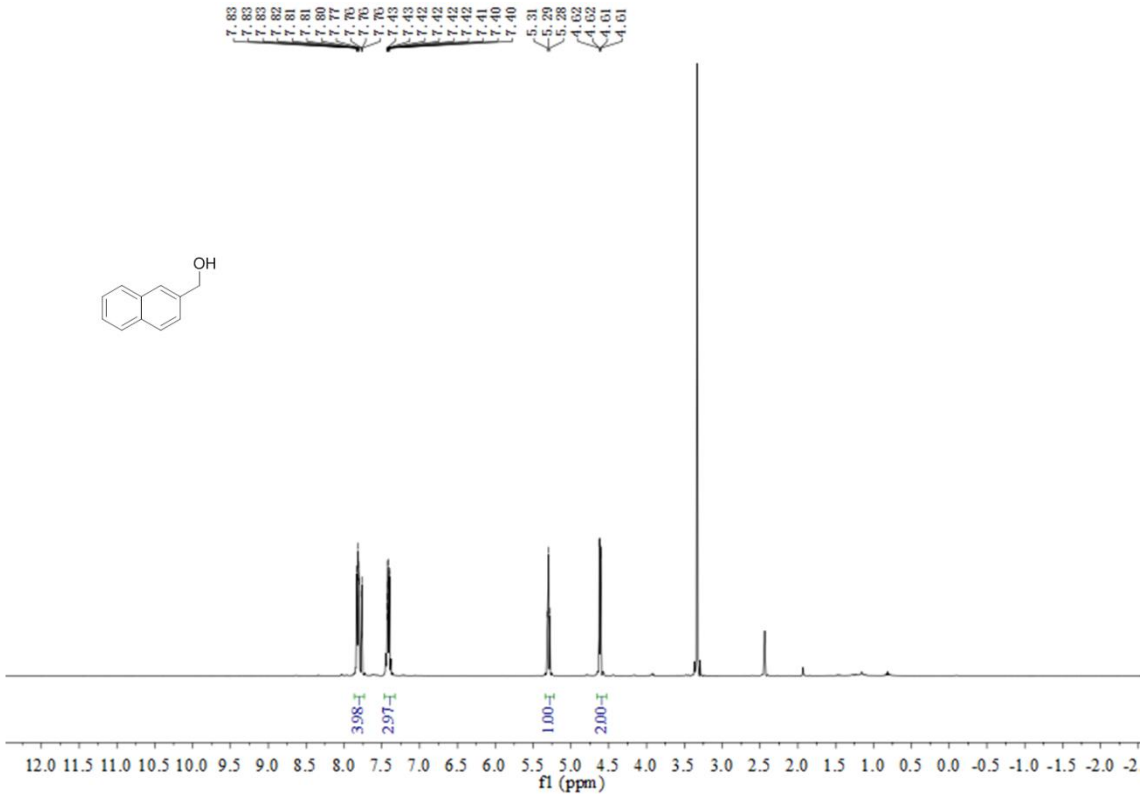

Figure S29. ^{1}H NMR spectrum for compound 2-Naphthalenemethanol (**2a**).

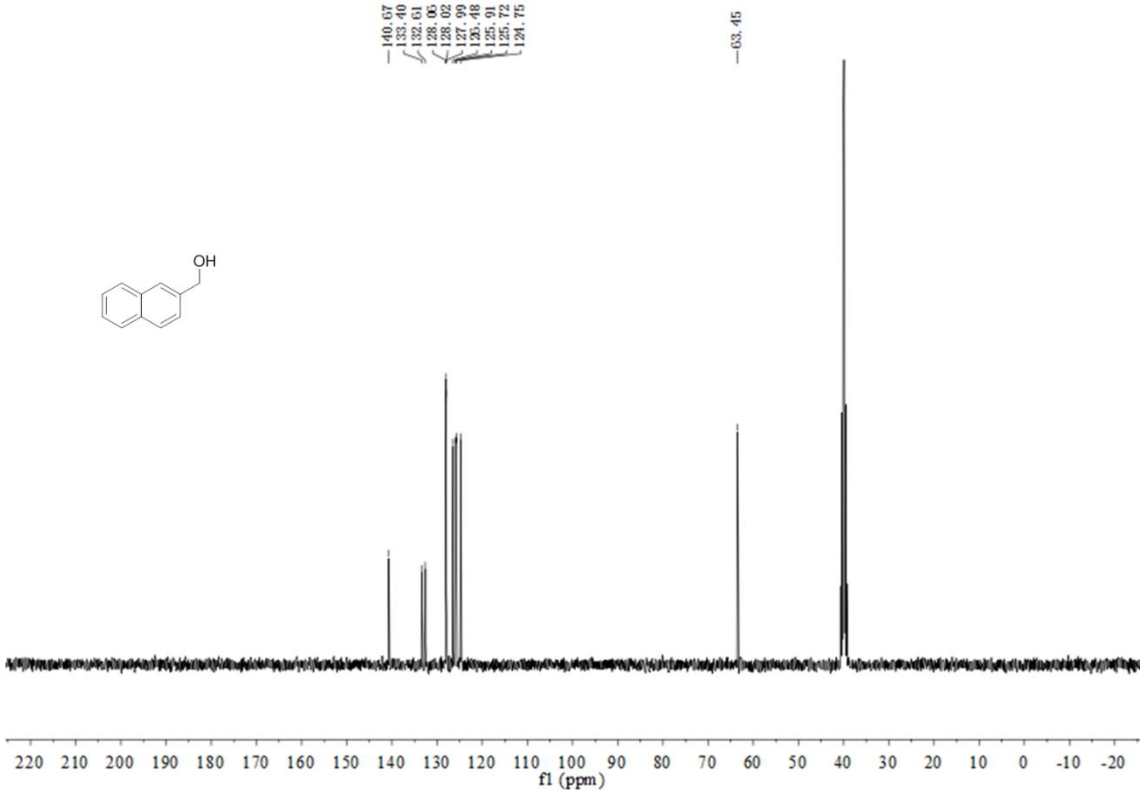

Figure S30. ^{13}C NMR spectrum for compound 2-Naphthalenemethanol (**2a**).

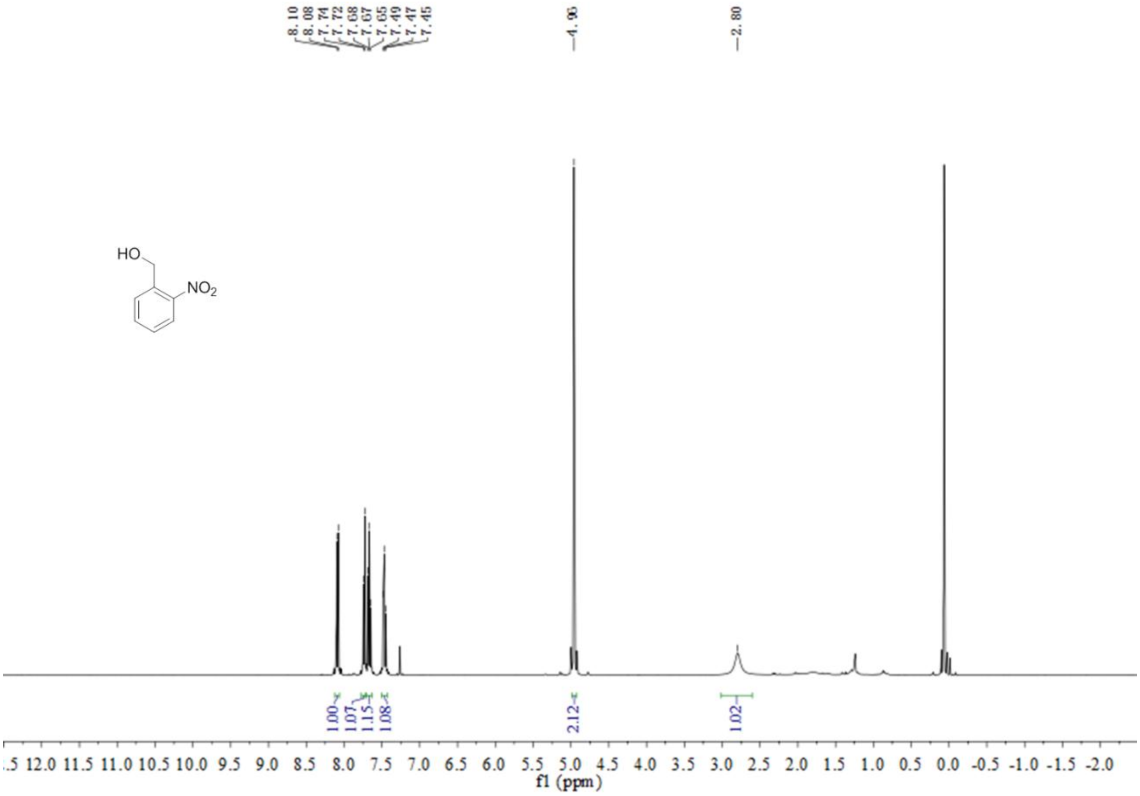

Figure S31. ^{1}H NMR spectrum for compound (2-Nitrophenyl)methanol(**2b**).

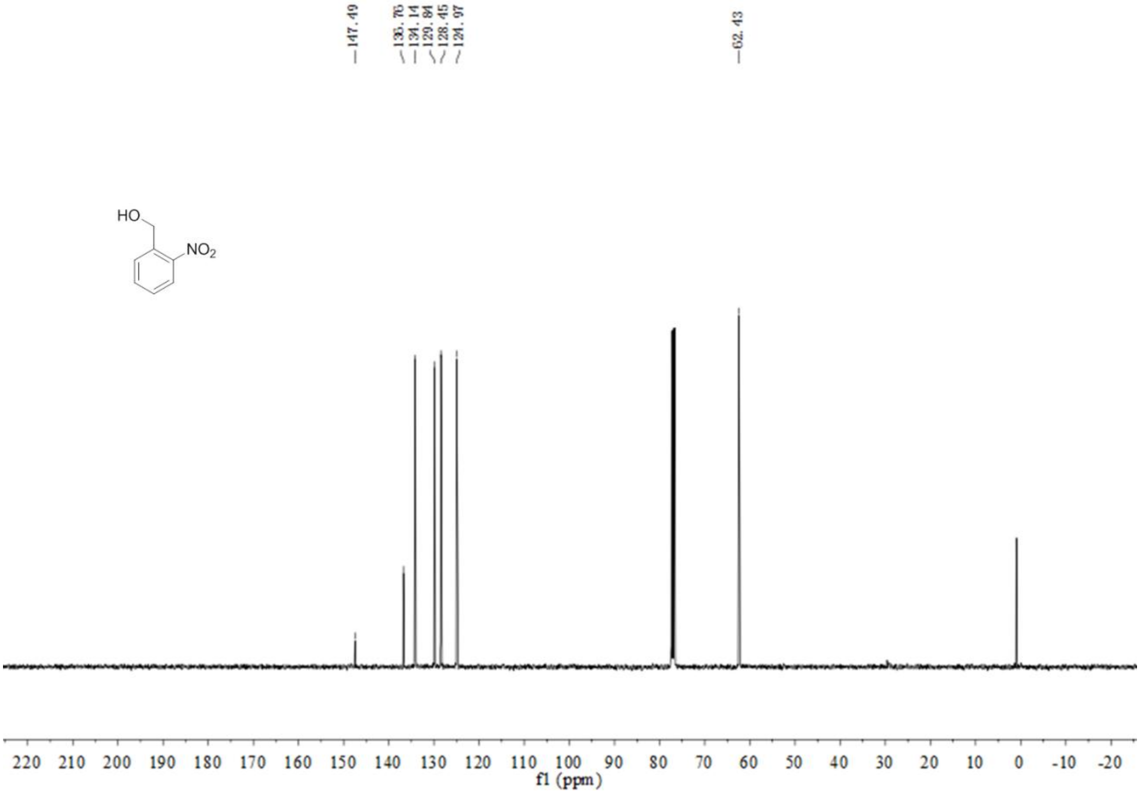

Figure S32. ^{13}C NMR spectrum for compound
(2-Nitrophenyl)methanol(**2b**)

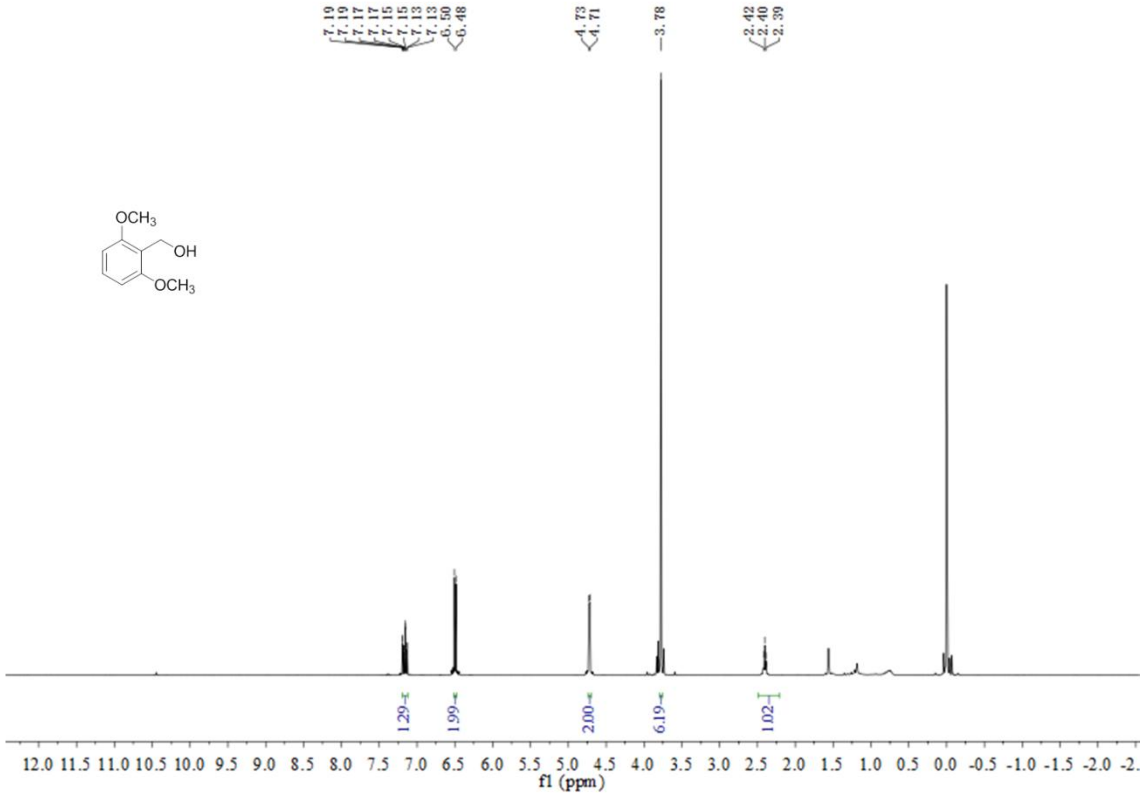

Figure S33. ^{1}H NMR spectrum for compound (2,6-Dimethoxyphenyl)methanol(**2c**).

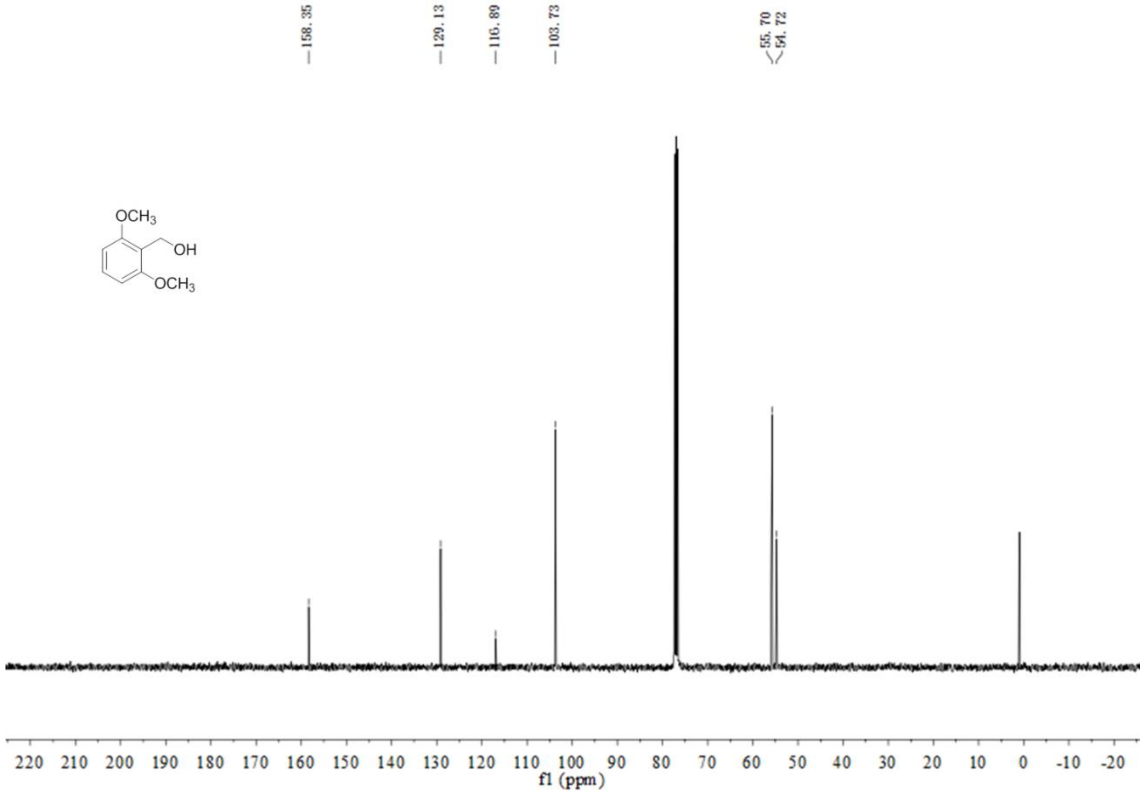

Figure S34. ^{13}C NMR spectrum for compound (2,6-Dimethoxyphenyl)methanol(**2c**).

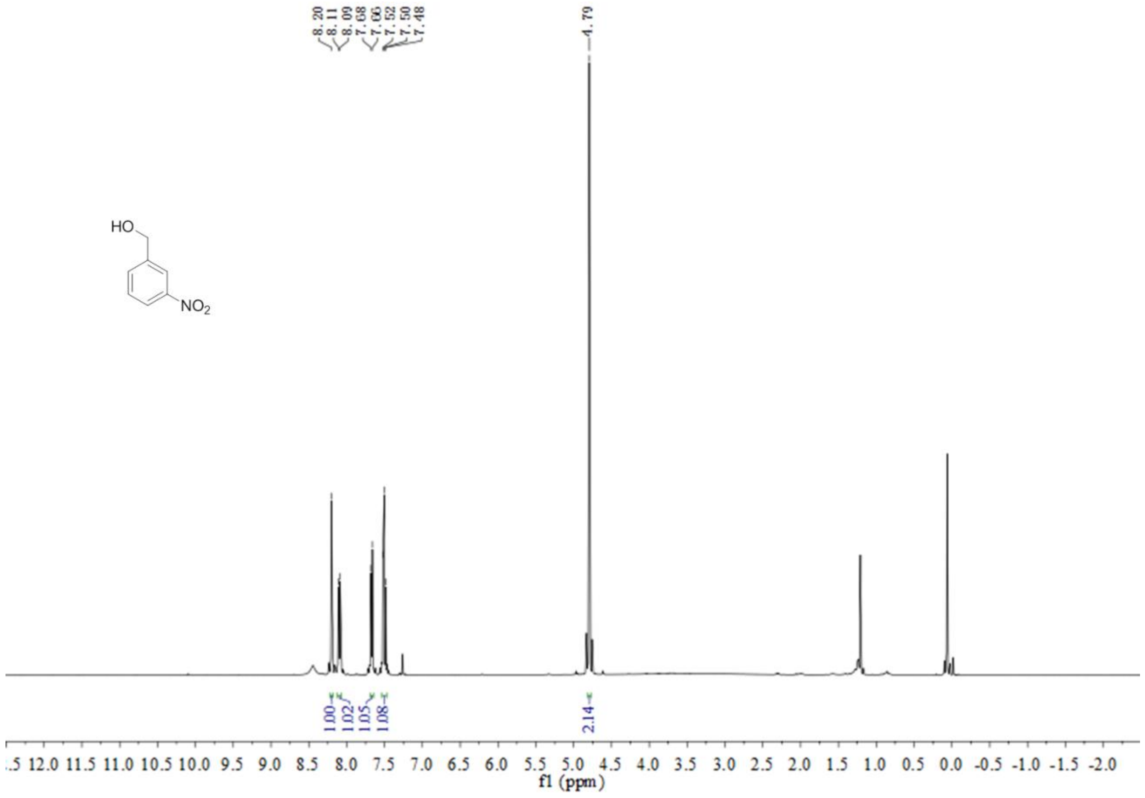

Figure S35. ^{1}H NMR spectrum for compound (3-Nitrophenyl)methanol(**2d**).

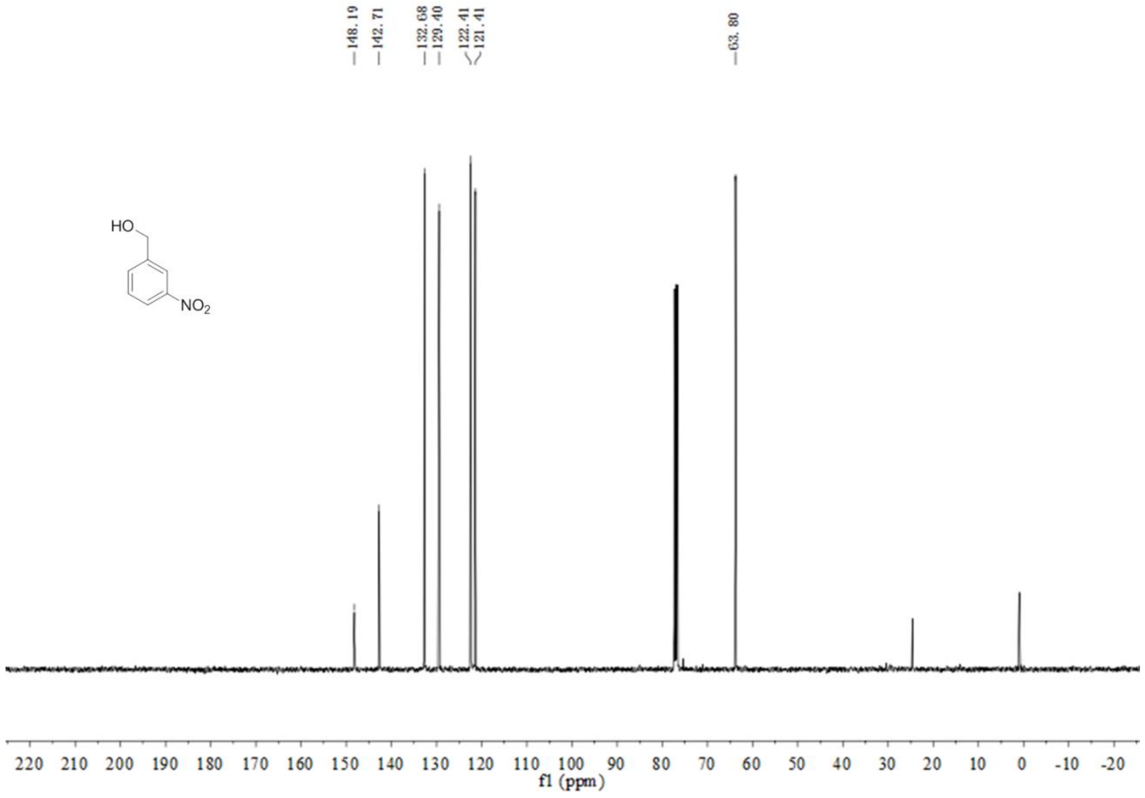

Figure S36. ^{13}C NMR spectrum for compound (3-Nitrophenyl)methanol(**2d**).

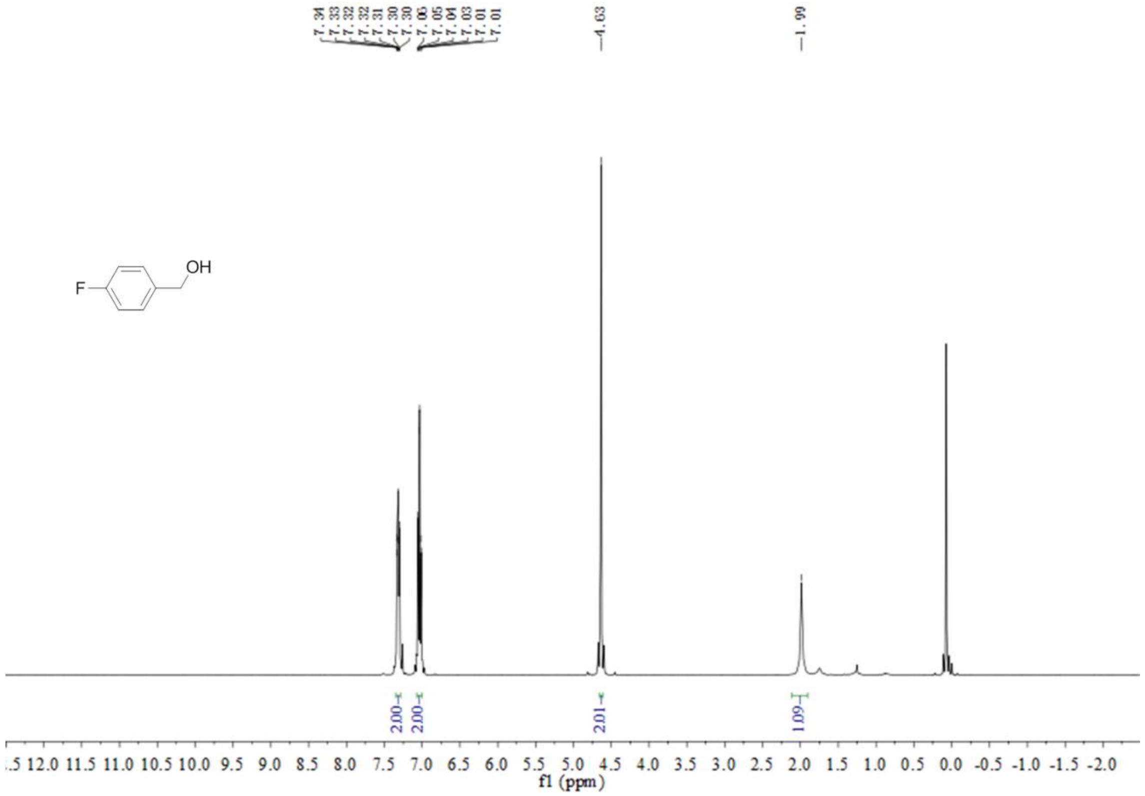

Figure S37. ^{1}H NMR spectrum for compound (4-Fluorophenyl)methanol(**2e**).

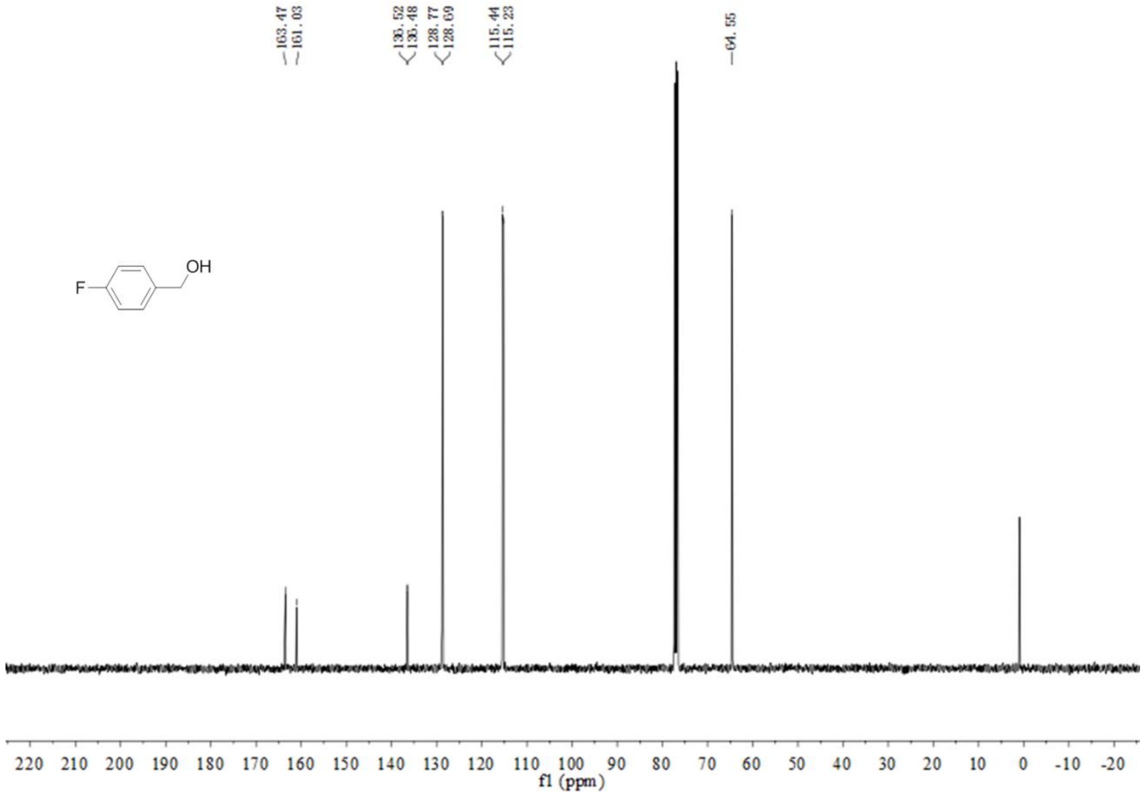

Figure S38. ^{13}C NMR spectrum for compound (4-Fluorophenyl)methanol(**2e**)

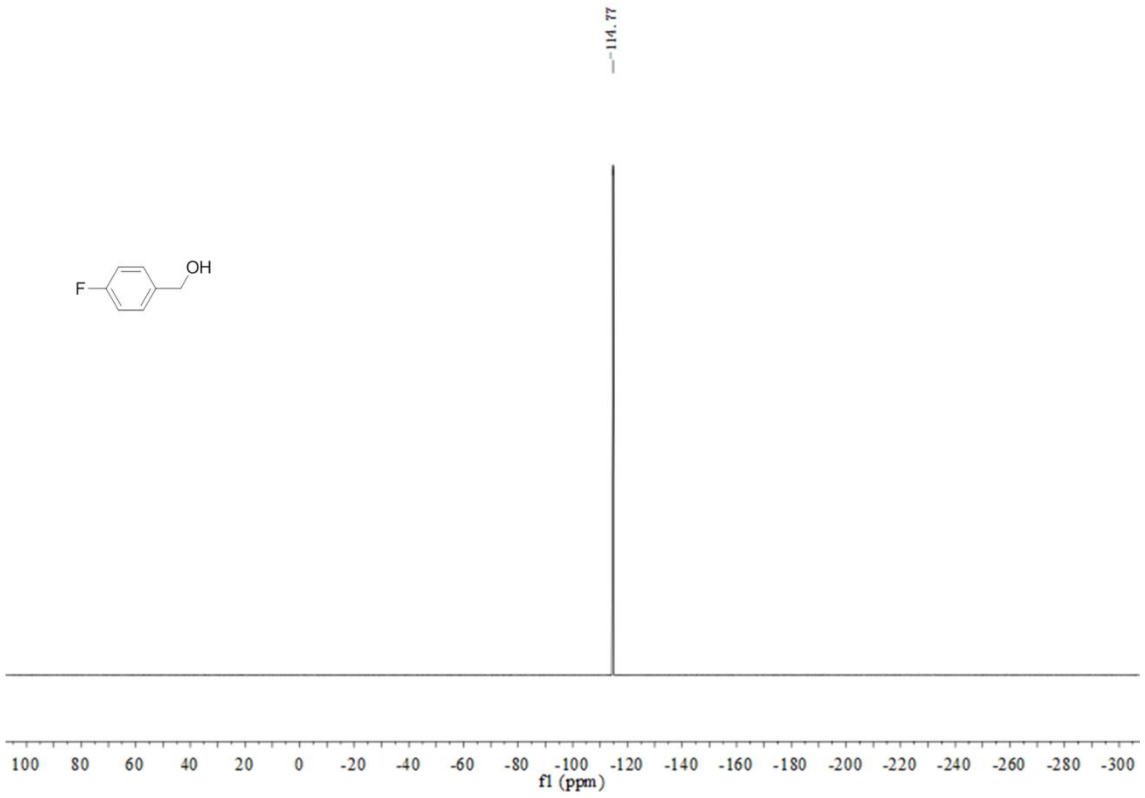

Figure S39. ^{19}FNMR spectrum for compound (4-Fluorophenyl)methanol(**2e**).

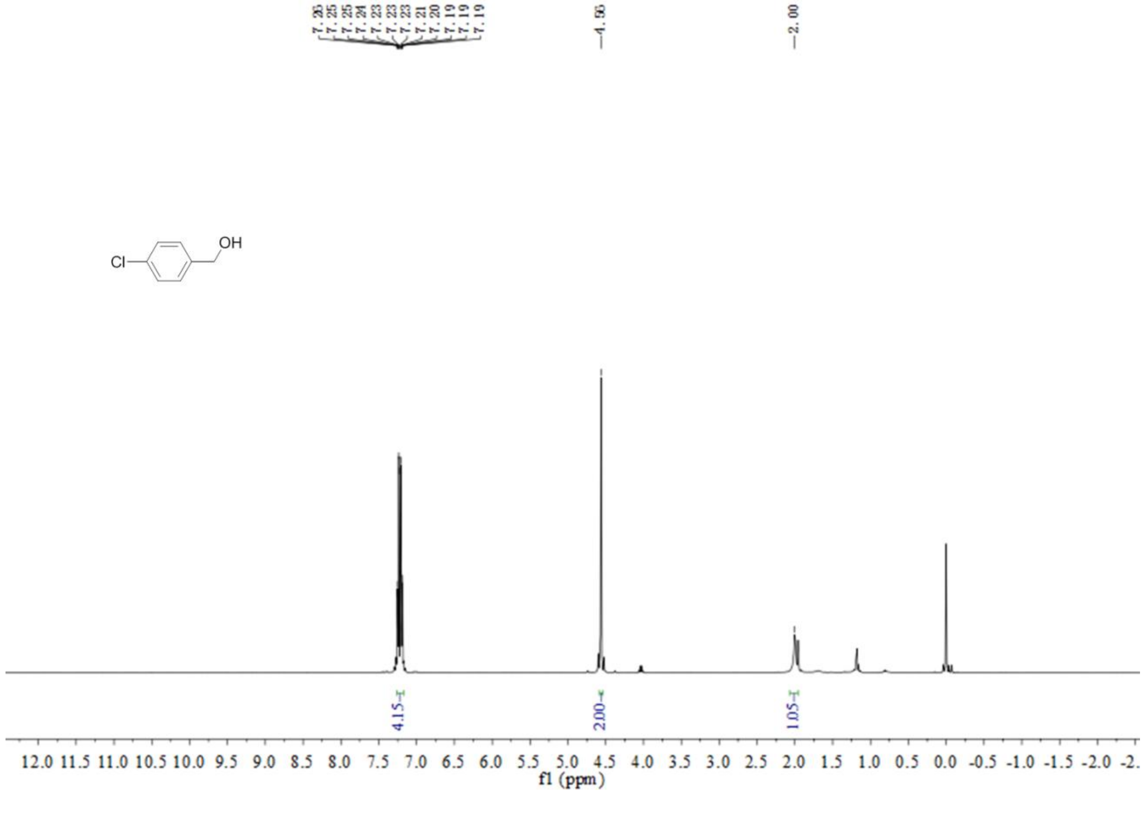

Figure S40. ^{1}H NMR spectrum for compound (4-Chlorophenyl)methanol(**2f**).

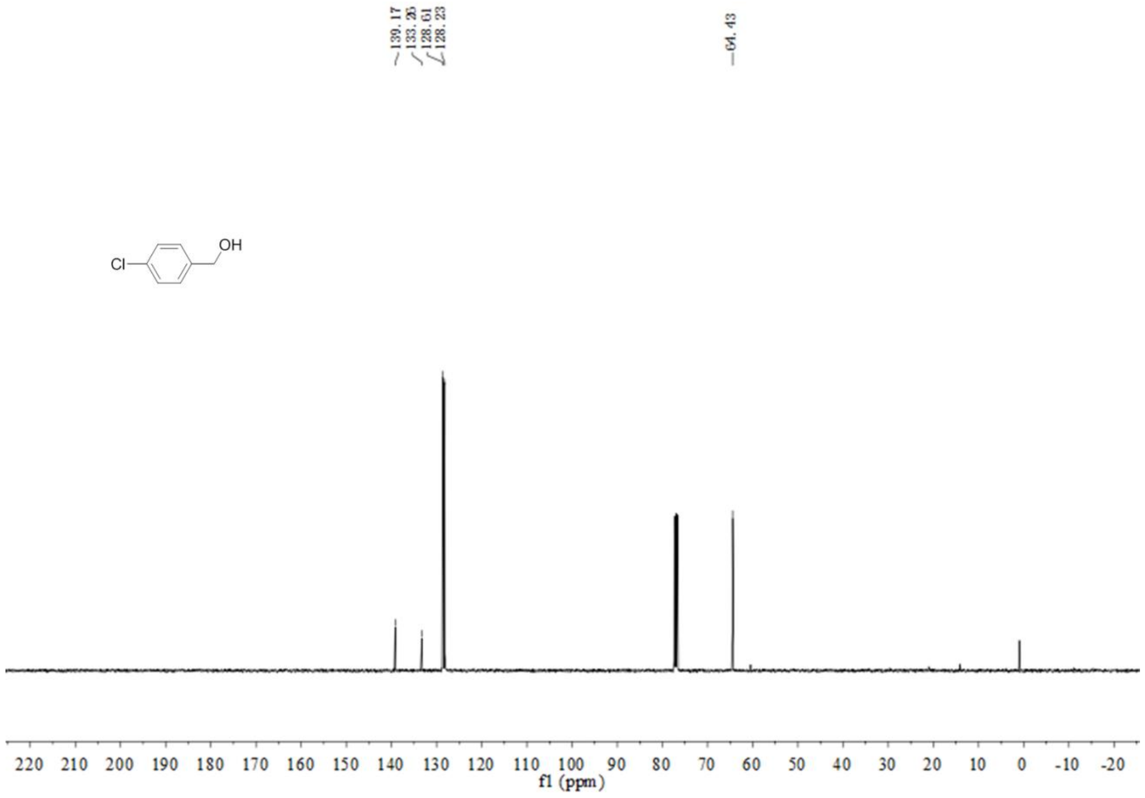

Figure S41. ^{13}C NMR spectrum for compound (4-Chlorophenyl)methanol(**2f**)

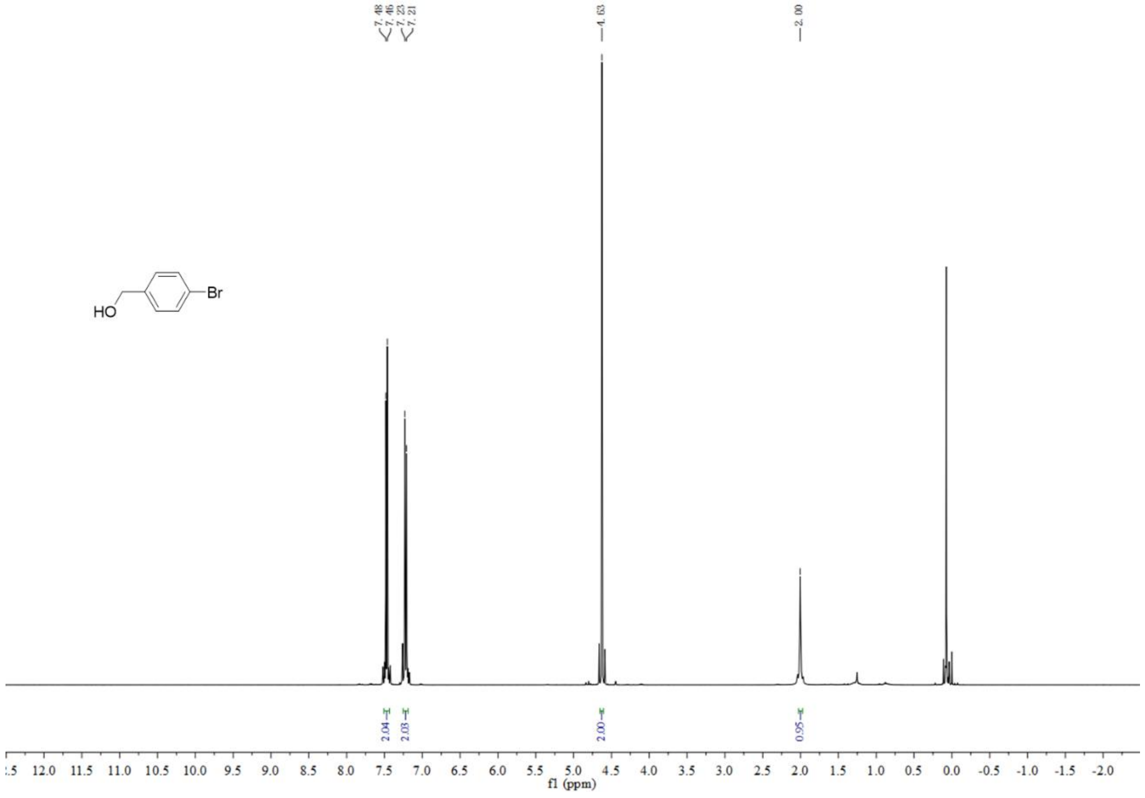

Figure S42. ^{1}H NMR spectrum for compound (4-bromophenyl)methanol (**4g**).

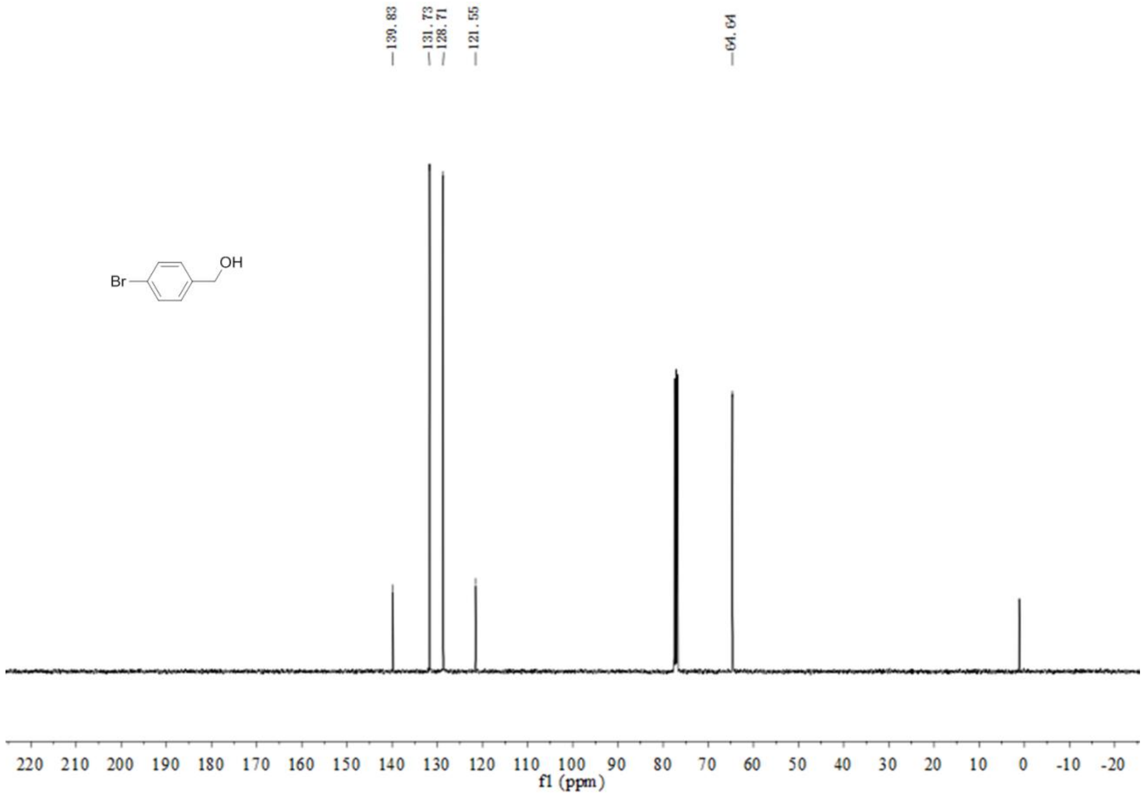

Figure S43. ^{13}C NMR spectrum for compound(4-bromophenyl)methanol (**4g**)

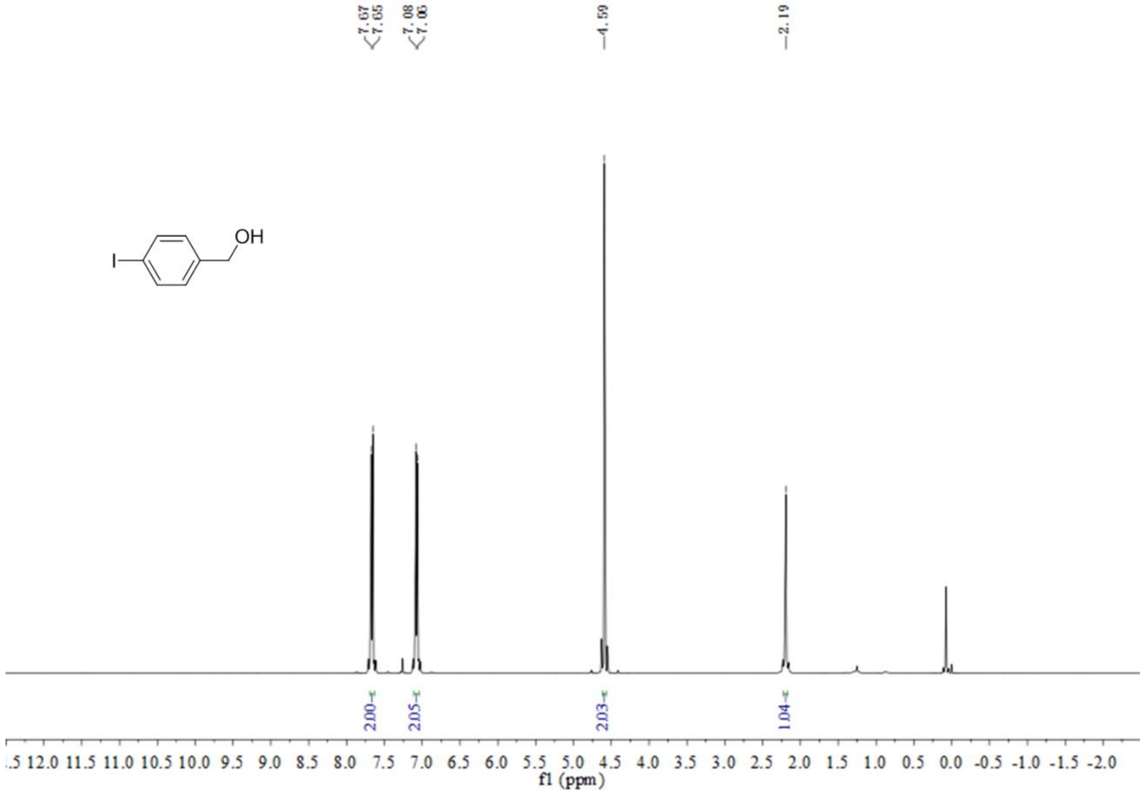

Figure S44. ^{1}H NMR spectrum for compound (4-Iodophenyl)methanol(**2h**)

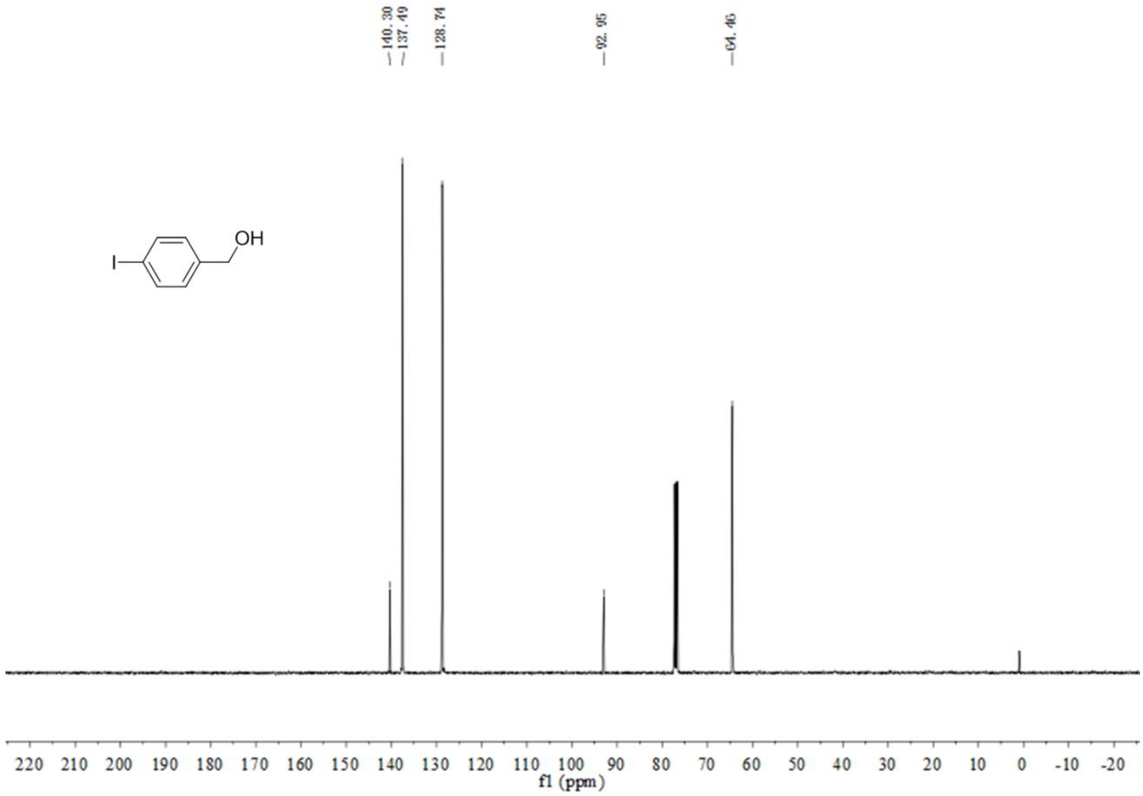

Figure S45. ^{13}C NMR spectrum for compound (4-Iodophenyl)methanol(**2h**)

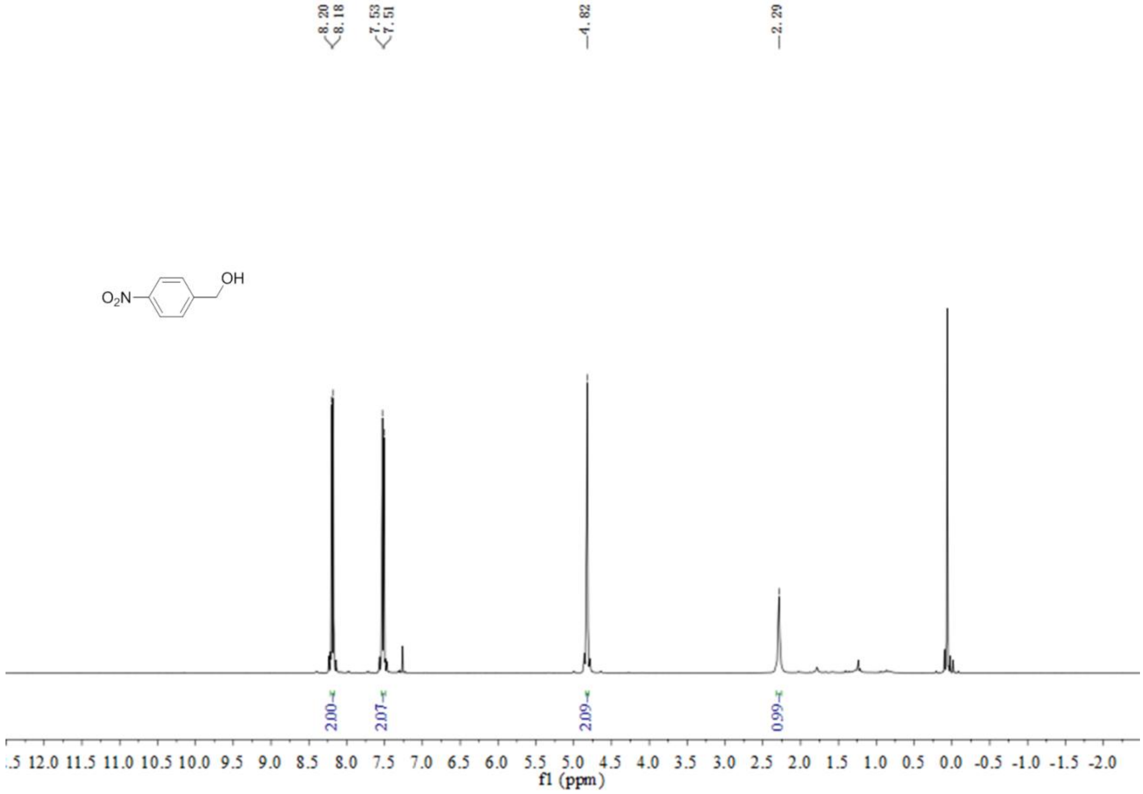

Figure S46. ^{1}H NMR spectrum for compound (4-Nitrophenyl)methanol(**2i**).

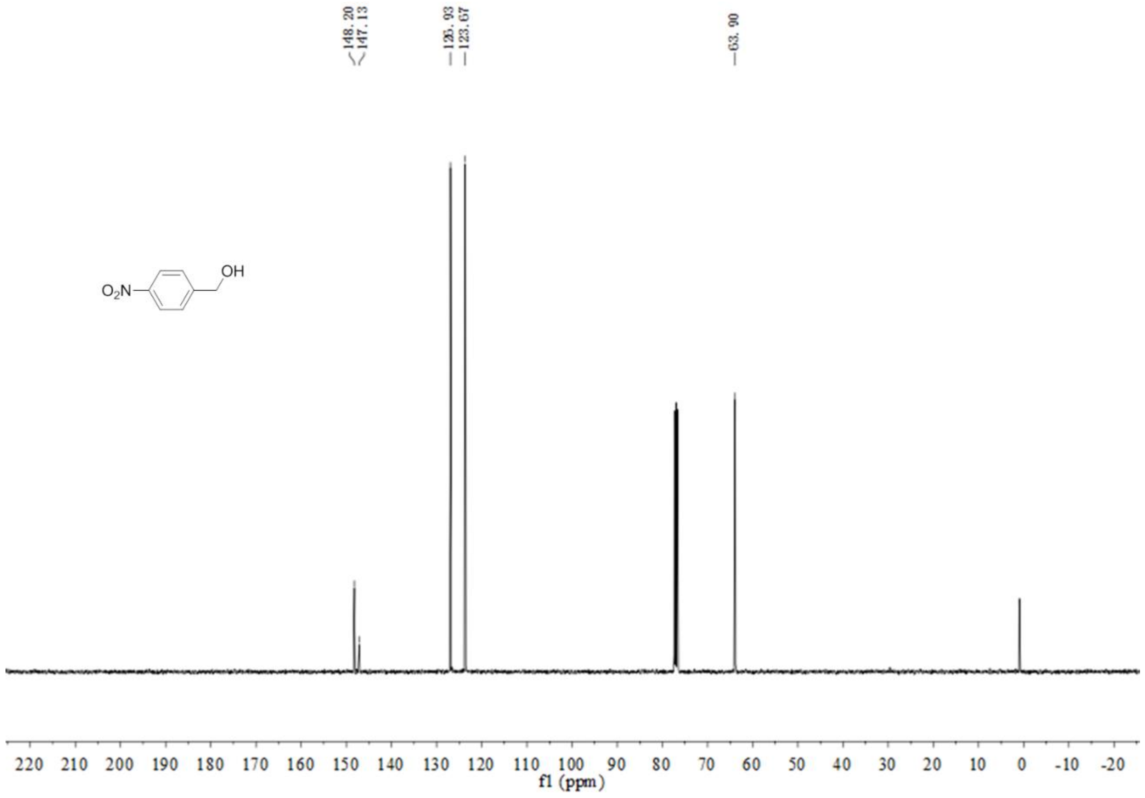

Figure S47. ^{13}C NMR spectrum for compound (4-Nitrophenyl)methanol(**2i**).

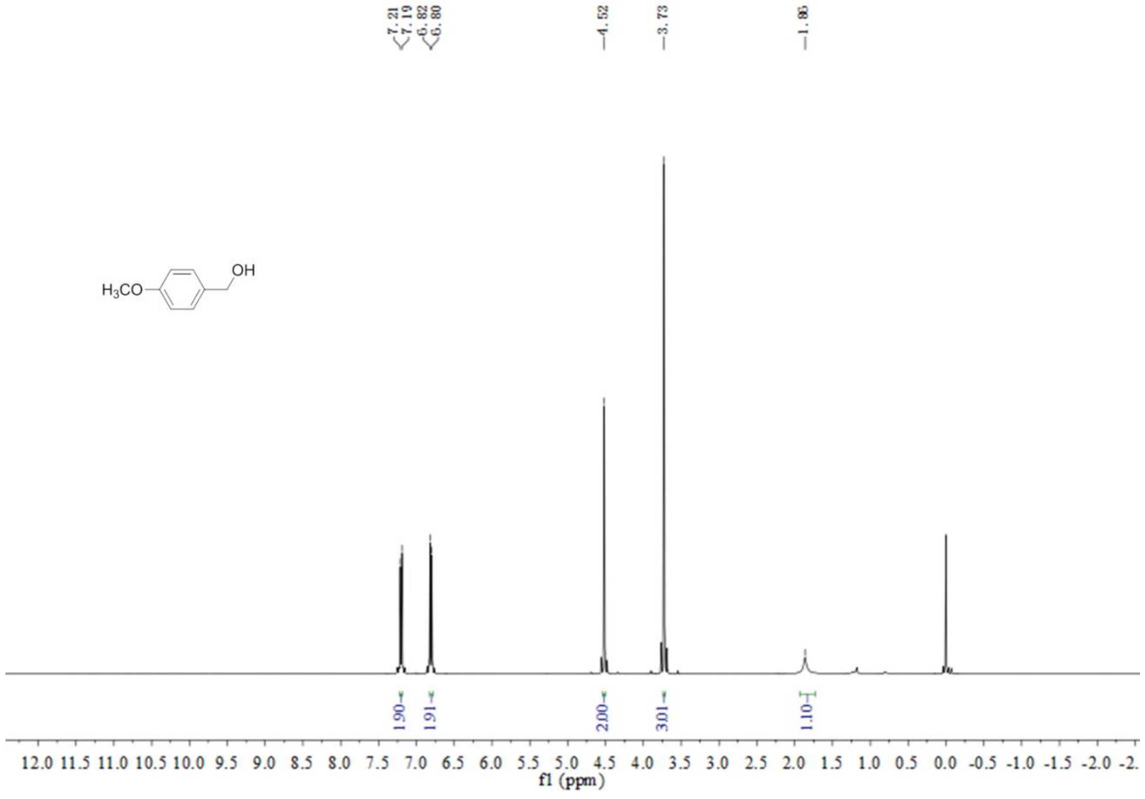

Figure S48. ^{1}H NMR spectrum for compound (4-Methoxyphenyl)methanol(**2j**).

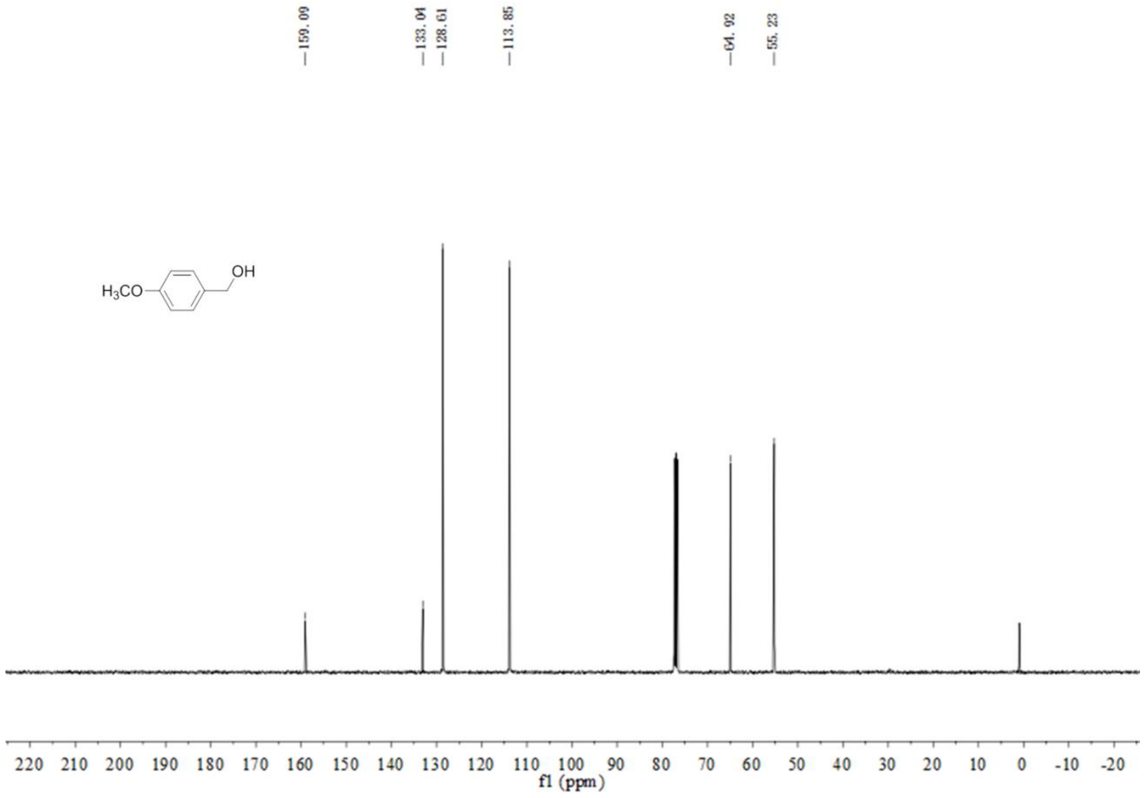

Figure S49. ^{13}C NMR spectrum for compound (4-Methoxyphenyl)methanol(**2j**).

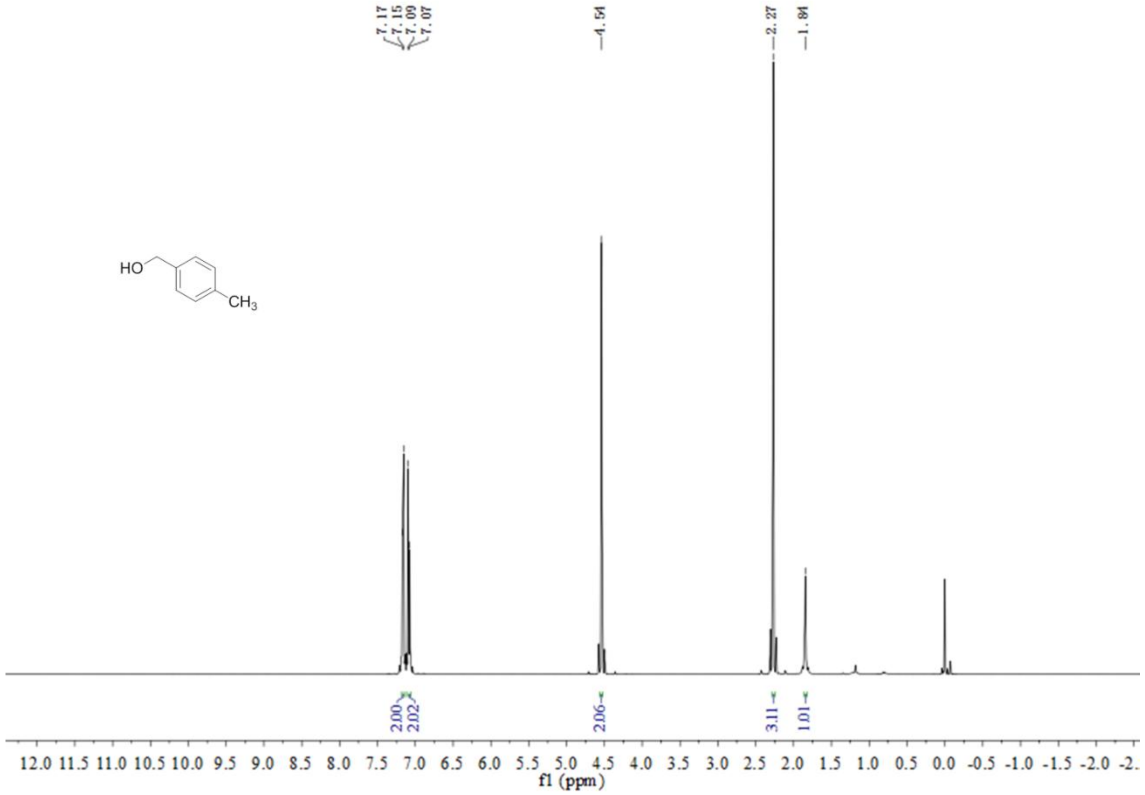

Figure S50. ^{1}H NMR spectrum for compound p-Tolylmethanol(**2k**).

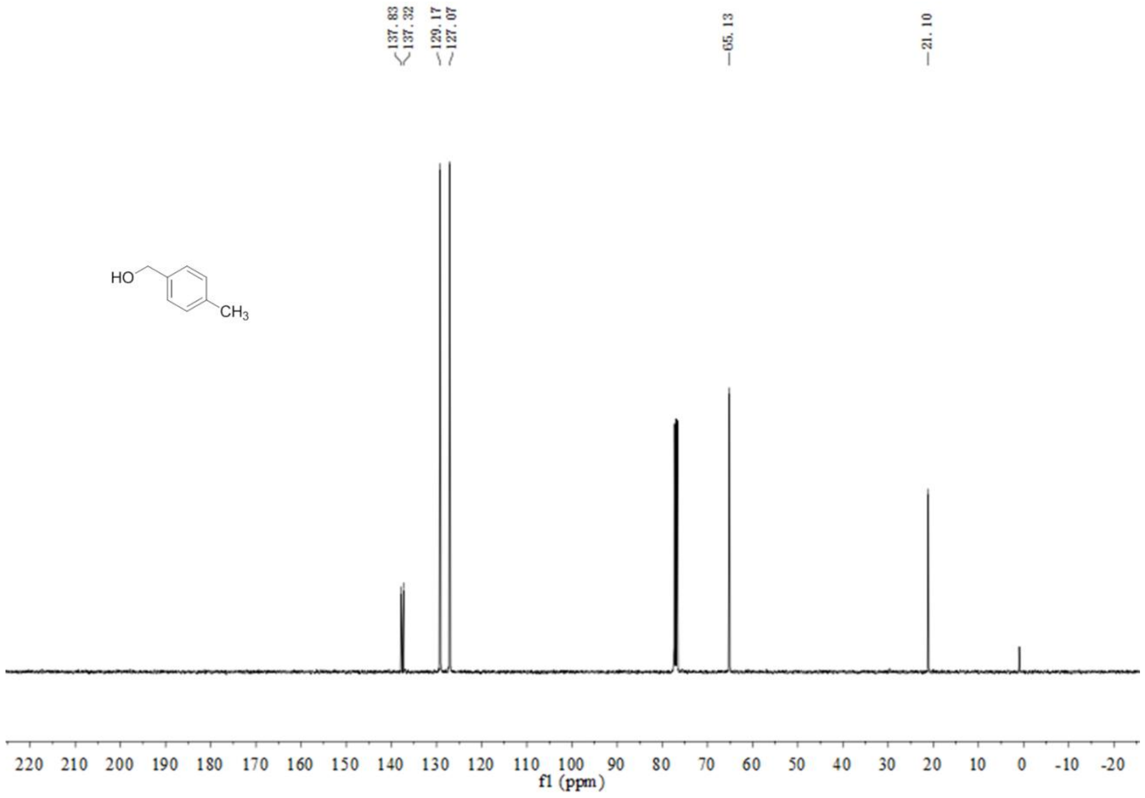

Figure S51. ^{13}C NMR spectrum for compound p-Tolylmethanol(**2k**).

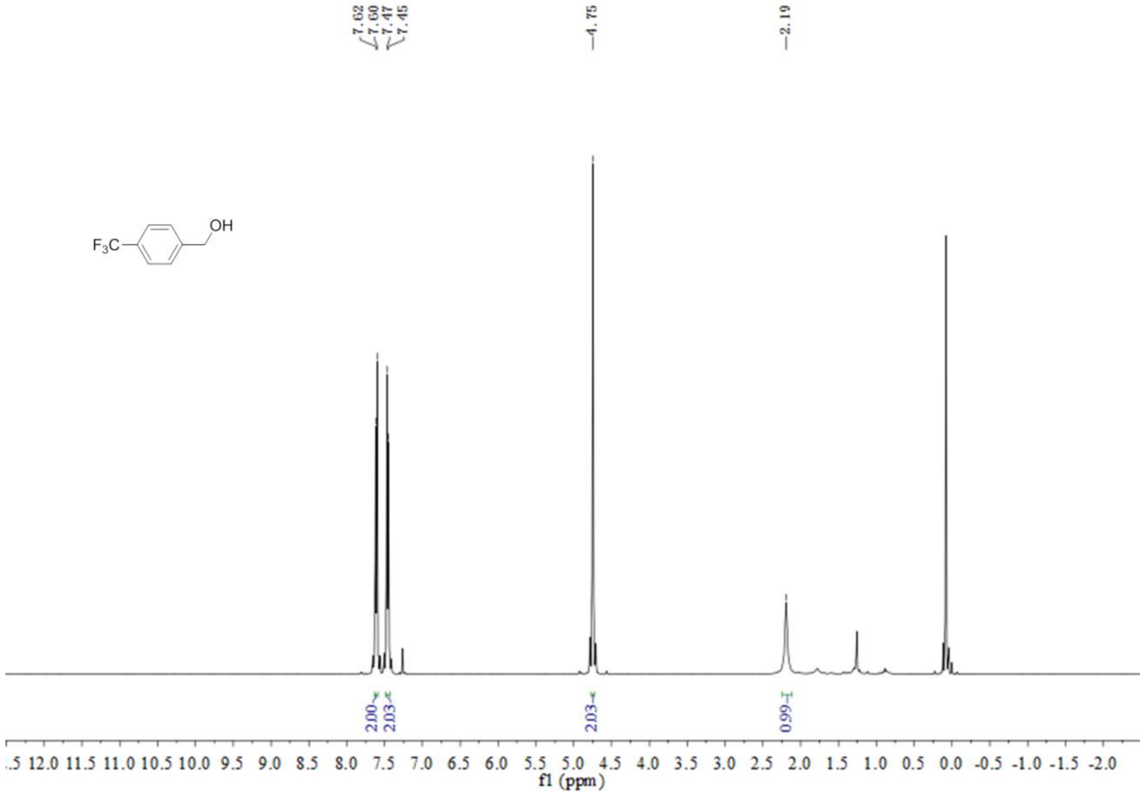

Figure S52. ^{1}H NMR spectrum for compound (4-(Trifluoromethyl)phenyl)methanol(**2l**).

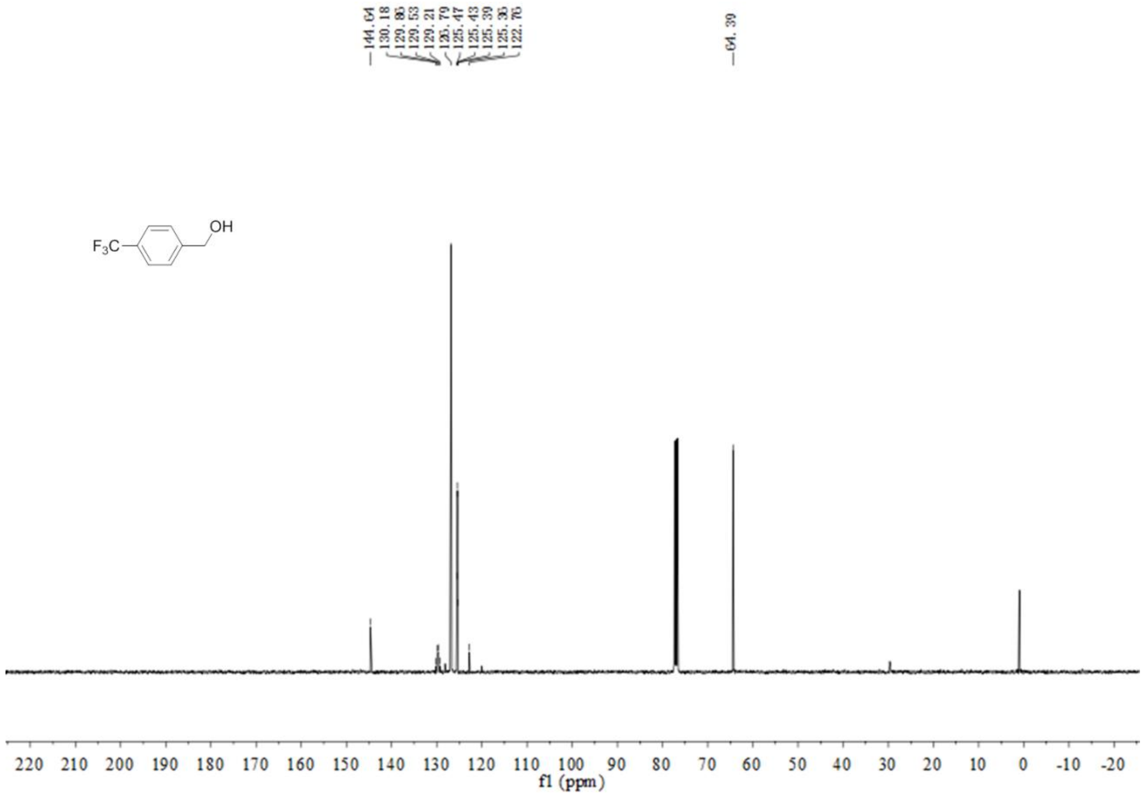

Figure S53. ^{13}C NMR spectrum for compound (4-(Trifluoromethyl)phenyl)methanol(**2l**).

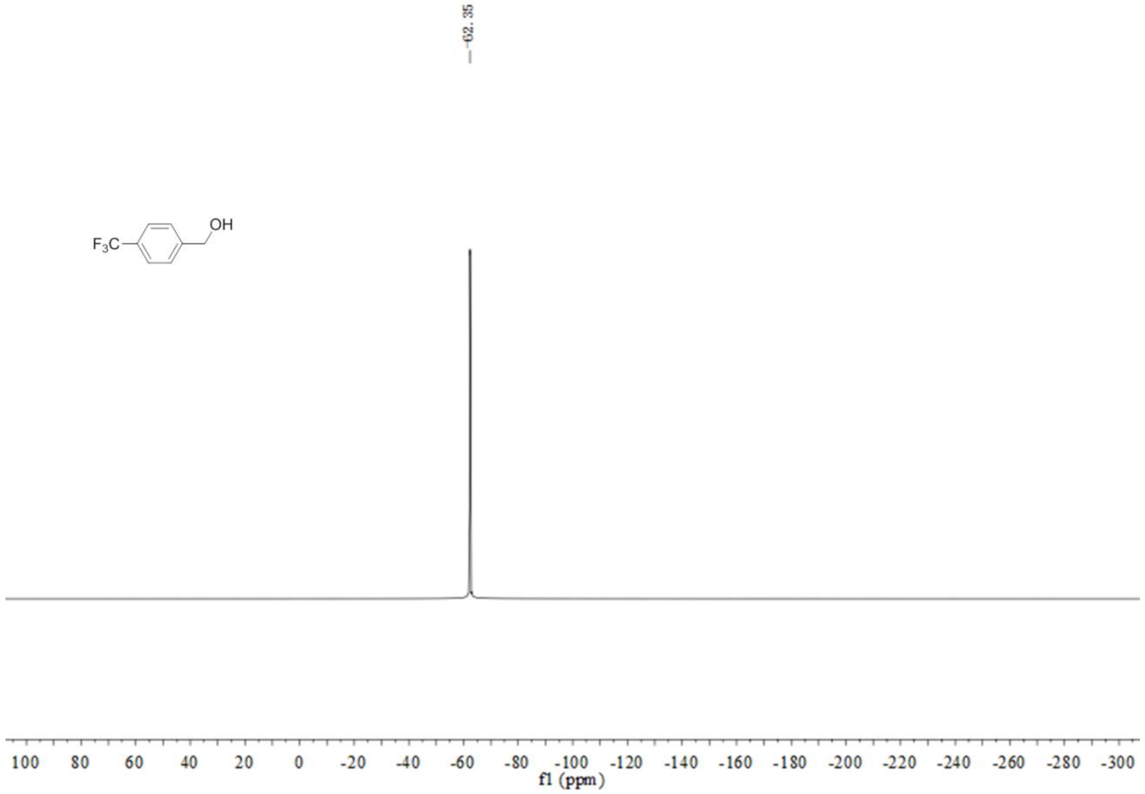

Figure S54. ^{19}F NMR spectrum for compound (4-(Trifluoromethyl)phenyl)methanol(**2l**).

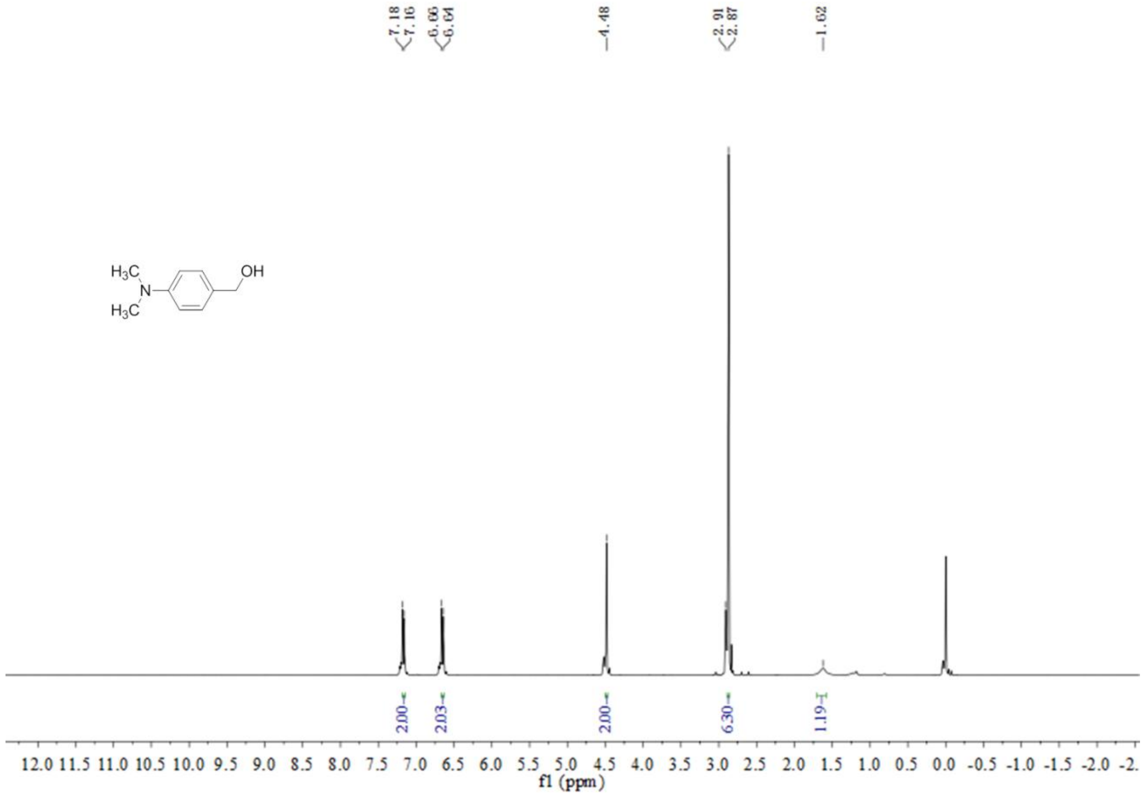

Figure S55. ^{1}H NMR spectrum for compound (4-(Dimethylamino)phenyl)methanol(**2m**).

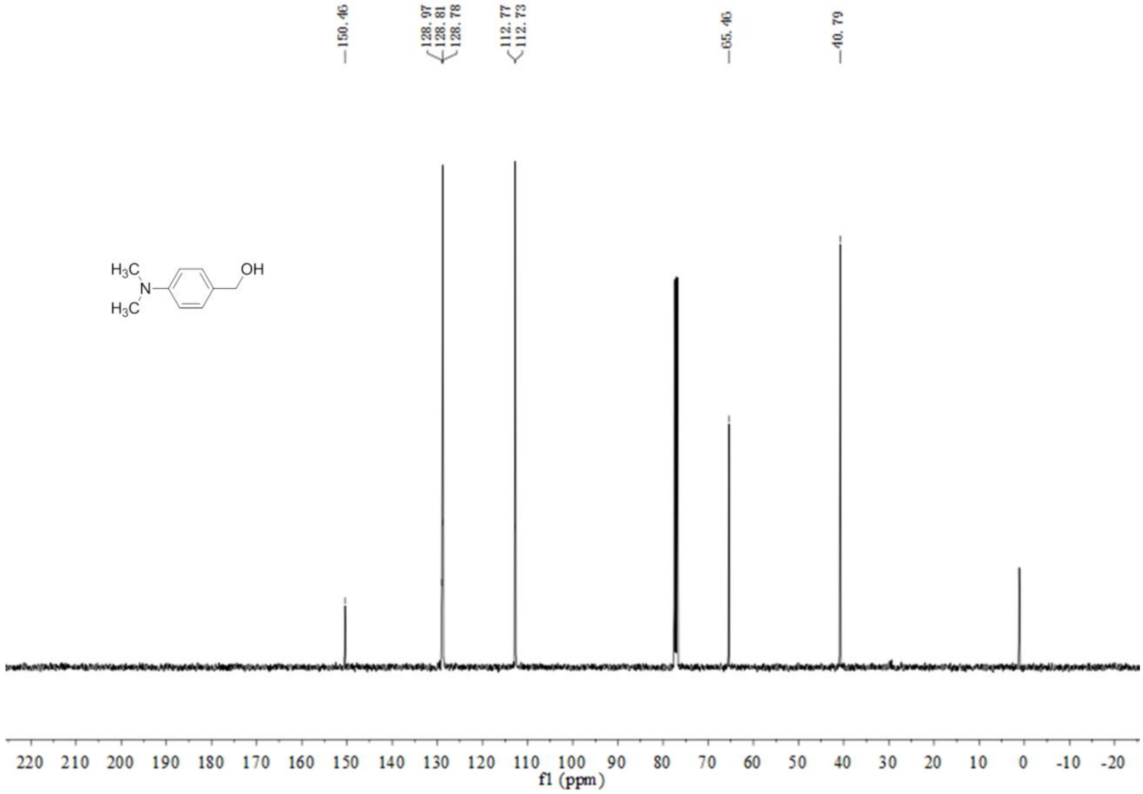

Figure S56. ^{13}C NMR spectrum for compound (4-(Dimethylamino)phenyl)methanol(**2m**).

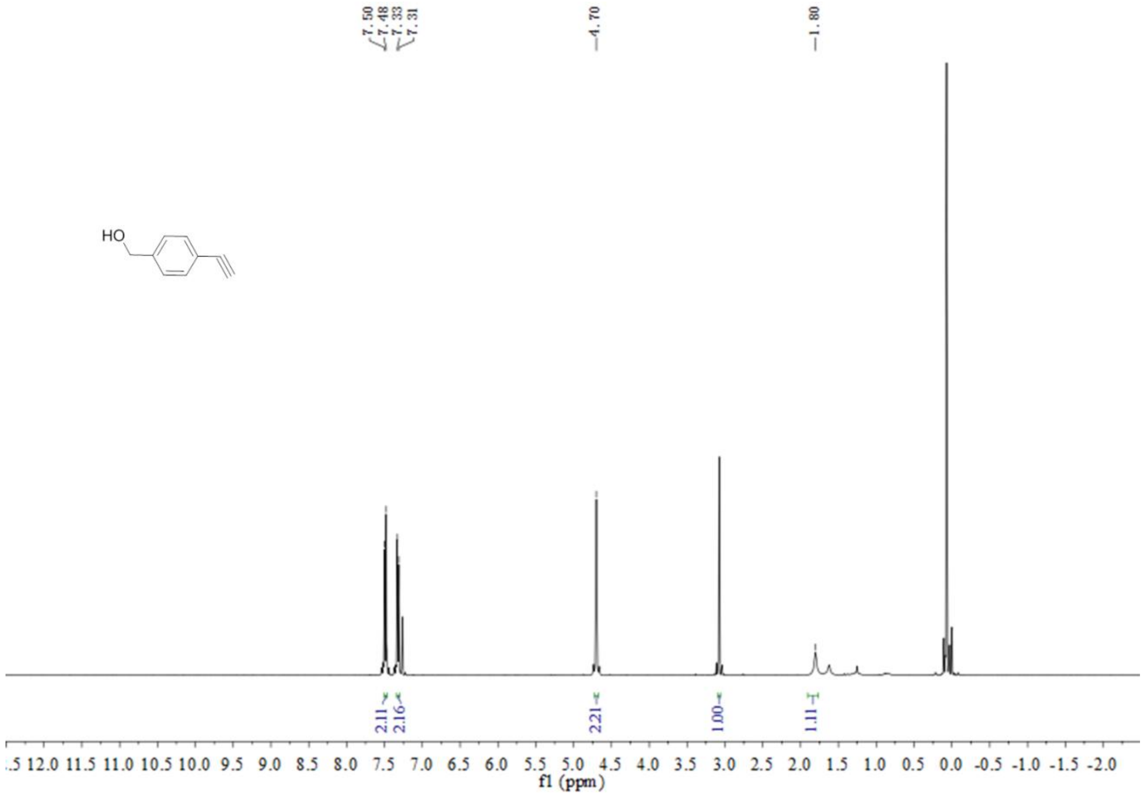

Figure S57. ^{1}H NMR spectrum for compound (4-Ethynylphenyl)methanol(**2n**).

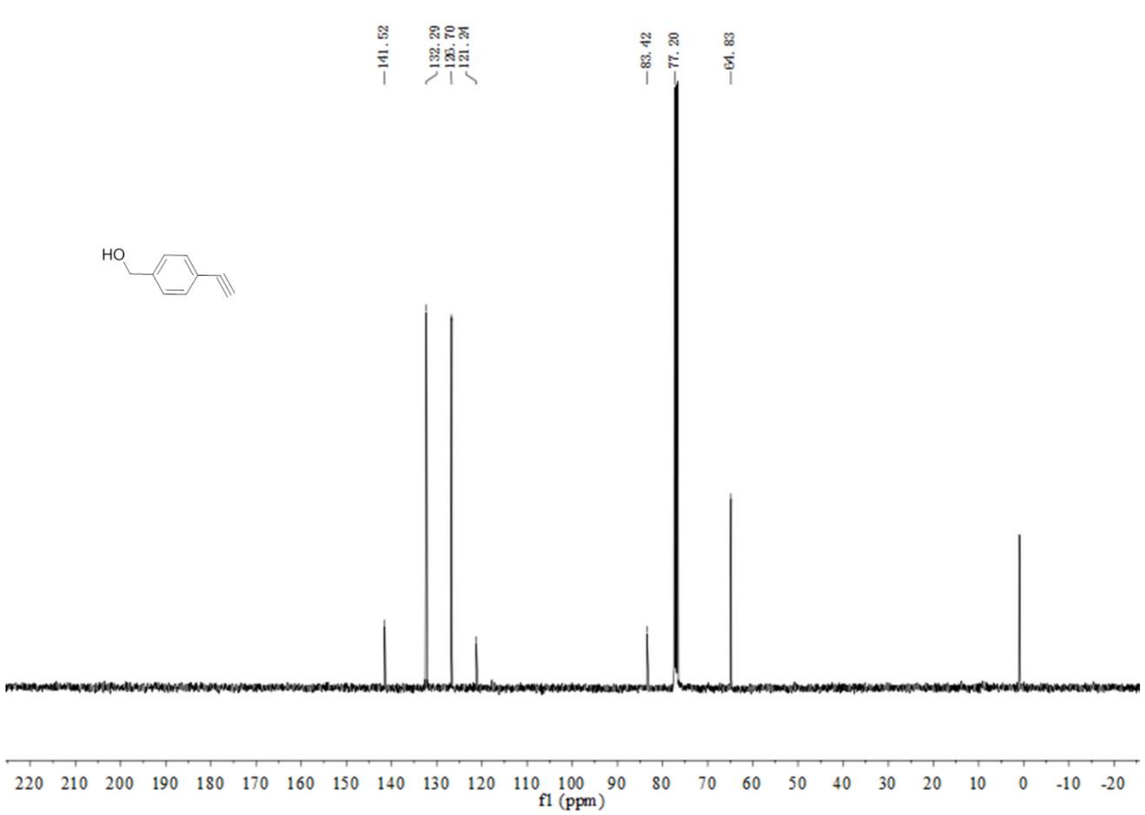

Figure S58. ^{13}C NMR spectrum for compound (4-Ethynylphenyl)methanol(**2n**).

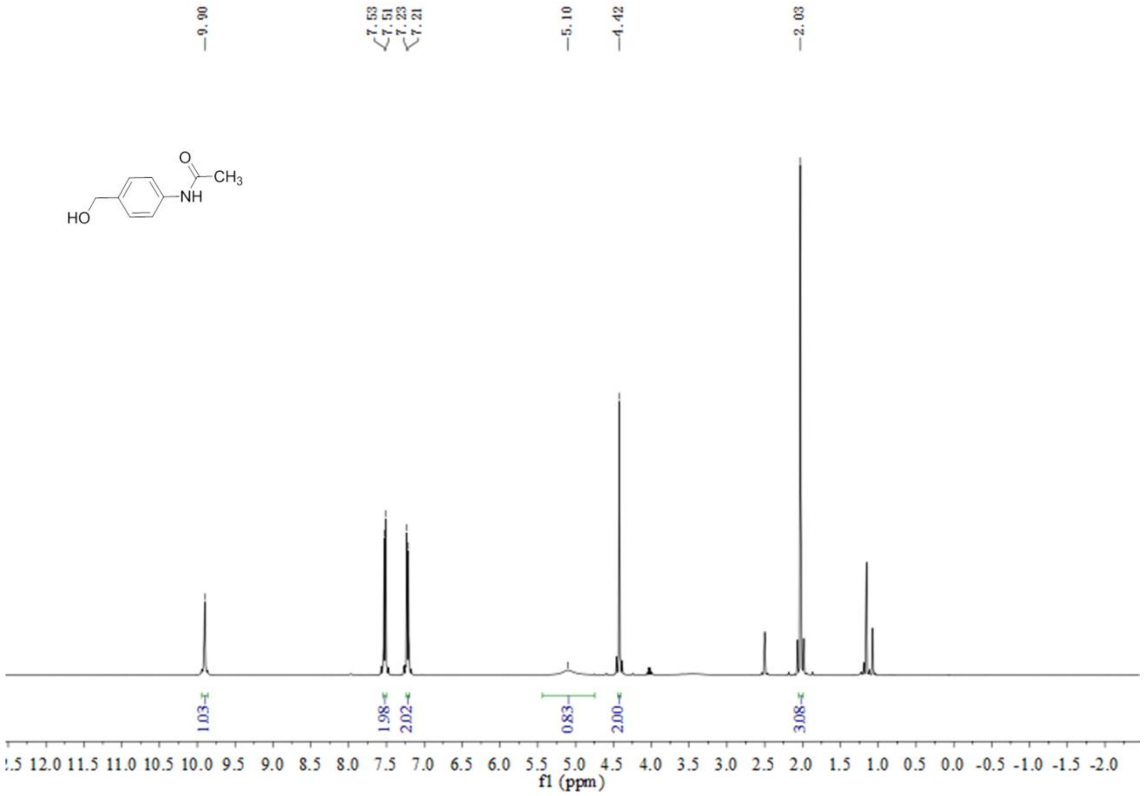

Figure S59. ^{1}H NMR spectrum for compound N-(4-(hydroxymethyl)phenyl)acetamide(**2o**).

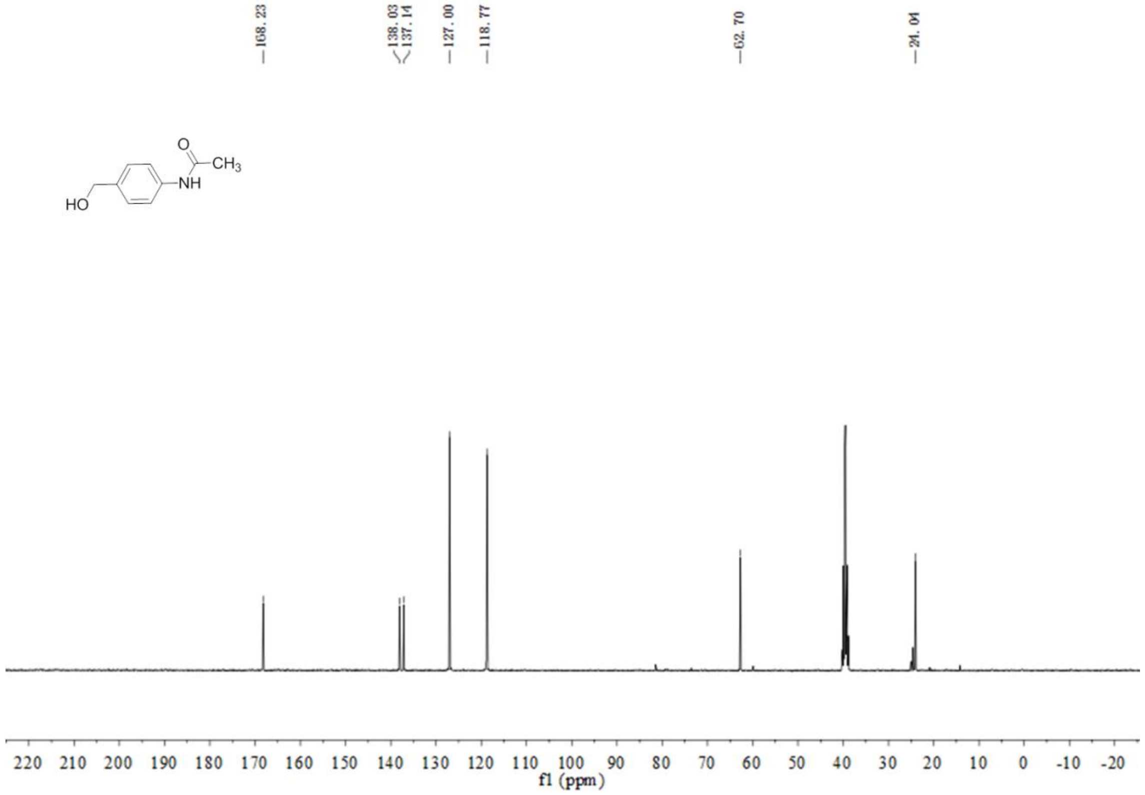

Figure S60. ^{13}C NMR spectrum for compound N-(4-(hydroxymethyl)phenyl)acetamide(**2o**).

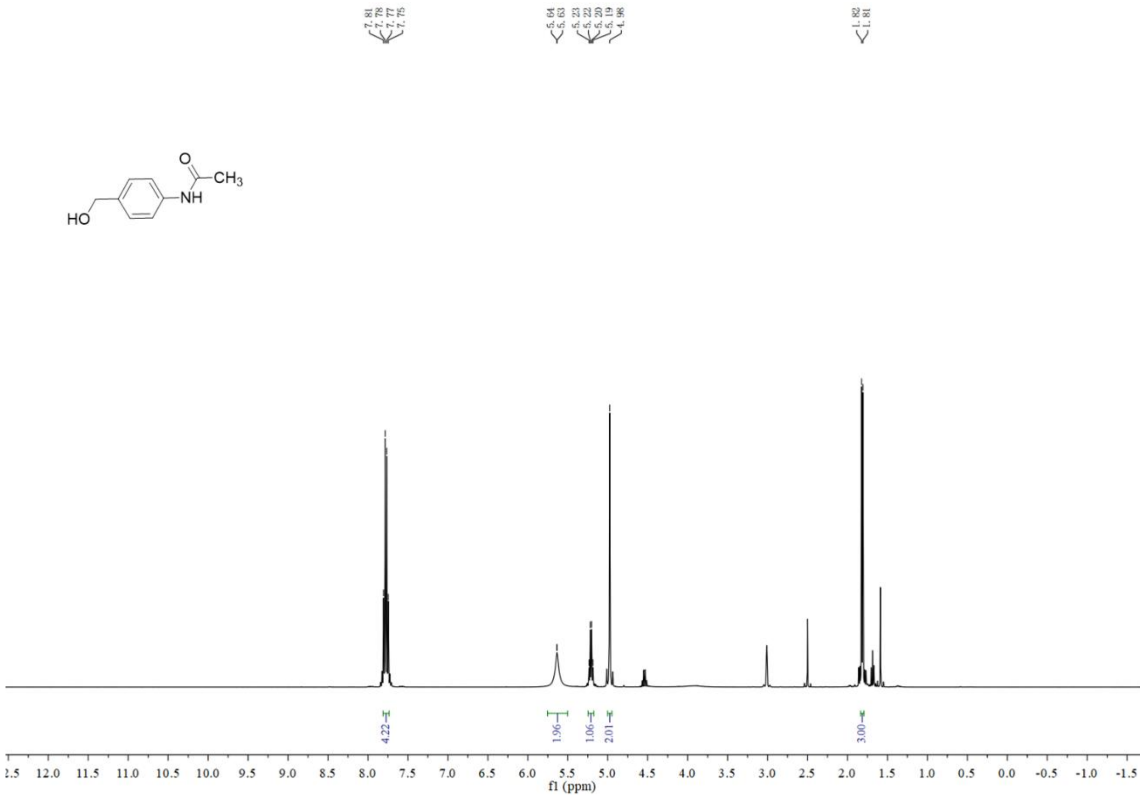

Figure S61. ^{1}H NMR spectrum for compound 1-(4-(Hydroxymethyl)phenyl)ethanol(**2p**)

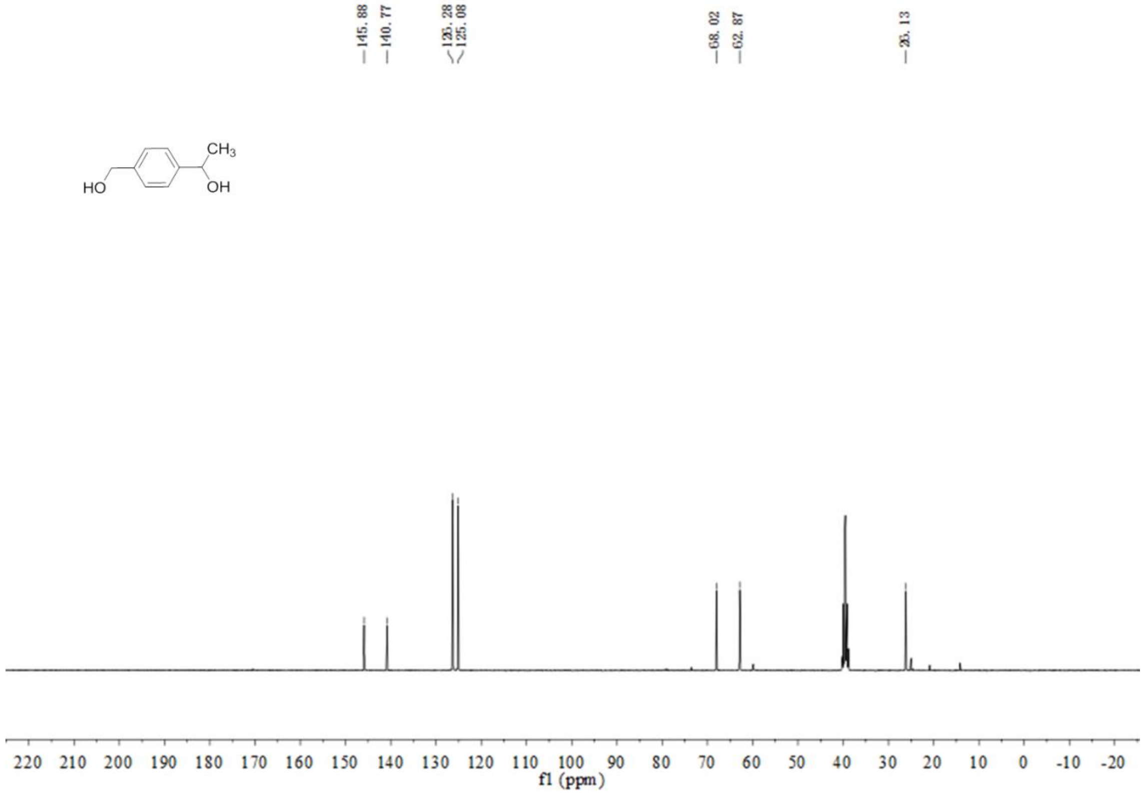

Figure S62. ^{13}C NMR spectrum for compound 1-(4-(Hydroxymethyl)phenyl)ethanol(**2p**).

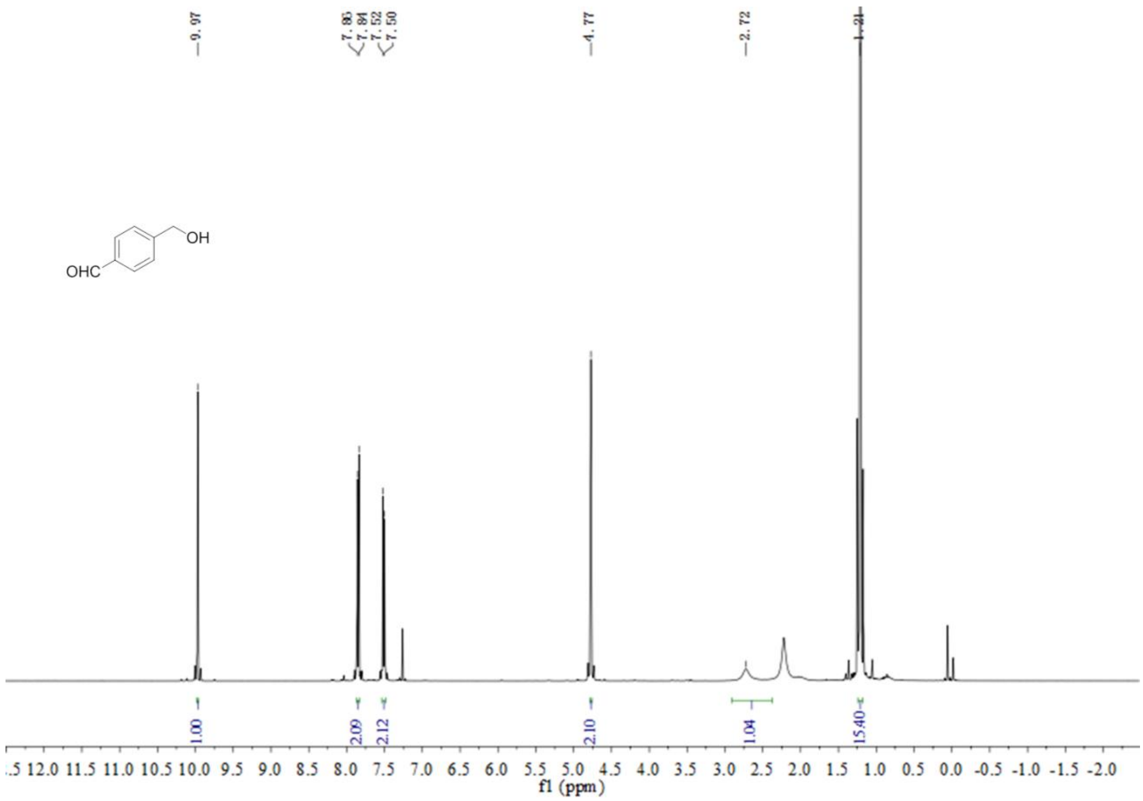

Figure S63. ^{1}H NMR spectrum for compound 4-(Hydroxymethyl)benzaldehyde(**2q**).

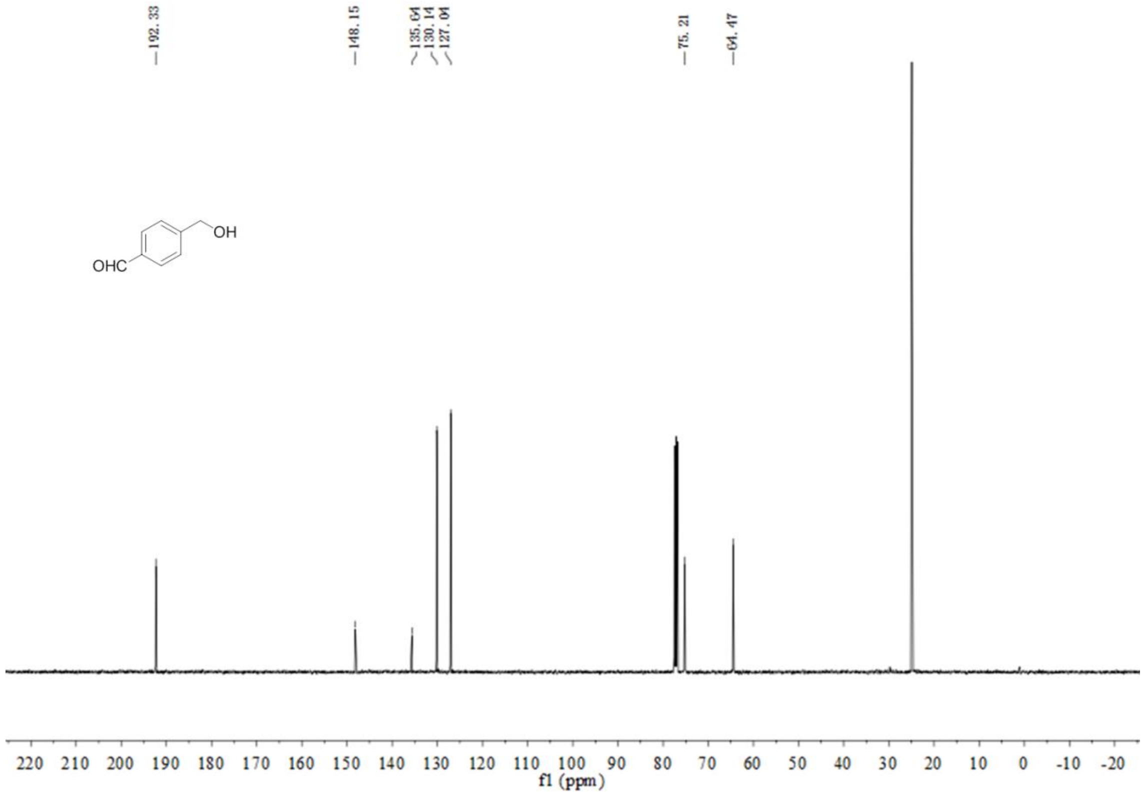

Figure S64. ^{13}C NMR spectrum for compound 4-(Hydroxymethyl)benzaldehyde(**2q**).

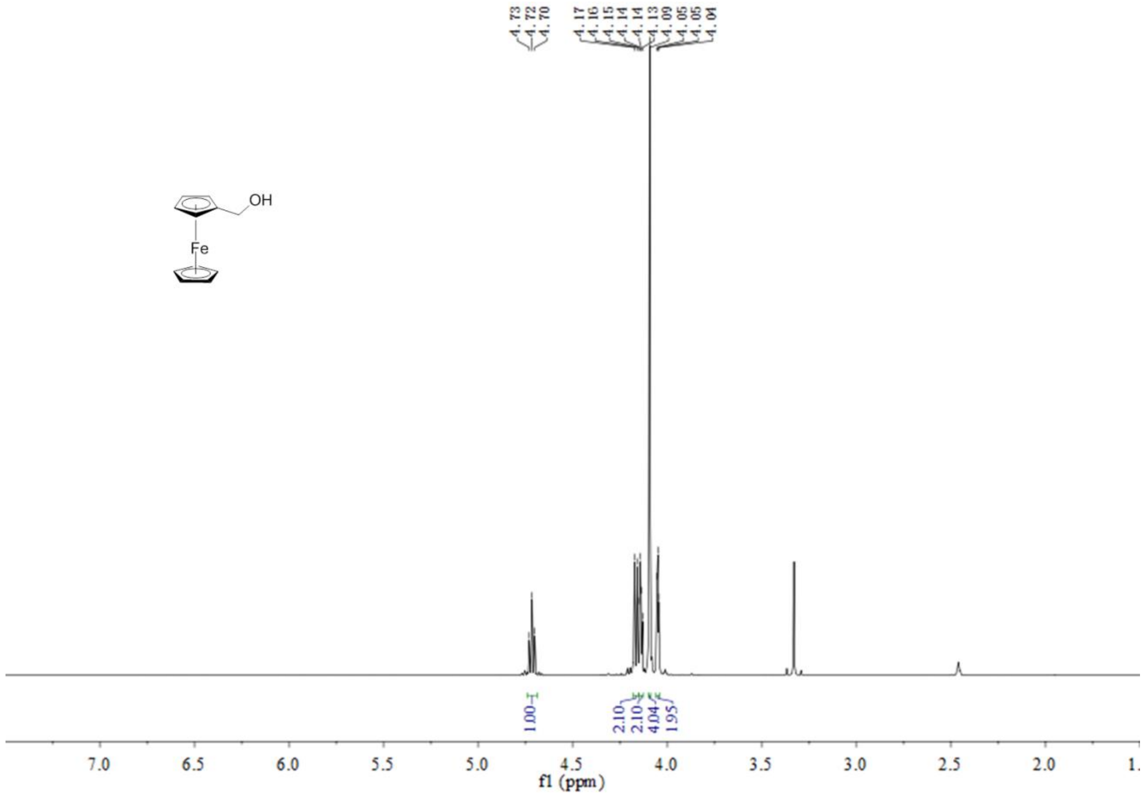

Figure S65. ^{1}H NMR spectrum for compound Ferrocenemethanol(**2r**).

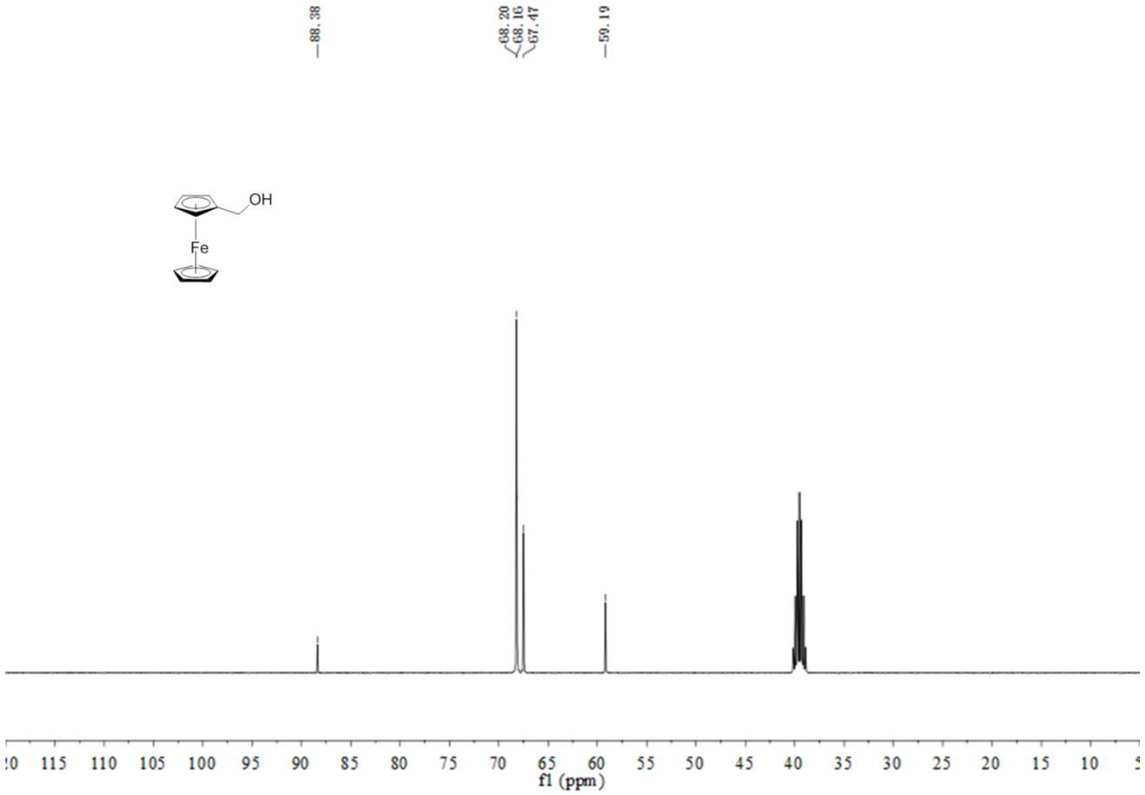

Figure S66. ^{13}C NMR spectrum for compound Ferrocenemethanol(**2r**).

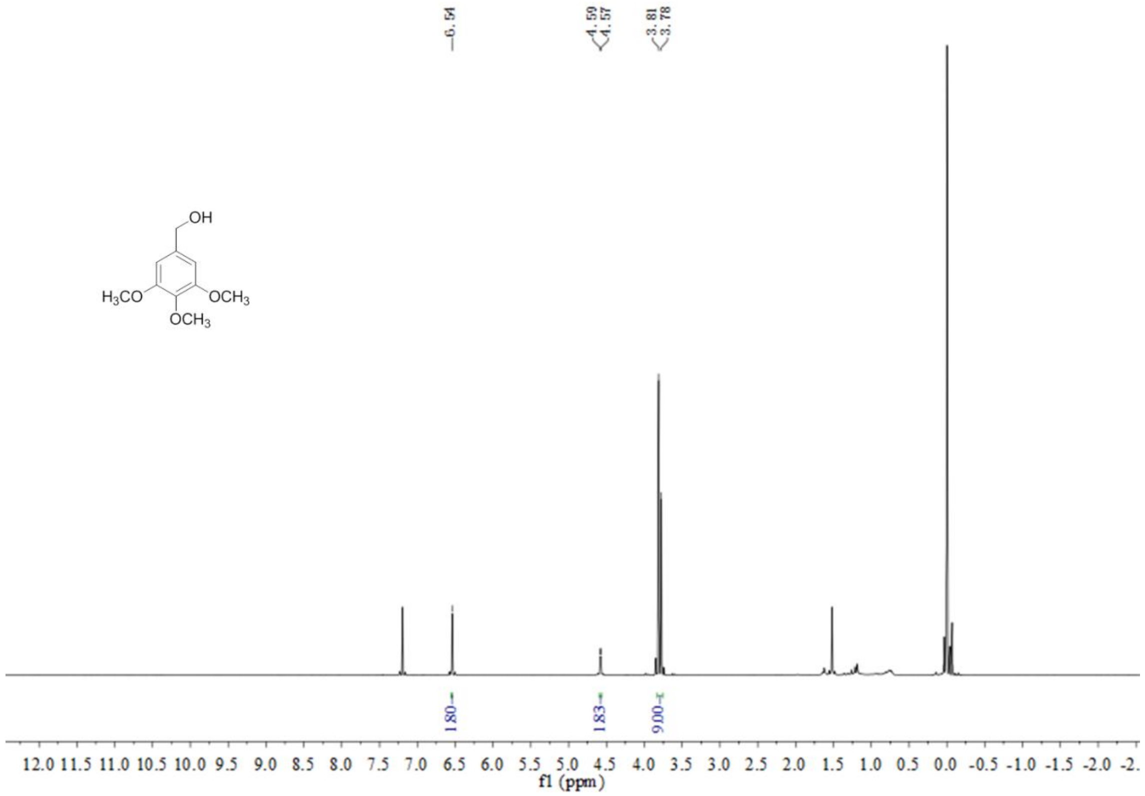

Figure S67. ^{1}H NMR spectrum for compound (3,4,5-Trimethoxyphenyl)methanol(**2s**).

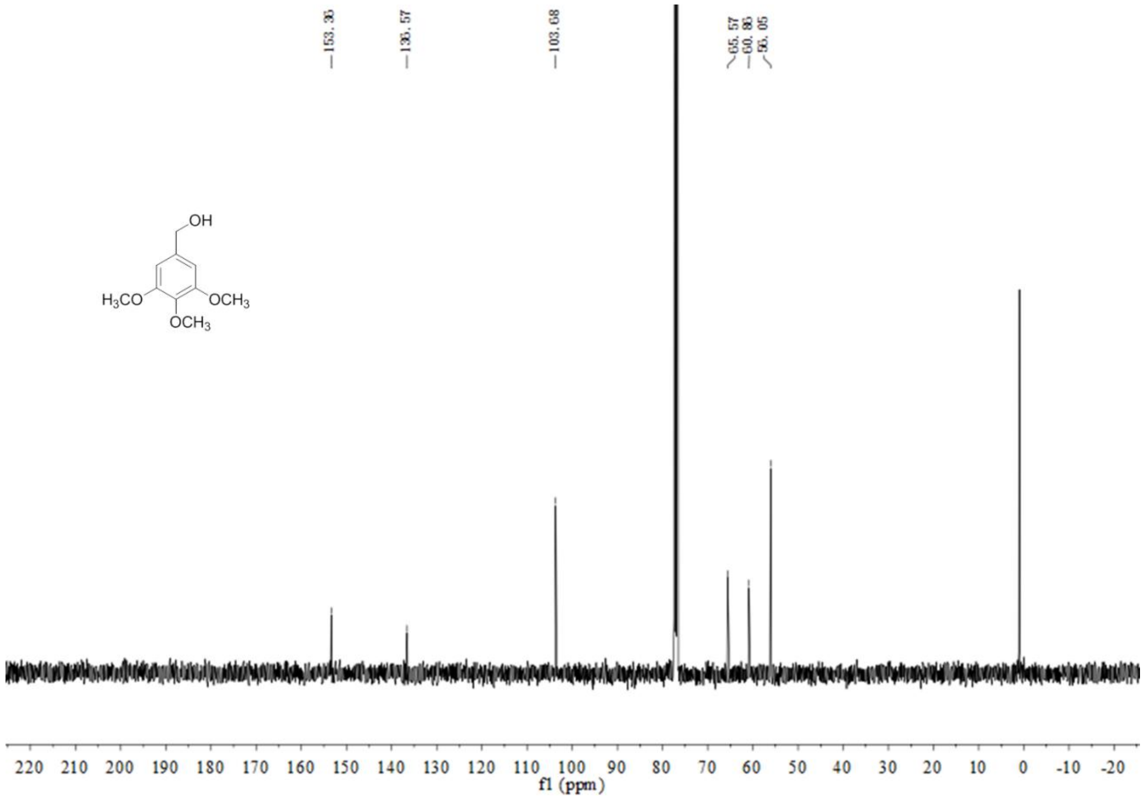

Figure S68. ^{13}C NMR spectrum for compound (3,4,5-Trimethoxyphenyl)methanol(**2s**).

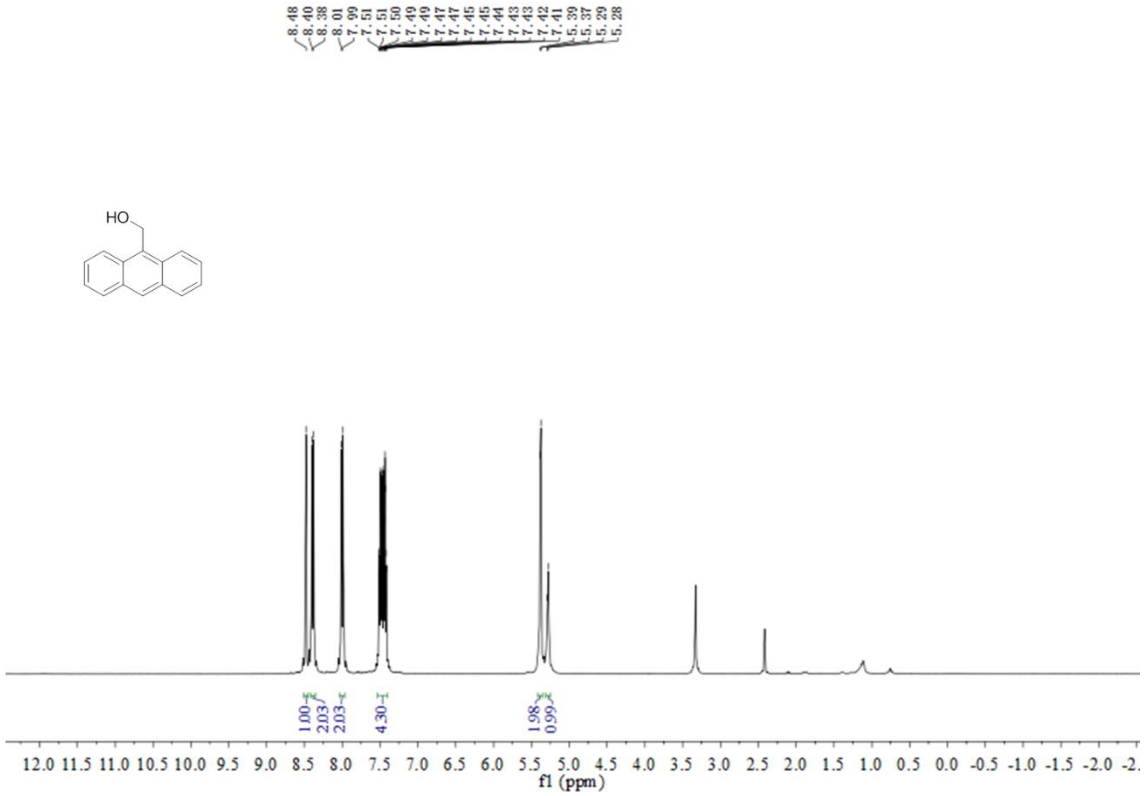

Figure S69. ^{1}H NMR spectrum for compound Anthracen-9-ylmethanol(**2t**).

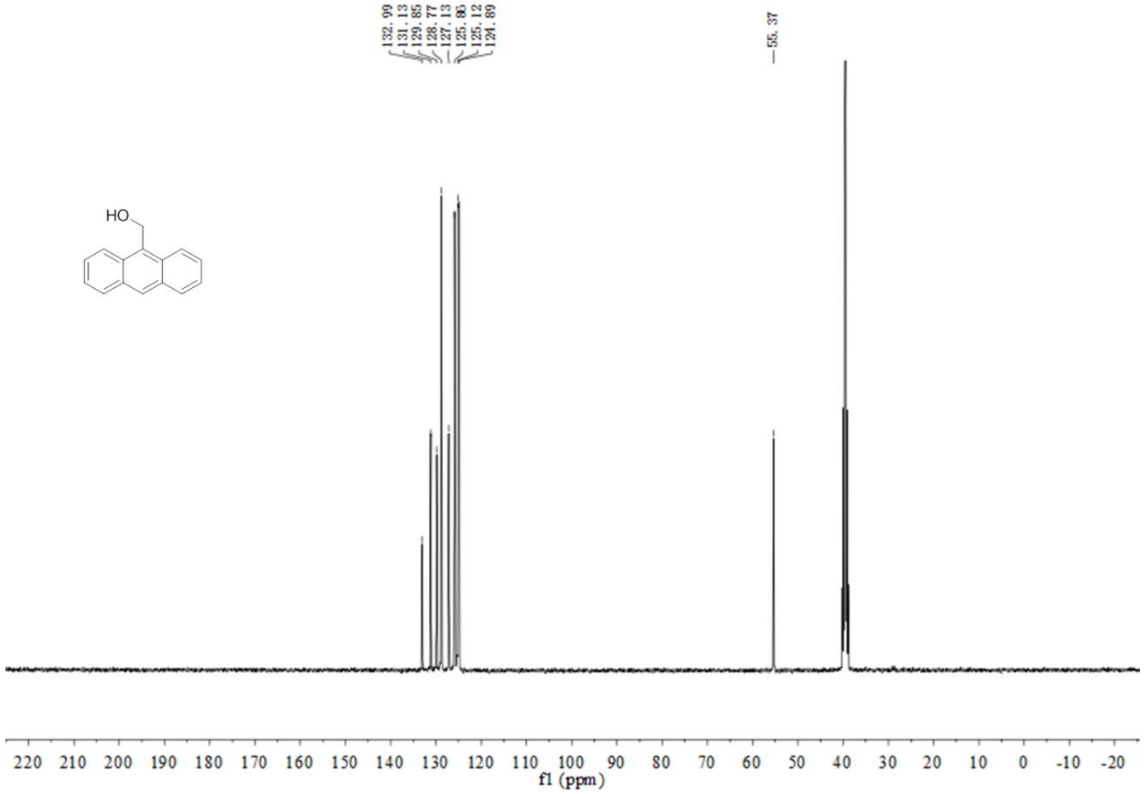

Figure S70. ^{13}C NMR spectrum for compound Anthracen-9-ylmethanol(**2t**).

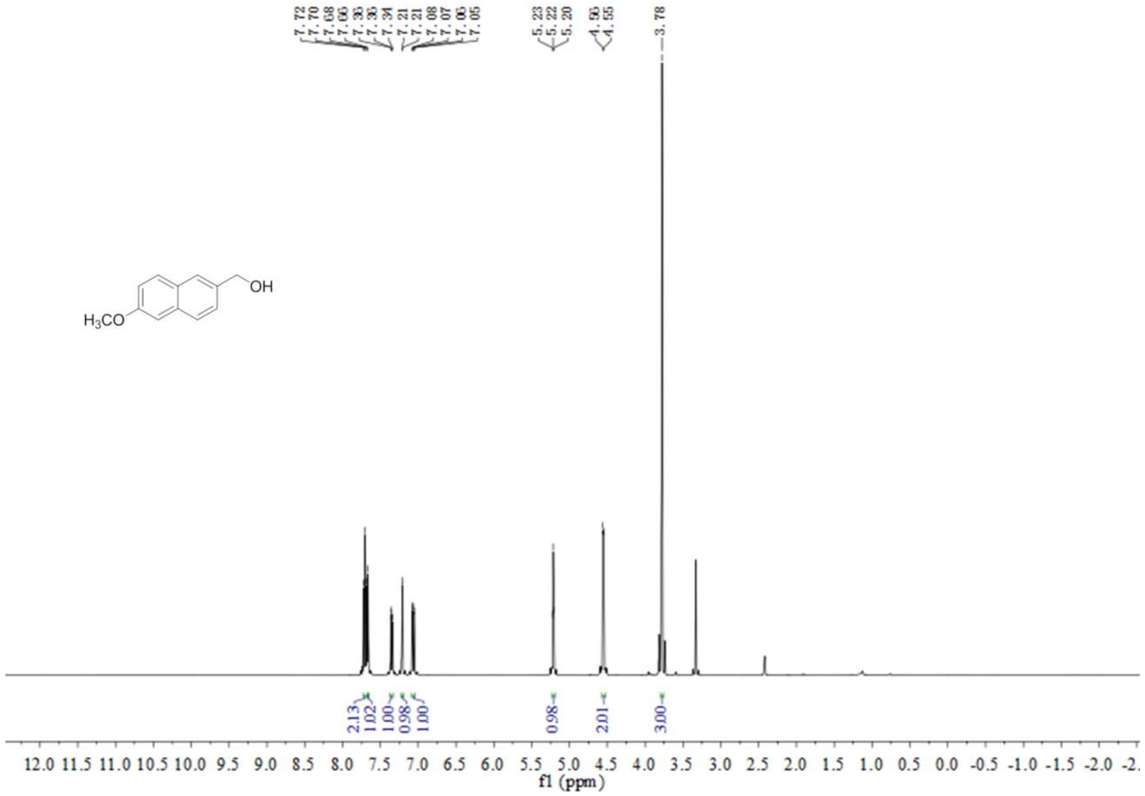

Figure S71. ^{1}H NMR spectrum for compound (6-Methoxynaphthalen-2-yl)methanol(**2u**).

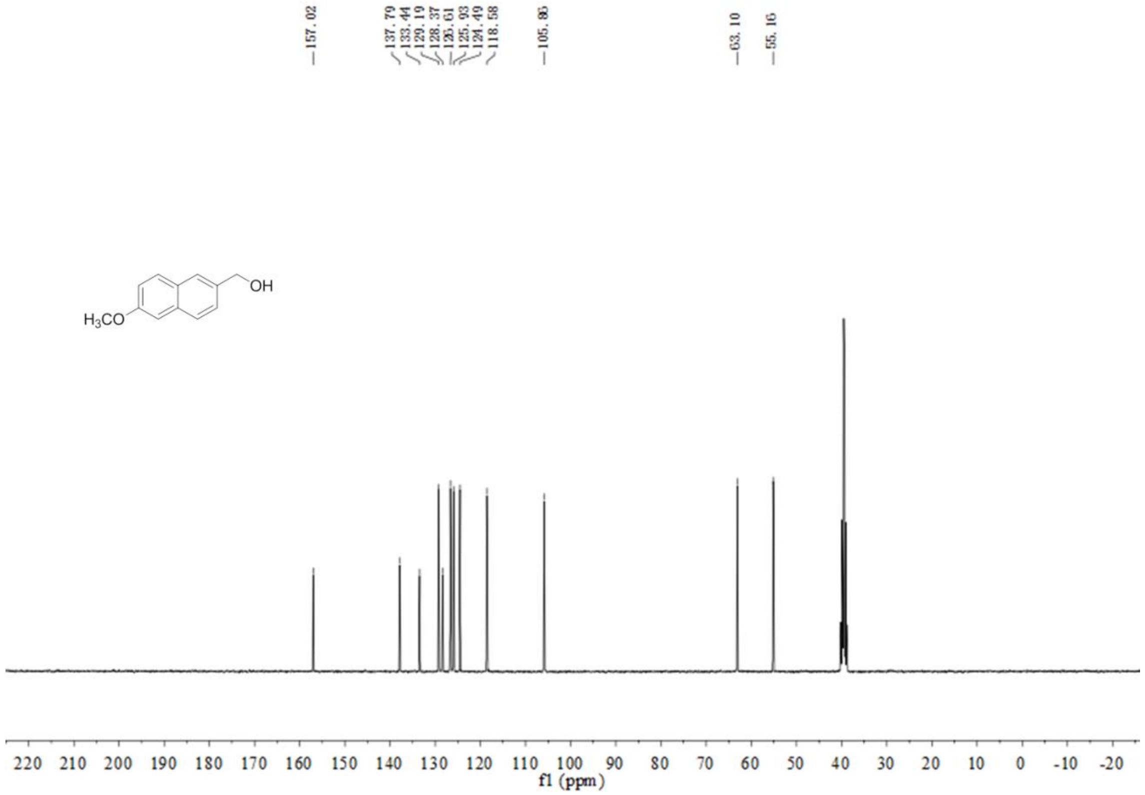

Figure S72. ^{13}C NMR spectrum for compound (6-Methoxynaphthalen-2-yl)methanol(**2u**).

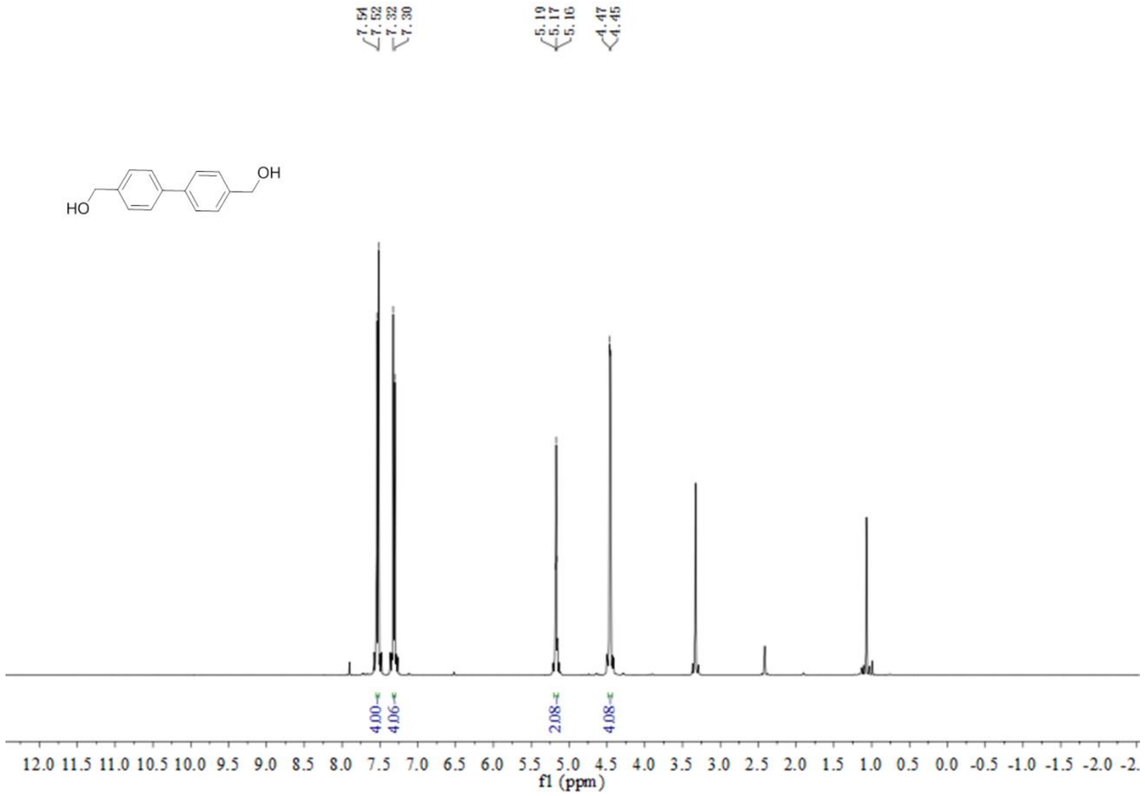

Figure S73. ^{1}H NMR spectrum for compound 4,4'-Biphenyldiyldimethanol (**2v**).

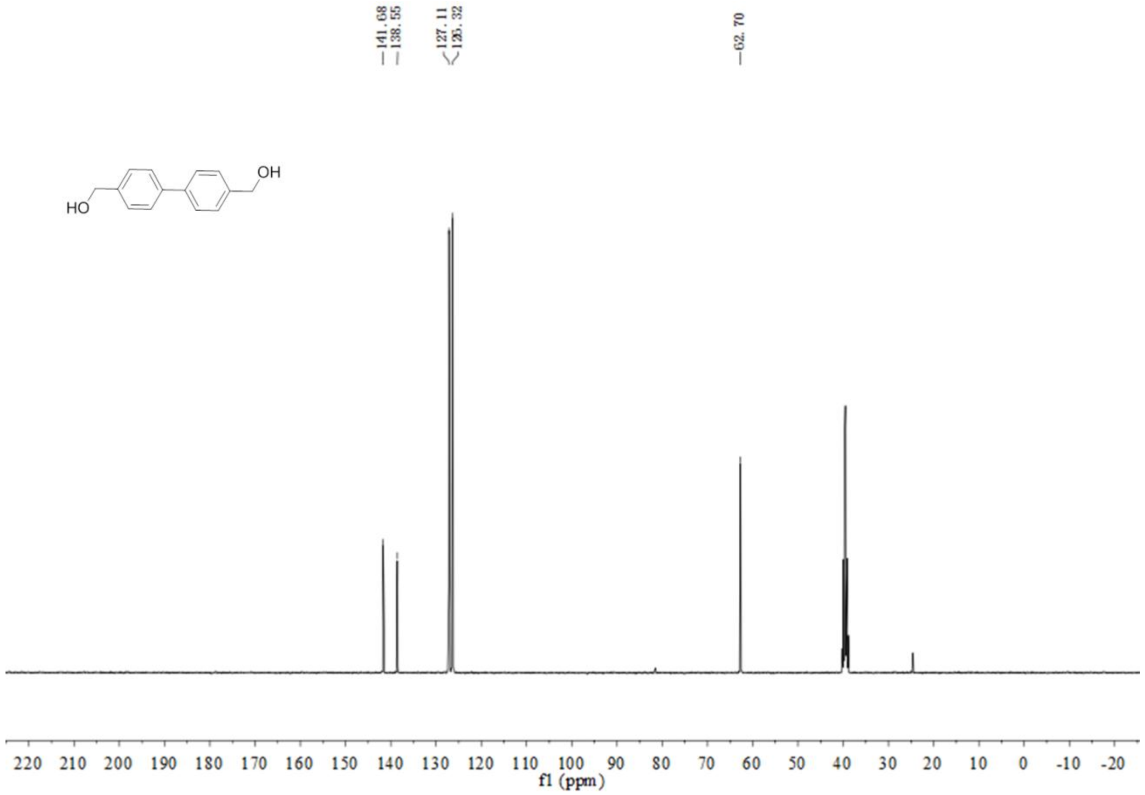

Figure S74. ^{13}C NMR spectrum for compound 4,4'-Biphenyldiyldimethanol (**2v**).

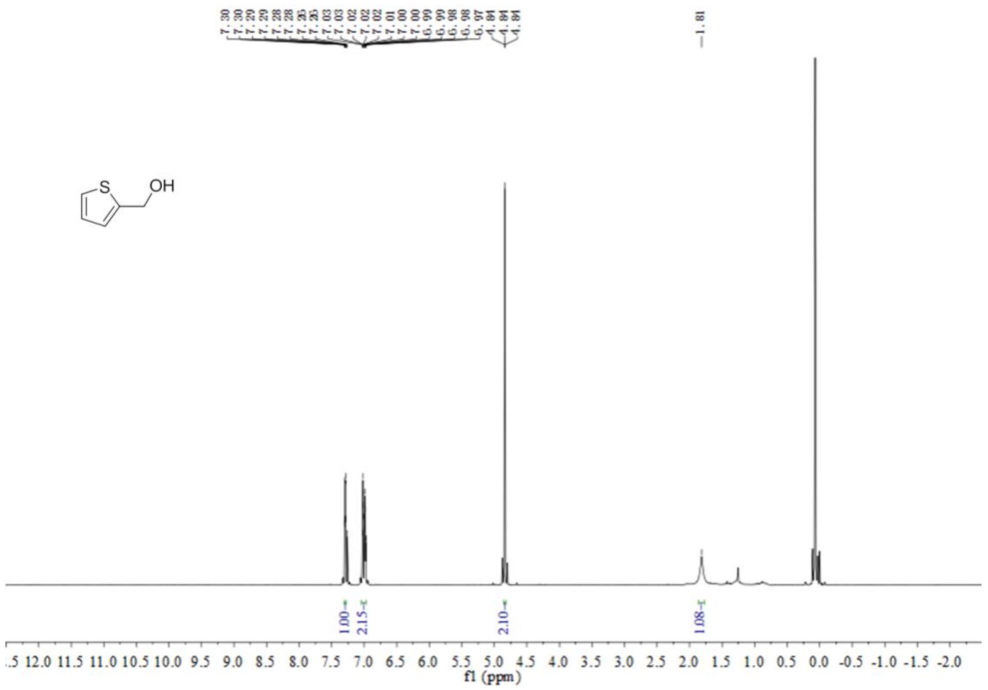

Figure S75. ^{1}H NMR spectrum for compound Thiophen-2-ylmethanol (**2w**).

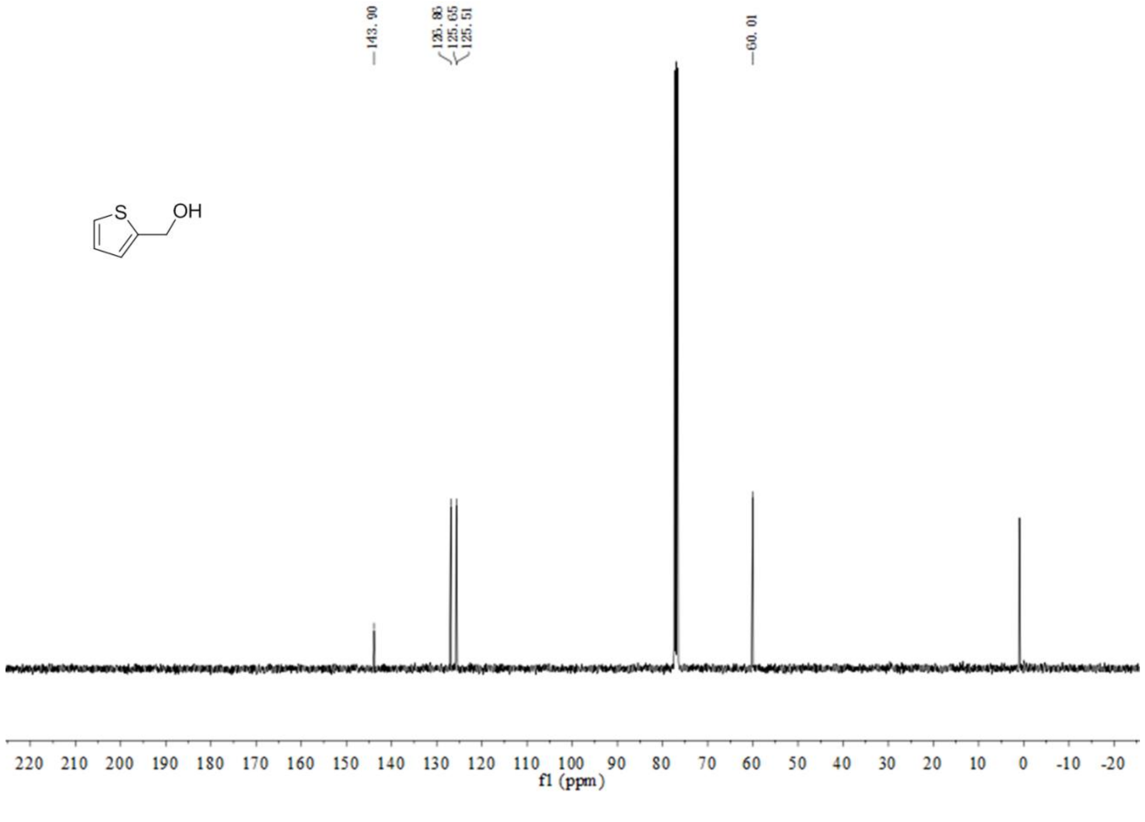

Figure S76. ^{13}C NMR spectrum for compound Thiophen-2-ylmethanol (**2w**).

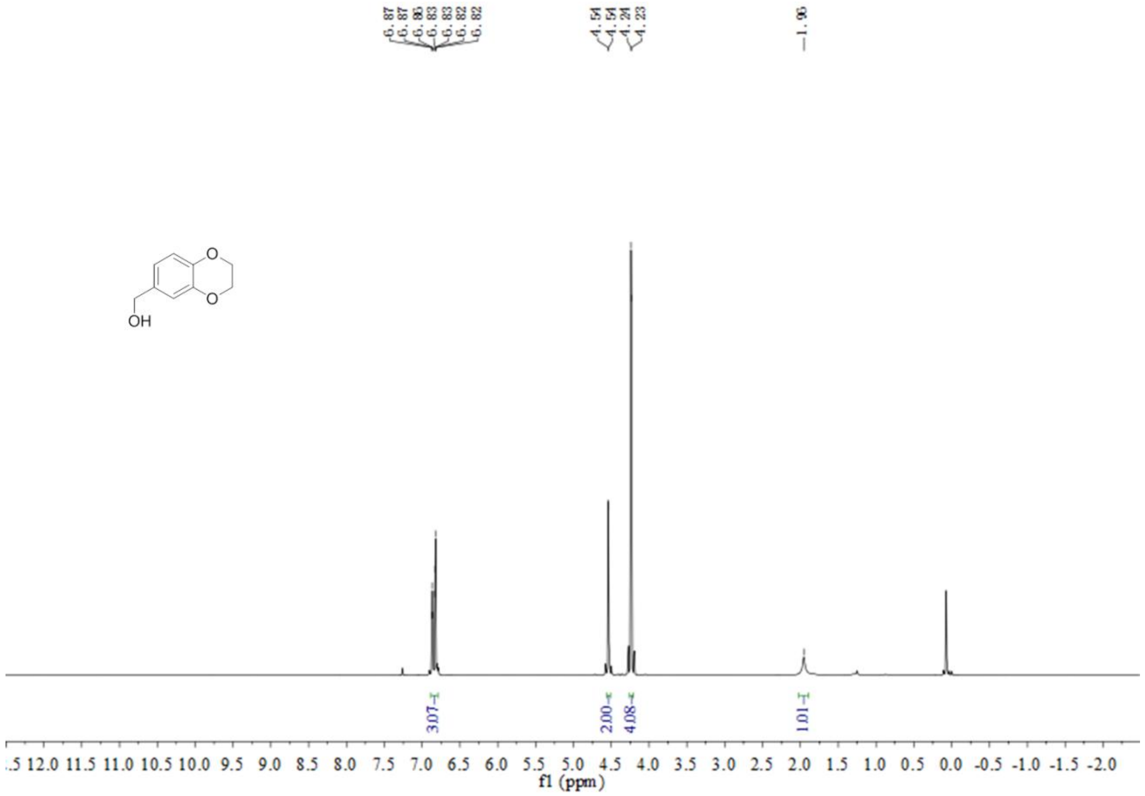

Figure S77. ^{1}H NMR spectrum for compound (2,3-Dihydrobenzo[b][1,4]dioxin-6-yl)methanol (**2x**).

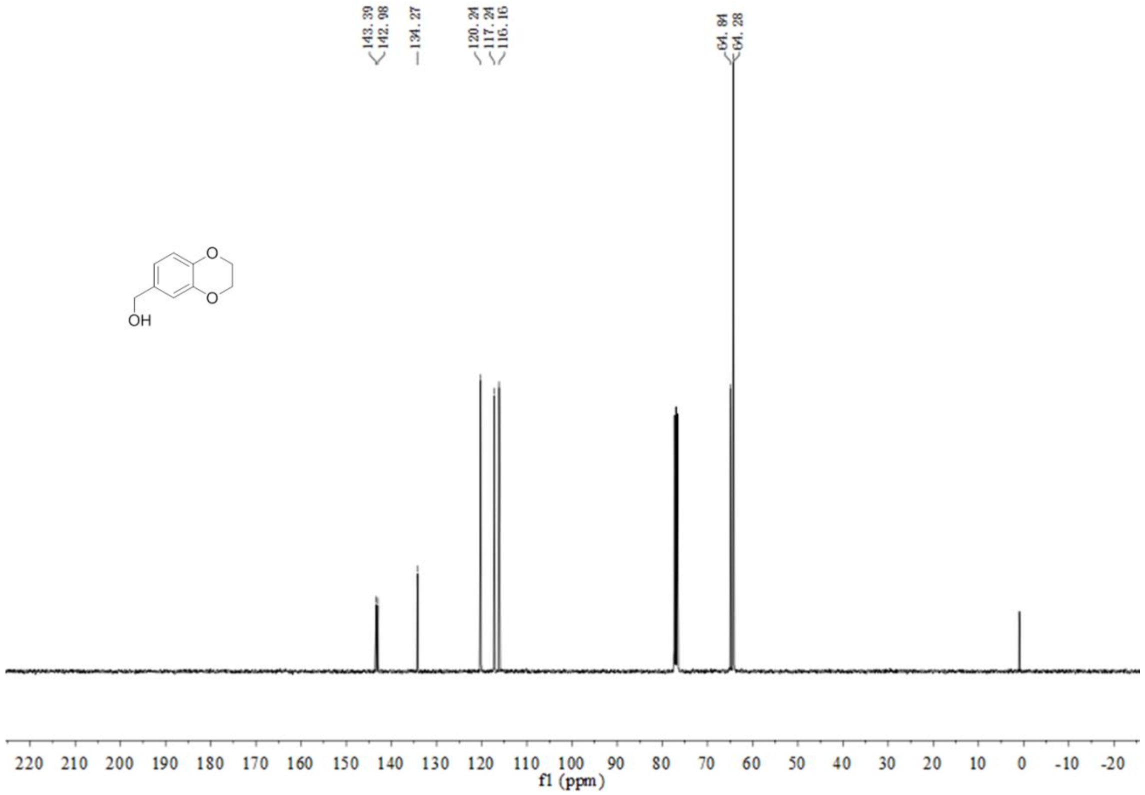

Figure S78. ^{13}C NMR spectrum for compound (2,3-Dihydrobenzo[b][1,4]dioxin-6-yl)methanol (**2x**).

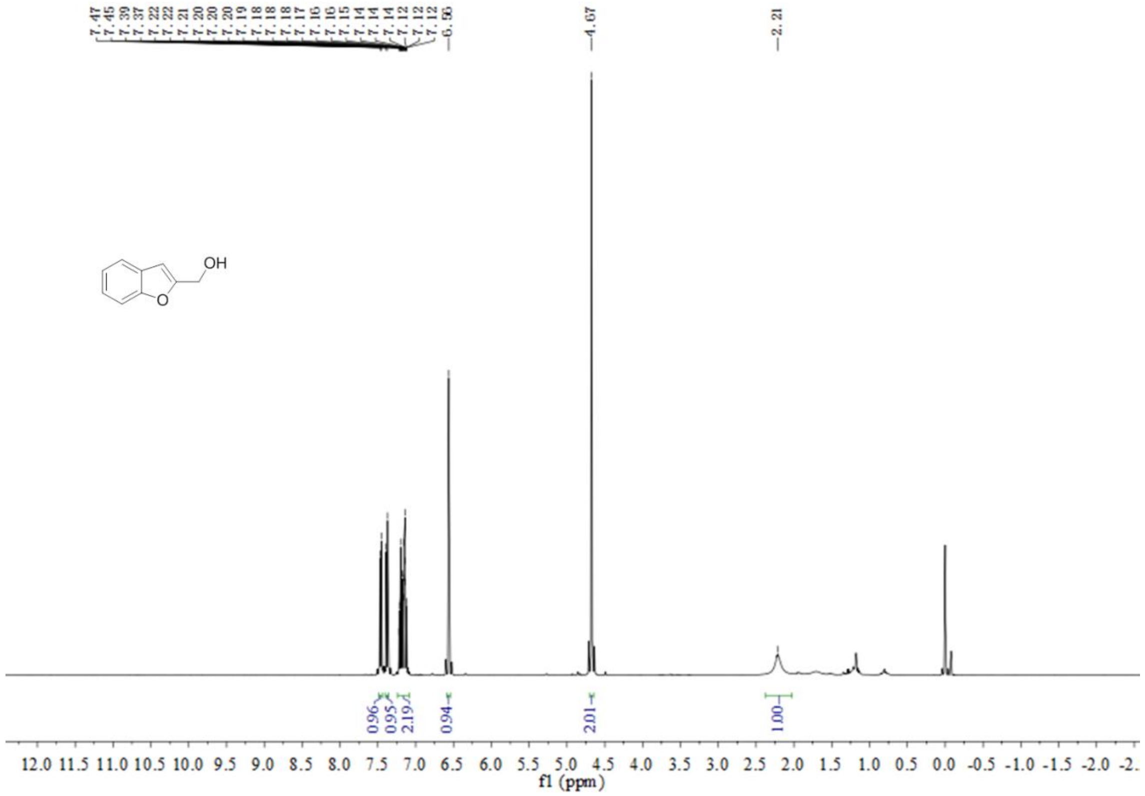

Figure S79. ^{1}H NMR spectrum for compound Benzofuran-2-ylmethanol(**2y**).

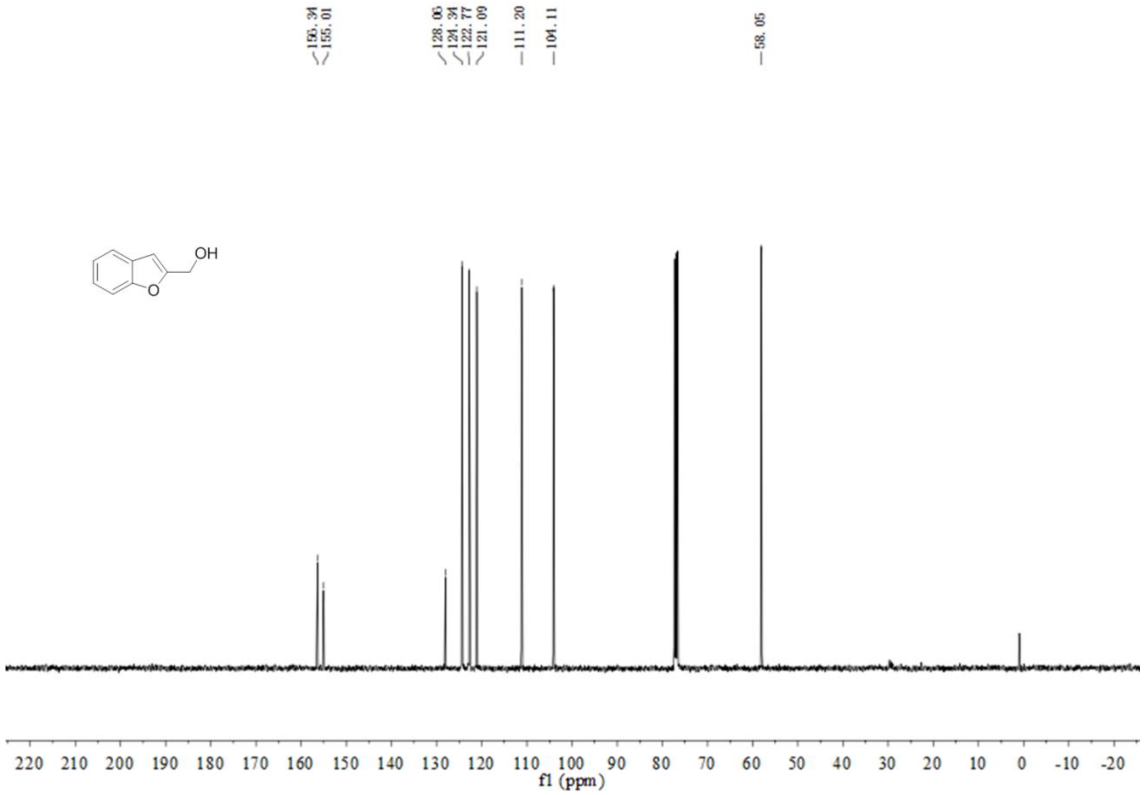

Figure S80. ^{13}C NMR spectrum for compound Benzofuran-2-ylmethanol(**2y**).

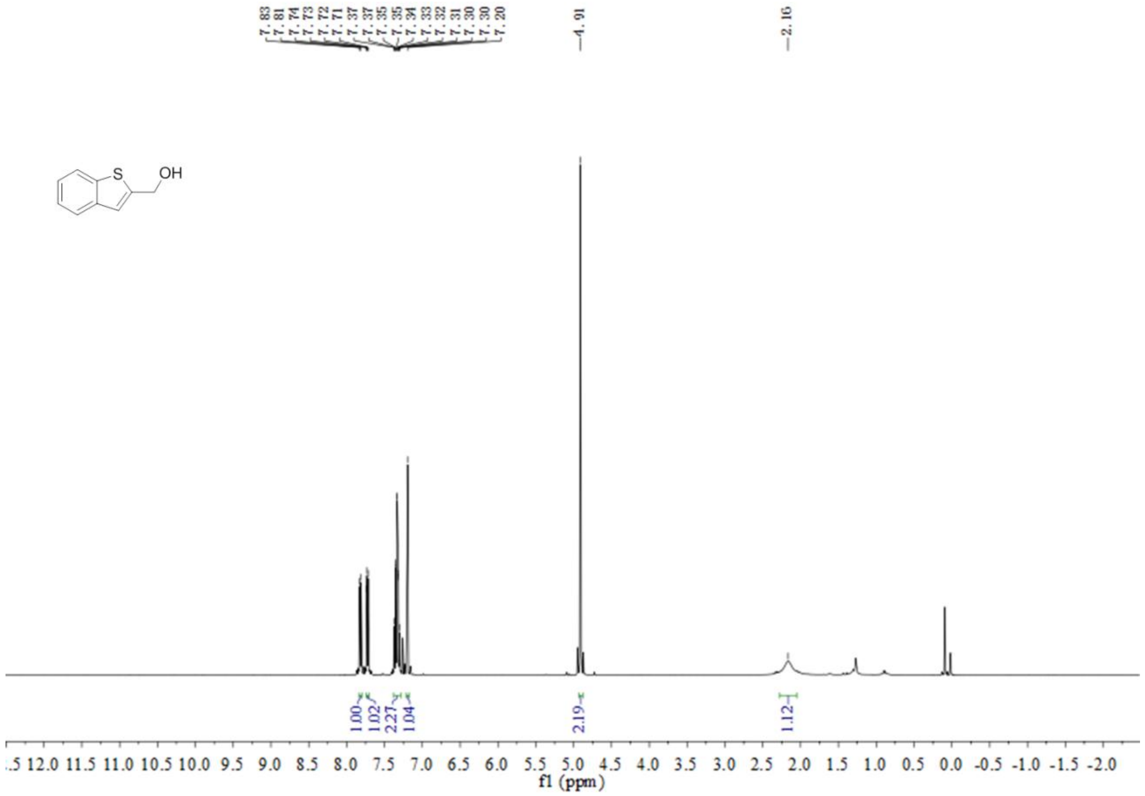

Figure S81. ^{1}H NMR spectrum for compound Benzo[b]thiophen-2-ylmethanol (**2z**).

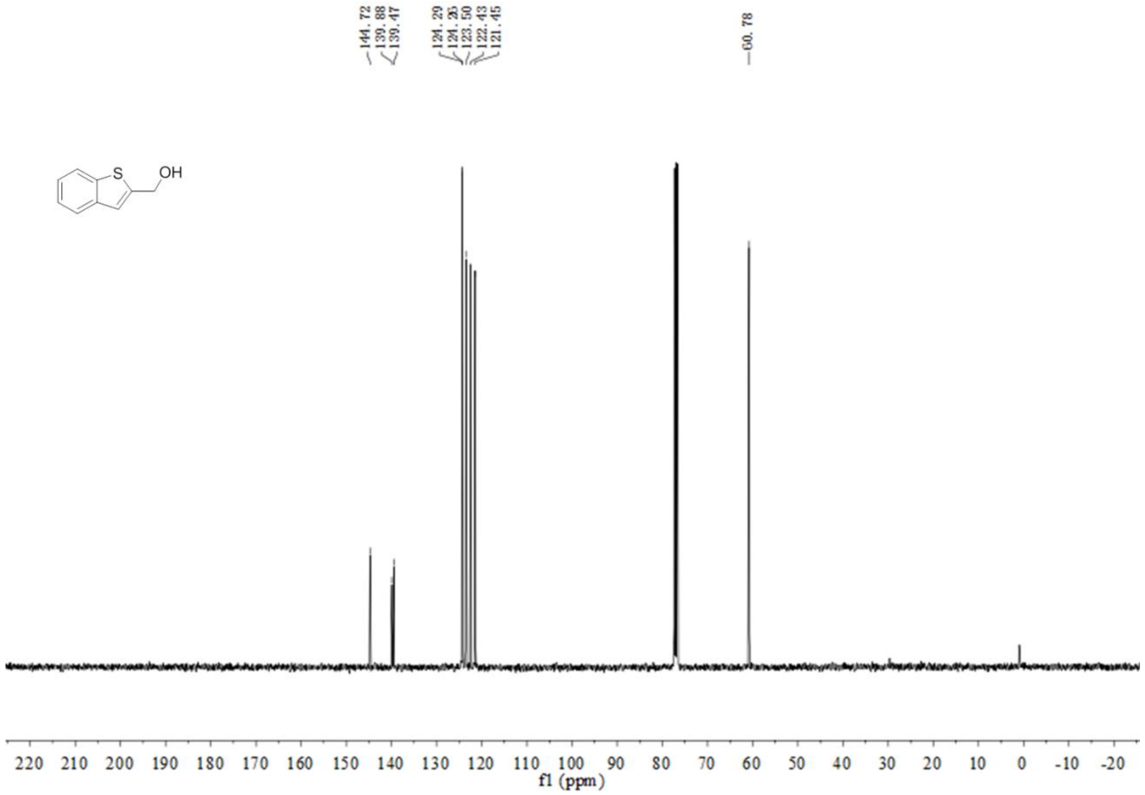

Figure S82. ^{13}C NMR spectrum for compound Benzo[b]thiophen-2-ylmethanol (**2z**).

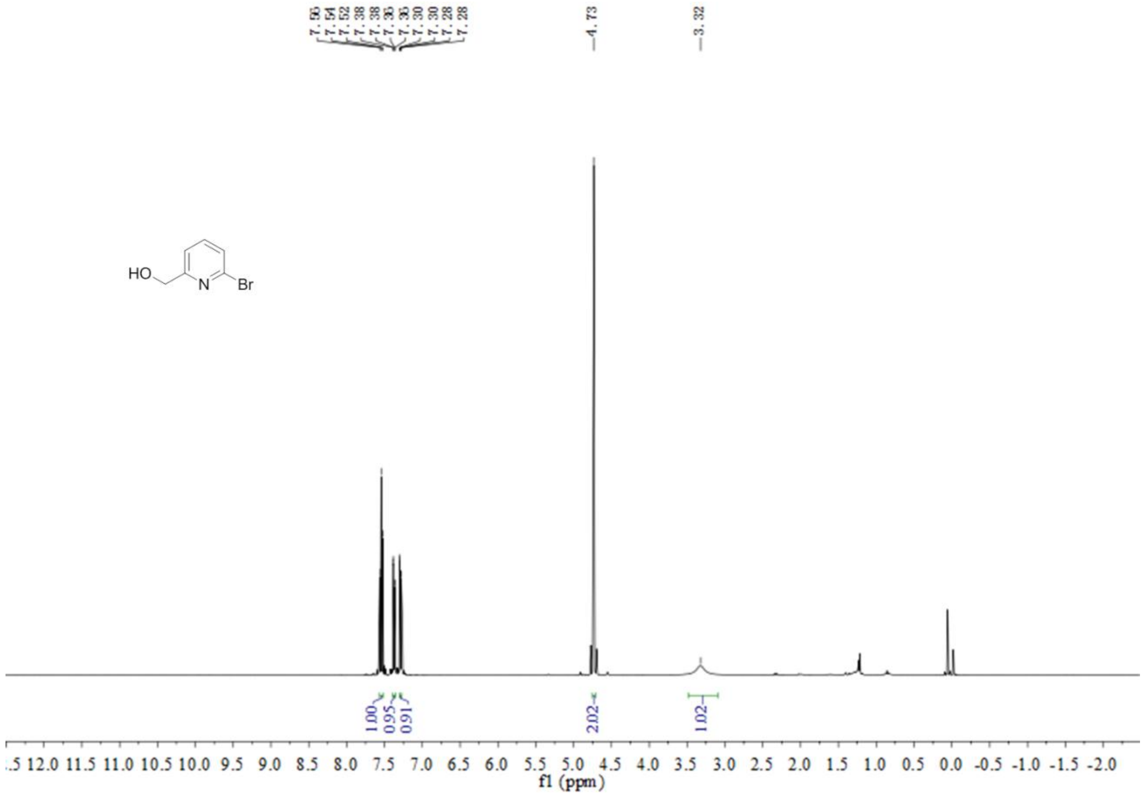

Figure S83. ^{1}H NMR spectrum for compound (6-Bromopyridin-2-yl)methanol (**2aa**).

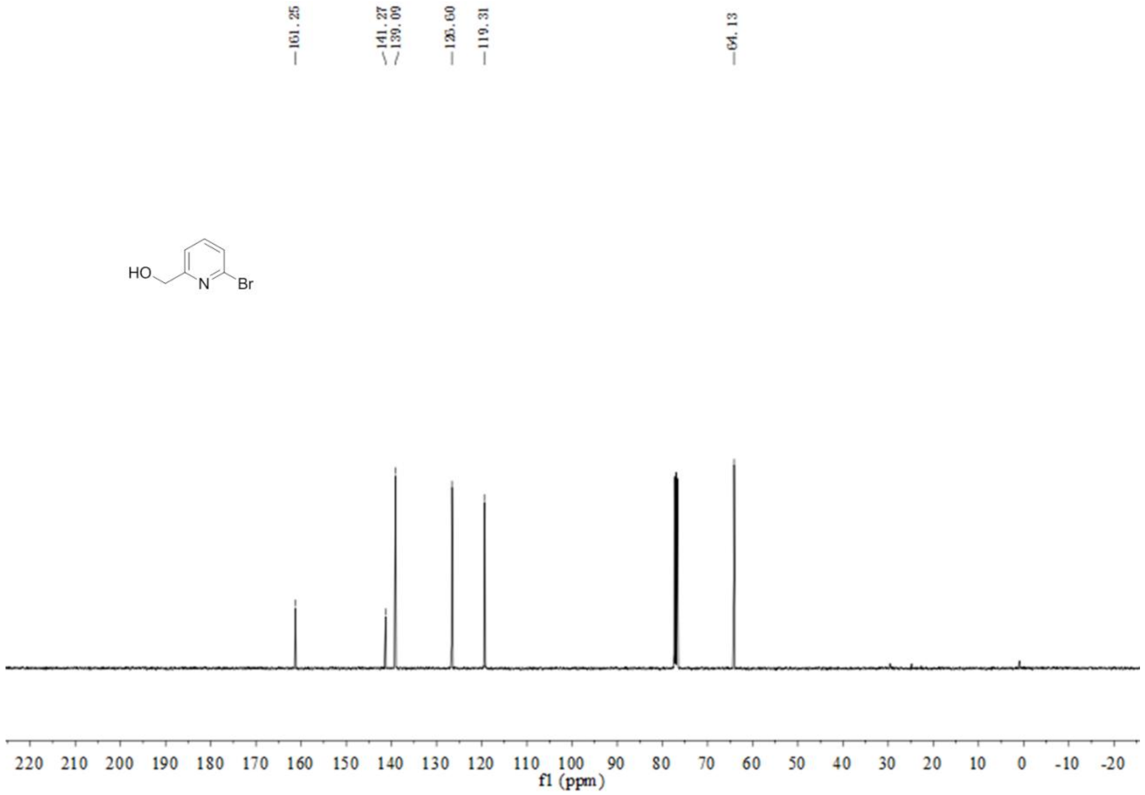

Figure S84. ^{13}C NMR spectrum for compound (6-Bromopyridin-2-yl)methanol (**2aa**).

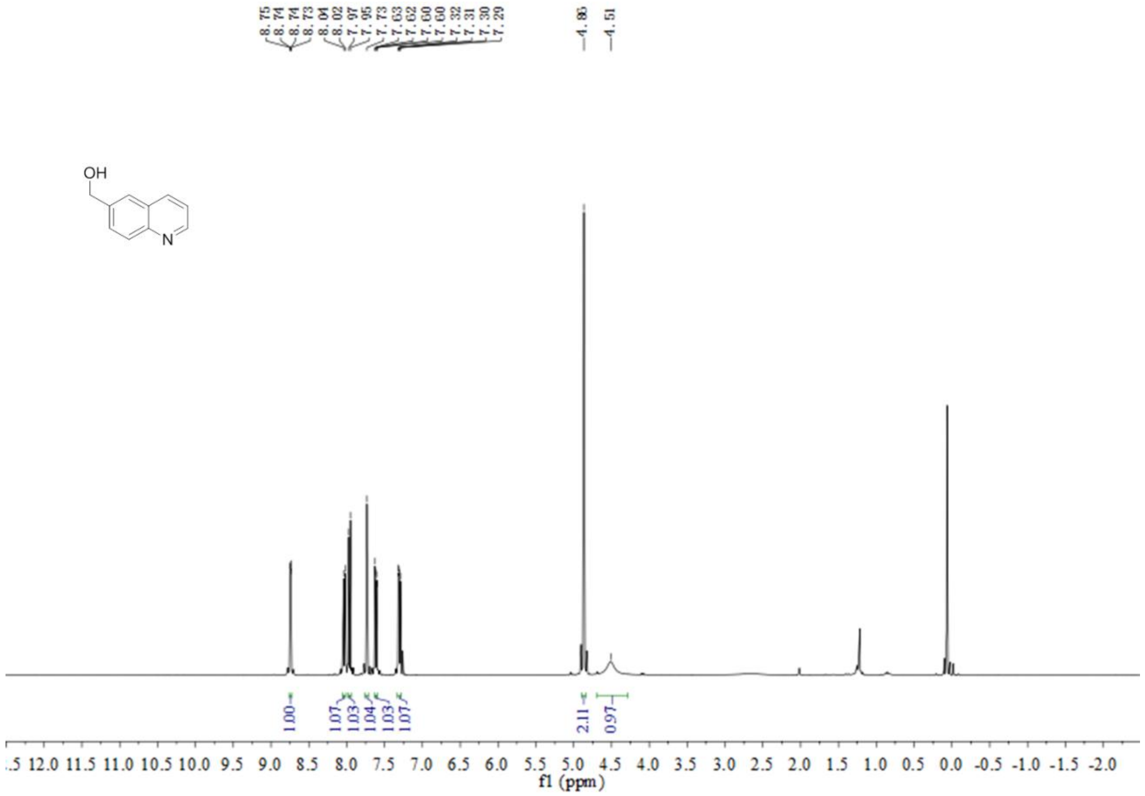

Figure S85. ^{1}H NMR spectrum for compound Quinolin-6-ylmethanol (**2ab**).

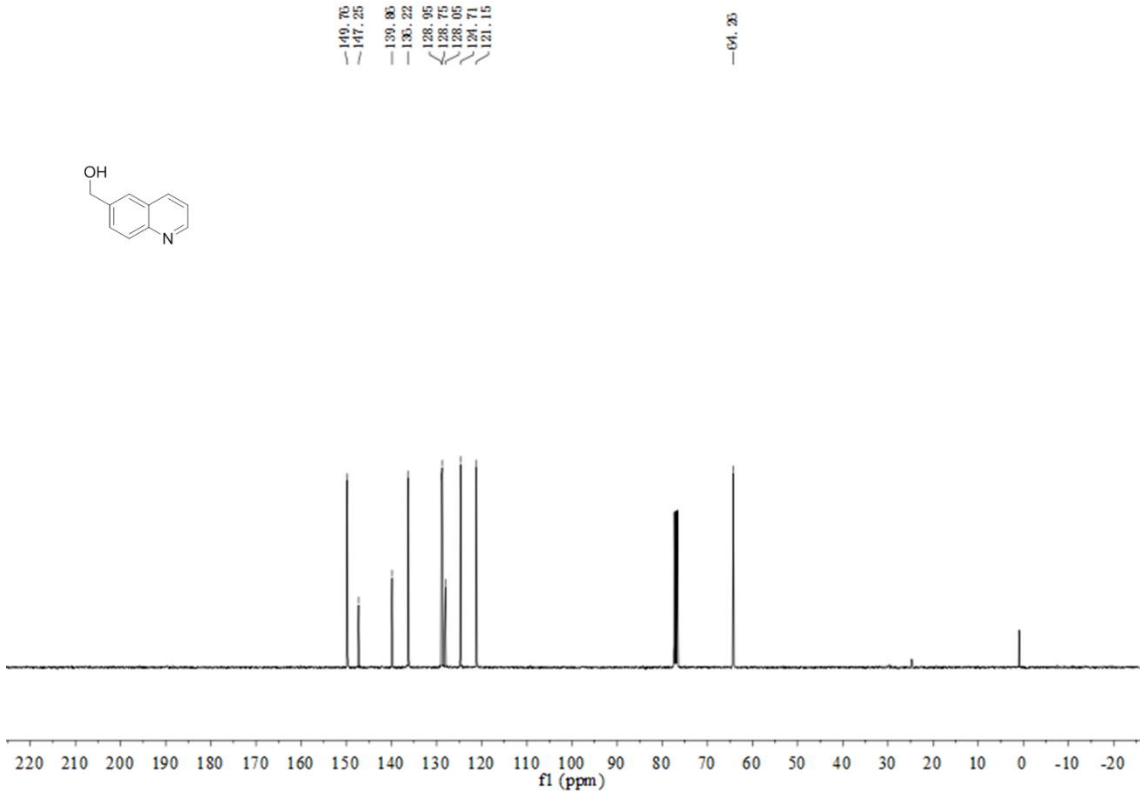

Figure S86. ^{13}C NMR spectrum for compound Quinolin-6-ylmethanol (**2ab**).

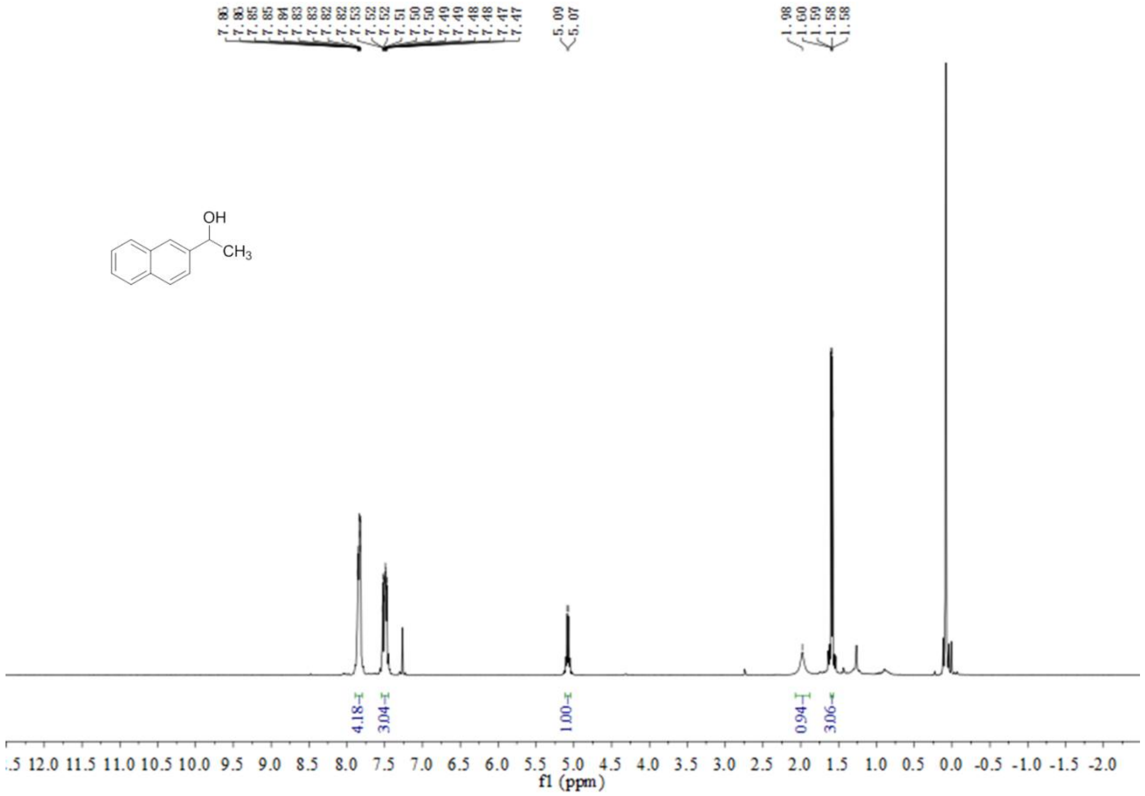

Figure S87. ^{1}H NMR spectrum for compound 1-(Naphthalen-2-yl)ethanol(**4a**).

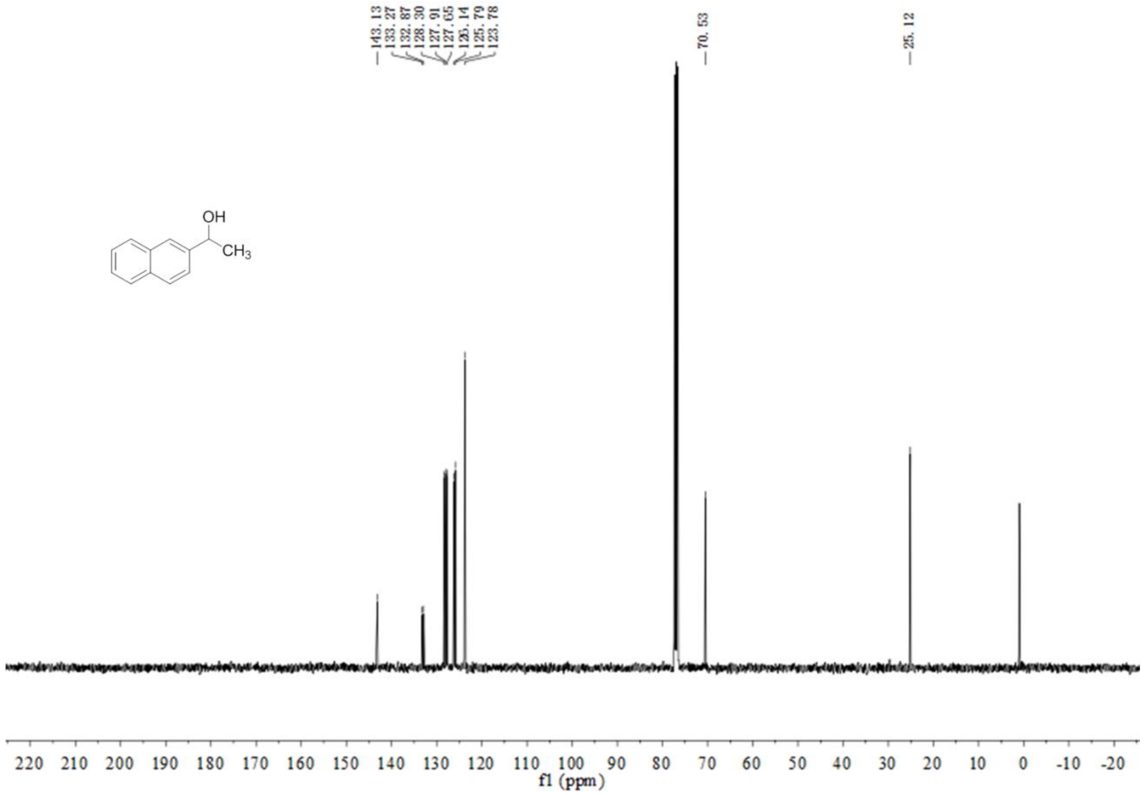

Figure S88. ^{13}C NMR spectrum for compound 1-(Naphthalen-2-yl)ethanol (**4a**).

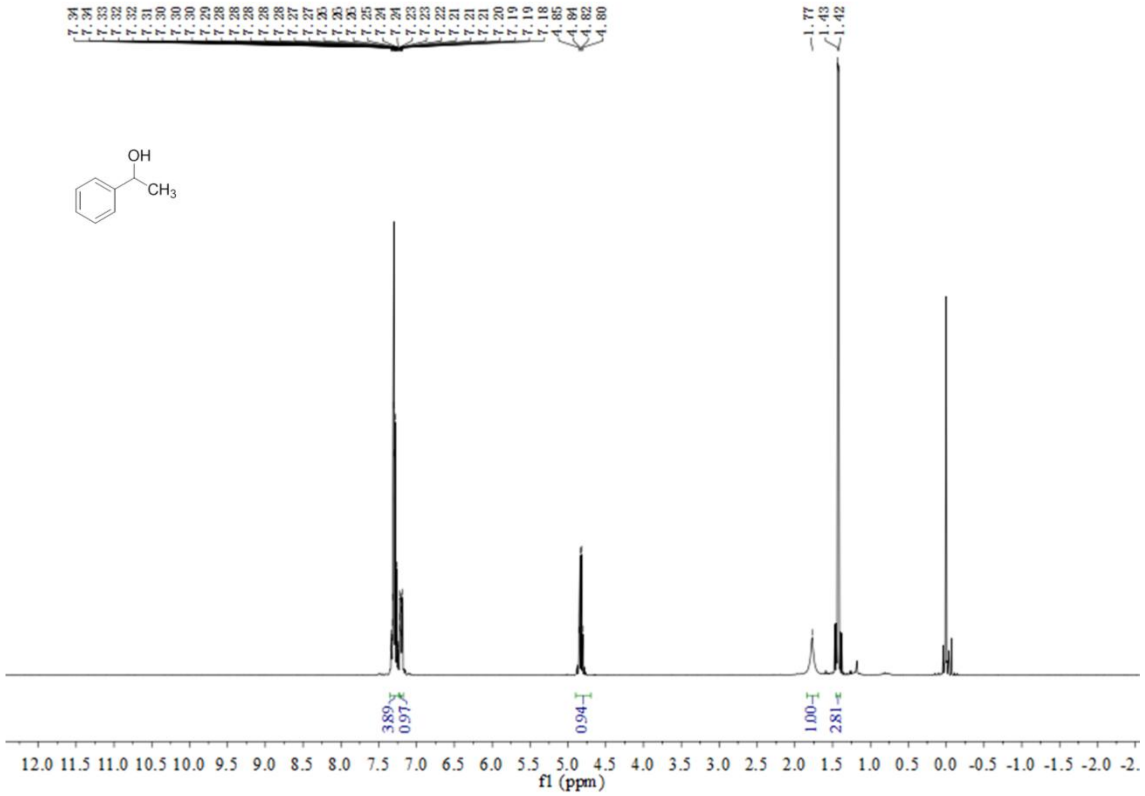

Figure S89. ^{1}H NMR spectrum for compound 1-Phenylethanol(**4b**).

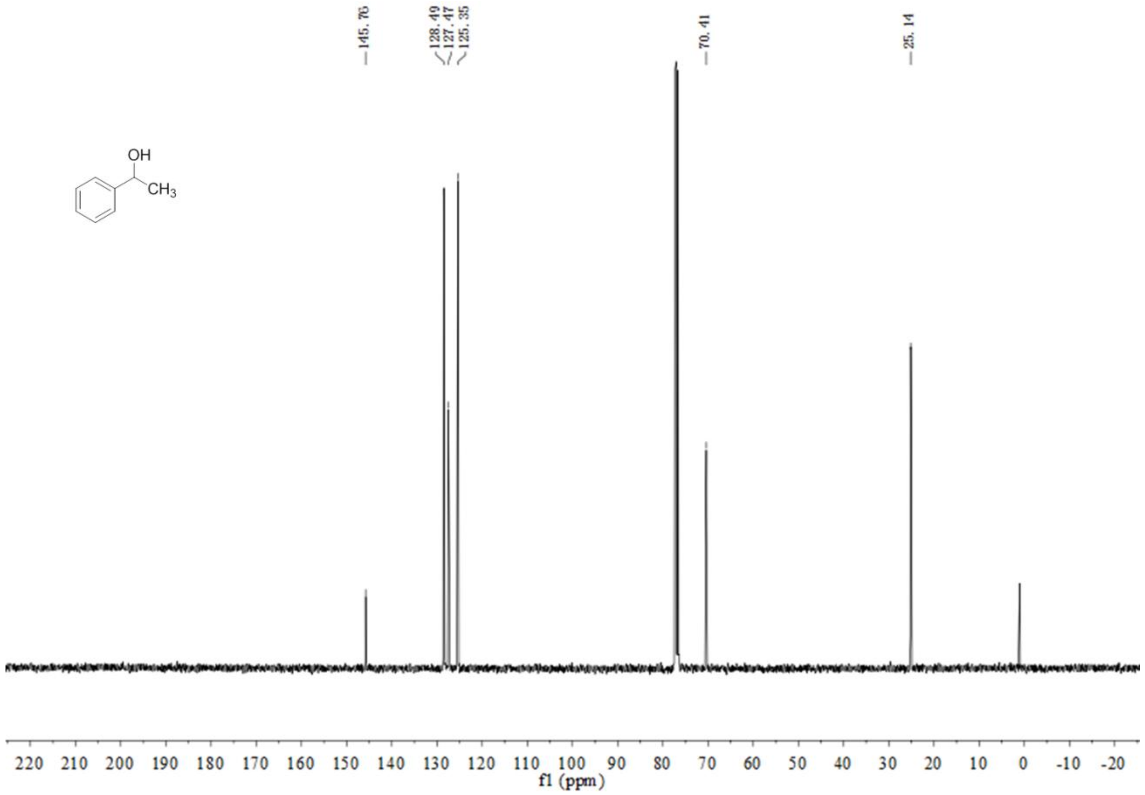

Figure S90. ^{13}C NMR spectrum for compound 1-Phenylethanol(**4b**).

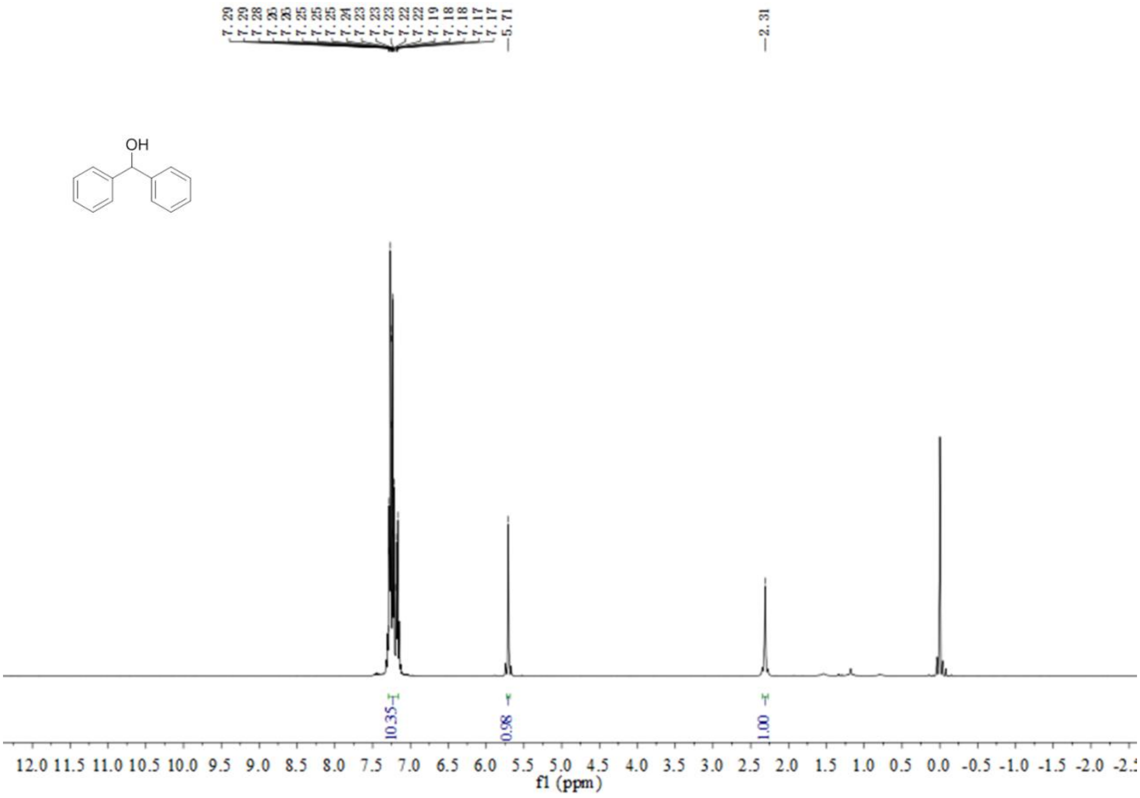

Figure S91. ^{1}H NMR spectrum for compound Diphenylmethanol(**4c**).

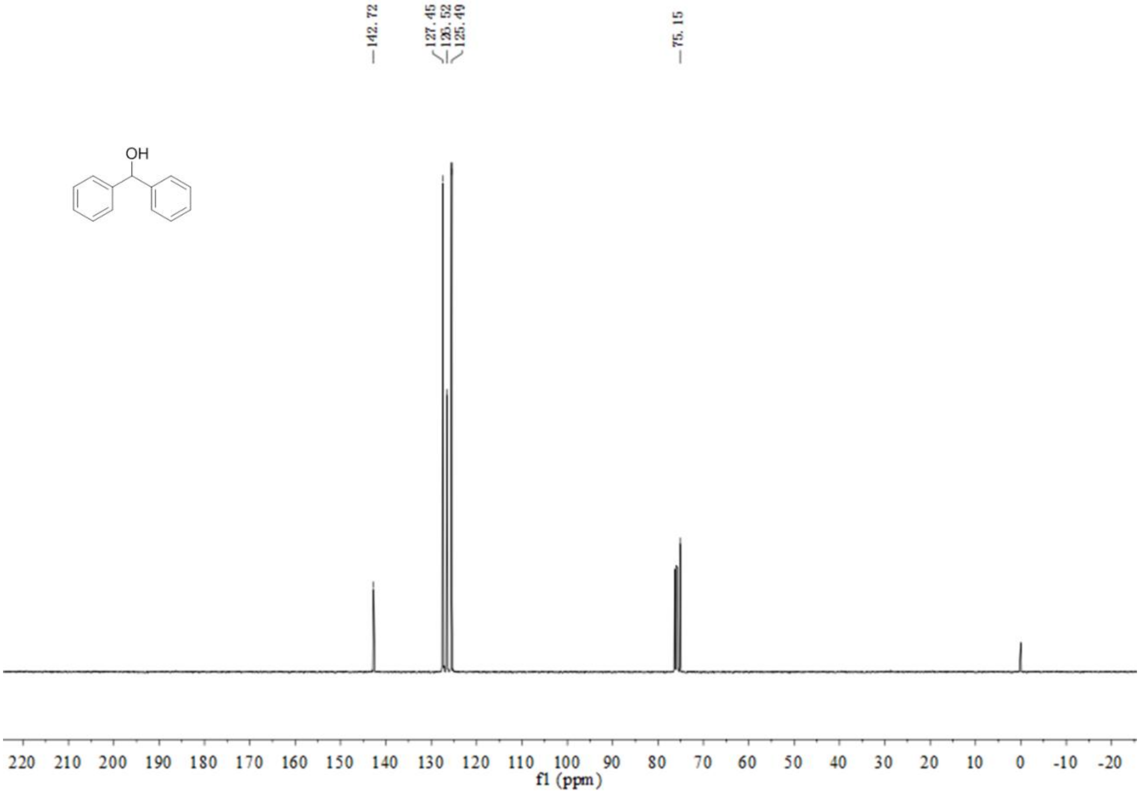

Figure S92. ^{13}C NMR spectrum for compound Diphenylmethanol(**4c**).

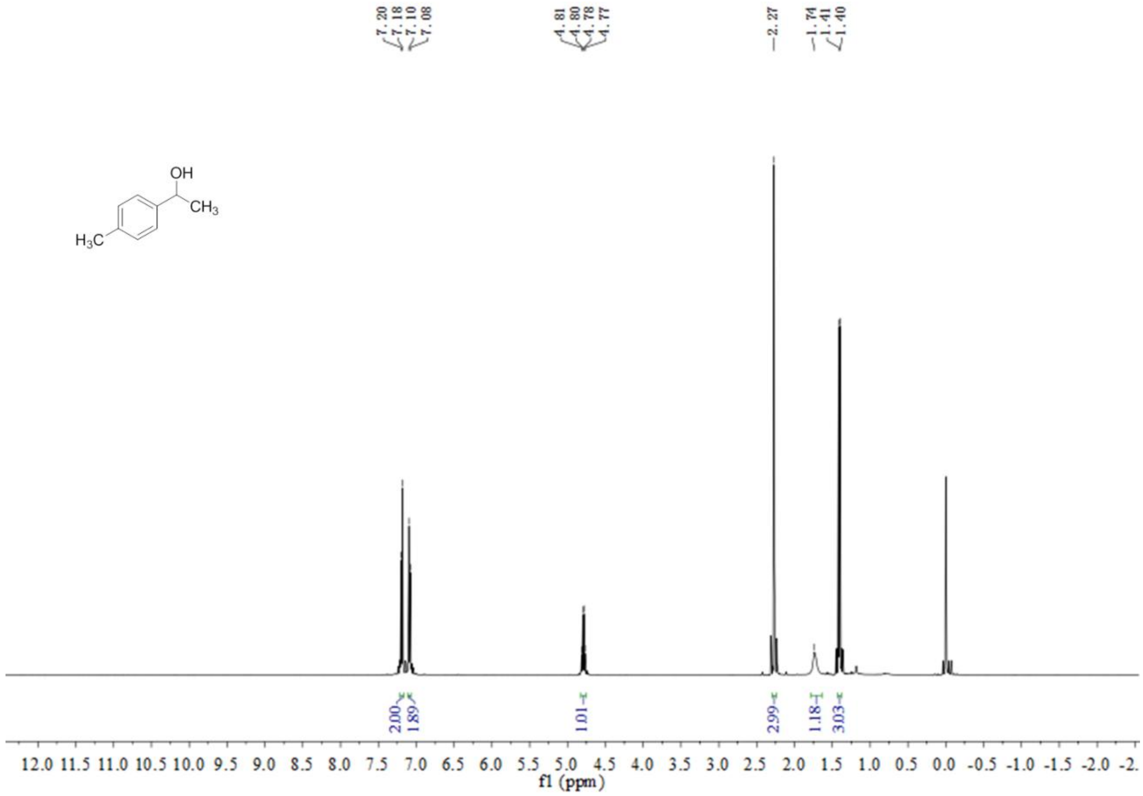

Figure S93. ^{1}H NMR spectrum for compound1-(p-tolyl)ethanol(**4d**).

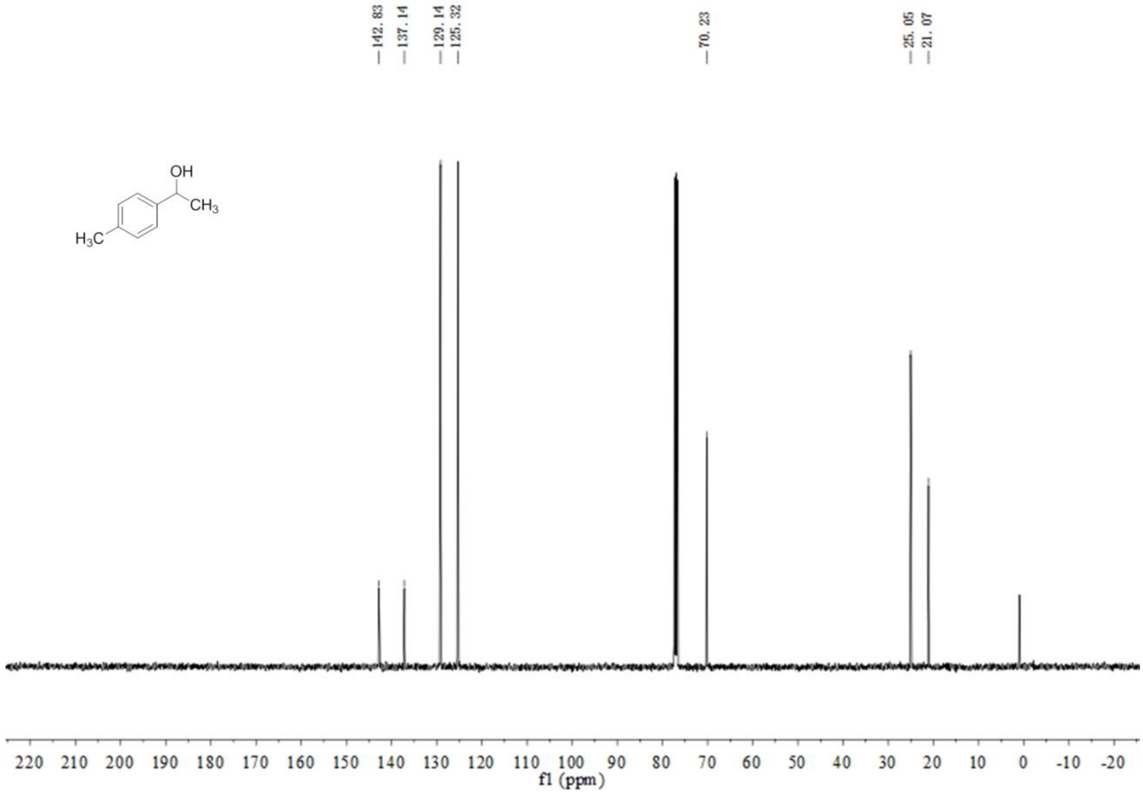

Figure S94. ^{13}C NMR spectrum for compound 1-(p-tolyl)ethanol(**4d**).

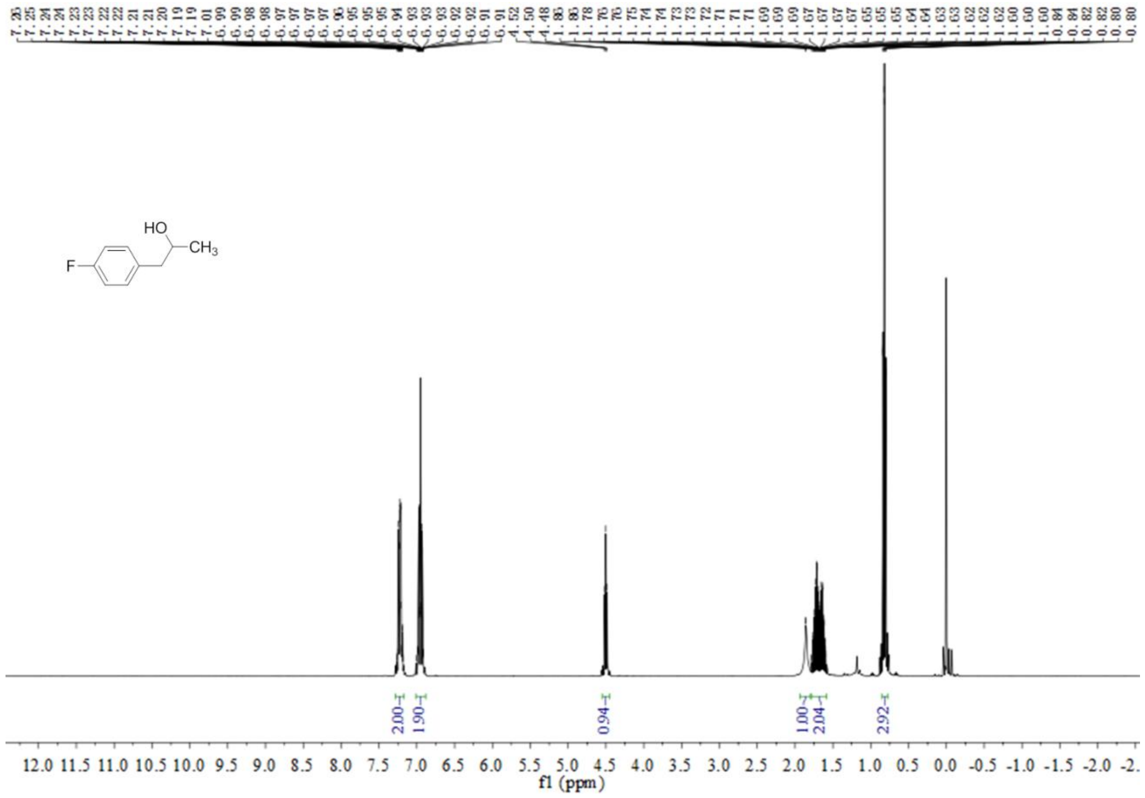

Figure S95. ^{1}H NMR spectrum for compound1-(4-Fluorophenyl)propan-2-ol(**4e**).

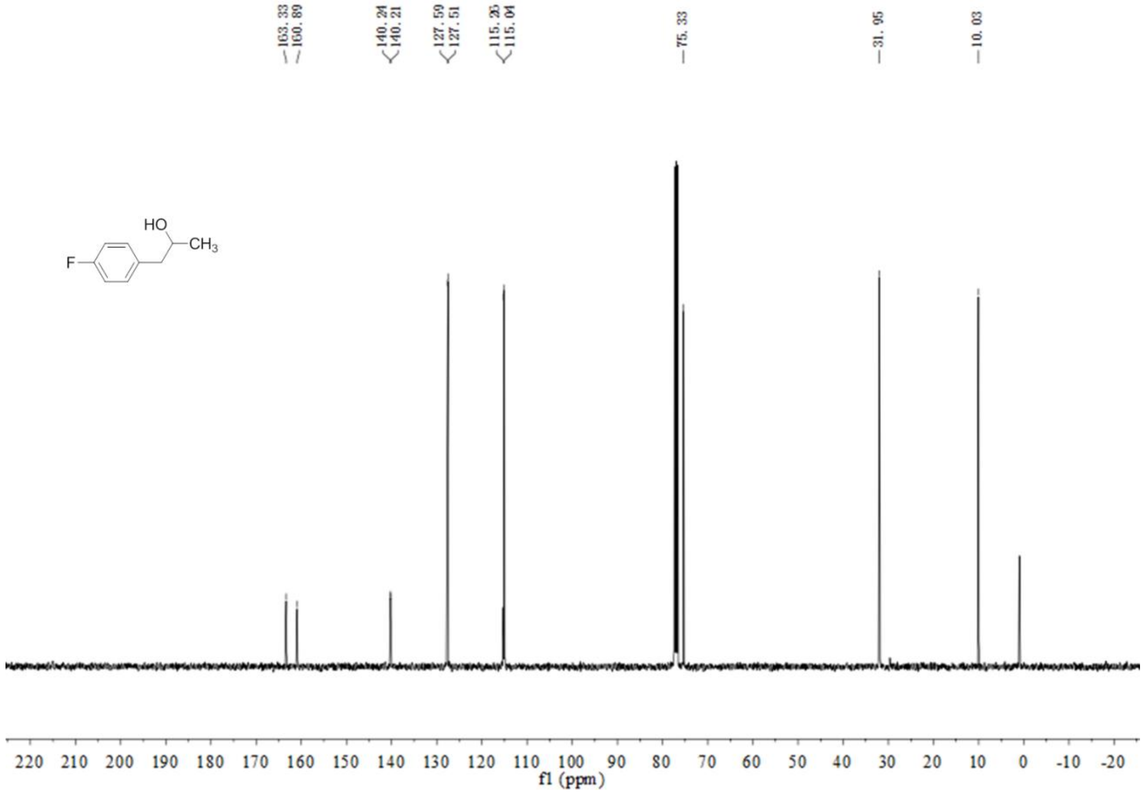

Figure S96. ^{13}C NMR spectrum for compound 1-(4-Fluorophenyl)propan-2-ol(**4e**).

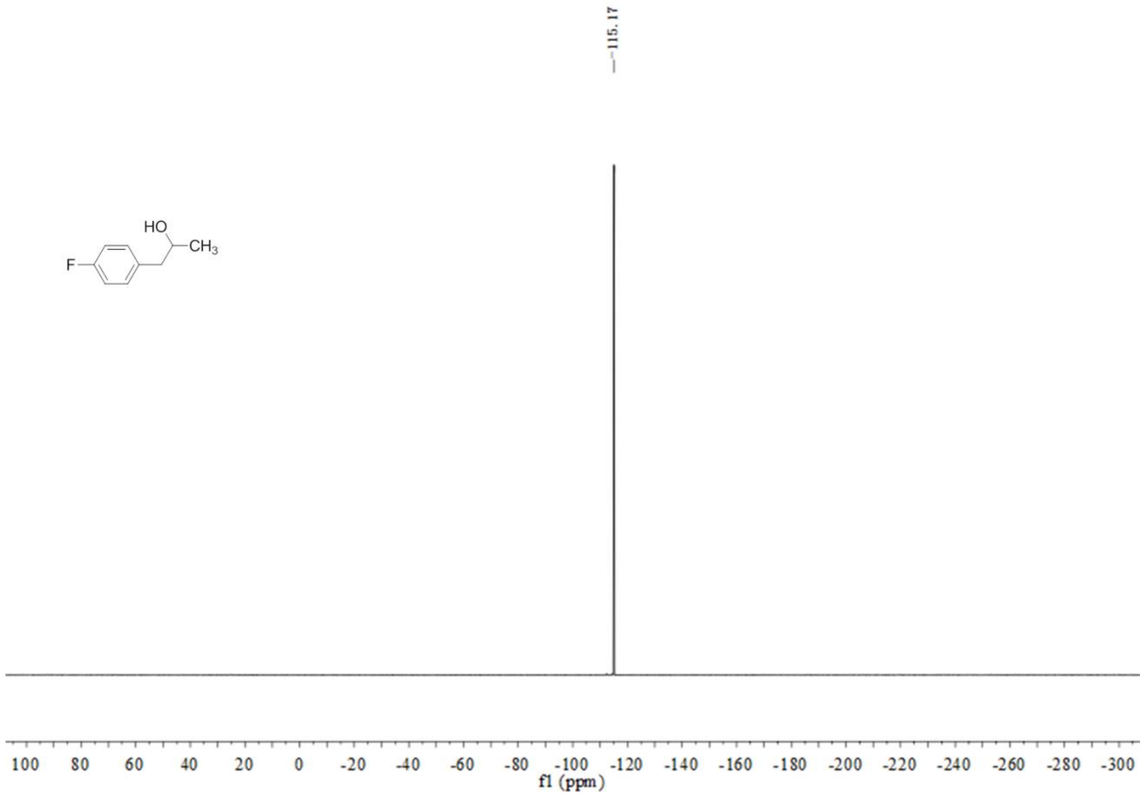

Figure S97. ^{19}F NMR spectrum for compound 1-(4-Fluorophenyl)propan-2-ol(**4e**).

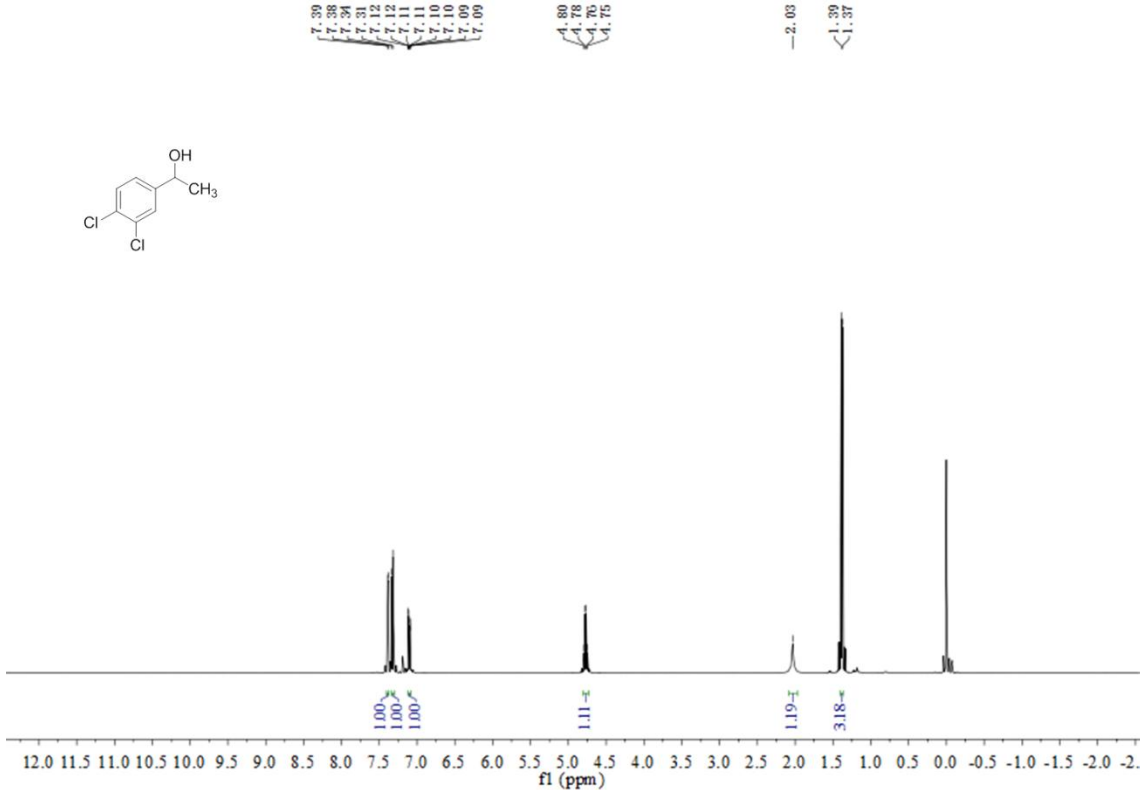

Figure S98. ^{1}H NMR spectrum for compound1-(3,4-Dichlorophenyl)ethanol(**4f**).

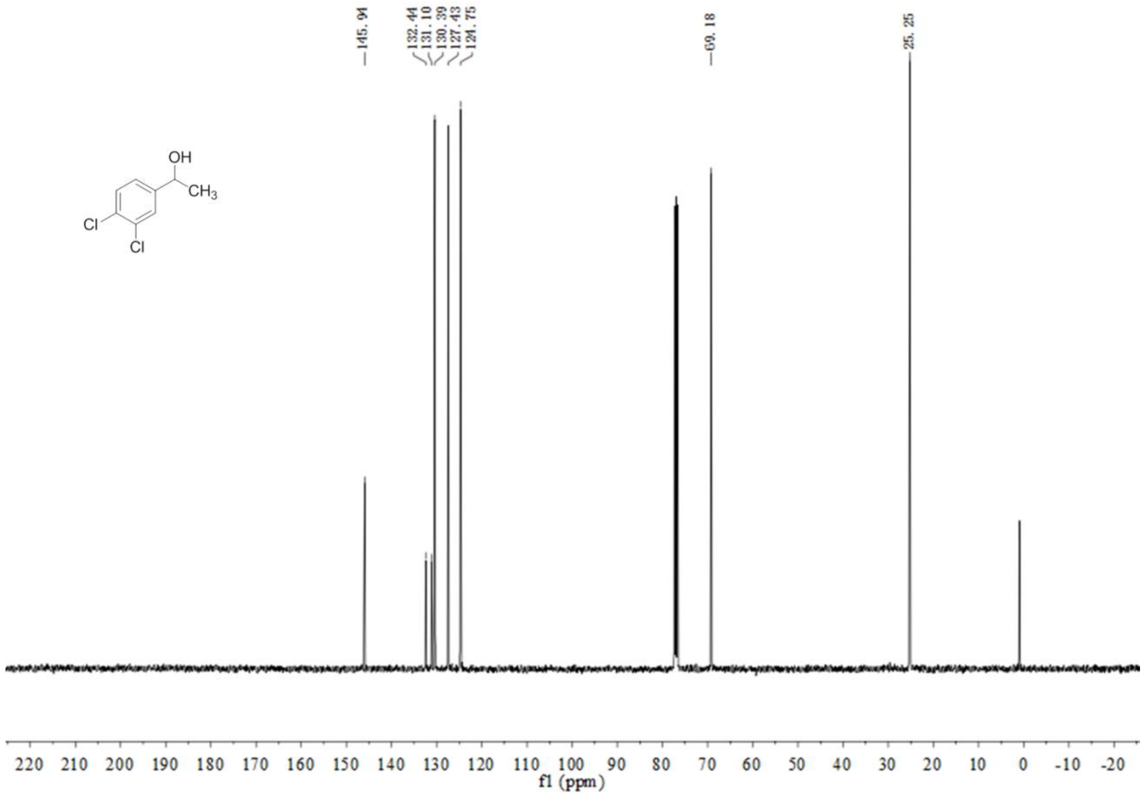

Figure S99. ^{13}C NMR spectrum for compound 1-(3,4-Dichlorophenyl)ethanol(**4f**).

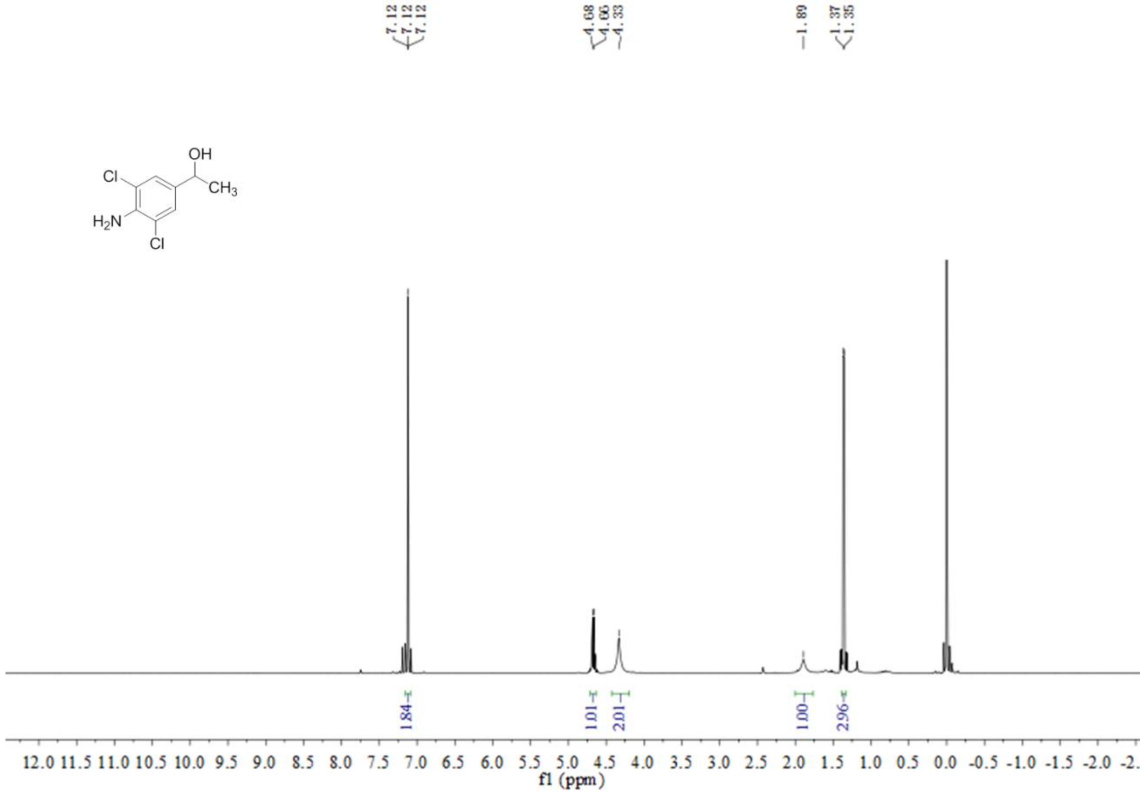

Figure S100. ^{1}H NMR spectrum for compound1-(4-Amino-3,5-dichlorophenyl)ethanol(**4g**).

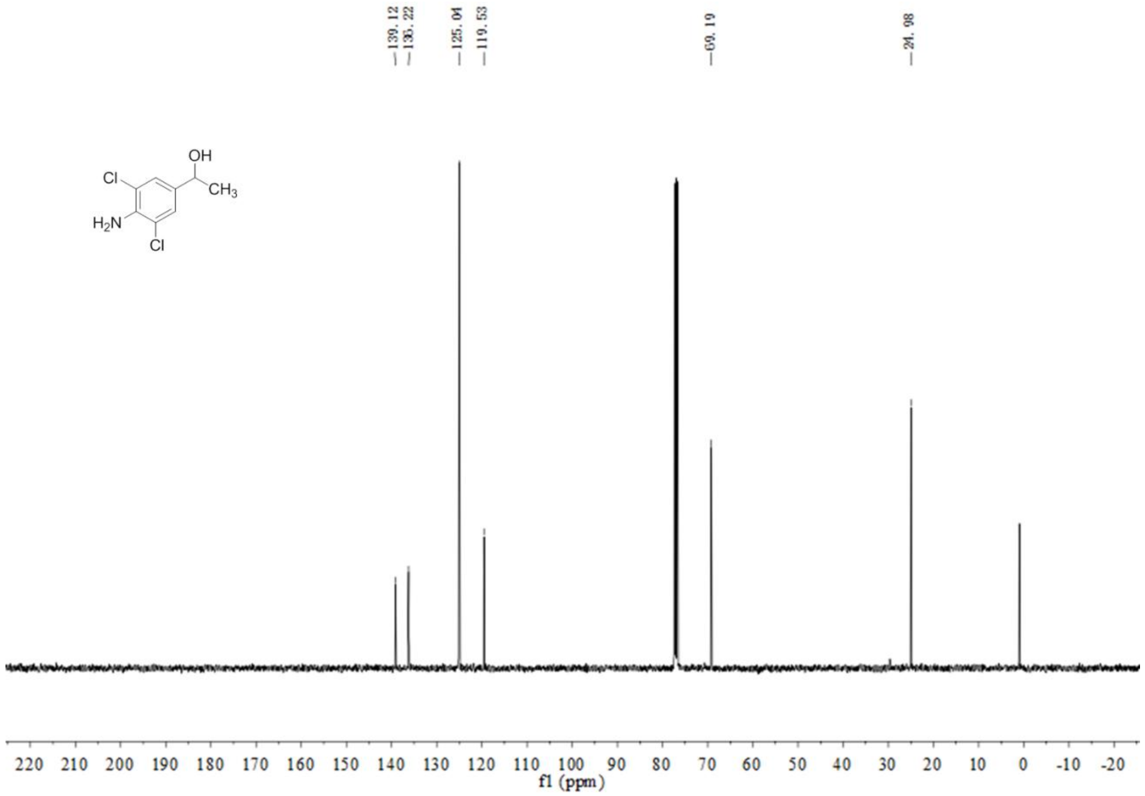

Figure S101. ^{13}C NMR spectrum for compound 1-(4-Amino-3,5-dichlorophenyl)ethanol(**4g**).

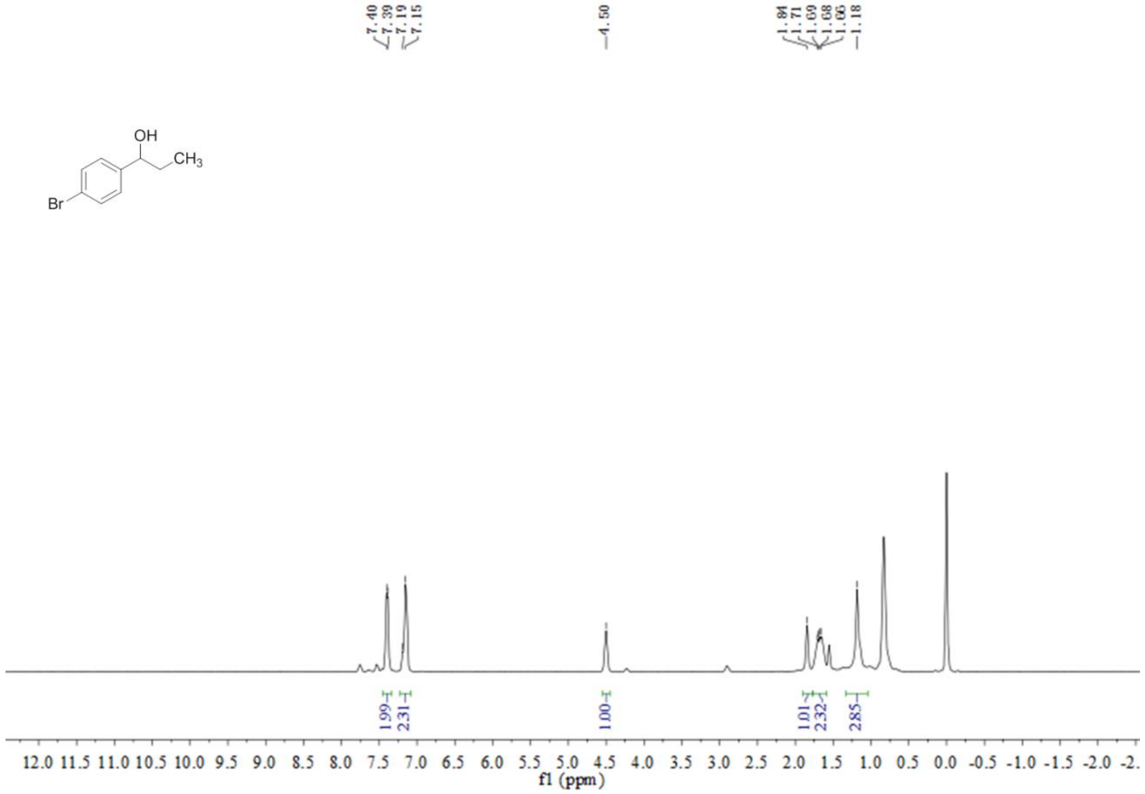

Figure S102. ^{1}H NMR spectrum for compound1-(4-bromophenyl)propan-1-ol (**4h**).

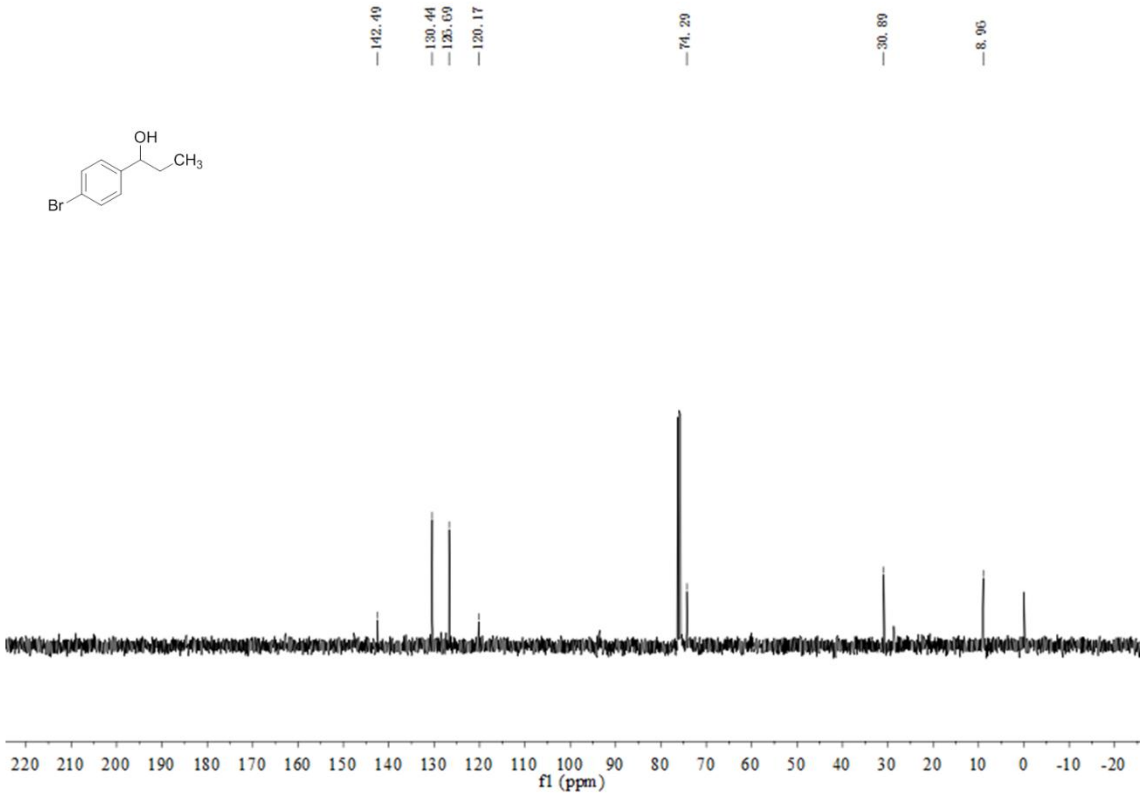

Figure S103. ^{13}C NMR spectrum for compound 1-(4-bromophenyl)propan-1-ol (**4h**).

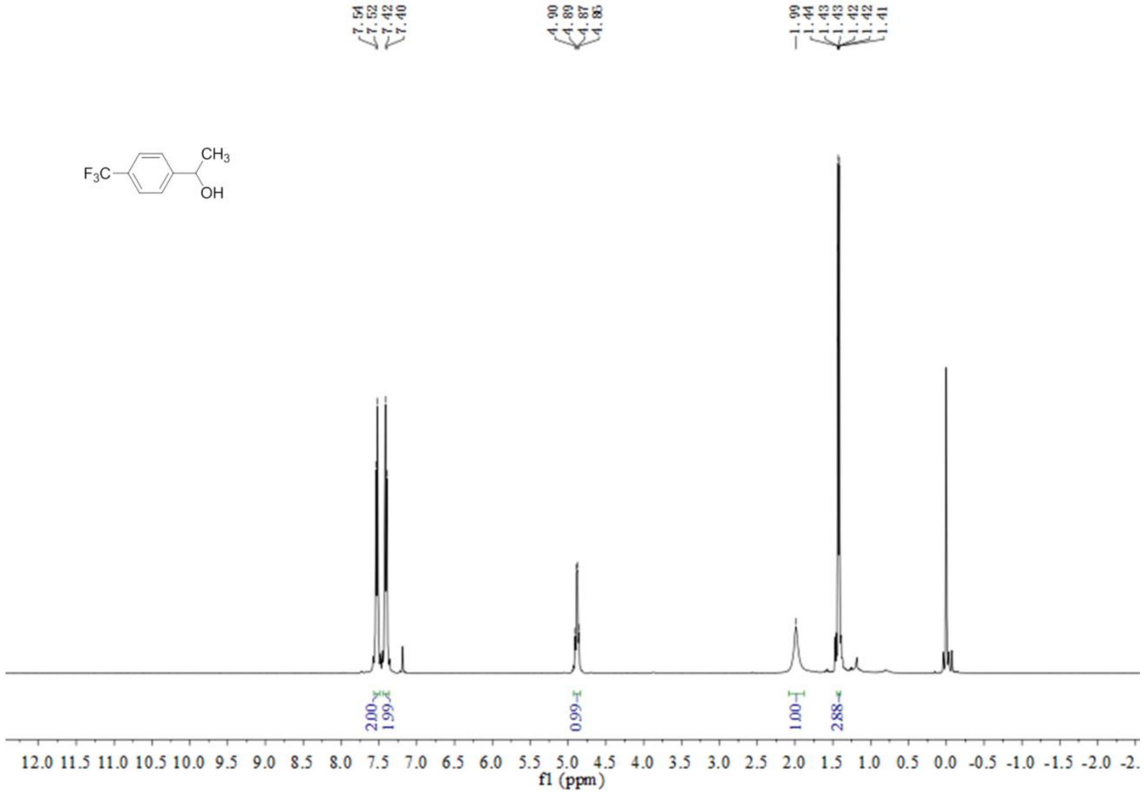

Figure S104. ^{1}H NMR spectrum for compound1-(4-(Trifluoromethyl)phenyl)ethanol(**4i**).

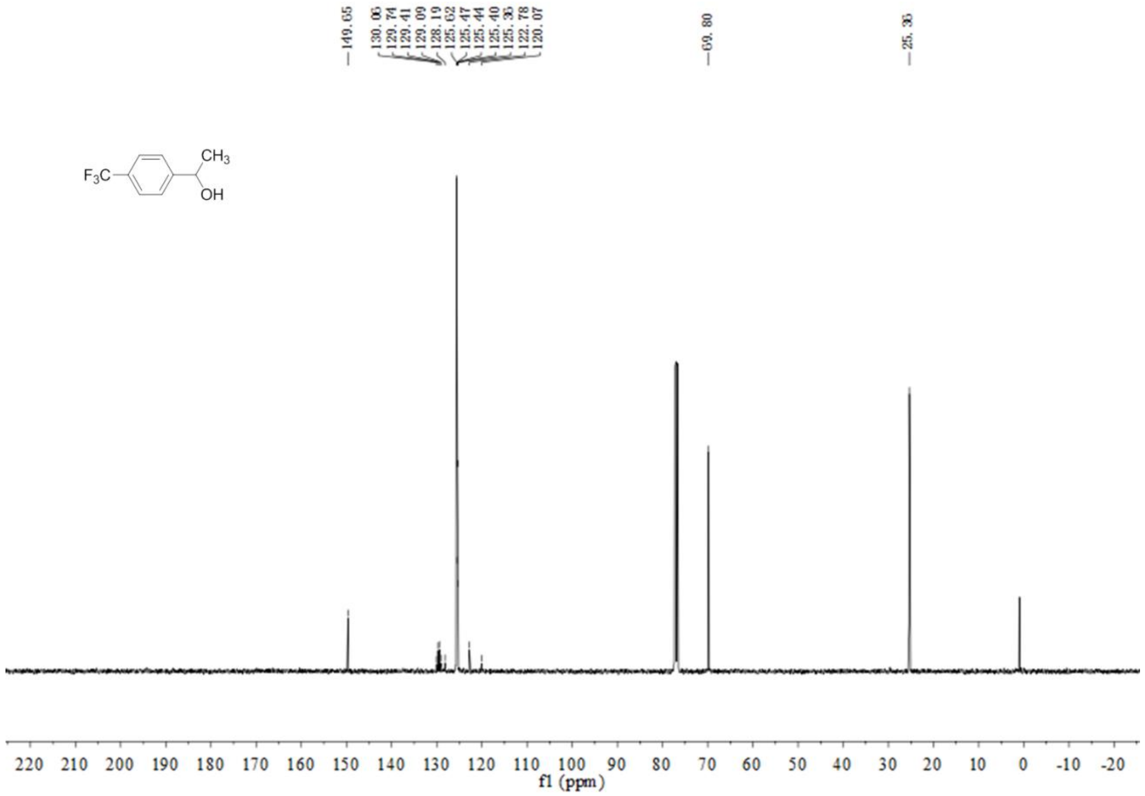

Figure S105. ^{13}C NMR spectrum for compound 1-(4-(Trifluoromethyl)phenyl)ethanol(**4i**).

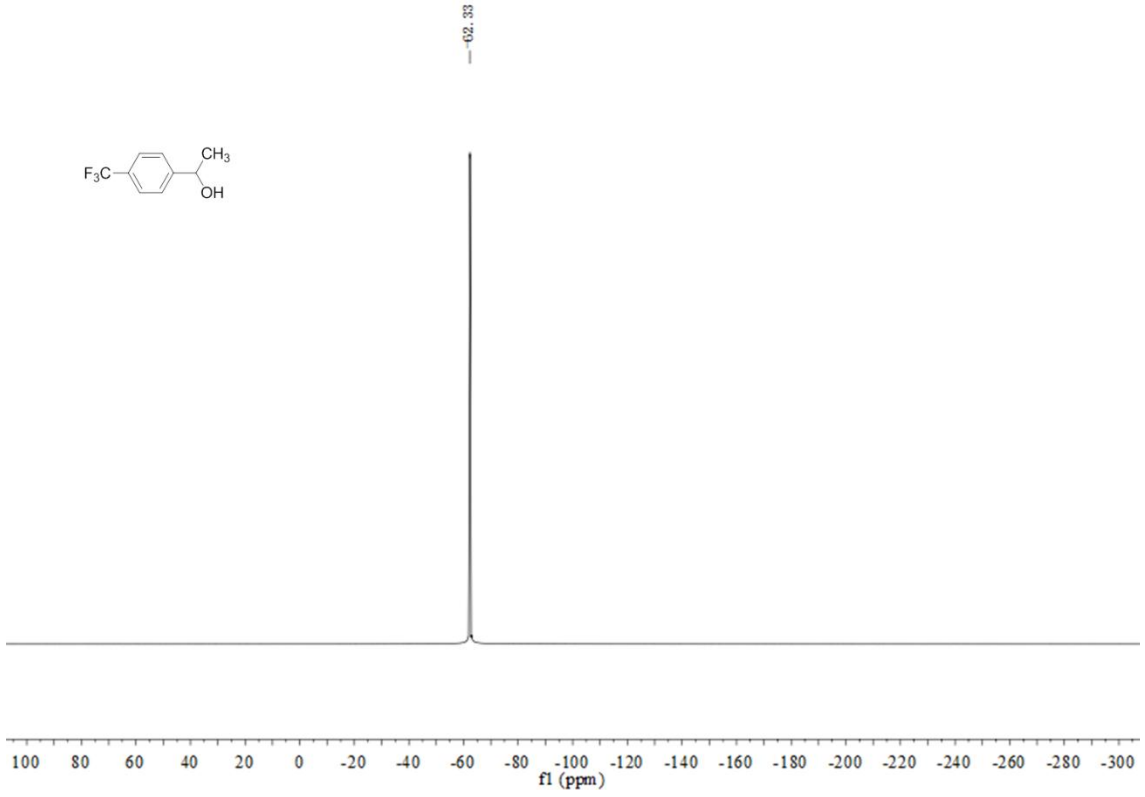

Figure S106. ^{19}F NMR spectrum for compound 1-(4-(Trifluoromethyl)phenyl)ethanol(**4i**).

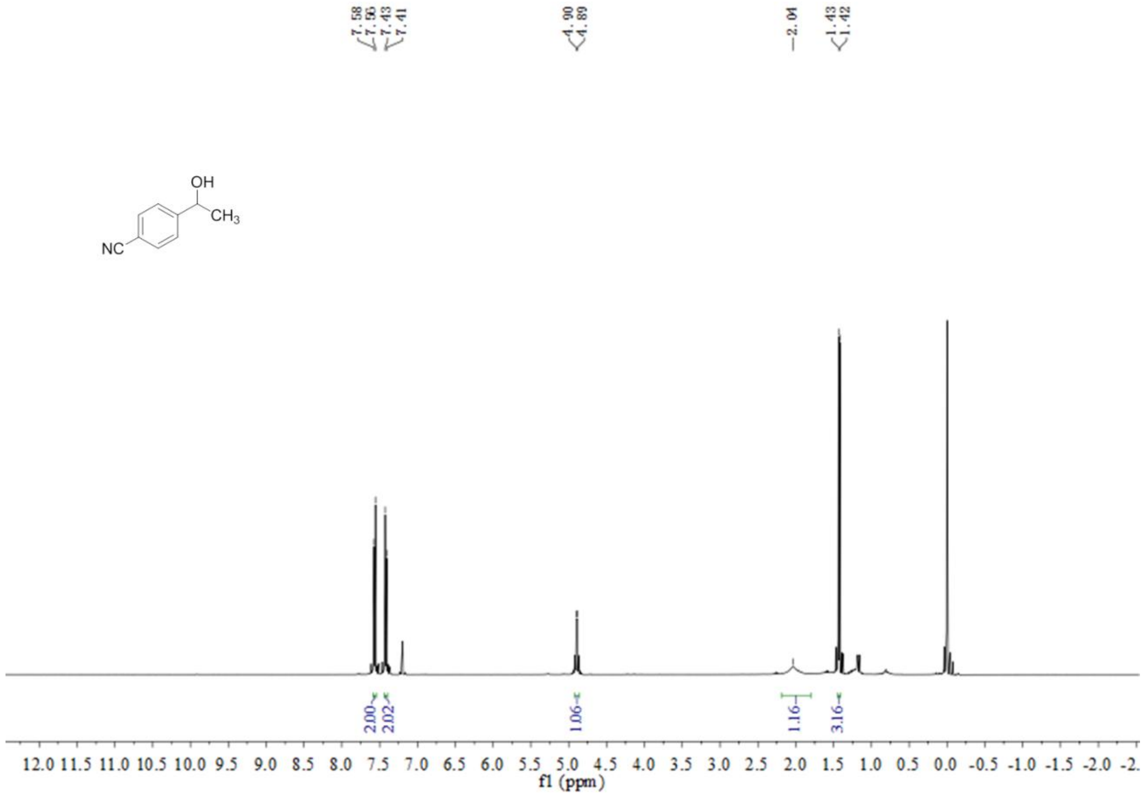

Figure S107. ¹H NMR spectrum for compound 4-(1-Hydroxyethyl)benzonitrile(**4j**).

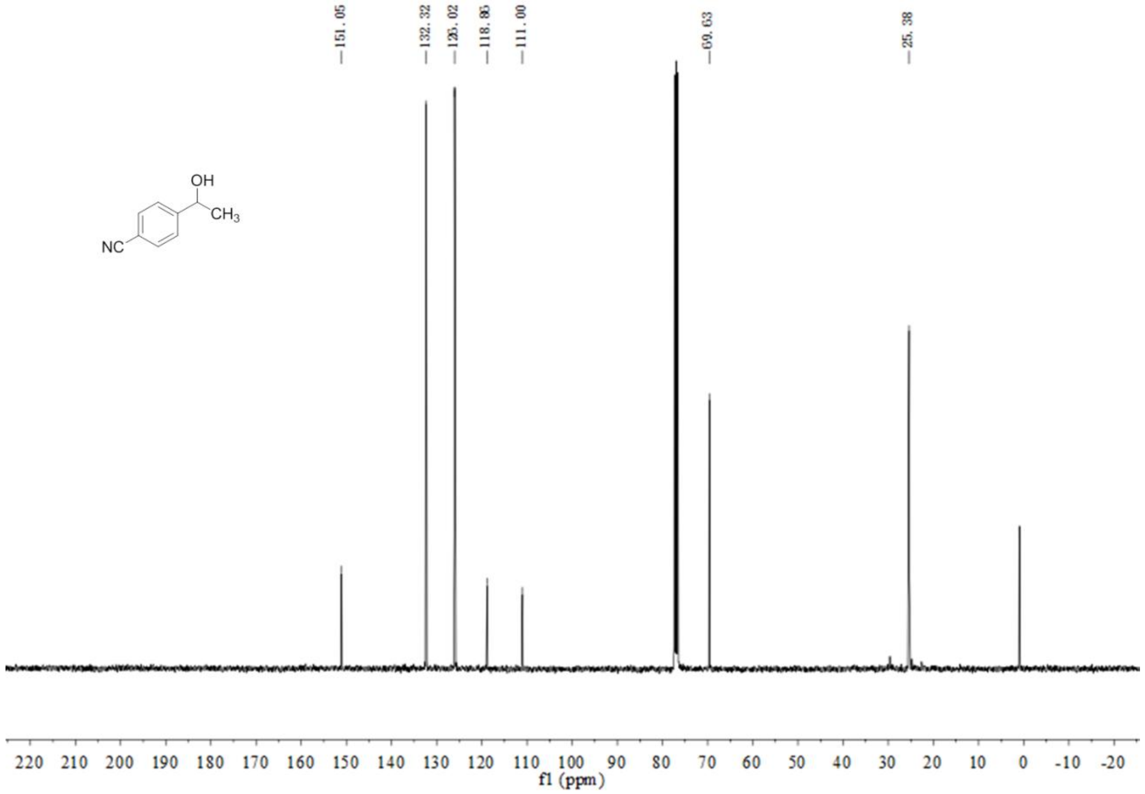

Figure S108. ¹³C NMR spectrum for compound 4-(1-Hydroxyethyl)benzonitrile(**4j**).

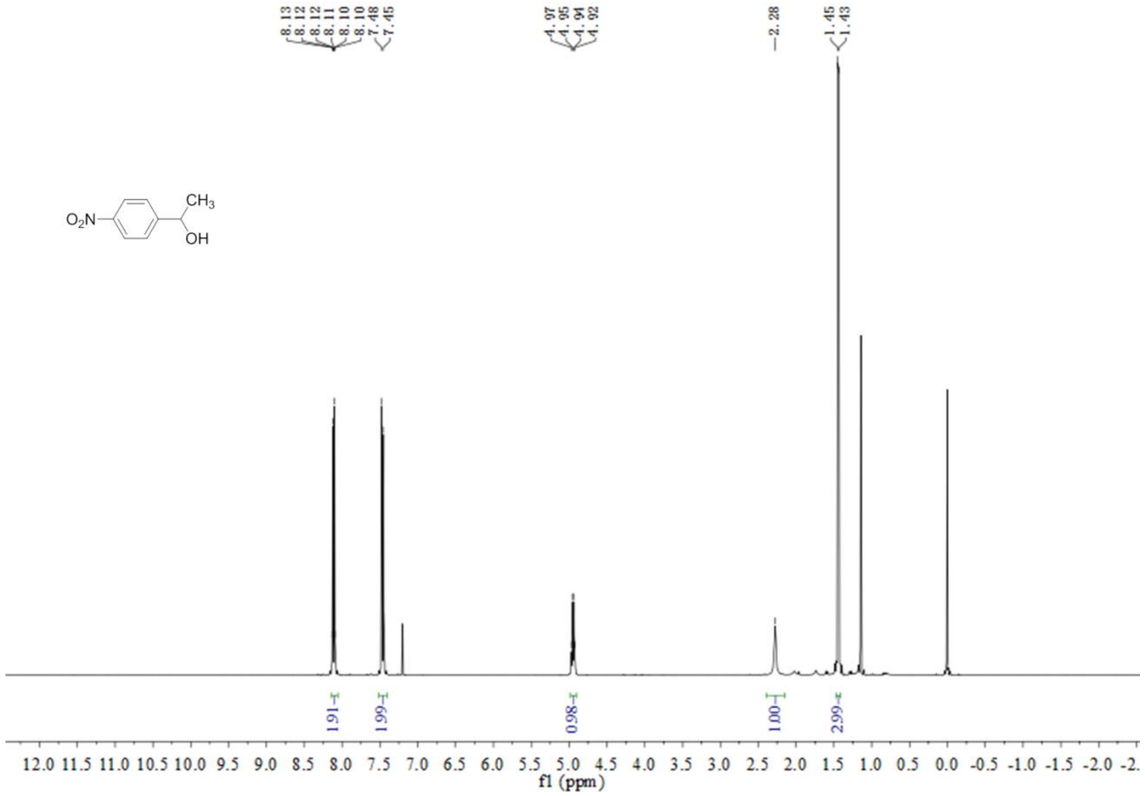

Figure S109. ^{1}H NMR spectrum for compound 1-(4-Nitrophenyl)ethanol(**4k**).

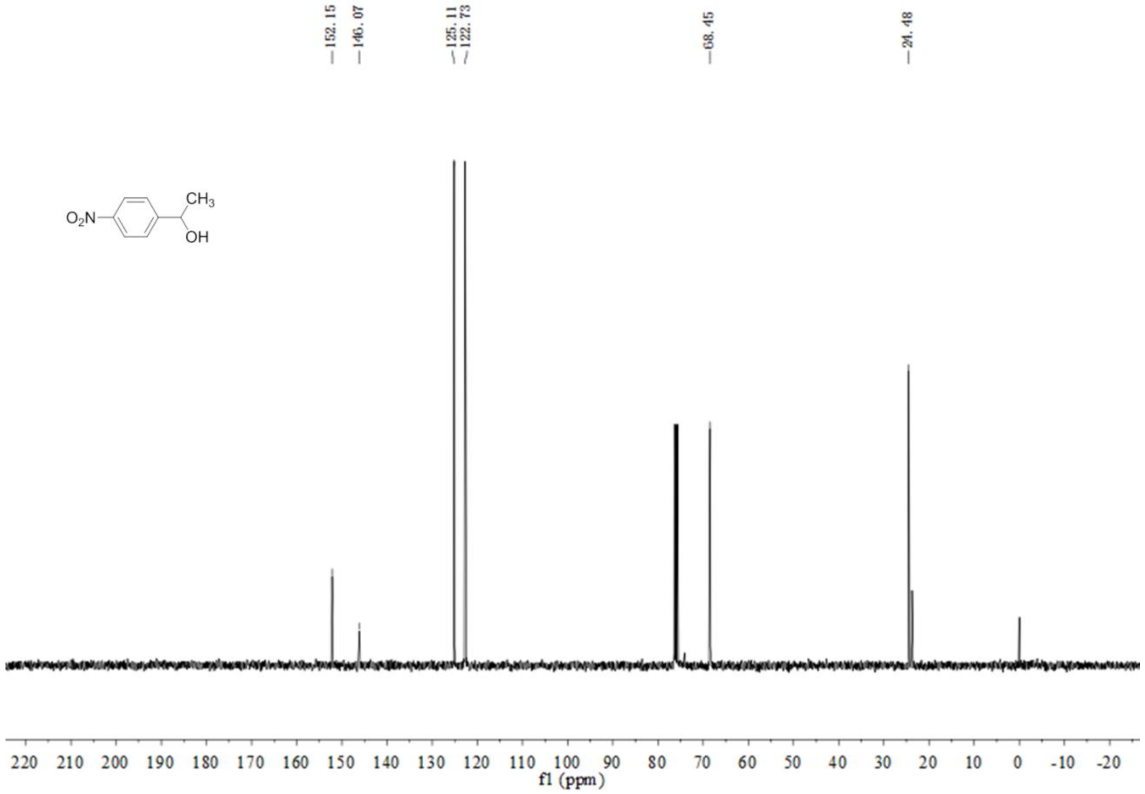

Figure S110. ^{13}C NMR spectrum for compound 1-(4-Nitrophenyl)ethanol(**4k**).

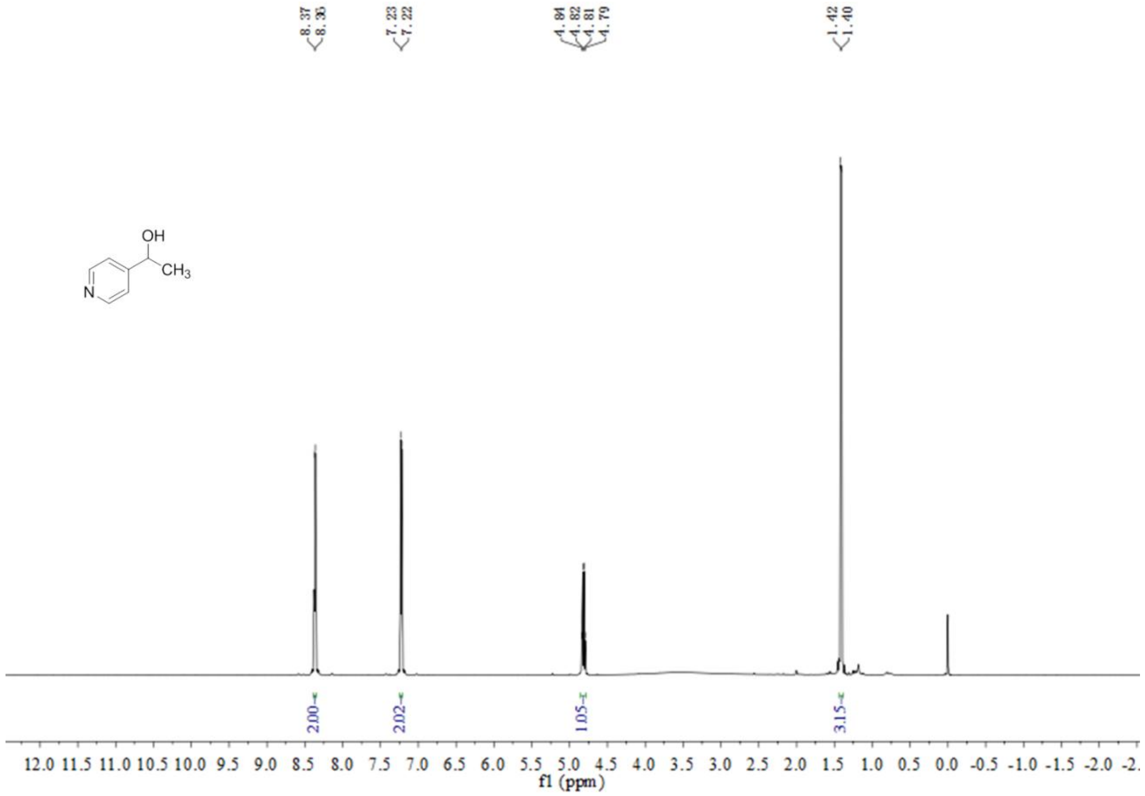

Figure S111. ^{1}H NMR spectrum for compound 1-(Pyridin-4-yl)ethanol(**4i**).

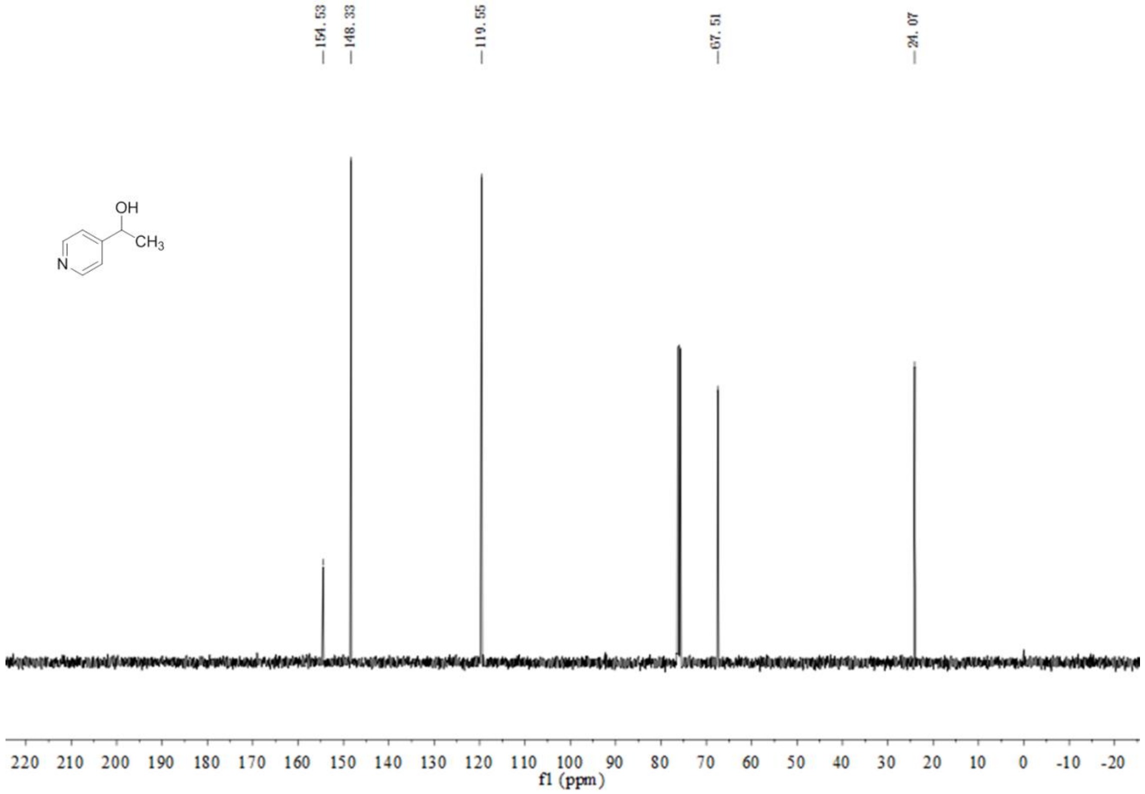

Figure S112. ^{13}C NMR spectrum for compound 1-(Pyridin-4-yl)ethanol(**4i**).

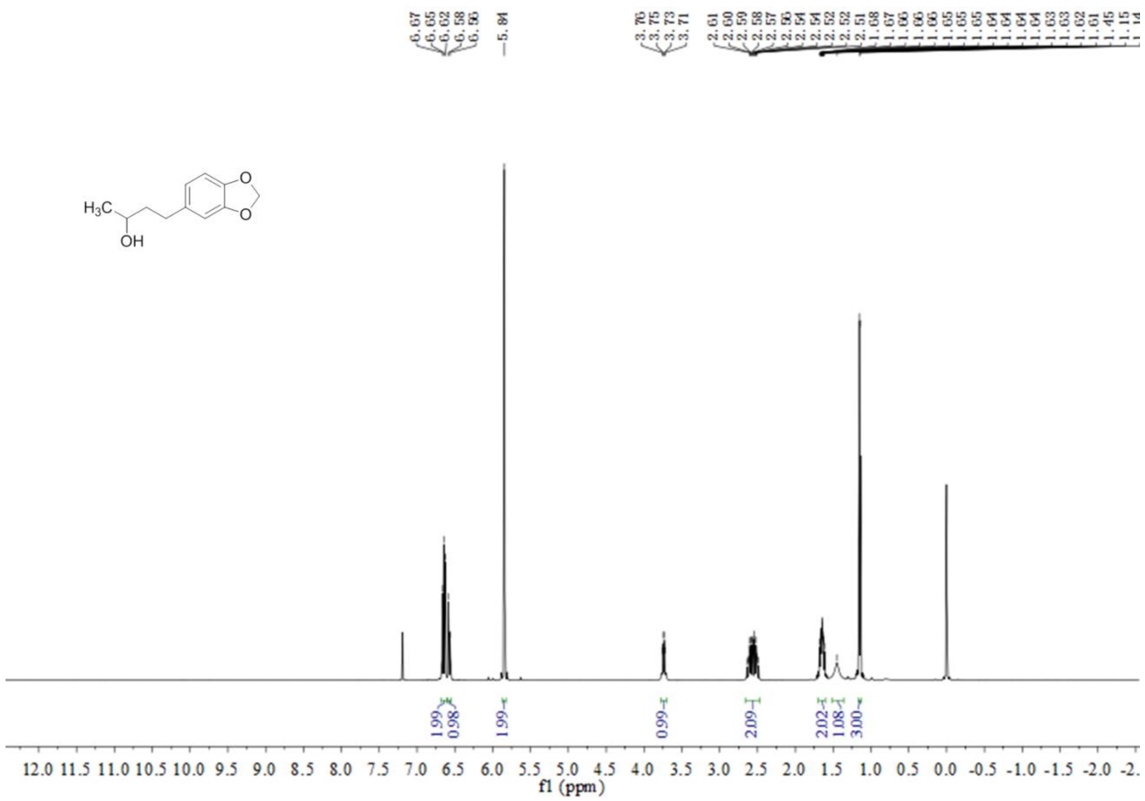

Figure S113. ^{1}H NMR spectrum for compound 4-(benzo[d][1,3]dioxol-5-yl)butan-2-ol(**4m**).

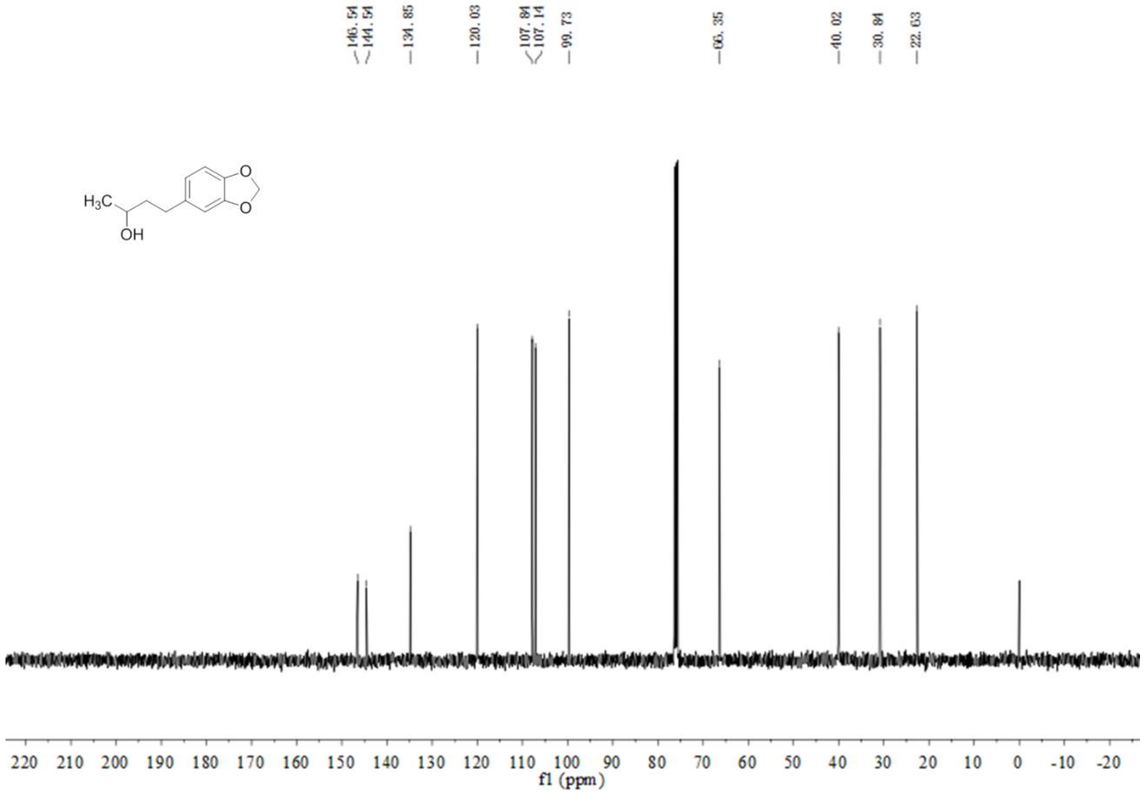

Figure S114. ^{13}C NMR spectrum for compound 4-(benzo[d][1,3]dioxol-5-yl)butan-2-ol(**4m**).

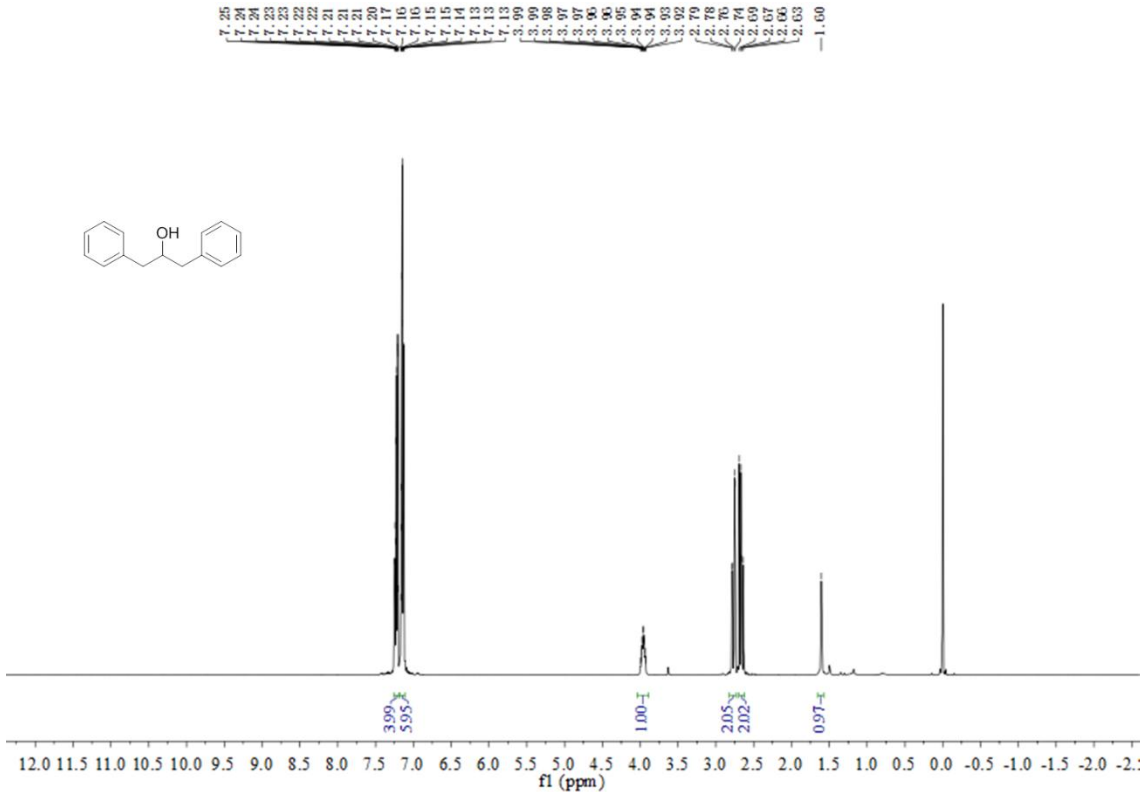

Figure S115. ^{1}H NMR spectrum for compound 1,3-Diphenylpropan-2-ol(**4n**).

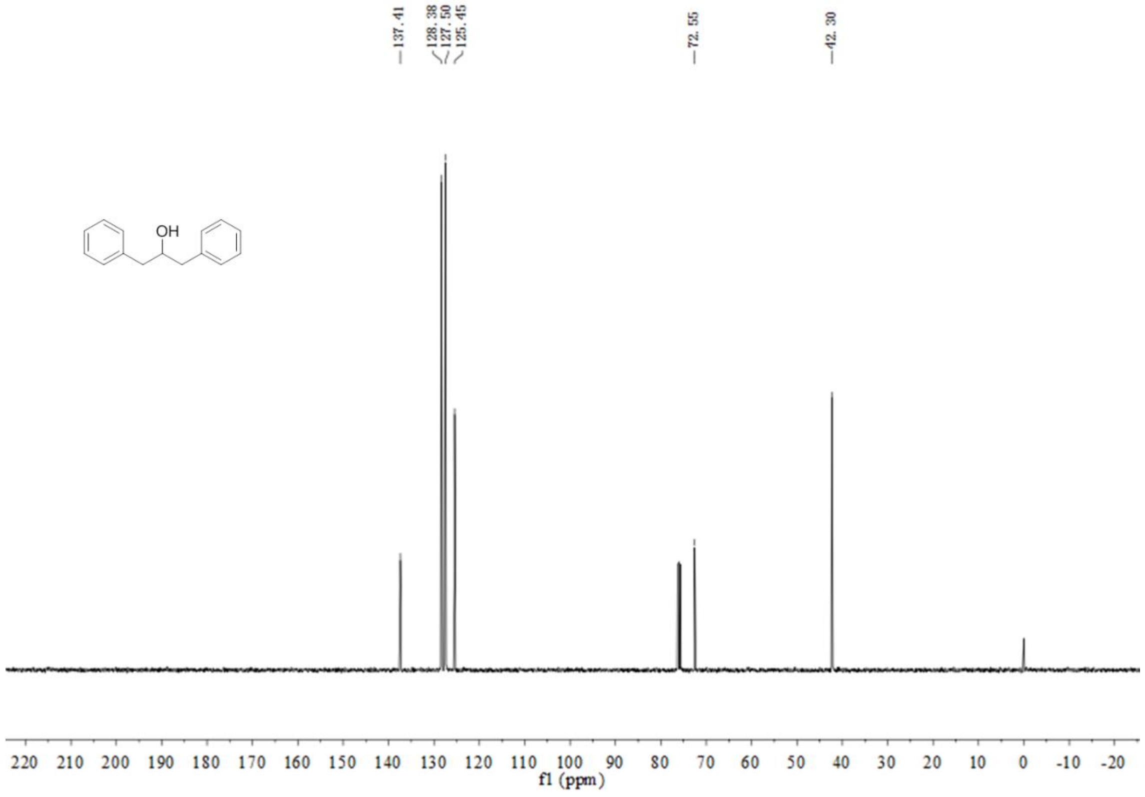

Figure S116. ^{13}C NMR spectrum for compound 1,3-Diphenylpropan-2-ol(**4n**).

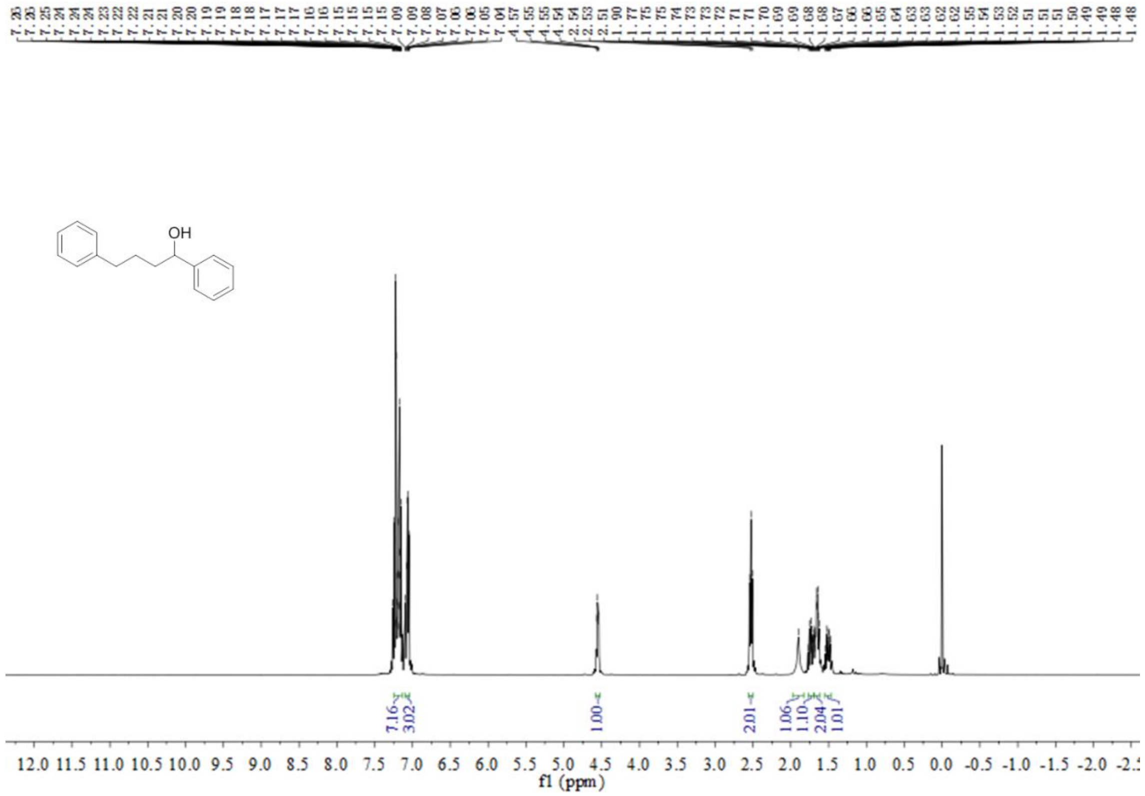

Figure S117. ^{1}H NMR spectrum for compound 1,4-Diphenylbutan-1-ol(**4o**).

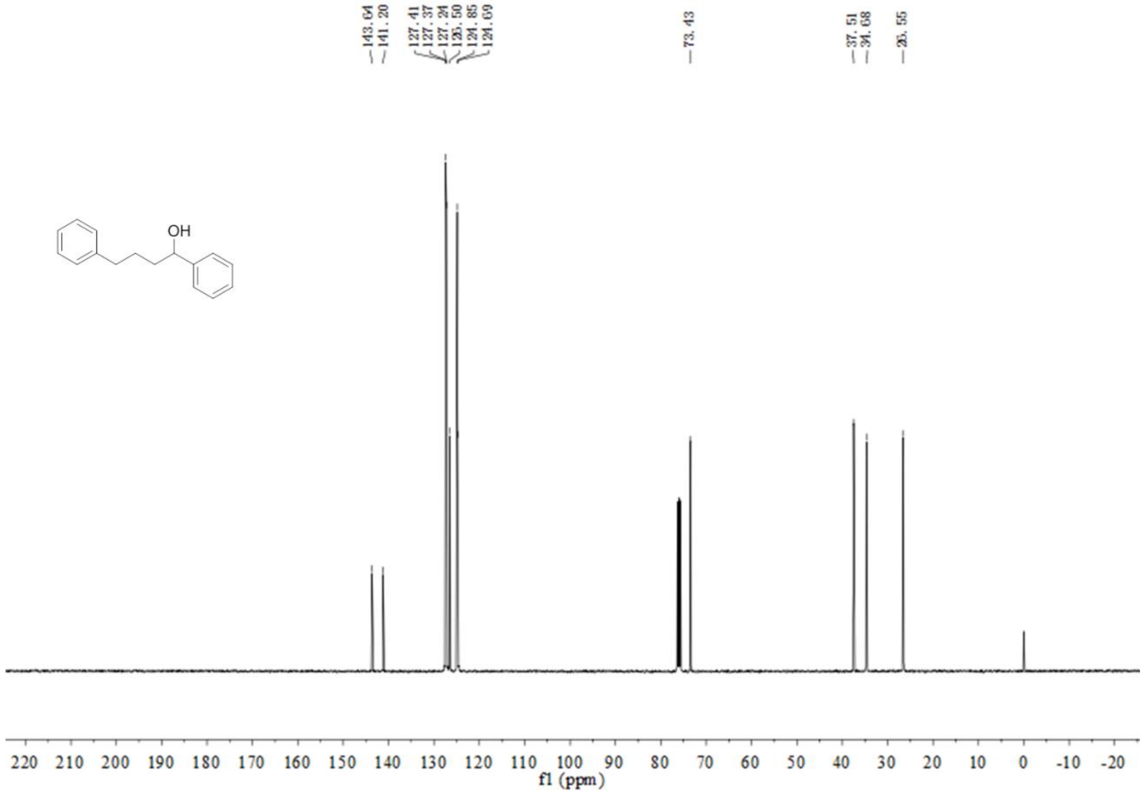

Figure S118. ^{13}C NMR spectrum for compound 1,4-Diphenylbutan-1-ol(**4o**).

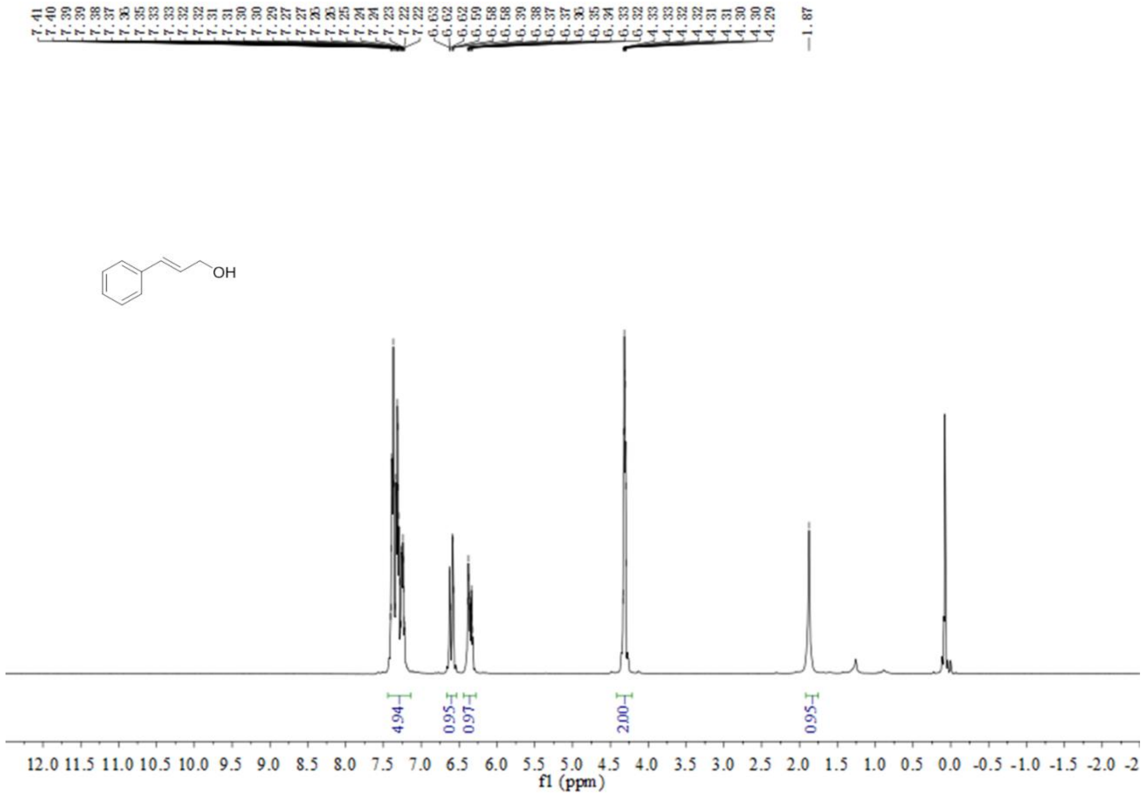

Figure S119. ^{1}H NMR spectrum for compound (E)-3-phenylprop-2-en-1-ol(**6a**).

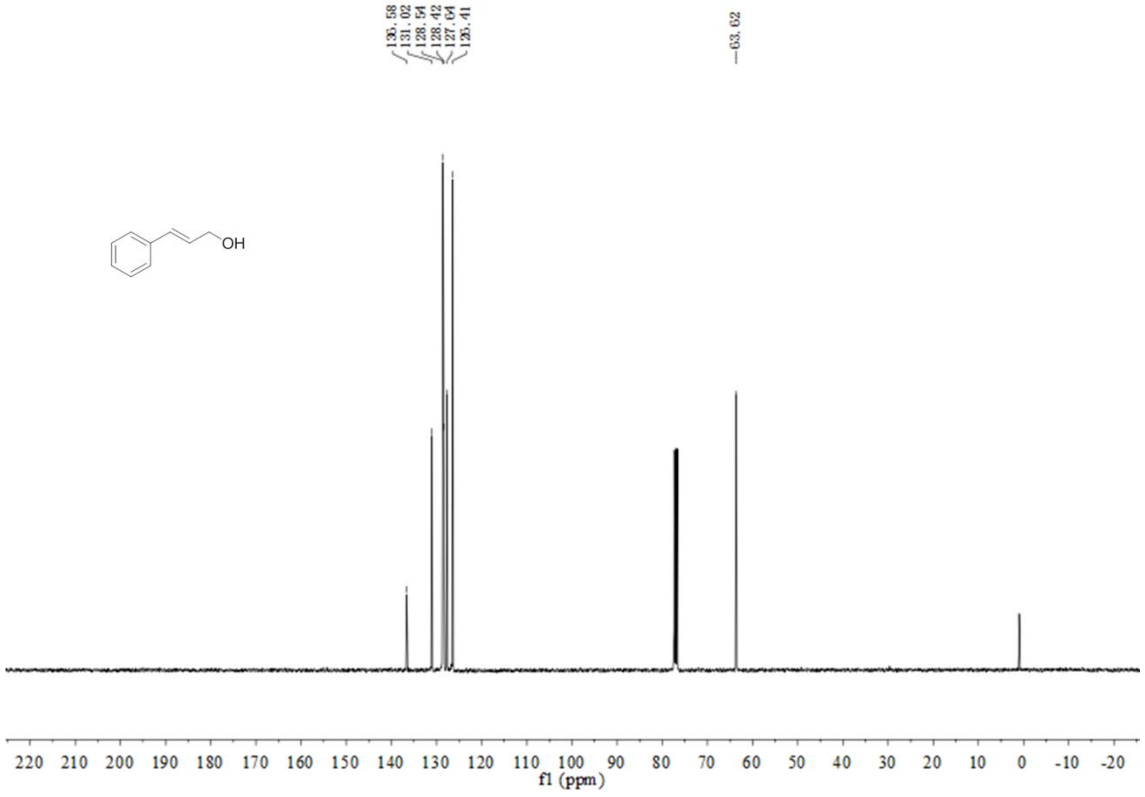

Figure S120. ^{13}C NMR spectrum for compound (E)-3-phenylprop-2-en-1-ol(**6a**).

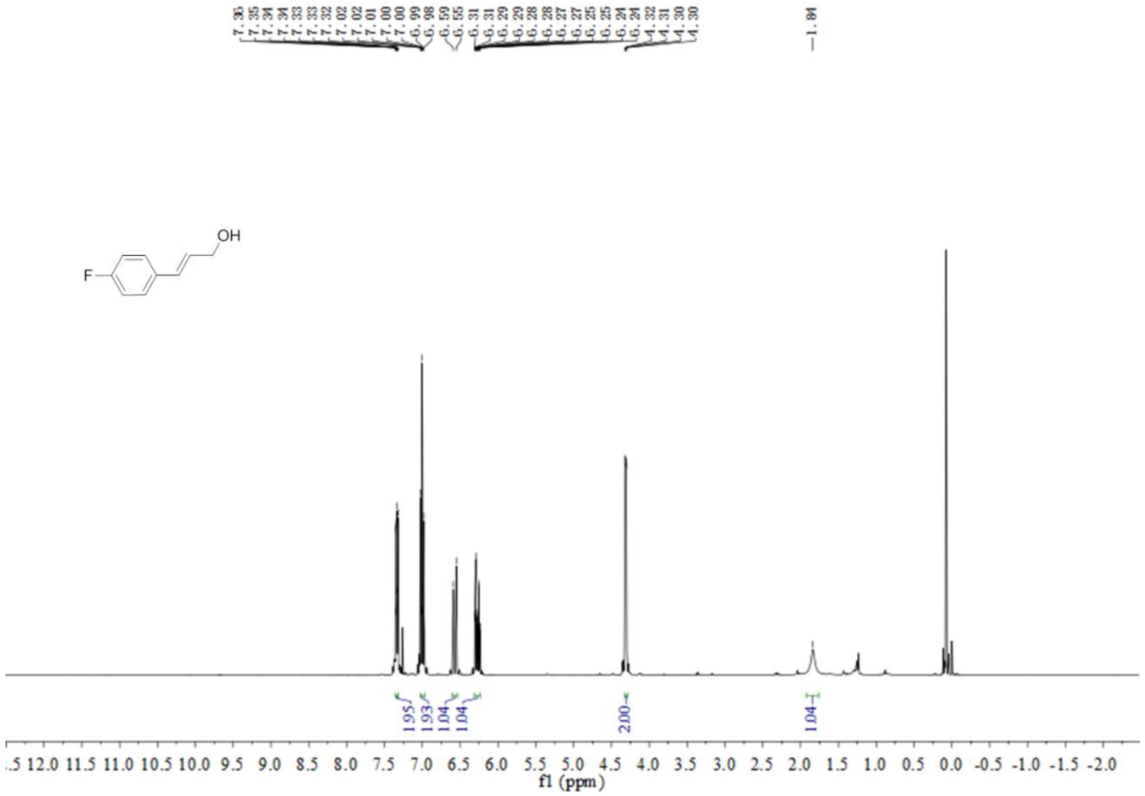

Figure S121. ^{1}H NMR spectrum for compound (E)-3-(4-fluorophenyl)prop-2-en-1-ol(**6b**).

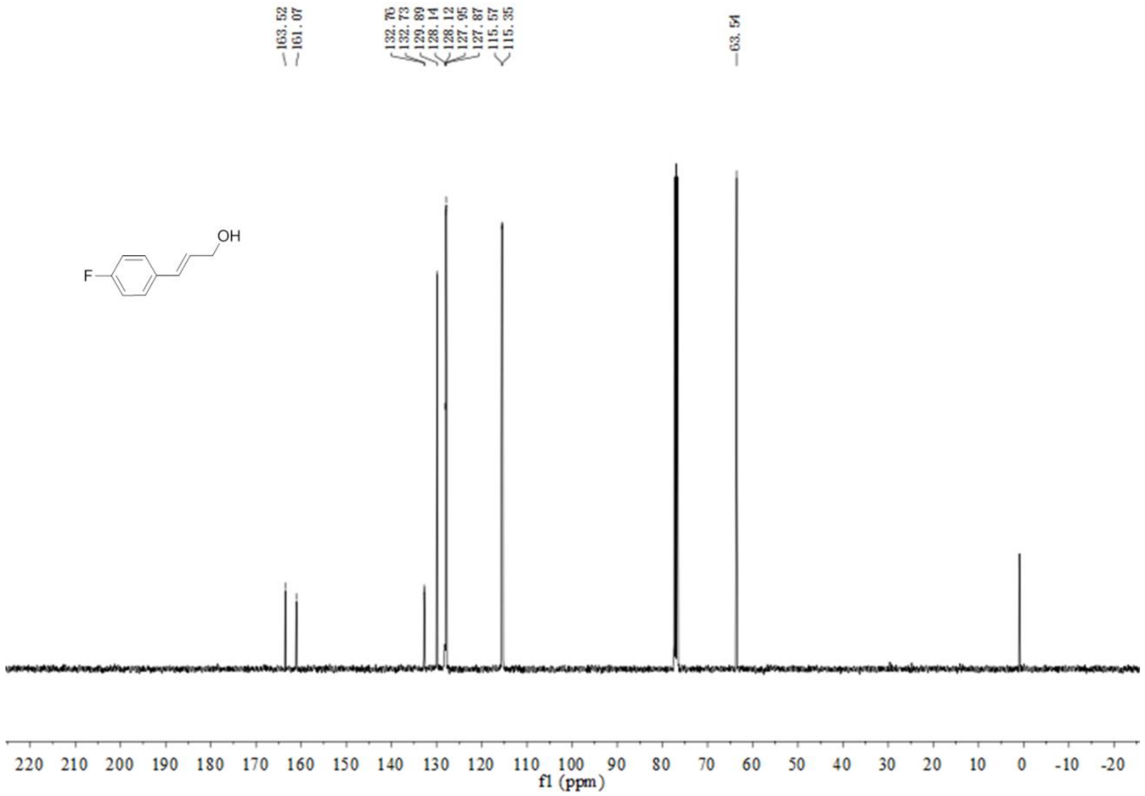

Figure S122. ^{13}C NMR spectrum for compound (E)-3-(4-fluorophenyl)prop-2-en-1-ol(**6b**).

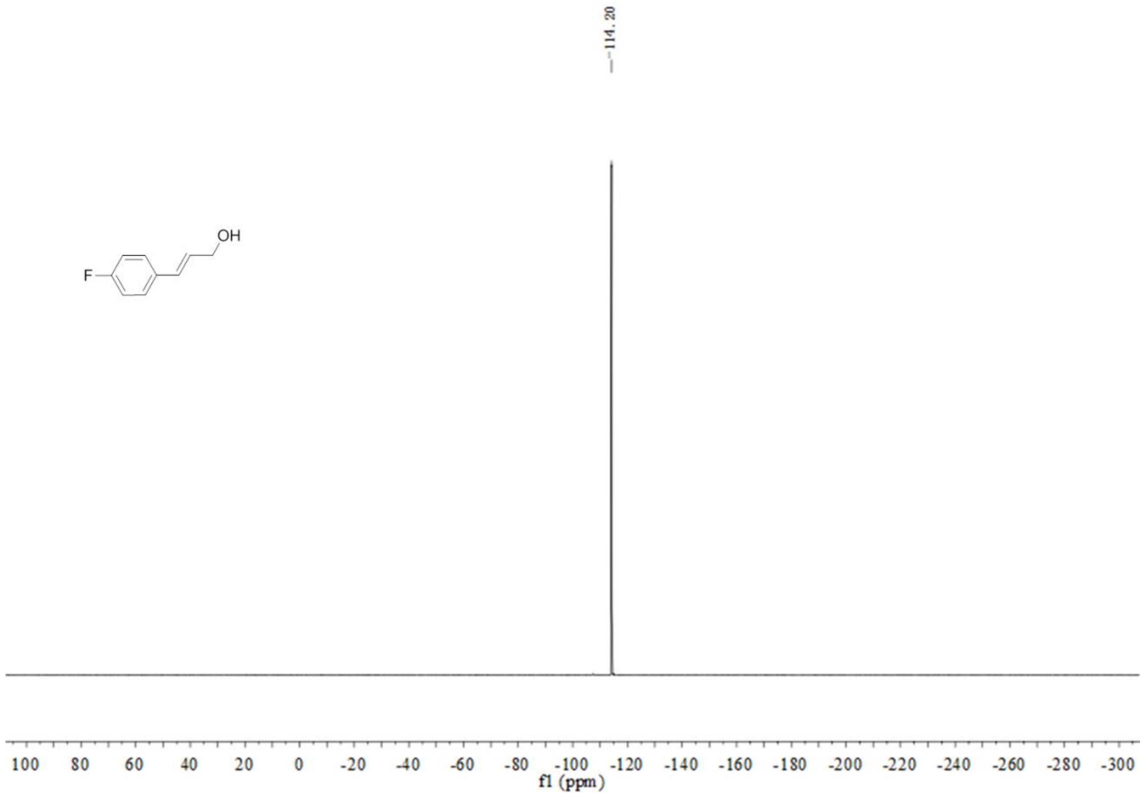

Figure S123. ^{13}C NMR spectrum for compound (E)-3-(4-fluorophenyl)prop-2-en-1-ol(**6b**).

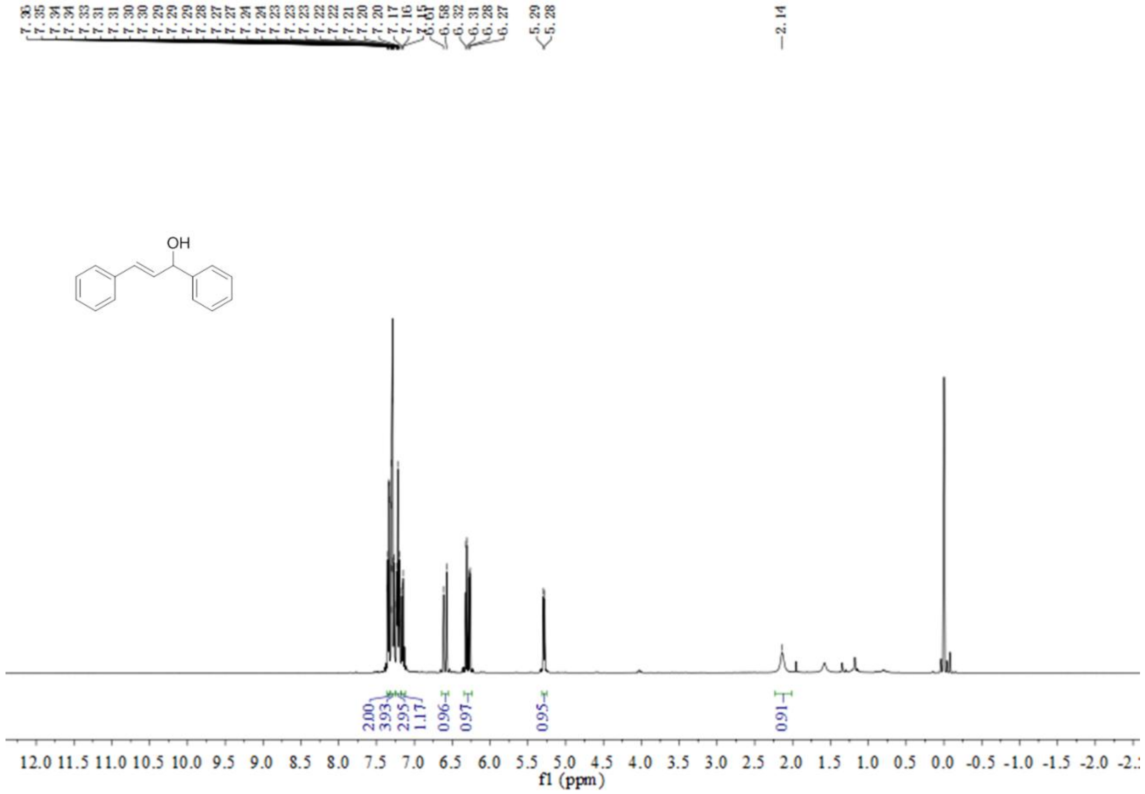

Figure S124. ^{1}H NMR spectrum for compound (E)-1,3-diphenylprop-2-en-1-ol (**6c**).

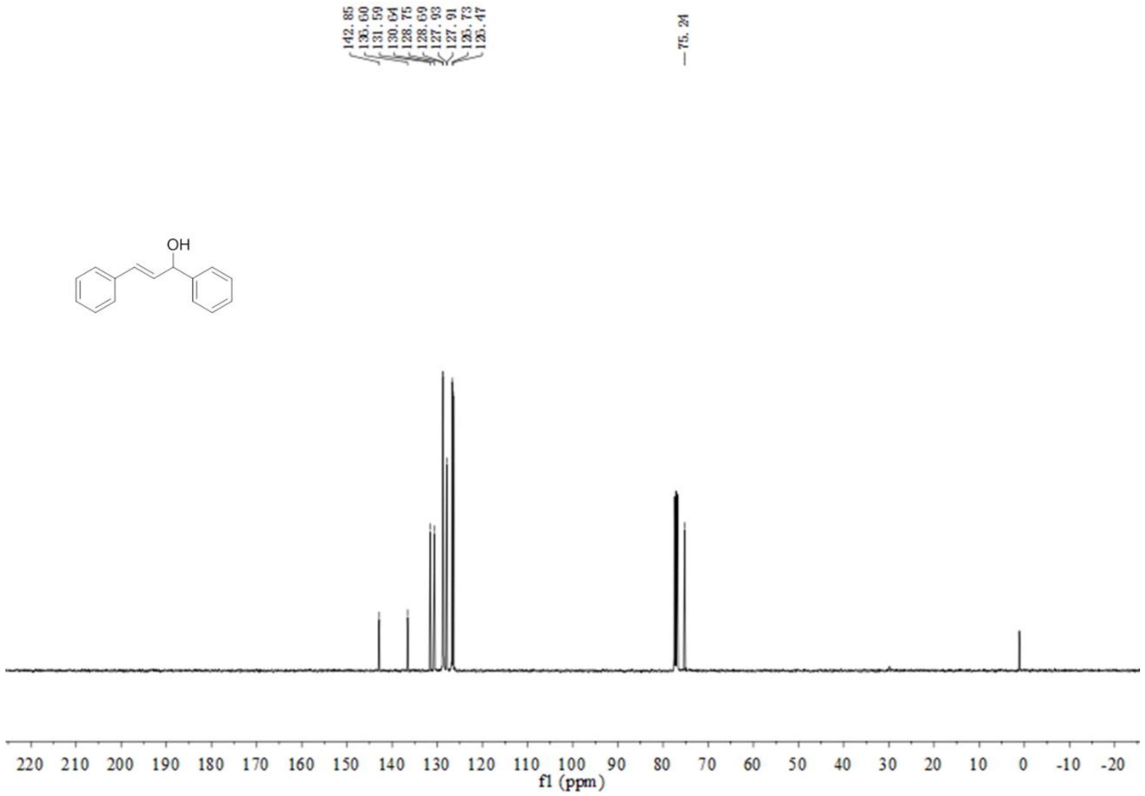

Figure S125. ^{13}C NMR spectrum for compound (E)-1,3-diphenylprop-2-en-1-ol (**6c**).

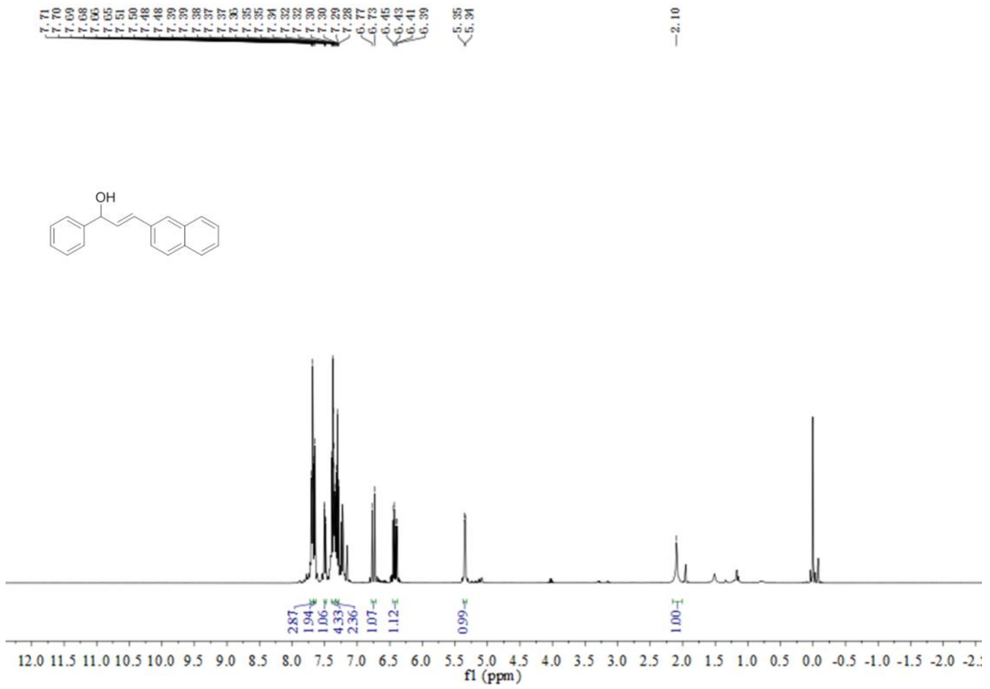

Figure S126. ^{1}H NMR spectrum for compound (E)-3-(naphthalen-2-yl)-1-phenylprop-2-en-1-ol(**6d**).

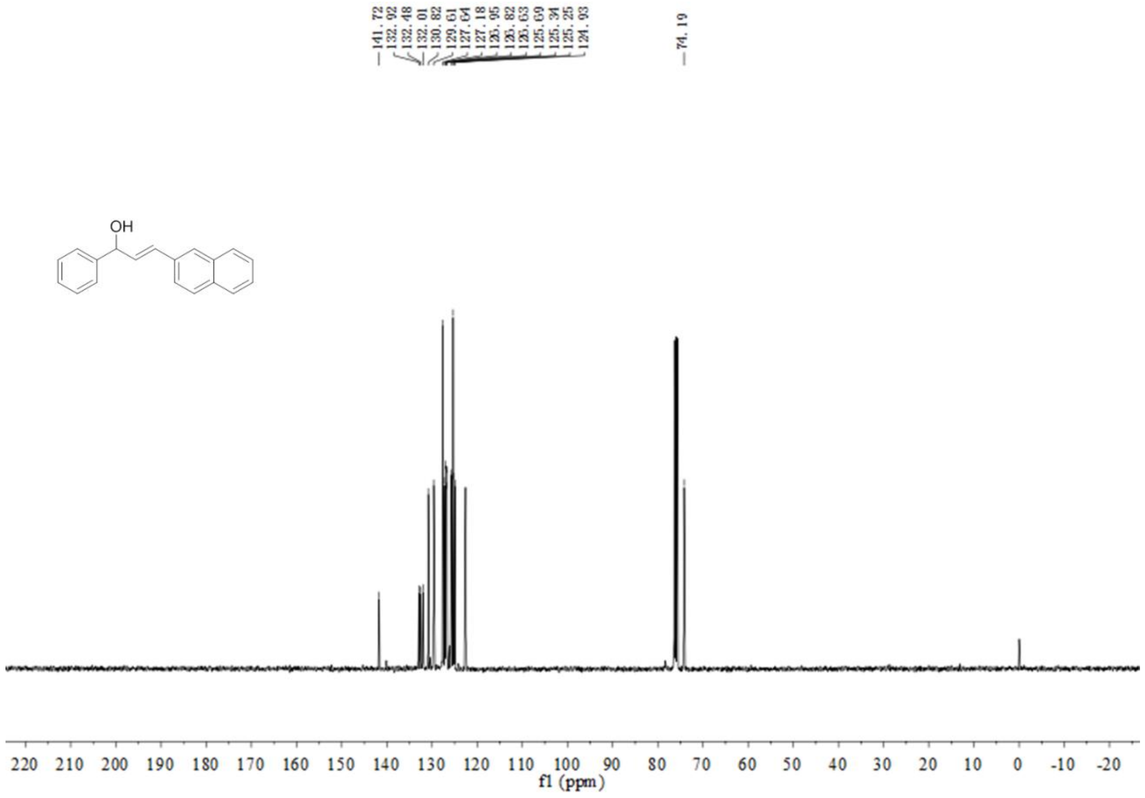

Figure S127. ^{13}C NMR spectrum for compound (E)-3-(naphthalen-2-yl)-1-phenylprop-2-en-1-ol(**6d**)

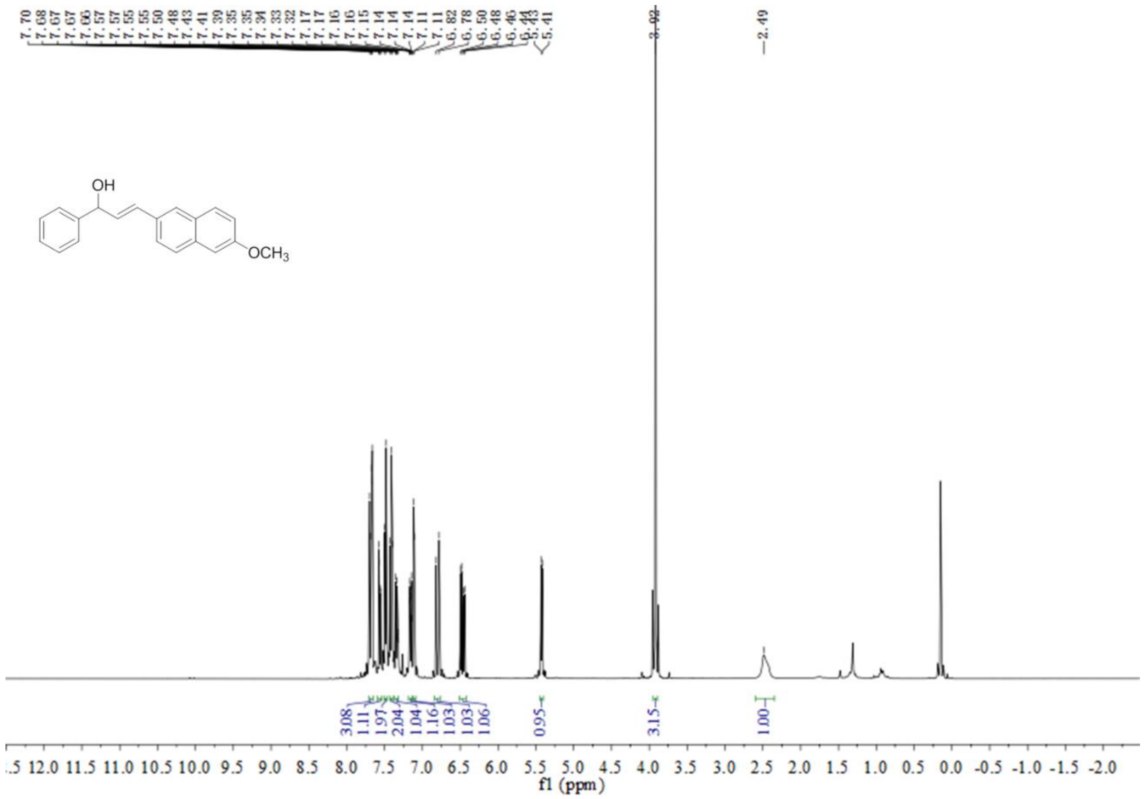

Figure S128. ^{1}H NMR spectrum for compound (E)-3-(6-methoxynaphthalen-2-yl)-1-phenylprop-2-en-1-ol(**6e**).

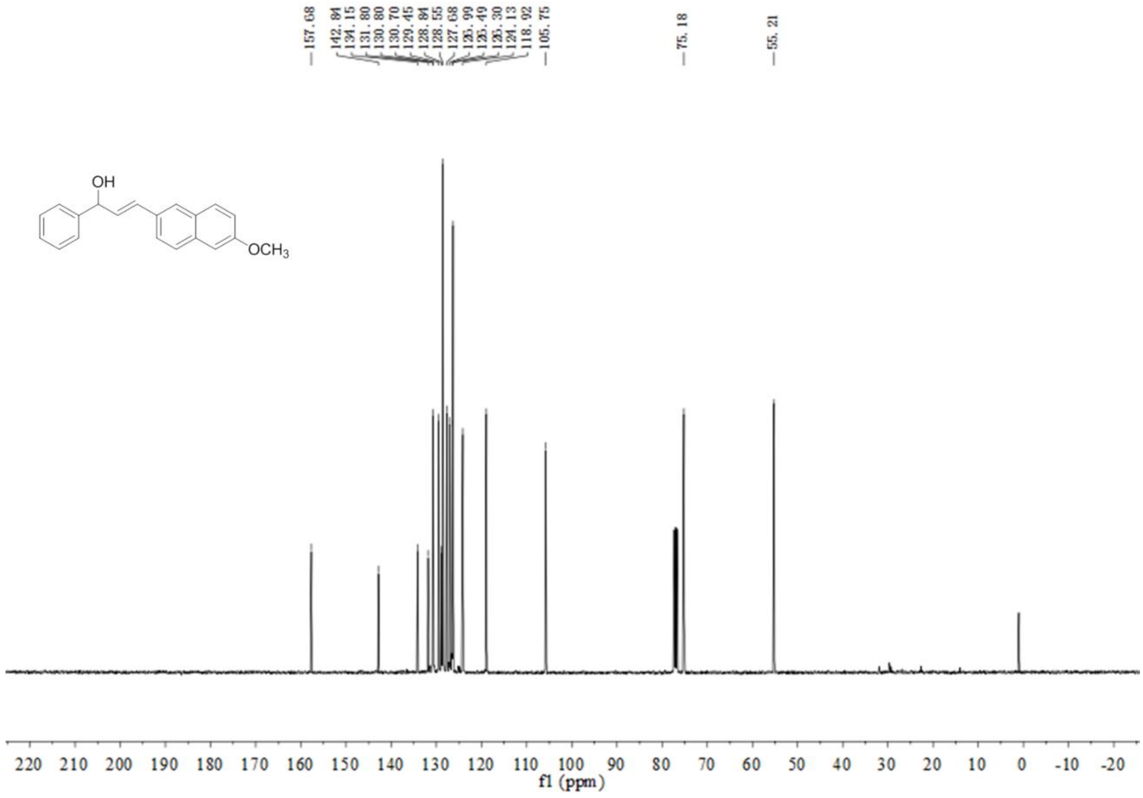

Figure S129. ^{13}C NMR spectrum for compound (E)-3-(6-methoxynaphthalen-2-yl)-1-phenylprop-2-en-1-ol(**6e**)

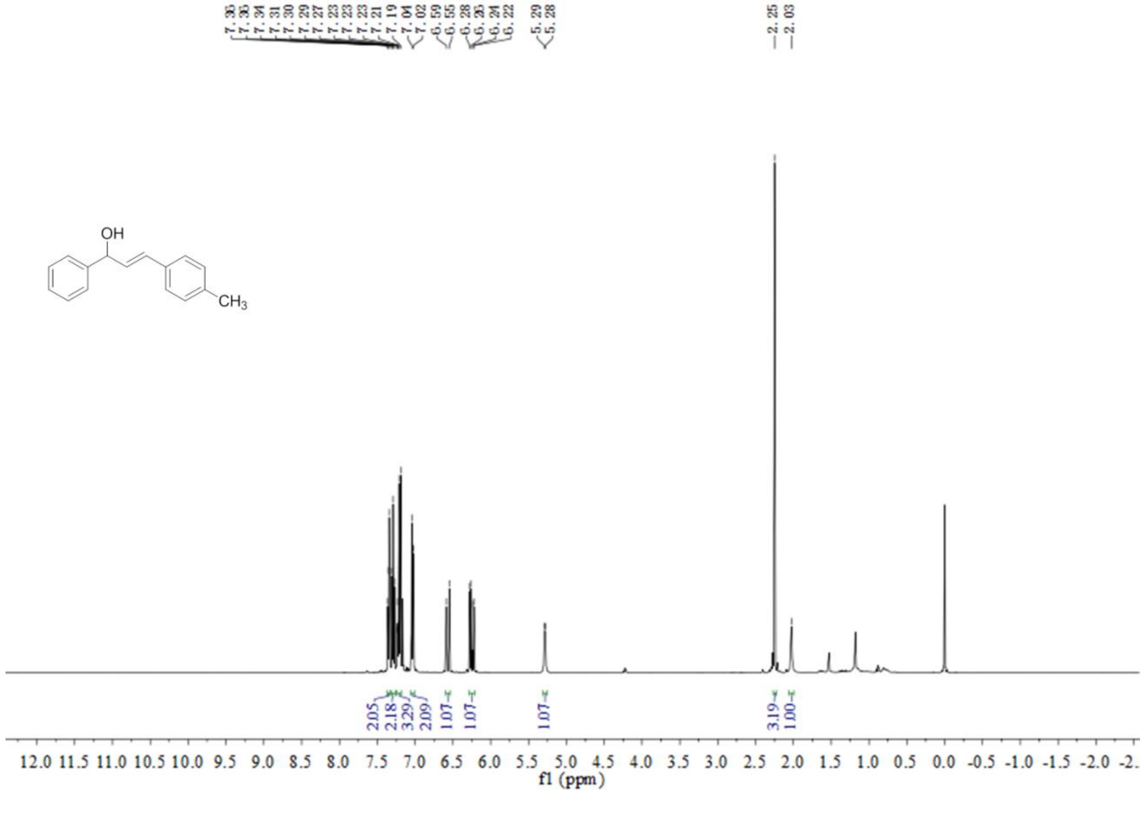

Figure S130. ^{1}H NMR spectrum for compound (E)-1-phenyl-3-(p-tolyl)prop-2-en-1-ol(**6f**).

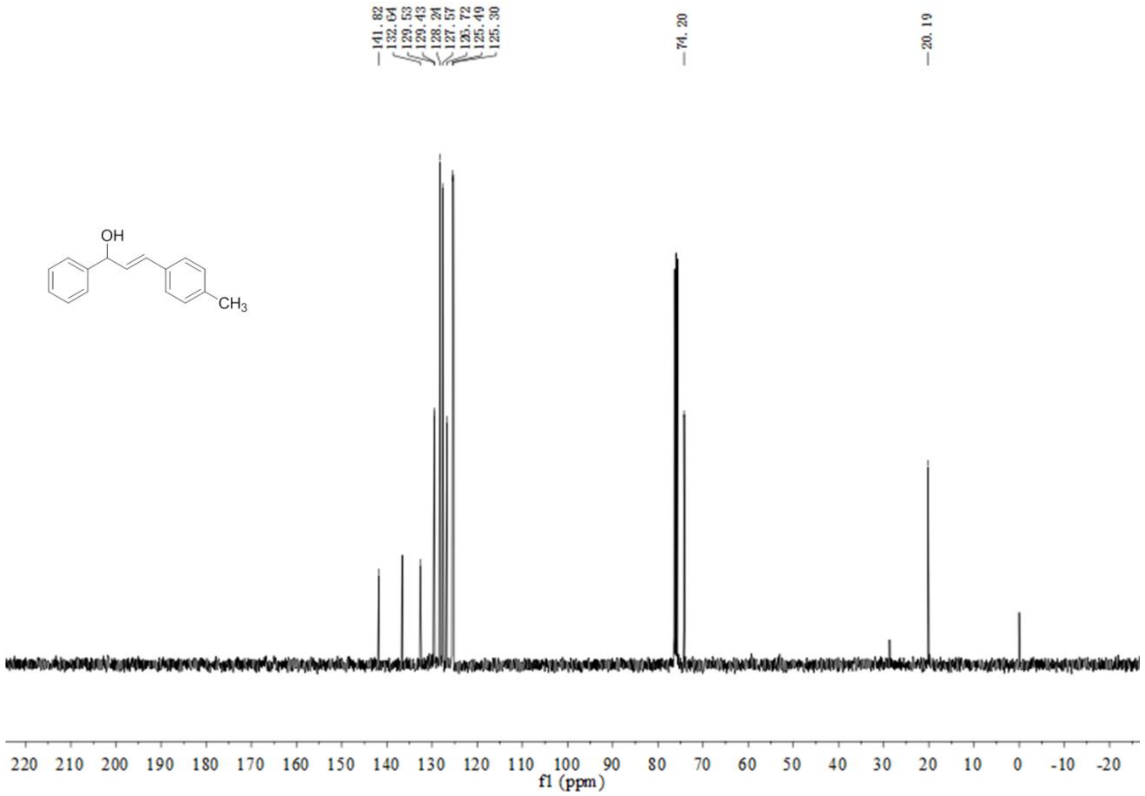

Figure S131. ^{13}C NMR spectrum for compound (E)-1-phenyl-3-(p-tolyl)prop-2-en-1-ol(**6f**)

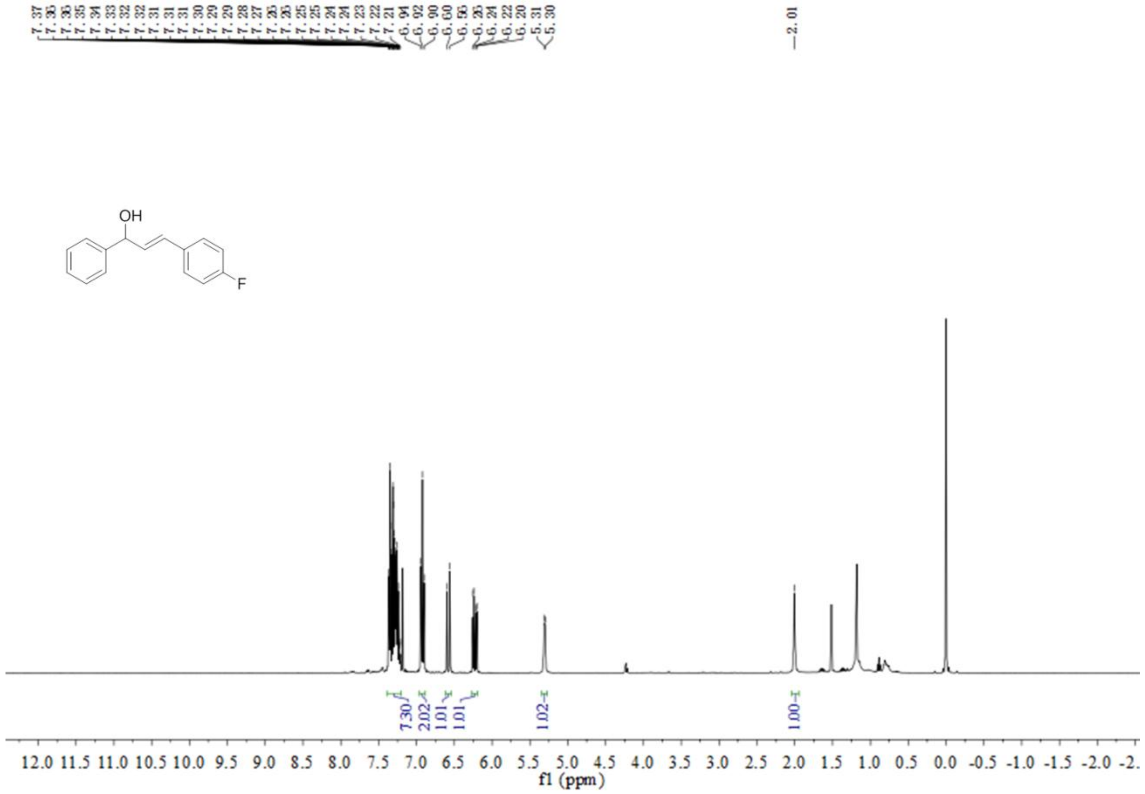

Figure S132. ^{1}H NMR spectrum for compound (E)-3-(4-fluorophenyl)-1-phenylprop-2-en-1-ol(**6g**).

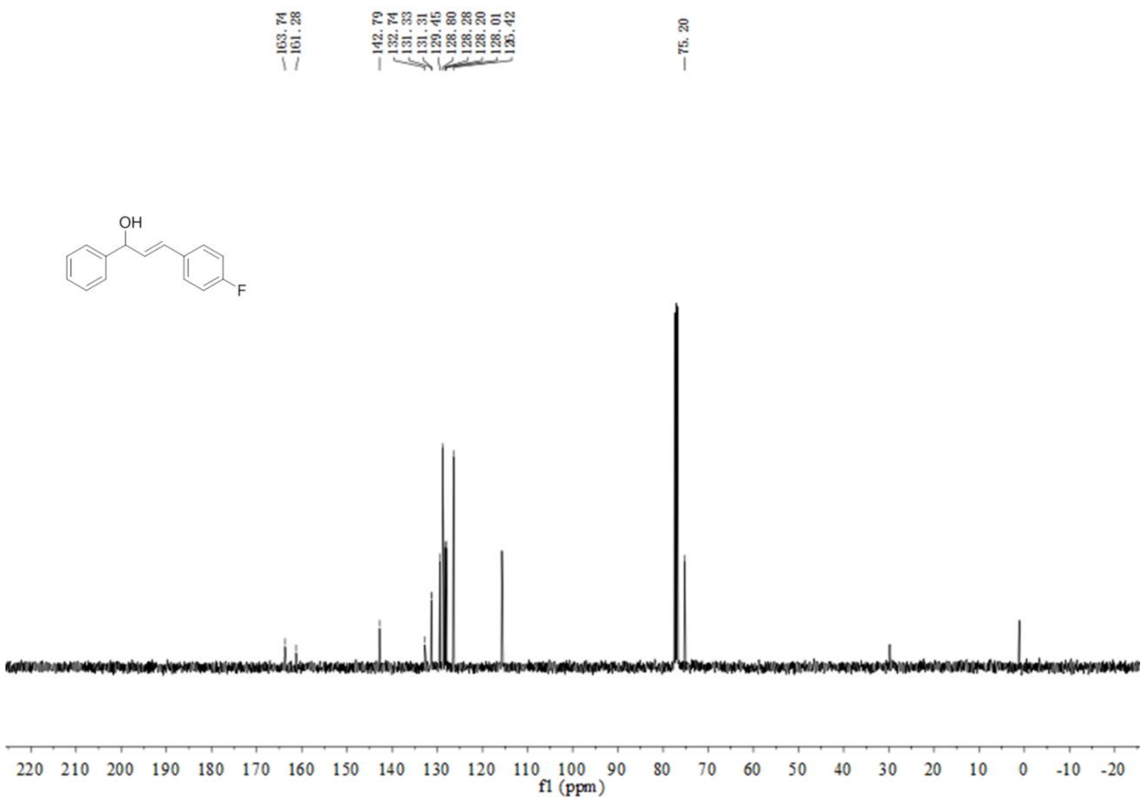

Figure S133. ^{13}C NMR spectrum for compound (E)-3-(4-fluorophenyl)-1-phenylprop-2-en-1-ol(**6g**).

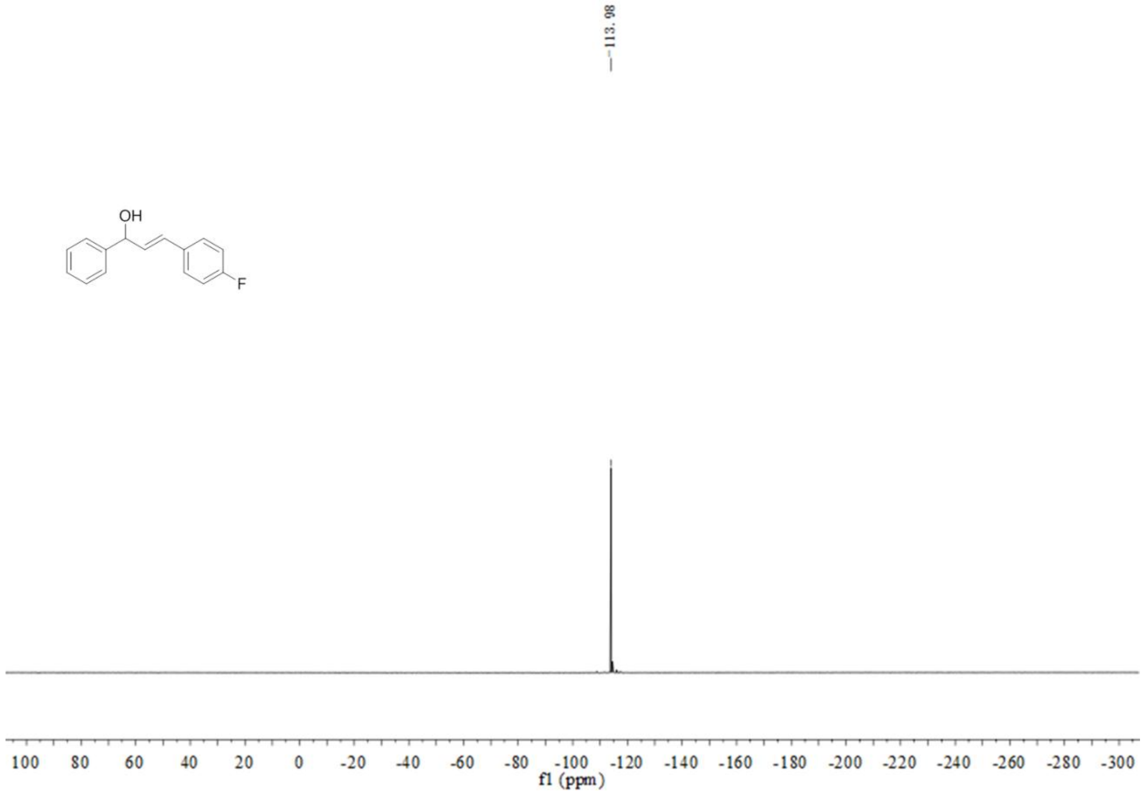

Figure S134. ^{19}F NMR spectrum for compound (E)-3-(4-fluorophenyl)-1-phenylprop-2-en-1-ol(**6g**).

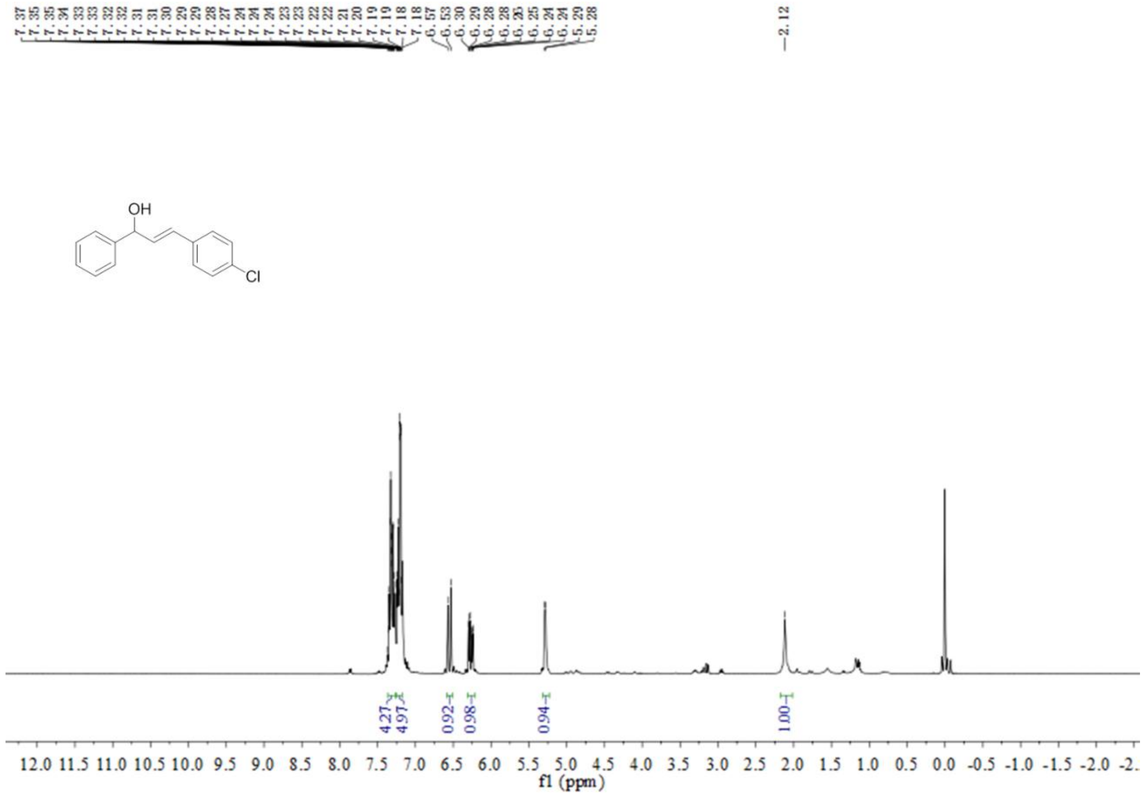

Figure S135. ^{1}H NMR spectrum for compound (E)-3-(4-chlorophenyl)-1-phenylprop-2-en-1-ol(**6h**).

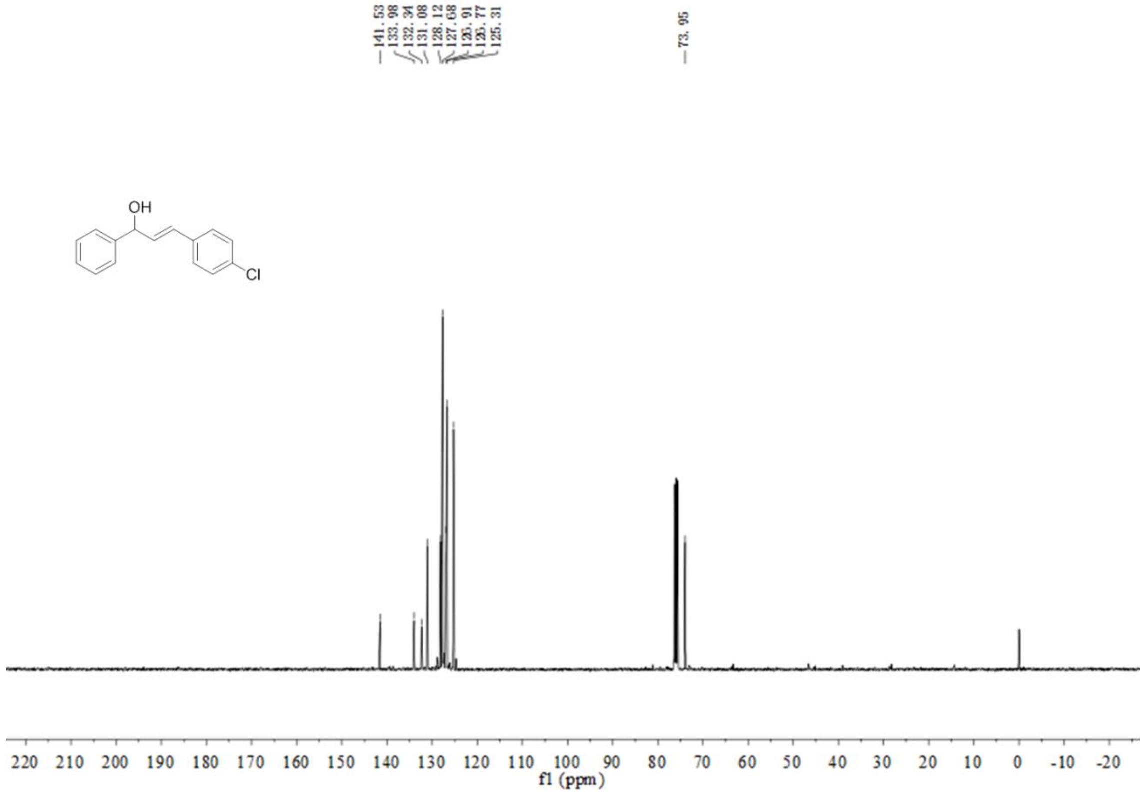

Figure S136. ^{13}C NMR spectrum for compound (E)-3-(4-chlorophenyl)-1-phenylprop-2-en-1-ol(**6h**).

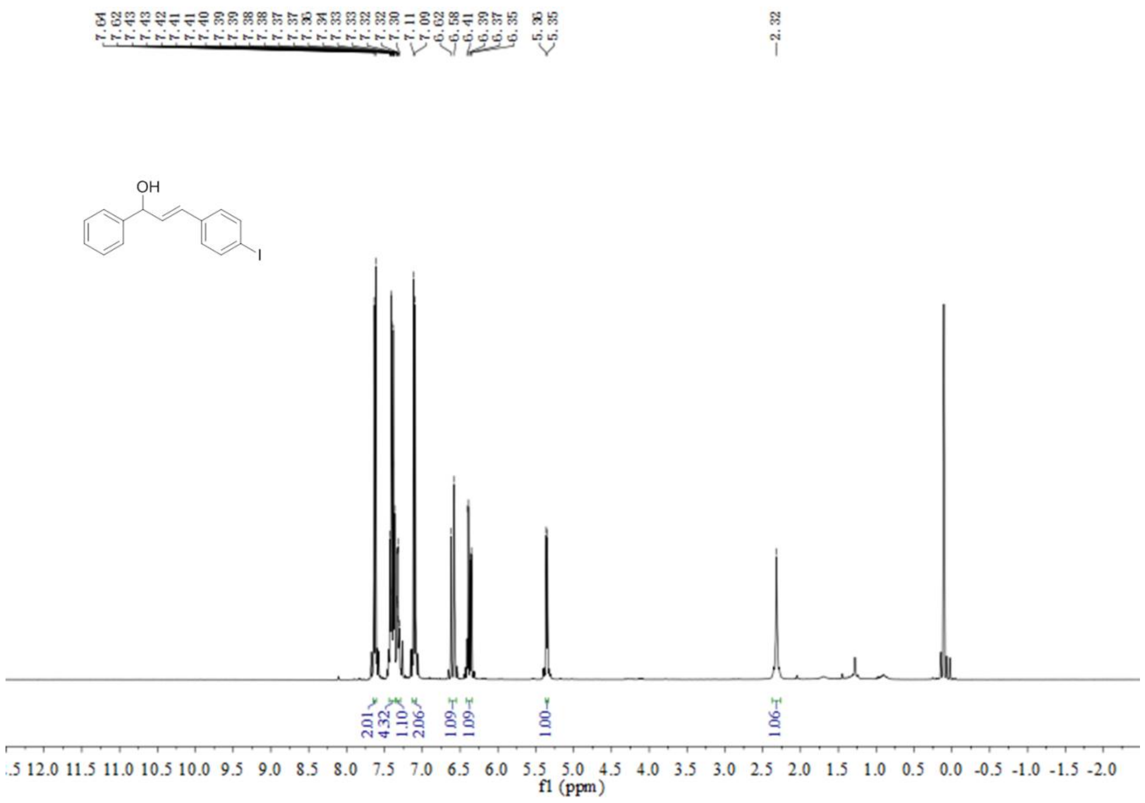

Figure S137. ^{1}H NMR spectrum for compound (E)-3-(4-iodophenyl)-1-phenylprop-2-en-1-ol(**6i**).

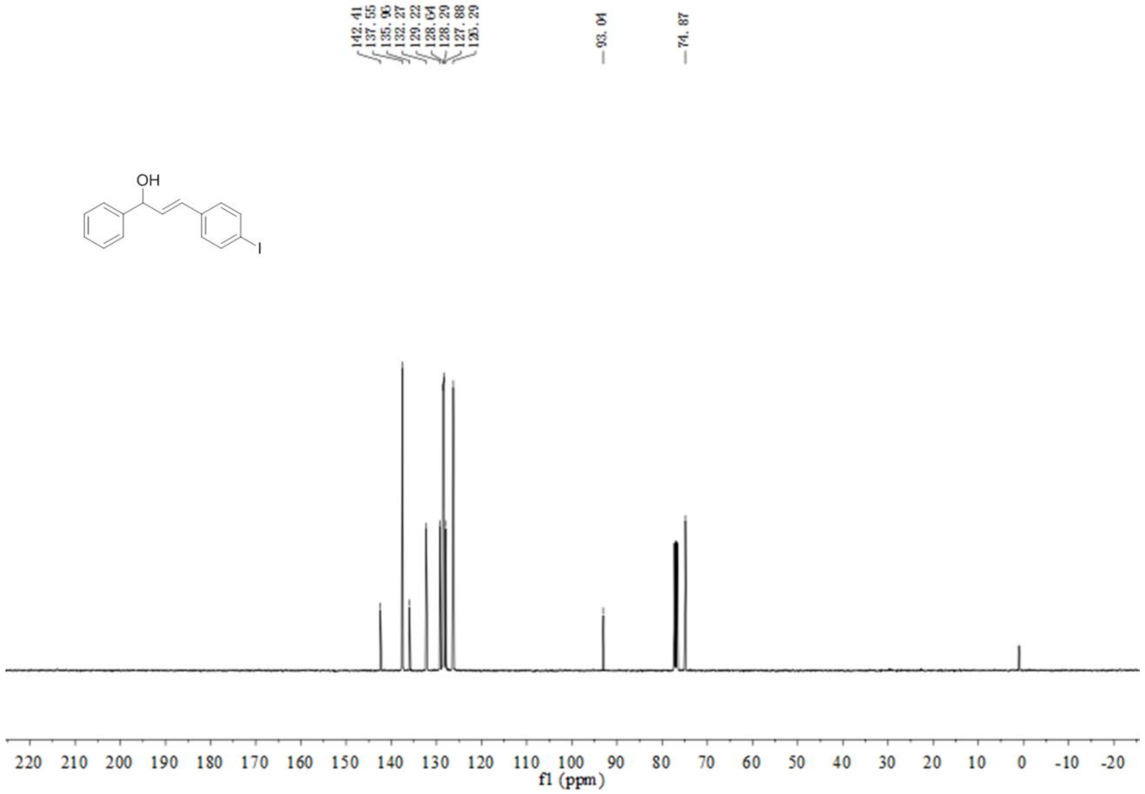

Figure S138. ^{13}C NMR spectrum for compound (E)-3-(4-iodophenyl)-1-phenylprop-2-en-1-ol(**6i**).

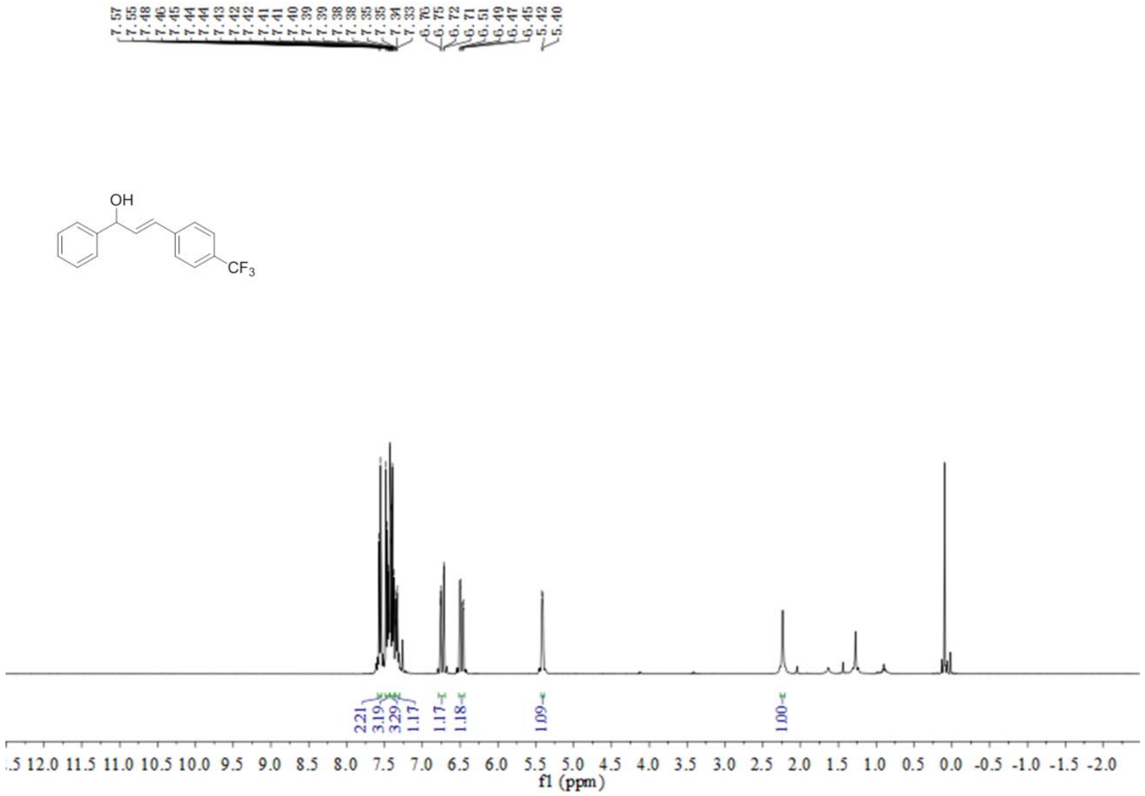

Figure S139. ^{1}H NMR spectrum for compound (E)-1-phenyl-3-(4-(trifluoromethyl)phenyl)prop-2-en-1-ol(**6j**).

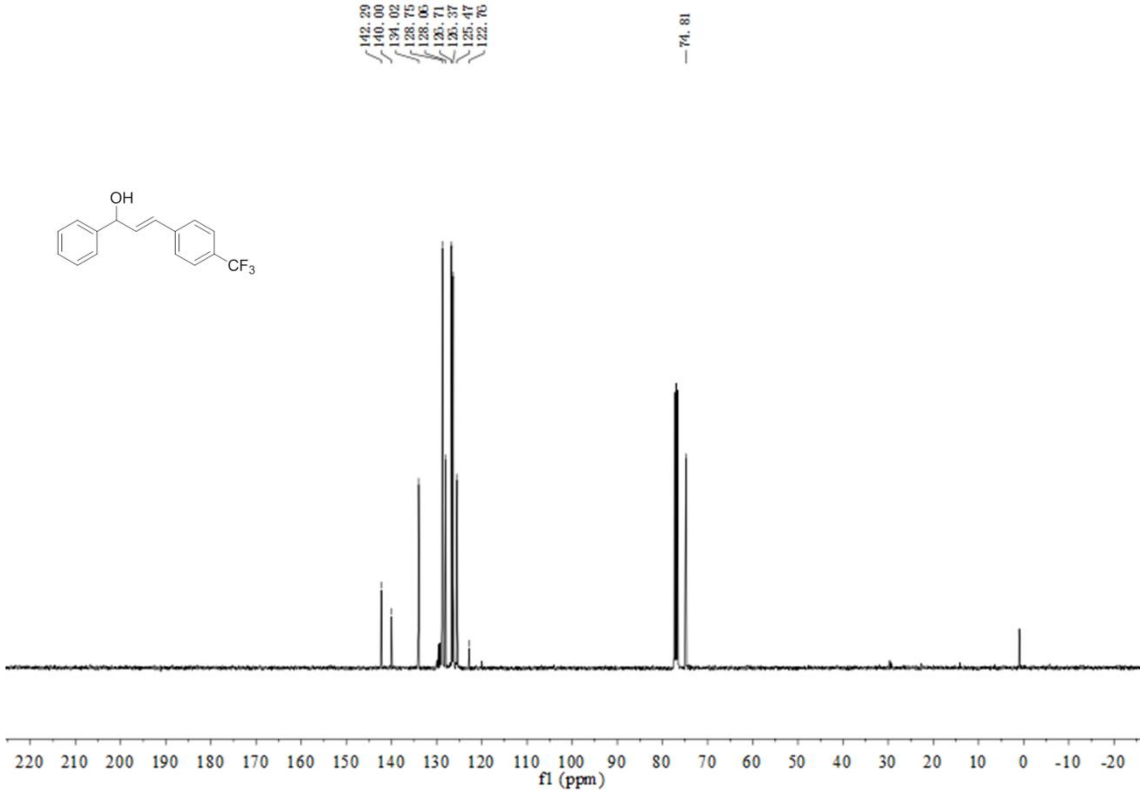

Figure S140. ^{13}C NMR spectrum for compound (E)-1-phenyl-3-(4-(trifluoromethyl)phenyl)prop-2-en-1-ol(**6j**).

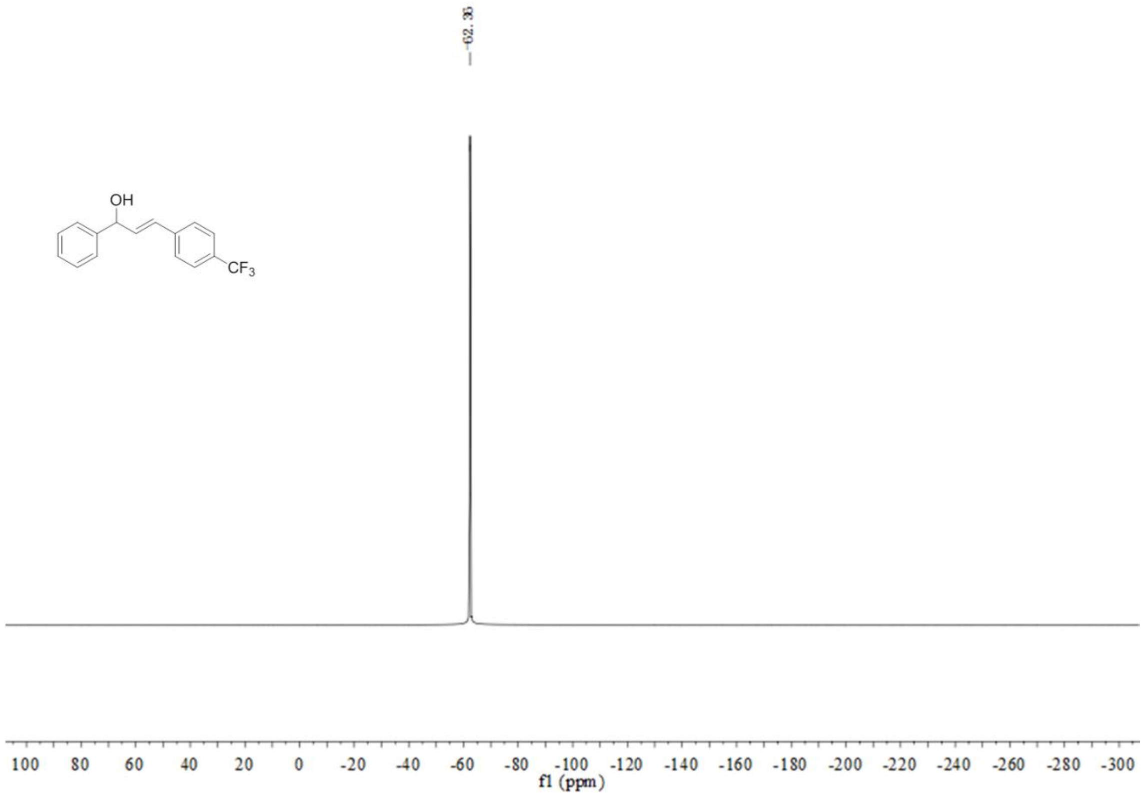

Figure S141. ^{19}F NMR spectrum for compound (E)-1-phenyl-3-(4-(trifluoromethyl)phenyl)prop-2-en-1-ol(**6j**).

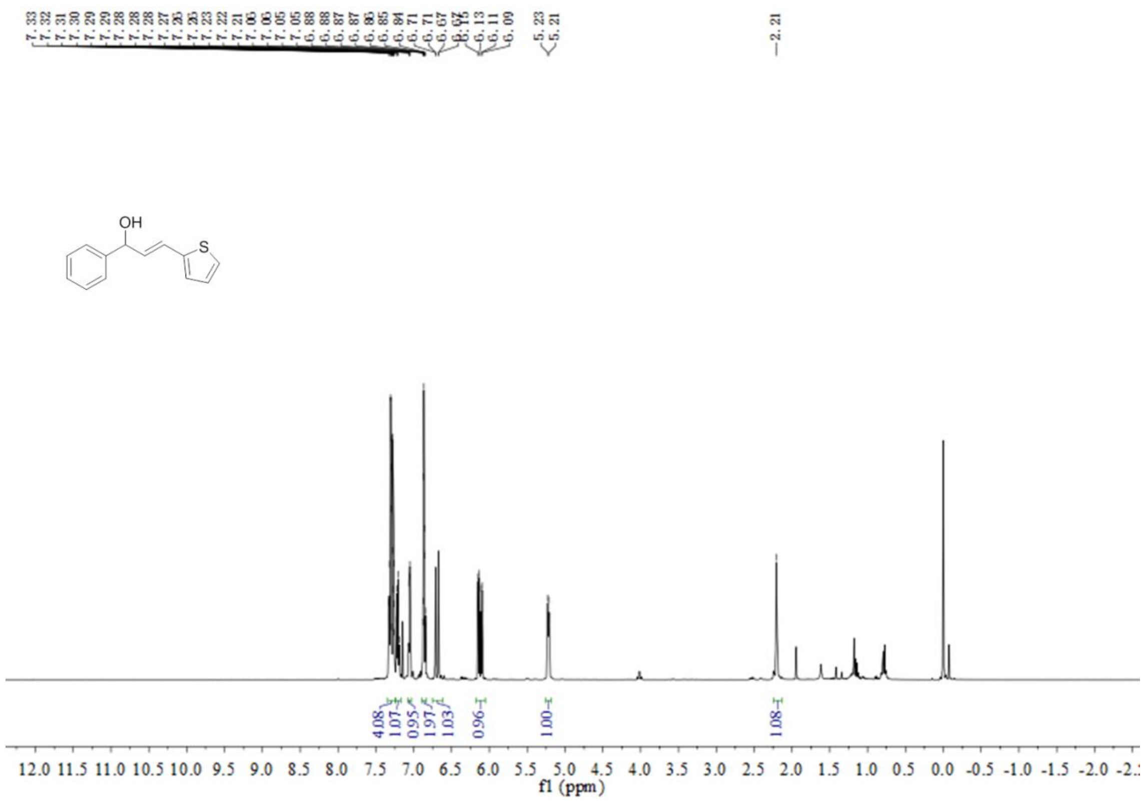

Figure S142. ^{1}H NMR spectrum for compound (E)-1-phenyl-3-(thiophen-2-yl)prop-2-en-1-ol(**6k**).

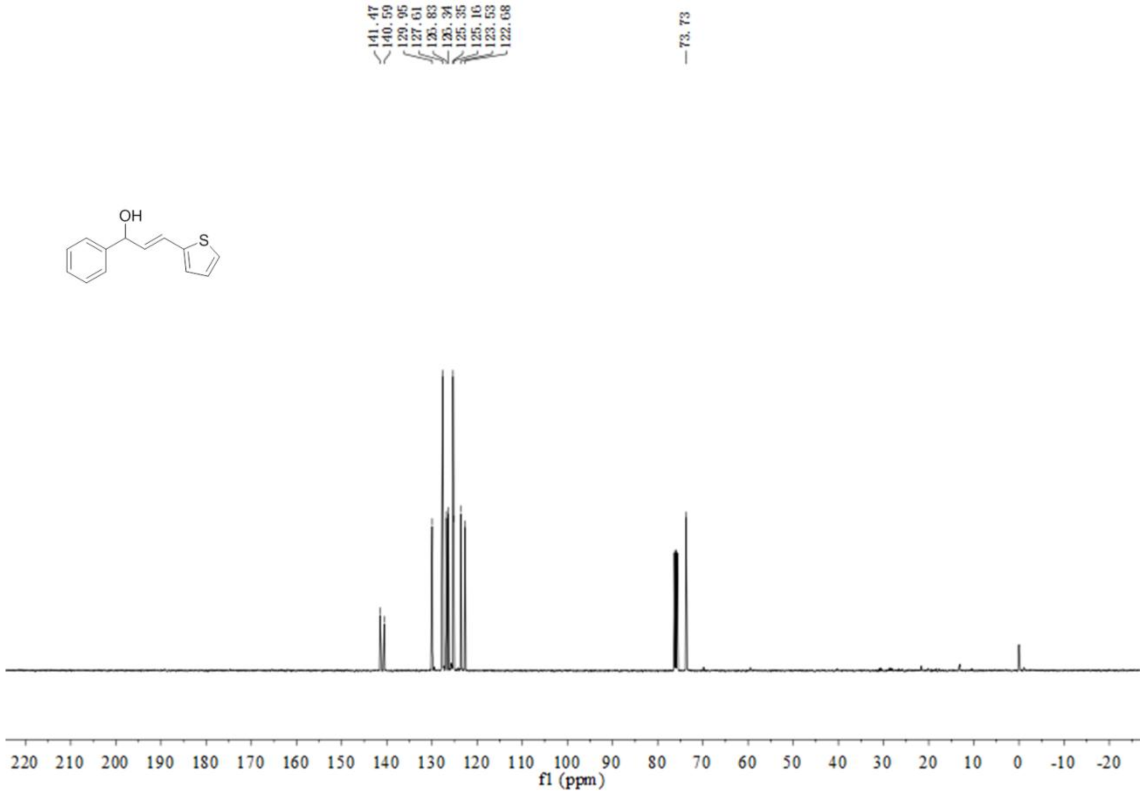

Figure S143. ^{13}C NMR spectrum for compound (E)-1-phenyl-3-(thiophen-2-yl)prop-2-en-1-ol(**6k**).

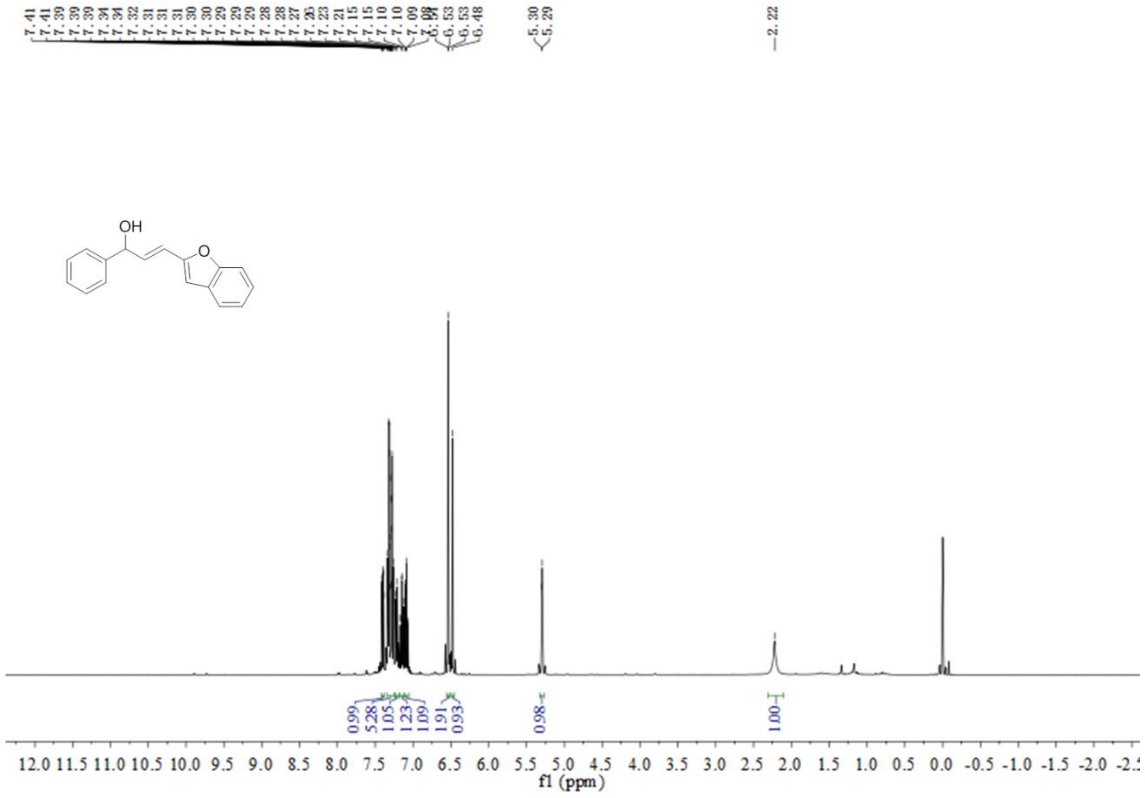

Figure S144. ^{1}H NMR spectrum for compound (E)-3-(benzofuran-2-yl)-1-phenylprop-2-en-1-ol(**6l**)

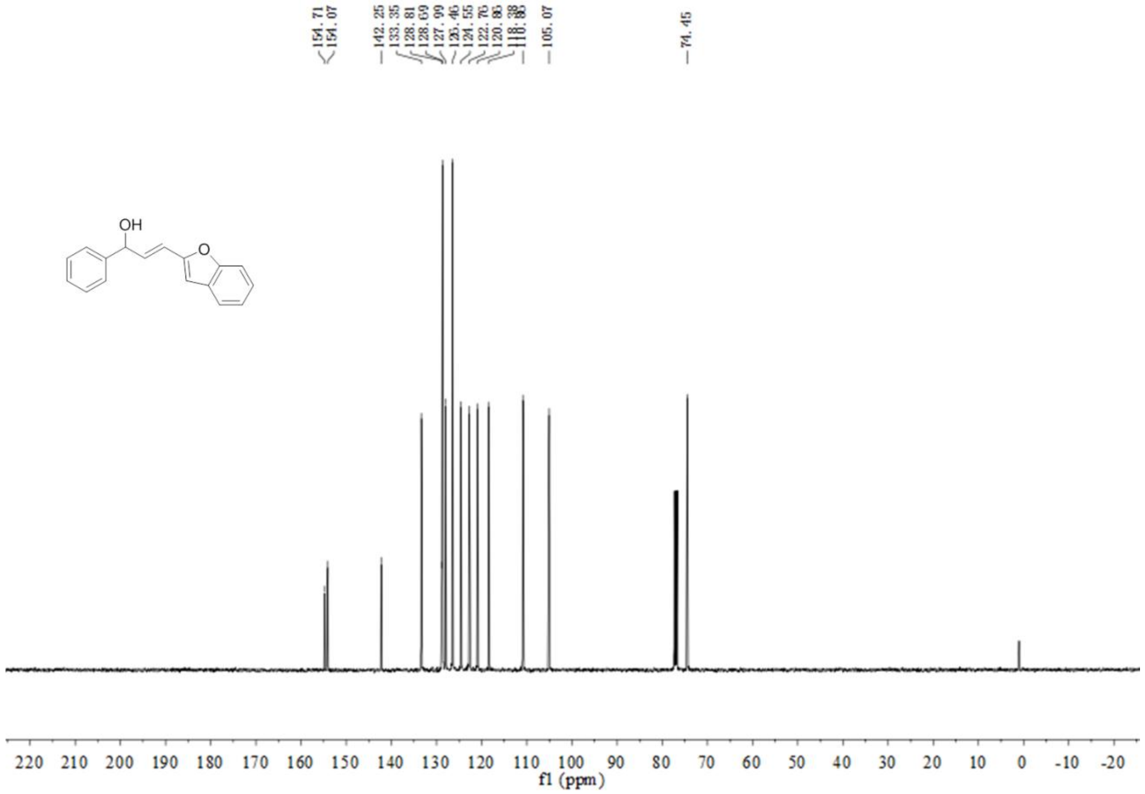

Figure S145. ^{13}C NMR spectrum for compound (E)-3-(benzofuran-2-yl)-1-phenylprop-2-en-1-ol(**6l**).

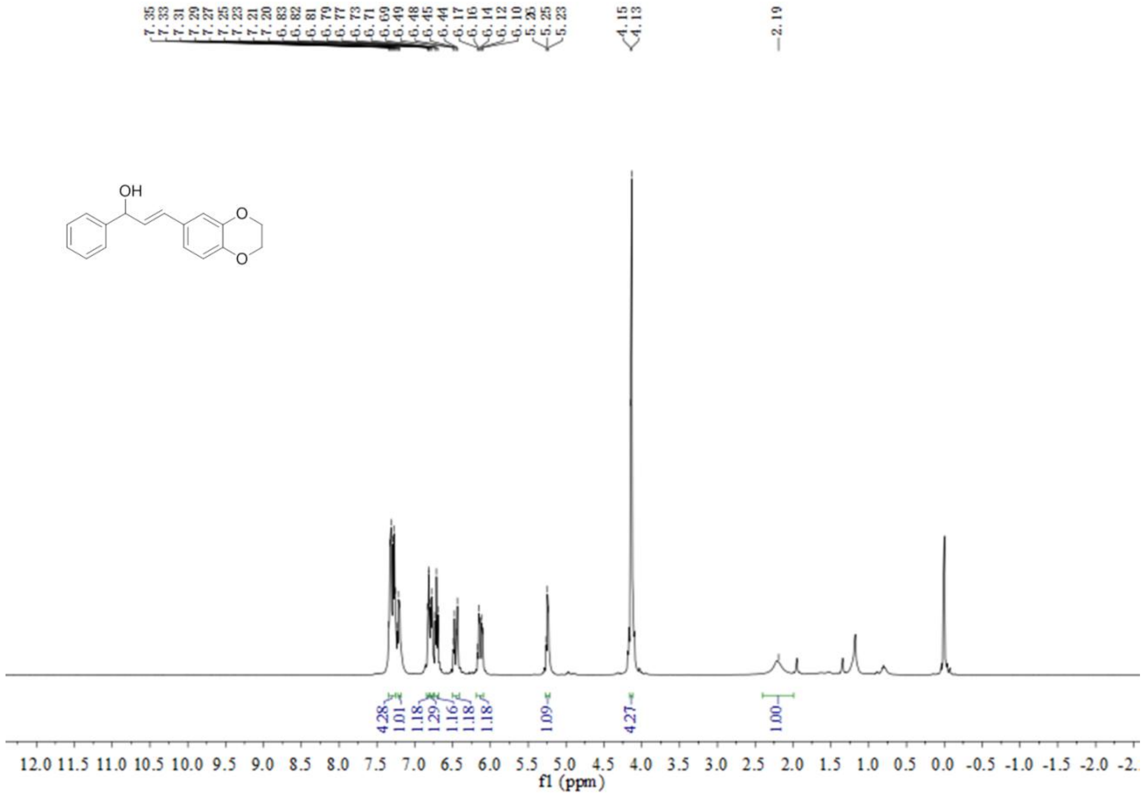

Figure S146. ^{1}H NMR spectrum for compound
(E)-3-(2,3-dihydrobenzo[b][1,4]dioxin-6-yl)-1-phenylprop-2-en-1-ol(**6m**)

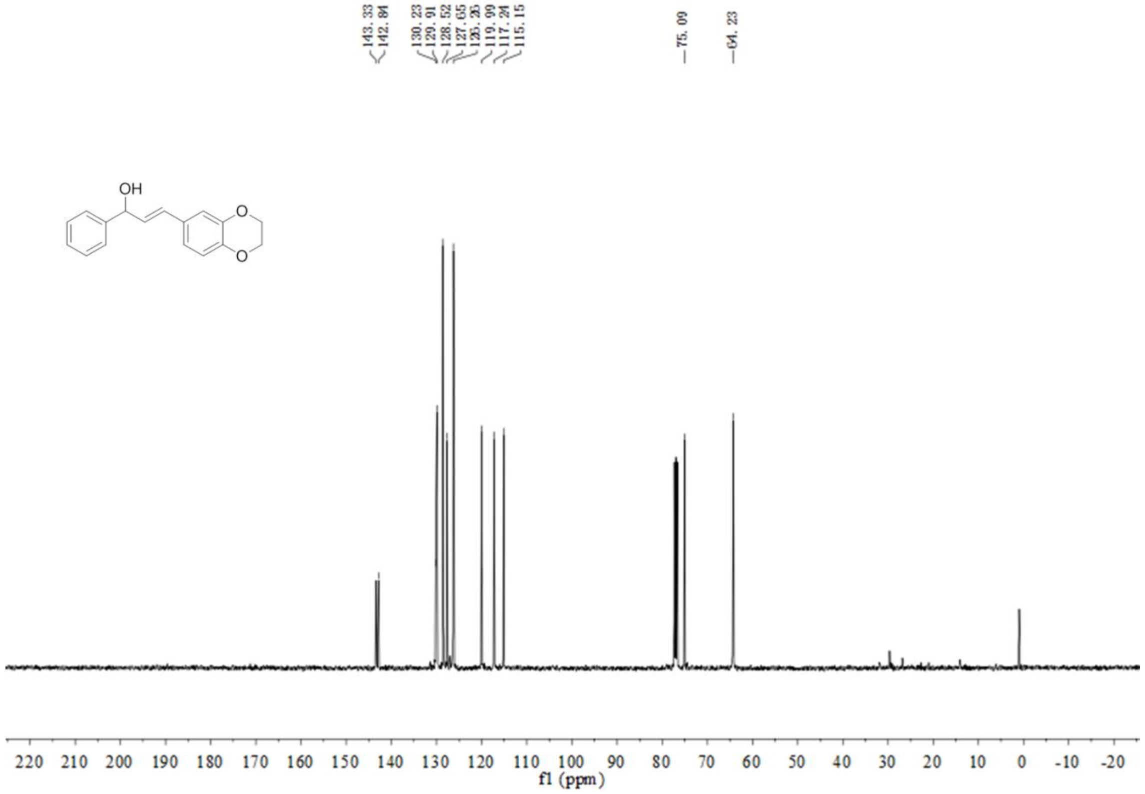

Figure S147. ^{13}C NMR spectrum for compound
(E)-3-(2,3-dihydrobenzo[b][1,4]dioxin-6-yl)-1-phenylprop-2-en-1-ol(**6m**).

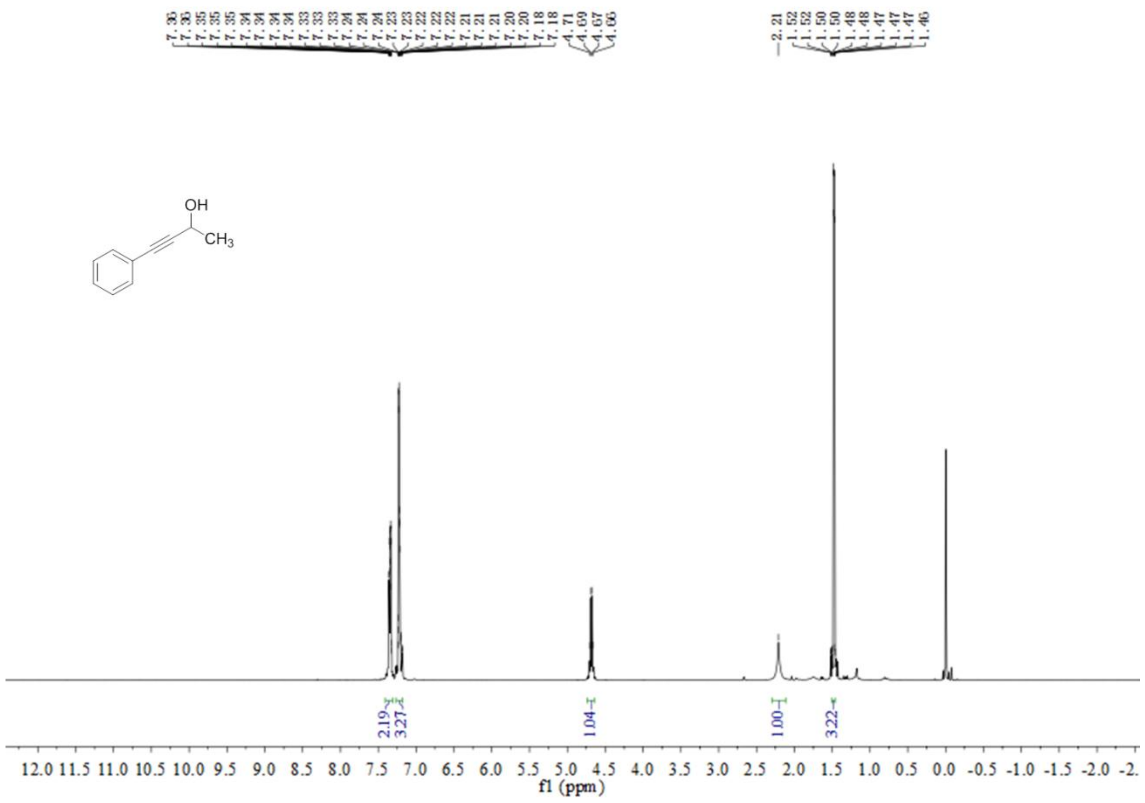

Figure S148. ^{1}H NMR spectrum for compound 4-phenylbut-3-yn-2-ol(**6n**)

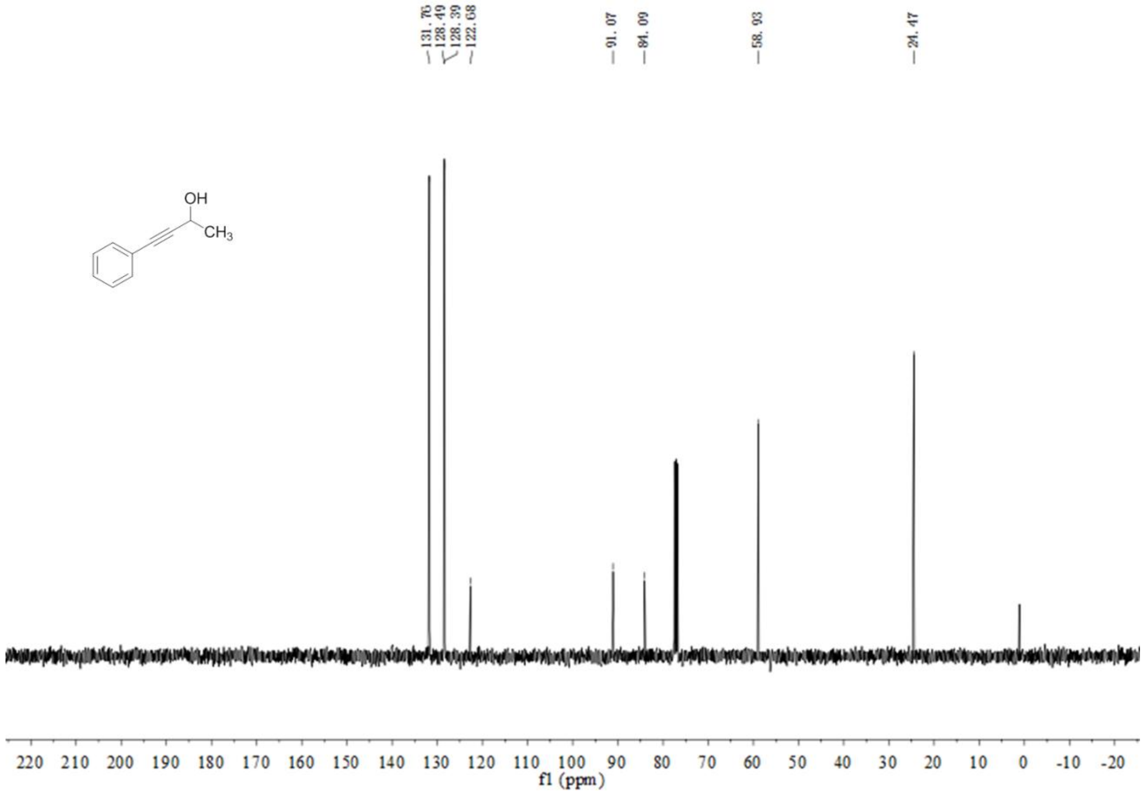

Figure S149. ^{13}C NMR spectrum for compound 4-phenylbut-3-yn-2-ol(**6n**).

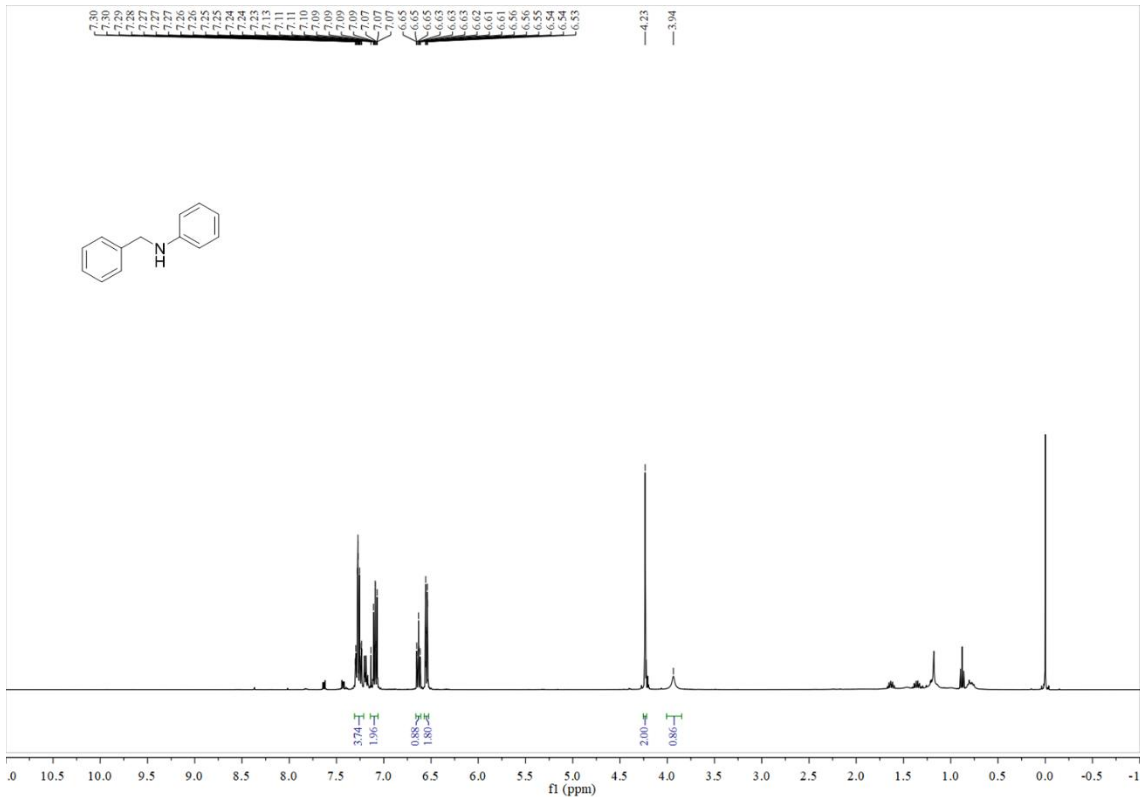

Figure S150. ¹H NMR spectrum for compound N-benzylaniline(**8a**)

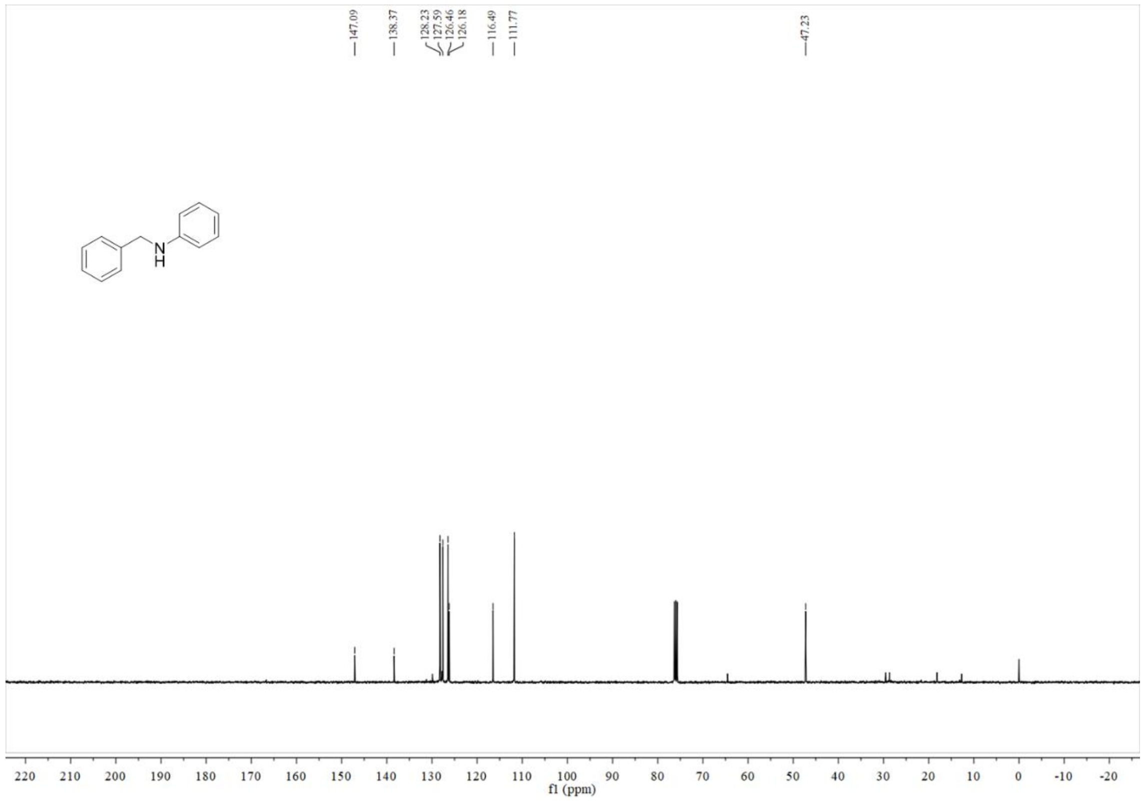

Figure S151. ^{13}C NMR spectrum for compound N-benzylaniline(**8a**)

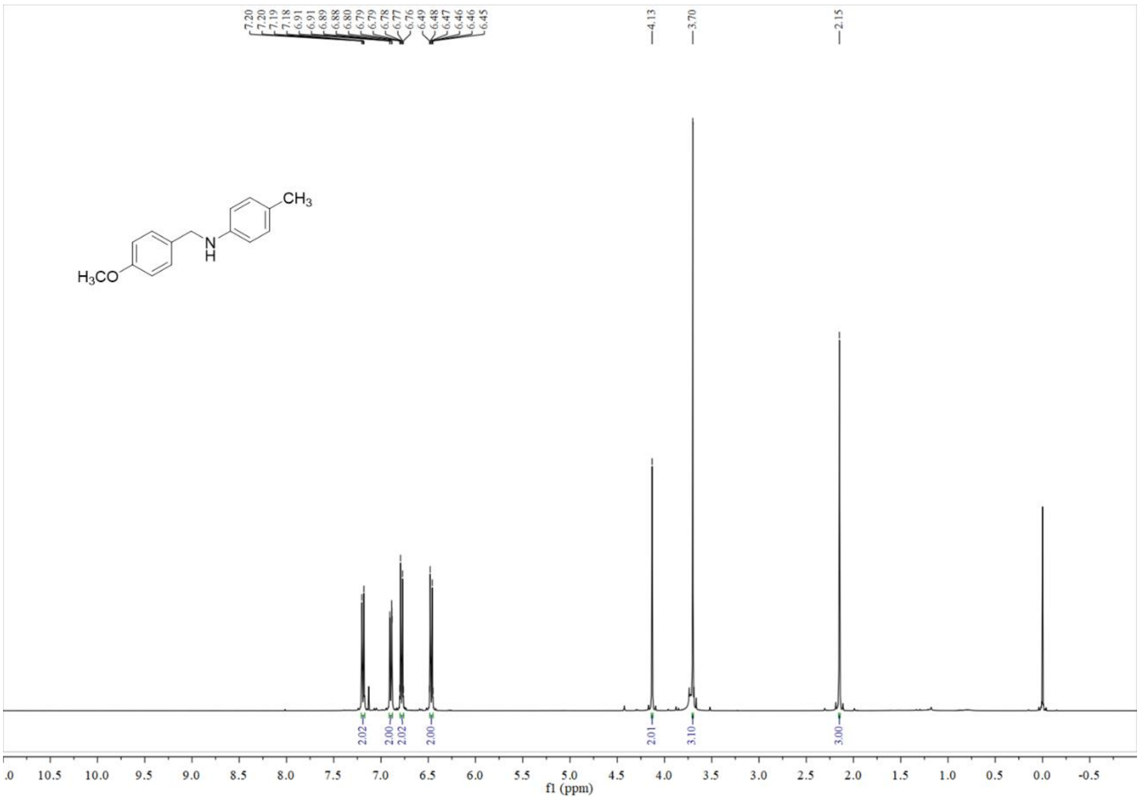

Figure S152. ^{1}H NMR spectrum for compound N-(4-methoxybenzyl)-4-methylaniline(**8b**).

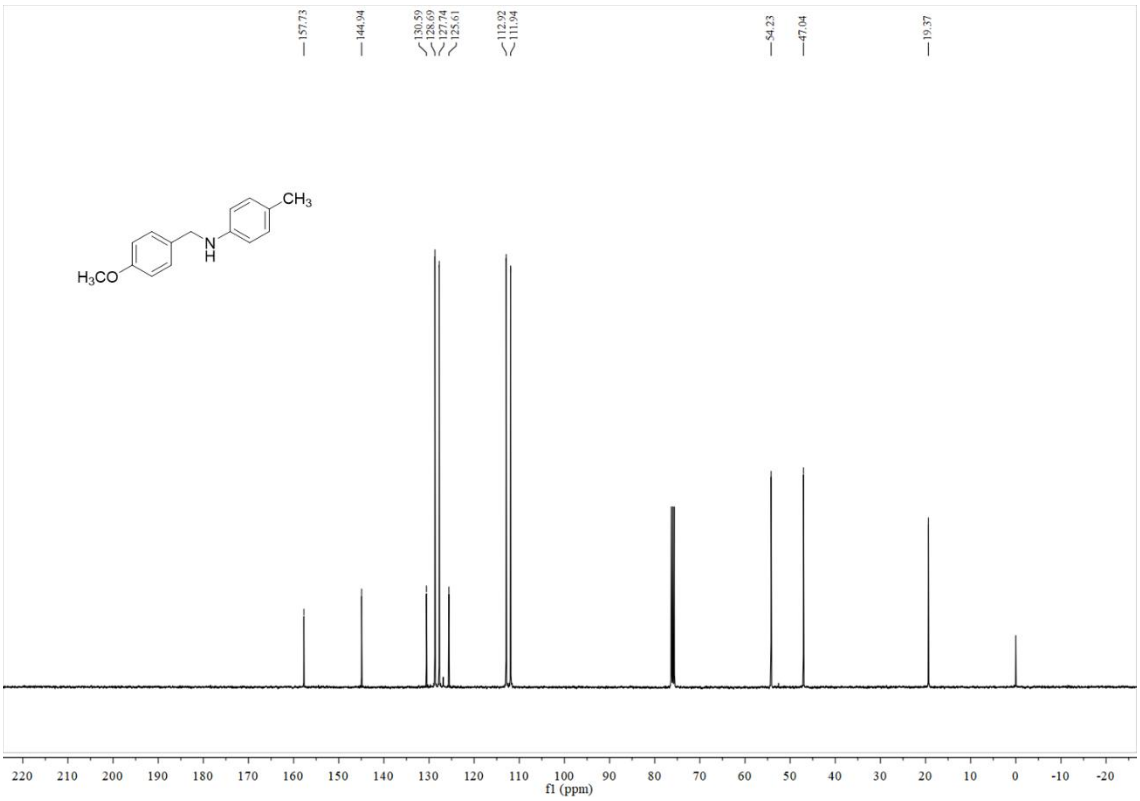

Figure S153. ^{13}C NMR spectrum for compound N-(4-methoxybenzyl)-4-methylaniline(**8b**).

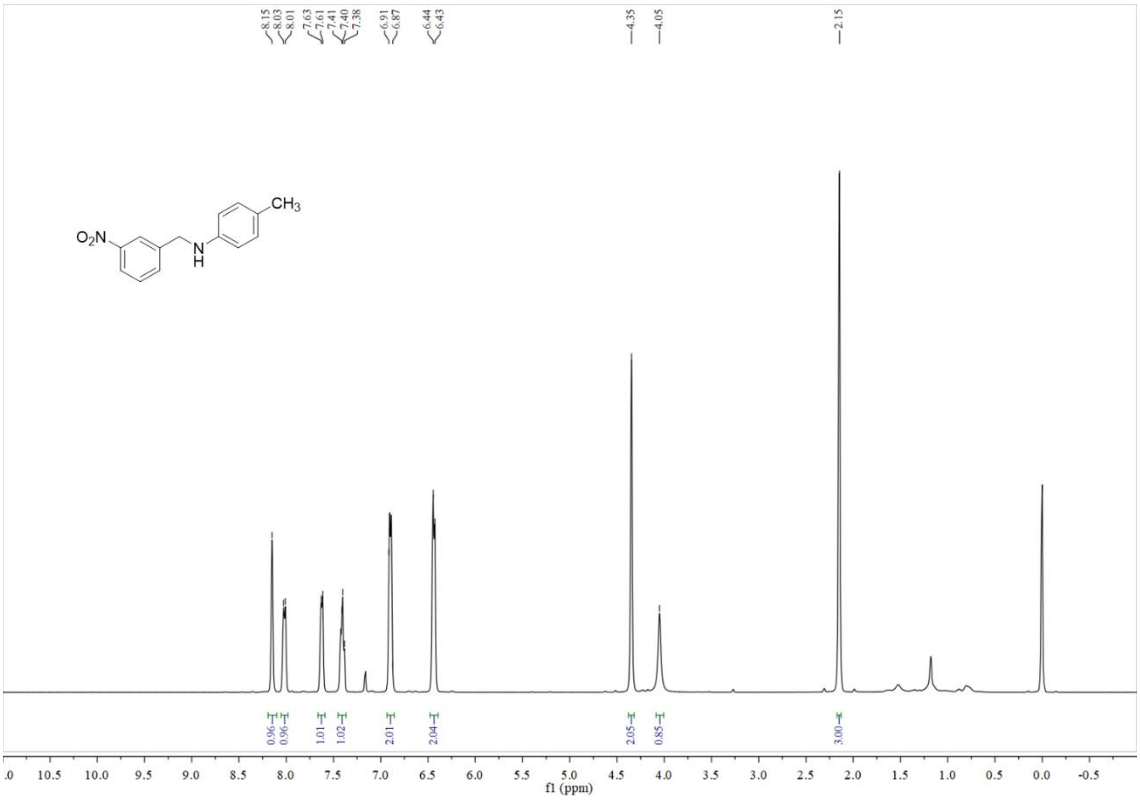

Figure S154. ^{1}H NMR spectrum for compound 4-methyl-N-(3-nitrobenzyl)aniline(**8c**).

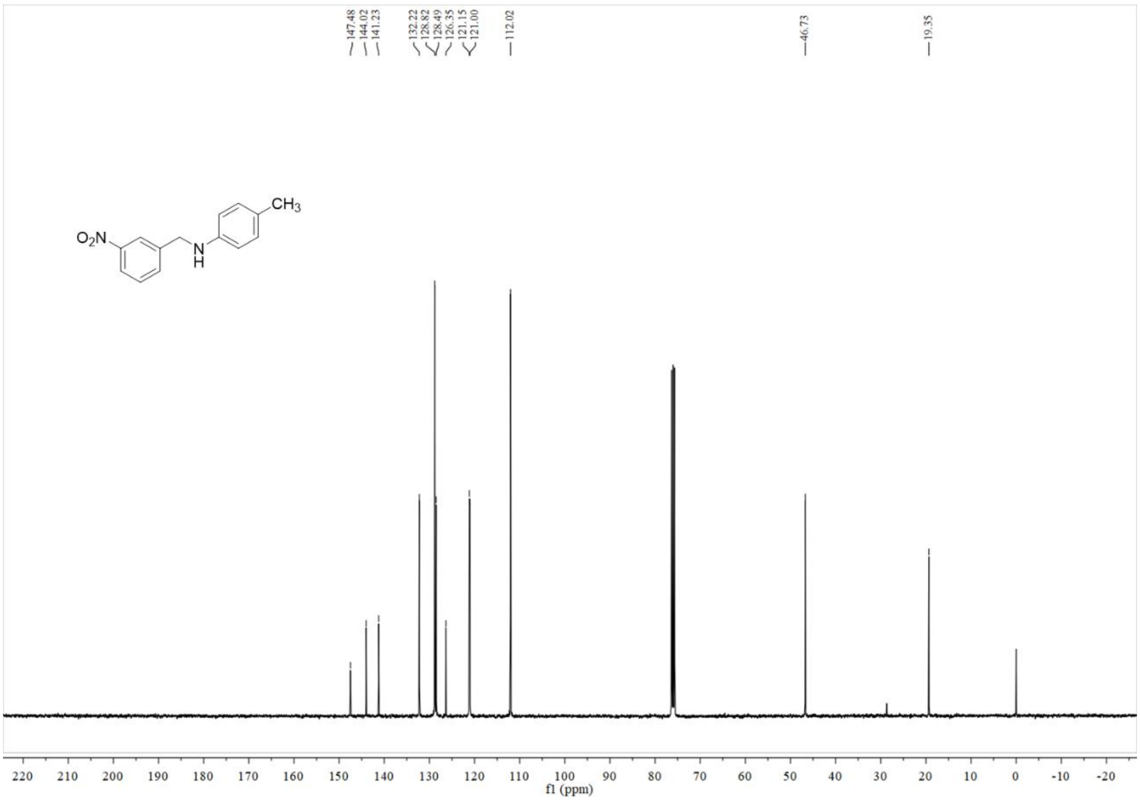

Figure S155. ^{13}C NMR spectrum for compound 4-methyl-N-(3-nitrobenzyl)aniline(**8c**).

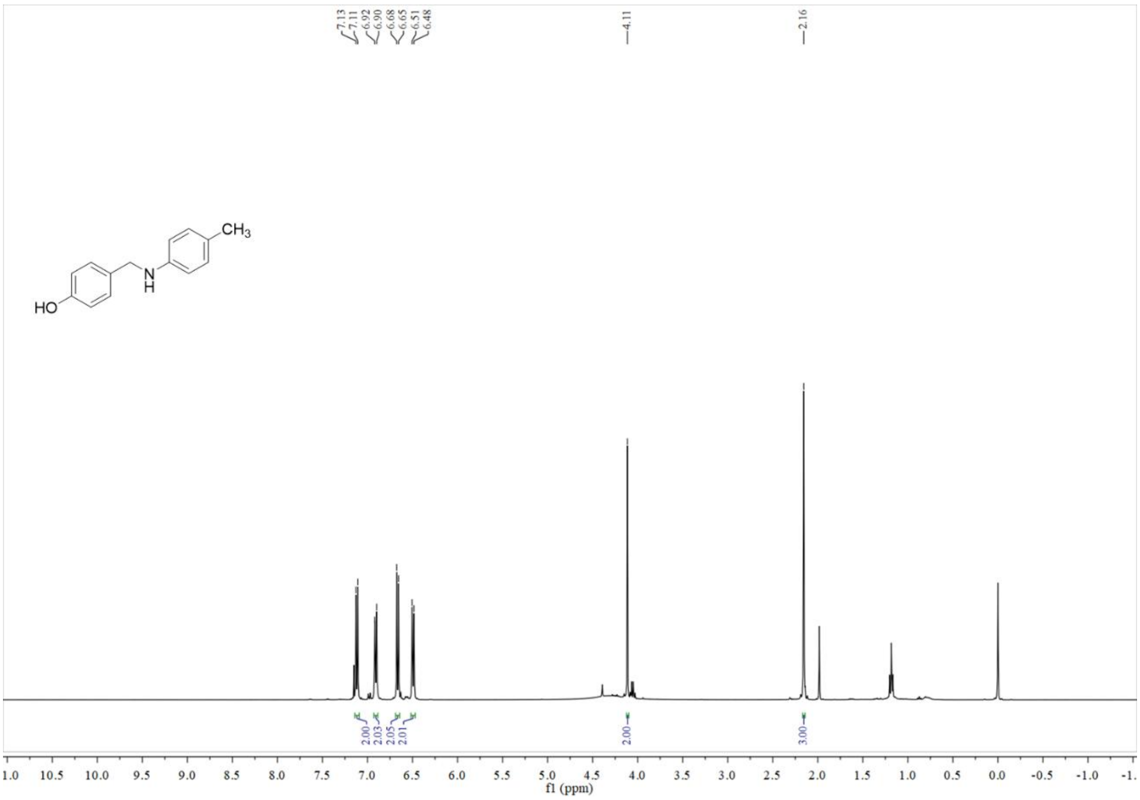

Figure S156. ^{1}H NMR spectrum for compound 4-((p-tolylamino)methyl)phenol(**8d**).

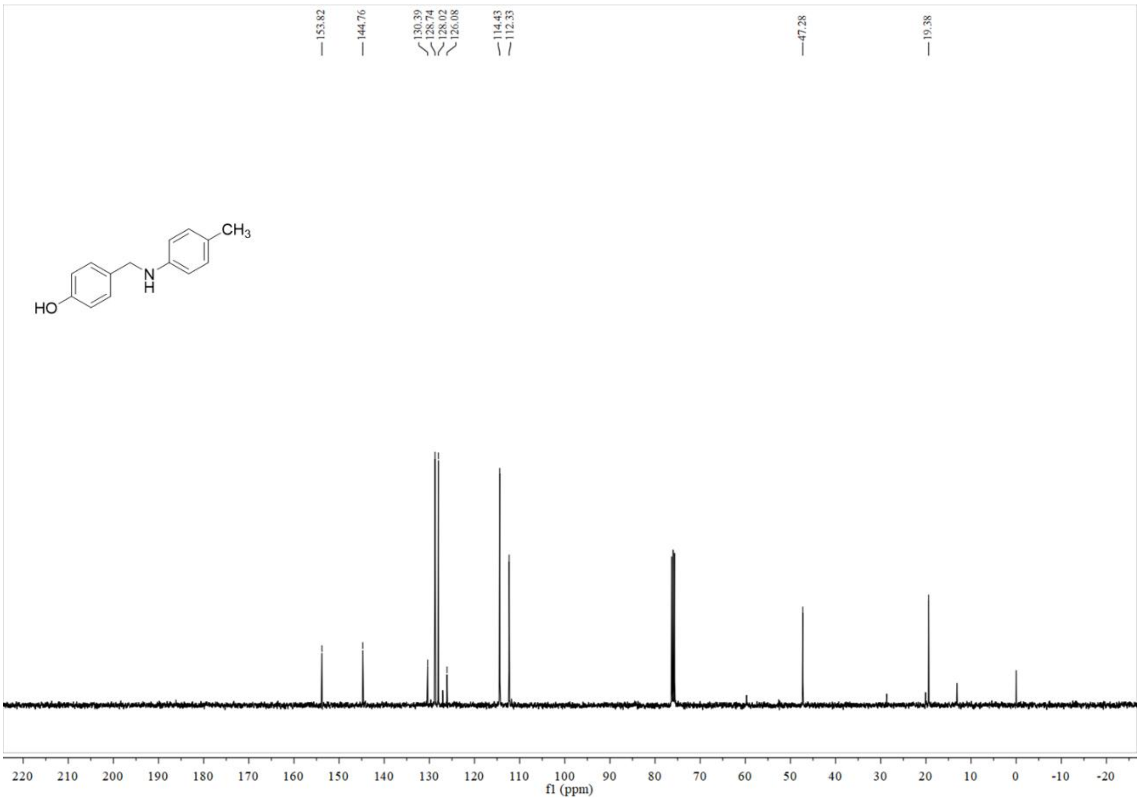

Figure S157. ^{13}C NMR spectrum for compound 4-((p-tolylamino)methyl)phenol(**8d**).

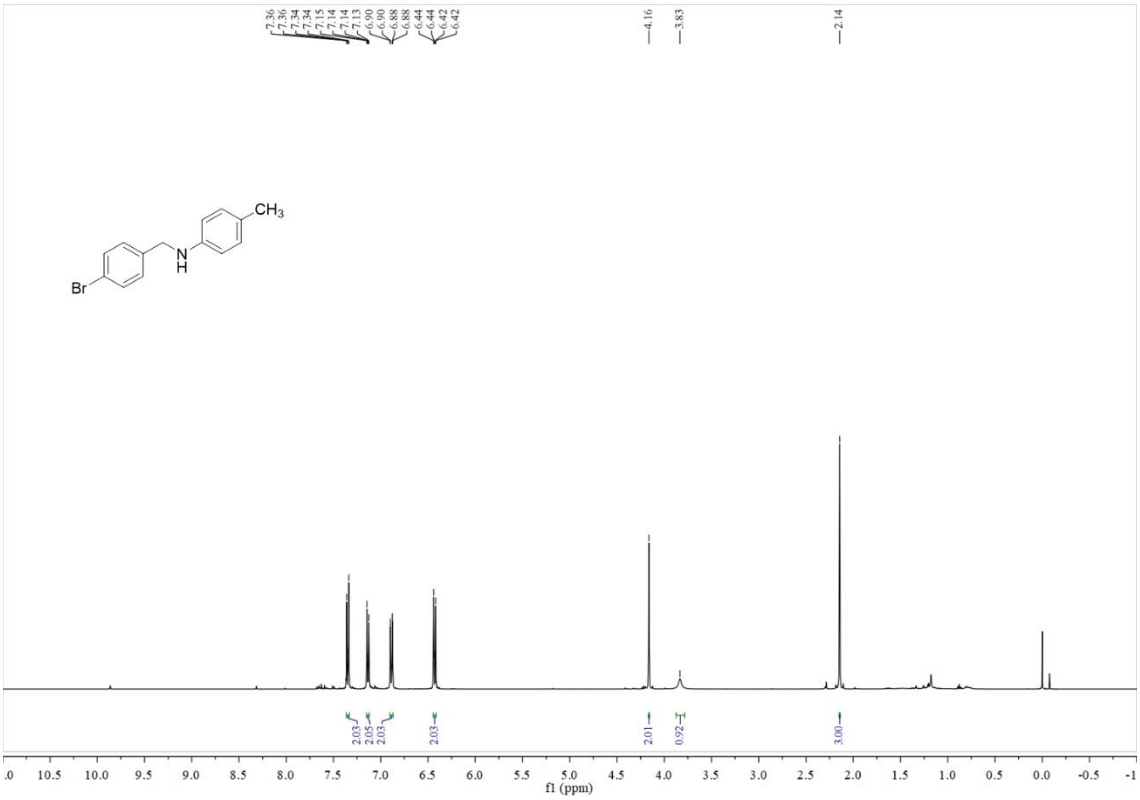

Figure S158. ^{1}H NMR spectrum for compound N-(4-bromobenzyl)-4-methylaniline(**8e**).

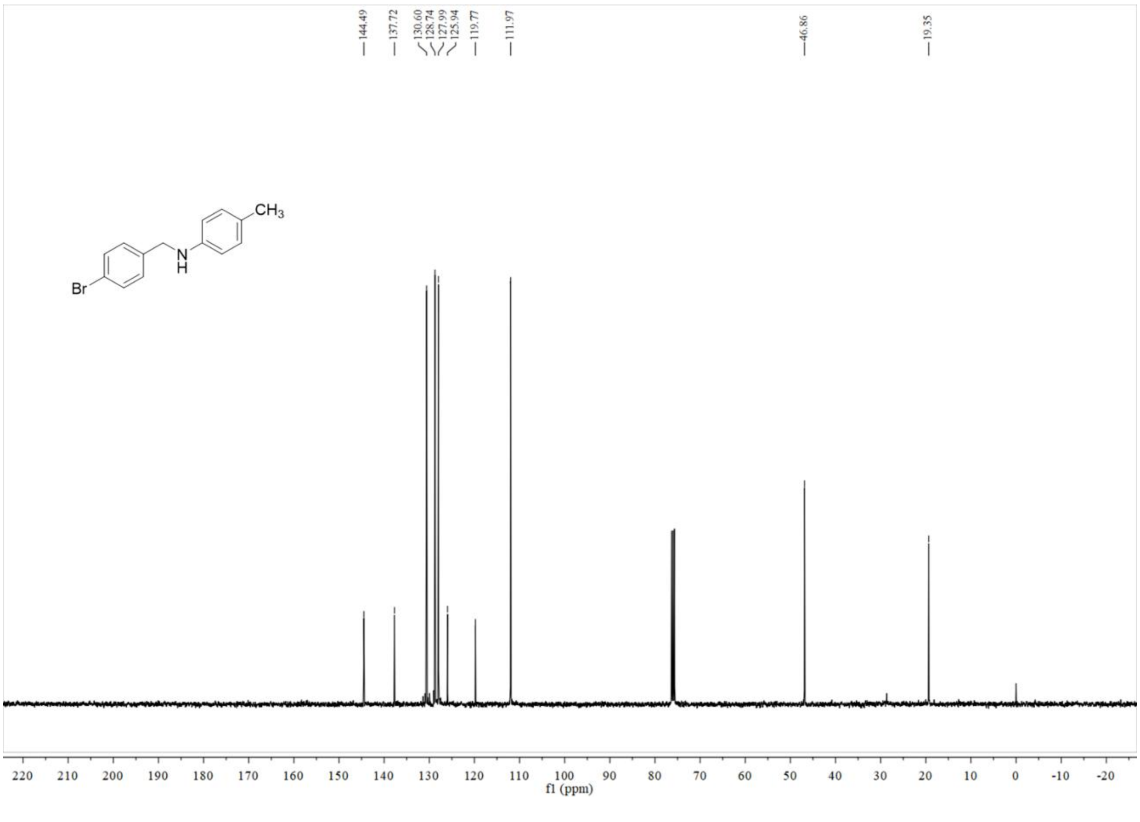

Figure S159. ^{13}C NMR spectrum for compound N-(4-bromobenzyl)-4-methylaniline(**8e**).
